# SELER: a database of super-enhancer-associated lncRNA- directed transcriptional regulation in human cancers

**DOI:** 10.1093/database/baz027

**Published:** 2019-02-26

**Authors:** Zhi-Wei Guo, Chen Xie, Kun Li, Xiang-Ming Zhai, Geng-Xi Cai, Xue-Xi Yang, Ying-Song Wu

**Affiliations:** 1 Institute of Antibody Engineering, School of Laboratory Medicine and Biotechnology, Southern Medical University, Guangzhou, China; 2 Key Laboratory of Liver Disease of Guangdong Province, The Third Affiliated Hospital of Sun Yat-sen University, Guangzhou, China; 3 Department of Breast Surgery, The First People’s Hospital of Foshan, Foshan, China

## Abstract

Super-enhancers (SEs) are enriched with a cluster of mediator binding sites, which are major contributors to cell-type-specific gene expression. Currently, a large quantity of long non-coding RNAs has been found to be transcribed from or to interact with SEs, which constitute super-enhancer associated long non-coding RNAs (SE-lncRNAs). These SE-lncRNAs play essential roles in transcriptional regulation through controlling SEs activity to regulate a broad range of physiological and pathological processes, especially tumorigenesis. However, the pathological functions of SE-lncRNAs in tumorigenesis are still obscure. In this paper, we characterized 5056 SE-lncRNAs and their associated genes by analysing 102 SE data sets. Then, we analysed their expression profiles and prognostic information derived from 19 cancer types to identify cancer-related SE-lncRNAs and to explore their potential functions. In total, 436 significantly differentially expressed SE-lncRNAs and 2035 SE-lncRNAs with high prognostic values were identified. Additionally, 3935 significant correlations between SE-lncRNAs and their regulatory genes were further validated by calculating their correlation coefficients in each cancer type. Finally, the SELER database incorporating the aforementioned data was provided for users to explore their physiological and pathological functions to comprehensively understand the blocks of living systems.

## Introduction

Super-enhancers (SEs) are enriched with clustered mediator binding sites and a variety of chromatin signatures, such as H3K4me1, H3K4me3, H3K27ac and P300 acetyltransferase, which play essential roles in regulating gene expression (1–3). The enriched chromatin signature could reflect the regulatory roles of genomic regions; therefore, they could be applied to identify SEs (2). SEs exist in a wide range of mammalian cells, and they can increase gene transcription over large genomic distances to regulate gene expression and to determine cell-type specificity (2, 4). More importantly, SEs are closely related with a variety of diseases, especially human cancers (5, 6). For instance, SEs have been shown to affect the invasion and metastasis of neuroendocrine tumor cells by controlling MET expression (7). As SEs play important roles in controlling gene expression to regulate cellular physiological and pathological processes, it is necessary to reveal their underlying regulatory mechanisms.

Currently, pervasive transcriptions of the human genome have been documented, and most of them are non-coding transcripts, especially long non-coding RNAs (lncRNAs), which are endogenous non-coding RNAs that are longer than 200 nucleotides (nt) (8, 9). LncRNAs have been proven to play essential roles in regulating the expression of genes that affect numerous biological processes, such as the cell cycle and apoptosis (10, 11). Recent discoveries have revealed that lncRNAs transcribed from or that are interact with SE regulatory elements constitute a specific type of lncRNAs, which were termed as super-enhancer associated lncRNAs (SE-lncRNAs) (12, 13). SE-lncRNAs regulate gene expression by affecting gene promoter activity (14–16). Although SE-lncRNAs significantly contributed to gene expression, the systematic identification of SE-lncRNAs and their regulated genes still lacks comprehensive recognition.

SE-lncRNAs have been proven to play essential roles in regulating physiological and pathological processes, especially tumorigenesis. For instance, SE-lncRNA cardiac mesoderm enhancer-associated noncoding RNA (CARMEN) is upregulated during the development process and it controls cardiac precursor cell differentiation (15). Moreover, upperhand can regulate heart development by affecting hand2 expression levels (14). In addition to regulating physiological processes, SE-lncRNAs are closely correlated with tumorigenesis (16). SE-lncRNA CCAT1-L promotes cancer growth by forming enhancer loops to activate the MYC expression (16). Despite of their critical roles in various physiological and pathological processes, their potential roles in human cancers still lack comprehensive investigation.

To systematically explore the potential regulatory roles of SE-lncRNAs in tumor progression, we developed SE-lncRNA directed transcriptional regulation in the human cancers (SELER) database. SELER first identified putative SE-lncRNAs and their associated genes. More importantly, their potential functions in cancers were further explored by analysing their expression profiles, correlation coefficient and prognostic value across 19 cancer types. Finally, SELER was built to store and display data.

## Methods

**Figure 1:**
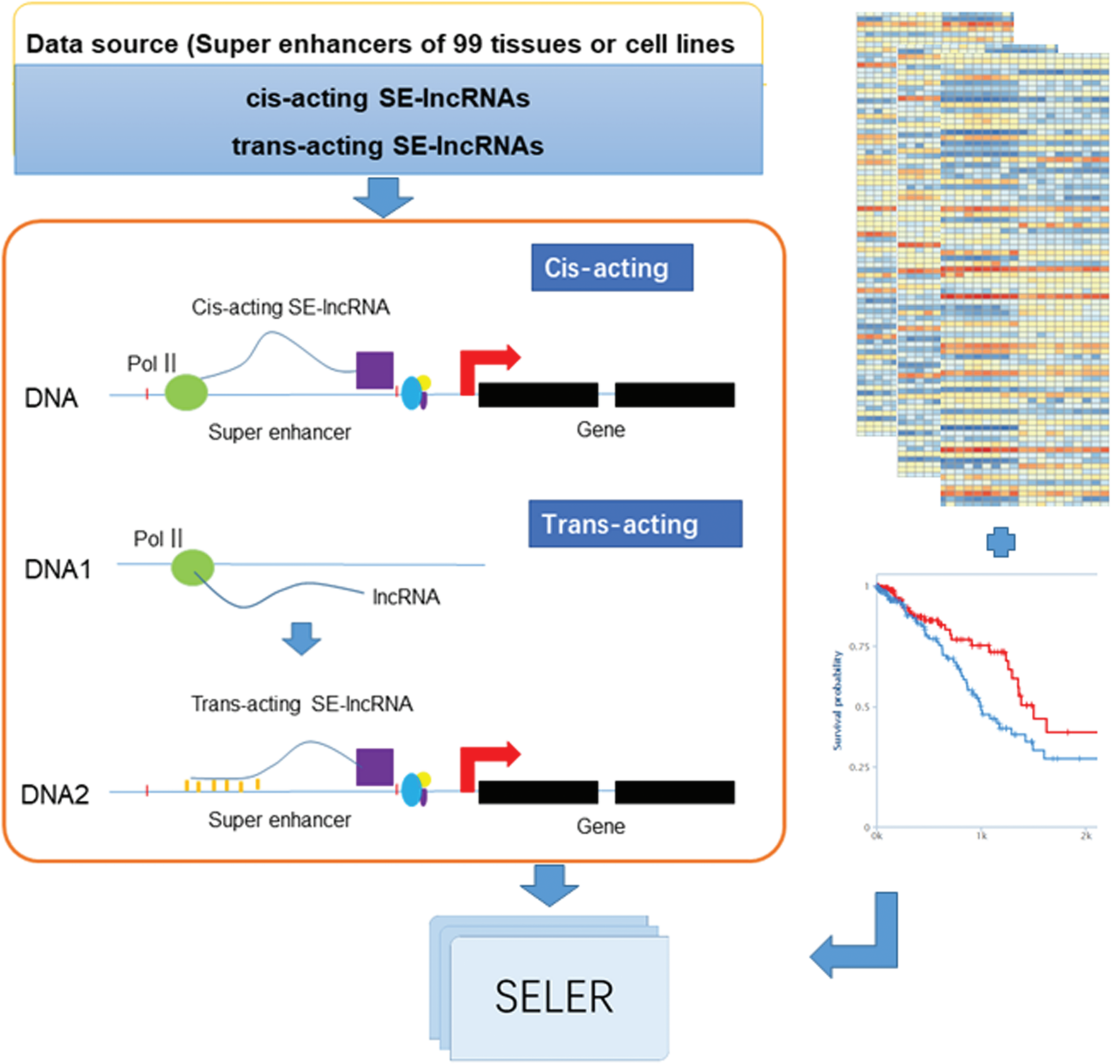
System overview of cancer-related SE-lncRNAs database construction. The workflow of cancer-related SE-lncRNA database construction mainly consisted of the following three sections: SE-lncRNA identification, cancer-related SE-lncRNA annotation and database construction. We first identified trans-acting and cis-acting SE-lncRNAs according to their regulatory mechanisms (left part of Figure 1). To explore cancer-related SE-lncRNAs, we identified significantly differentially expressed SE-lncRNAs and SE-lncRNAs with high prognostic values (right part of Figure 1). Moreover, we calculated the correlation coefficient along with the regulated genes of each cancer type to identify their truly regulatory relationships. Finally, the SELER database was built.

The analytical workflow of the construction of the cancer-related SE-lncRNA database mainly consisted of the following three sections: SE-lncRNA identification, cancer-related SE-lncRNA annotation and database construction (Figure 1).

### SE-lncRNA identification

The SEs of 102 different cell lines and the lncRNA information were downloaded from dbSUPER (downloaded on 1 October 2018) (2) and GENCODE (v27) (17), respectively. The human reference genome (hg19) was applied to handle genomic coordinates. By comparing genomic coordinates, the whole length or the transcription start site (TSS) of an lncRNA within an SE was taken as a cis-acting SE-lncRNA. A previous study has identified hundreds of trans-acting SE-lncRNAs, which were downloaded from its Supplementary Materials (13). As trans-acting SE-lncRNAs mainly exerted their functions in the nucleus, we applied lncLocator (18) to predict nuclear-retained lncRNAs. Then, the interactions between SEs and trans-acting SE-lncRNAs were predicted using Triplexator with options: -l 19 -e 5 -c 1 (19). As the previous study revealed, most of regulated genes of SEs were within 50 kilobase (kb) (20), and the TSSs of the protein-coding genes covered by SEs or within a segment 50 kb upstream or downstream of the SEs were taken as their regulated genes. Functional enrichment analysis was performed using PANTHER with the default setting to analyse the regulated genes of two types of SE-lncRNAs (Supplementary Material, Table 1) (21).

### Cancer-related SE-lncRNA annotation

The lncRNA expression profiles, Pearson’s correlation coefficient between lncRNA and protein coding genes and prognostic values across 19 cancer types were downloaded from TANRIC (v1.0) (22). By comparing the SE-lncRNAs expression levels in cancer tissues with their corresponding adjacent normal tissues, differentially expressed SE-lncRNAs were identified by using Student’s *t*-test. The *P*-value was adjusted to the false discovery rate (FDR) by using the Benjamini–Hochberg method. FDR ≤ 0.1 and |log_2_fold change| ≥ 1 were taken as the criteria to identify significantly differentially expressed lncRNAs. We identified SE-lncRNAs with highly significant prognostic value (*P*-value ≤ 0.05) from the survival analysis results of TANRIC. To annotate the functions of SE-lncRNAs in cancers or other diseases, the databases of LncRNADisease (11) and Lnc2Cancer (10) were used (downloaded on 1 May 2018).

### Database construction

To store and display data, a LAMP (Linux, Apache, MySQL and PHP)-based database and web server were developed, which were provided for users to explore the potential regulatory functions of cancer-related SE-lncRNAs.

## Results

### Putative regulatory genes of SE-lncRNAs

Previous studies have reported that two types of SE-lncRNAs exist, including cis-acting and trans-acting SE-lncRNAs (2, 12). The cis-acting SE-lncRNAs were transcribed from SEs to regulate the gene nearby, and the trans-acting SE-lncRNAs were transcribed from other genomic coordinates that interact with SEs to regulate genes from a distance. In total, we identified 5056 SE-lncRNAs, including 4996 cis-acting and 123 trans-acting SE-lncRNAs (Table 1). The cis-acting SE-lncRNAs were transcribed from 24 697 SEs and the trans-acting SE-lncRNAs interacted with 4629 SEs to regulate closet gene expression. By comparing the genome coordinates of SEs with the closet protein-coding genes, 8171 and 4557 regulated genes were inferred to be regulated by cis- and trans-acting SE-lncRNAs, respectively (Table 1). Then, we investigated the predominantly regulatory functions of the regulated genes of two types of SE-lncRNAs by gene functional enrichment analysis. The enriched results showed that the regulated genes of cis-acting and trans-acting SE-lncRNAs were enriched in the immune-related processes, indicating their potential roles in regulating human immune system (Supplementary Material, Table 2 and Supplementary Material, Table 3).

**Table 1:** Data statistics of SE-lncRNAs and their regulated genes

Mechanism	Super enhancer	lncRNA gene	lncRNA transcript	Gene	Gene transcript	Regulatory relationship
Cis-acting	24 697	4996	8821	8171	57 843	16 272
Trans-acting	4629	123	179	4577	32 676	11 214
Total	27 029	5056	8908	9491	67 899	27 481

Gene = regulated genes of SE-lncRNAs. Gene transcript = regulated gene transcripts of SE-lncRNAs. Regulatory relationship = regulatory relations between SE-lncRNA and their regulated genes.

**Table 2:** Data statistics of cancer-related SE-lncRNAs

Type	DF	PV	DF&PV	Significant relationship
Cis-acting	430	2032	347	3622
Trans-acting	13	19	7	401
Total	436	2035	349	3935

DF = significantly differentially expressed SE-lncRNAs. PV = SE-lncRNAs with a high prognostic value. DF&PV = significantly differentially expressed SE-lncRNAs with a high prognostic value. Significant relationship = significant regulatory relationship with *P*-value≤0.05 in Cox’s proportional hazard analysis.

### Substantial cancer-related SE-lncRNAs identified in multiple cancers

**Figure 2:**
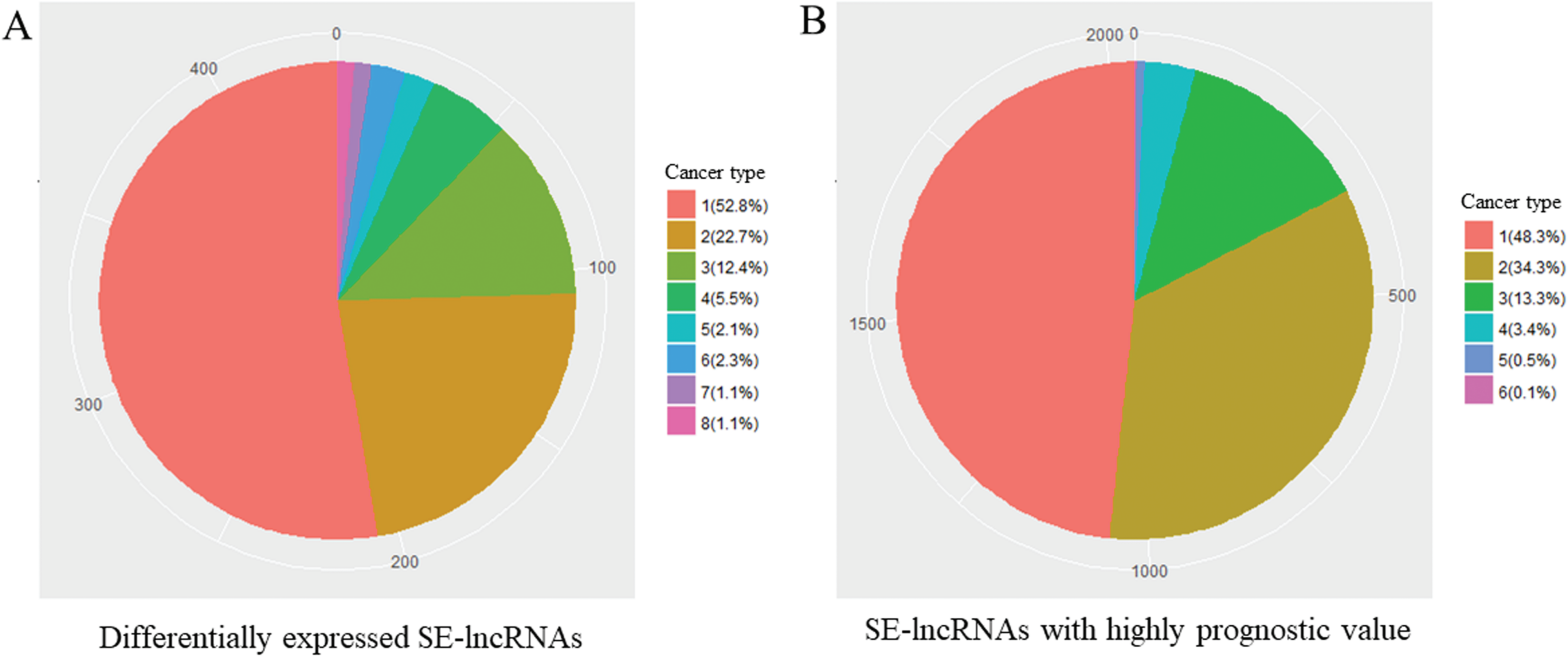
Number of different cancer type with identical SE-lncRNAs. (**A**) Significantly differentially expressed SE-lncRNAs. (**B**) SE-lncRNAs with high prognostic values. Number means the number of cancer types with identical SE-lncRNAs.

To recognize cancer-related SE-lncRNAs, we first identified significantly differentially expressed SE-lncRNAs and SE-lncRNAs with a highly significant prognostic value in each cancer type. In total, we found 436 differentially expressed SE-lncRNAs and ~53% of these molecules were dysregulated in one cancer type, which may reflect their tissue-specific regulation in tumorigenesis (Table 2 and Figure 2A). In addition to these dysregulated SE-lncRNAs, we identified 2035 SE-lncRNAs with a highly significant prognostic value by using Cox’s proportional hazard model (Table 2). Similar to the dysregulated genes, most of the SE-lncRNAs with superior prognostic value showed the cancer type-specific pattern (Table 2 and Figure 2B).

**Figure 3:**
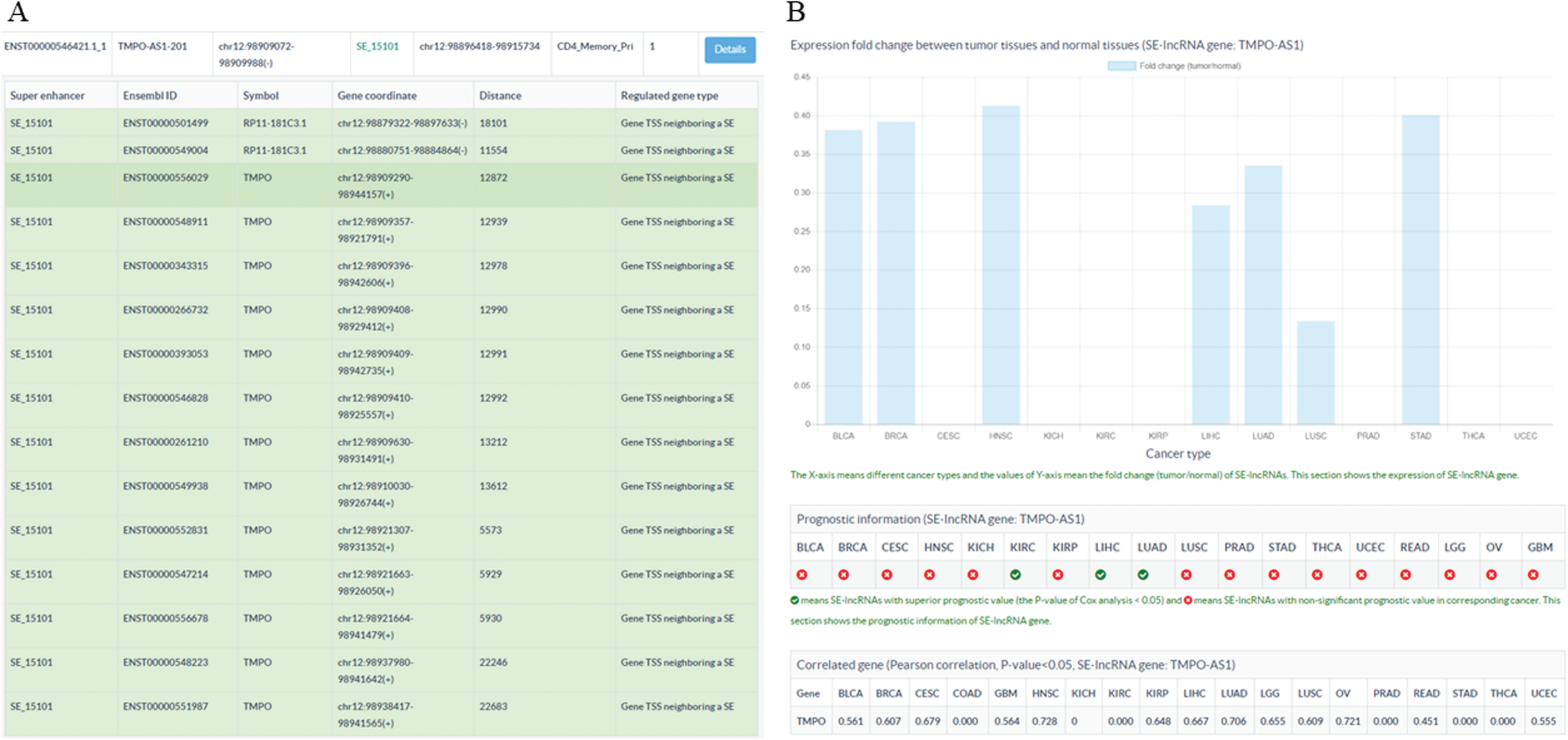
Sample output diagram for the result of the SE-lncRNA section. (**A**) Information about super enhancers, SE-lncRNAs and their associated genes. (**B**) Cancer-related information about SE-lncRNAs, including their expression profiles, prognostic information and significantly associated genes in cancers.

To reveal the potential functions of SE-lncRNAs in cancers, it is important to identify the genes that are truly regulated by the SE-lncRNAs in each cancer type. As the close correlation between the expression of SE-lncRNAs and their regulated genes in cancers may reflect their regulatory relationships, we next evaluated their relationships by calculating their Pearson’s correlation coefficients for each cancer type. We found that 3622 cis-acting regulatory relationships and 401 trans-acting regulatory relationships were significantly correlated, which may reveal the truly regulated genes of SE-lncRNAs in cancers (Table 2). For some lncRNAs for which the function is known, we found that the pathological functions of 91 SE-lncRNAs were annotated by the LncRNADisease and Lnc2Cancer databases.

### Database introduction

To store and display the related data of SE-lncRNAs, a database SELER was built. The search results of SELER consisted of the following three sections: the putative SE-lncRNAs section, cancer-related SE-lncRNAs section and function known SE-lncRNA section. The putative SE-lncRNAs section offered basic information on SEs, SE-lncRNAs and their putative regulated genes (Figure 3A). The cancer-related SE-lncRNAs section provided their expression profiles, prognostic values and significantly correlated genes in different cancers (Figure 3B). The function-known SE-lncRNA section provided the experimentally validated functions of SE-lncRNAs in diseases. Apart from being shown in the database, all of the abovementioned data could be downloaded from the download interface of SELER.

To identify cancer-related SE-lncRNAs and to explore their potential functions, users could apply filtering options to select significantly differentially expressed SE-lncRNAs with a high prognostic value. Moreover, the correlation coefficient between SE-lncRNAs and their regulated genes could help users to identify their truly regulated genes. For instance, we first chose the filtering options of DF&PV option to retain significantly differentially expressed cis-acting SE-lncRNAs with highly prognostic value. Then, we input TMPO-AS1-201 to search the database. We found that TMPO-AS1-201 was significantly differentially expressed in seven cancer types (Figure 3B). More importantly, TMPO-AS1-201 showed a high prognostic value in two of the seven cancer types, including liver hepatocellular carcinoma (LIHC) and lung squamous cell carcinoma (LUSC) (Figure 3B). To reveal the potential functions of TMPO-AS1-201 in these cancers, we investigated its significantly correlated genes in corresponding cancers and found that it was significantly correlated with thymopoietin (TMPO) in LIHC and LUSC (Figure 3B), which indicated that TMPO-AS1-201 may participate in tumorigenesis by regulating the gene expression of TMPO (Figure 3B).

## Discussion

Multiple experimental and computational data were integrated to identify cancer-related SE-lncRNAs and to explore their potential regulatory functions in tumorigenesis. In total, 2122 cancer-related SE-lncRNAs were identified, including 436 significantly differentially expressed lncRNAs and 2035 with a high prognostic value. Moreover, 3935 significantly correlated relationships between SE-lncRNAs and their regulated genes were identified in 19 cancer types. Finally, the SELER database was built to provide users with a useful tool to investigate SE-lncRNA functions in cancers.

Compared to other lncRNA databases, the distinctive features of SELER are as follows: (i) SELER mainly focused on SE-lncRNAs, which regulated the gene expression by controlling SE activity. Our database systematically identified SE-lncRNAs and their putative regulated genes. (ii) SELER integrated large amounts of expression profile data sets and prognostic information derived from 19 cancer types to identify cancer-associated SE-lncRNAs. (iii) SELER comprehensively annotated SE-lncRNAs for which the functions are known. (iv) SELER provided a user-friendly interface for users to explore the potential roles of SE-lncRNAs in tumorigenesis.

In summary, SELER is a novel database that integrates large amounts of experimental and computational data to decode the regulated functions of SE-lncRNAs. Considerable information is offered to facilitate the investigation of SE-lncRNAs in tumorigenesis. SELER is expected to improve our comprehensive understanding of the important and novel roles of SE-lncRNAs in the regulation of gene expression and in pathological processes.

## Supplementary Material

Supplementary Data

## Notes

**Database URL:** www.seler.cn
