# Supplementary material for: SELER: a database of super-enhancer-associated lncRNA- directed transcriptional regulation in human cancers
Source: Database (Oxford). 2019 Feb 26;2019:baz027. doi: 10.1093/database/baz027 (PMC6390648; doi:10.1093/database/baz027)
Supplement: Supplementary Data [file supplementary_materials_baz027.docx]

**Supplementary Table 1.** The list of regulated genes of cis-acting and trans-acting SE-lncRNAs.

| Number | Cis-acting SE-lncRNAs | Trans-acting SE-lncRNAs |
| --- | --- | --- |
| 1 | ENSG00000154548 | ENSG00000109819 |
| 2 | ENSG00000069011 | ENSG00000182575 |
| 3 | ENSG00000182575 | ENSG00000270249 |
| 4 | ENSG00000160013 | ENSG00000160013 |
| 5 | ENSG00000124151 | ENSG00000124151 |
| 6 | ENSG00000167394 | ENSG00000095637 |
| 7 | ENSG00000188906 | ENSG00000184361 |
| 8 | ENSG00000051596 | ENSG00000006283 |
| 9 | ENSG00000179698 | ENSG00000100796 |
| 10 | ENSG00000127329 | ENSG00000105701 |
| 11 | ENSG00000226104 | ENSG00000247270 |
| 12 | ENSG00000095637 | ENSG00000142871 |
| 13 | ENSG00000182511 | ENSG00000182450 |
| 14 | ENSG00000189241 | ENSG00000006468 |
| 15 | ENSG00000136925 | ENSG00000114654 |
| 16 | ENSG00000184361 | ENSG00000100218 |
| 17 | ENSG00000006283 | ENSG00000115486 |
| 18 | ENSG00000113845 | ENSG00000178233 |
| 19 | ENSG00000100796 | ENSG00000143507 |
| 20 | ENSG00000105701 | ENSG00000122786 |
| 21 | ENSG00000135541 | ENSG00000107954 |
| 22 | ENSG00000143376 | ENSG00000178372 |
| 23 | ENSG00000247270 | ENSG00000177370 |
| 24 | ENSG00000141367 | ENSG00000184497 |
| 25 | ENSG00000196155 | ENSG00000070495 |
| 26 | ENSG00000142871 | ENSG00000103005 |
| 27 | ENSG00000157216 | ENSG00000130748 |
| 28 | ENSG00000062282 | ENSG00000149483 |
| 29 | ENSG00000134452 | ENSG00000005884 |
| 30 | ENSG00000182450 | ENSG00000086102 |
| 31 | ENSG00000185988 | ENSG00000140455 |
| 32 | ENSG00000175130 | ENSG00000091128 |
| 33 | ENSG00000142539 | ENSG00000204165 |
| 34 | ENSG00000213213 | ENSG00000178934 |
| 35 | ENSG00000176845 | ENSG00000034510 |
| 36 | ENSG00000100479 | ENSG00000141956 |
| 37 | ENSG00000124562 | ENSG00000205362 |
| 38 | ENSG00000223609 | ENSG00000169967 |
| 39 | ENSG00000029639 | ENSG00000174684 |
| 40 | ENSG00000120669 | ENSG00000173436 |
| 41 | ENSG00000132604 | ENSG00000109971 |
| 42 | ENSG00000166405 | ENSG00000165566 |
| 43 | ENSG00000111218 | ENSG00000251246 |
| 44 | ENSG00000117477 | ENSG00000183128 |
| 45 | ENSG00000178372 | ENSG00000167536 |
| 46 | ENSG00000107954 | ENSG00000168259 |
| 47 | ENSG00000167244 | ENSG00000099864 |
| 48 | ENSG00000131015 | ENSG00000185088 |
| 49 | ENSG00000213901 | ENSG00000188770 |
| 50 | ENSG00000177370 | ENSG00000183624 |
| 51 | ENSG00000142185 | ENSG00000164077 |
| 52 | ENSG00000184497 | ENSG00000100379 |
| 53 | ENSG00000158710 | ENSG00000260170 |
| 54 | ENSG00000070495 | ENSG00000159259 |
| 55 | ENSG00000101194 | ENSG00000165527 |
| 56 | ENSG00000268434 | ENSG00000126500 |
| 57 | ENSG00000149483 | ENSG00000071282 |
| 58 | ENSG00000130748 | ENSG00000008283 |
| 59 | ENSG00000005884 | ENSG00000108468 |
| 60 | ENSG00000100211 | ENSG00000169495 |
| 61 | ENSG00000140455 | ENSG00000134250 |
| 62 | ENSG00000183617 | ENSG00000054523 |
| 63 | ENSG00000183196 | ENSG00000135148 |
| 64 | ENSG00000215621 | ENSG00000184709 |
| 65 | ENSG00000254943 | ENSG00000102057 |
| 66 | ENSG00000167232 | ENSG00000159579 |
| 67 | ENSG00000091128 | ENSG00000121067 |
| 68 | ENSG00000251380 | ENSG00000155846 |
| 69 | ENSG00000188089 | ENSG00000107338 |
| 70 | ENSG00000204533 | ENSG00000168274 |
| 71 | ENSG00000178934 | ENSG00000132669 |
| 72 | ENSG00000117394 | ENSG00000177098 |
| 73 | ENSG00000123594 | ENSG00000087086 |
| 74 | ENSG00000172519 | ENSG00000053371 |
| 75 | ENSG00000136161 | ENSG00000131238 |
| 76 | ENSG00000141956 | ENSG00000158186 |
| 77 | ENSG00000187608 | ENSG00000077463 |
| 78 | ENSG00000153922 | ENSG00000178804 |
| 79 | ENSG00000205362 | ENSG00000122378 |
| 80 | ENSG00000169967 | ENSG00000165512 |
| 81 | ENSG00000240510 | ENSG00000225362 |
| 82 | ENSG00000174684 | ENSG00000043462 |
| 83 | ENSG00000173436 | ENSG00000123636 |
| 84 | ENSG00000109971 | ENSG00000155792 |
| 85 | ENSG00000171872 | ENSG00000163704 |
| 86 | ENSG00000141298 | ENSG00000178567 |
| 87 | ENSG00000272162 | ENSG00000154556 |
| 88 | ENSG00000165566 | ENSG00000131153 |
| 89 | ENSG00000251246 | ENSG00000127084 |
| 90 | ENSG00000183128 | ENSG00000058453 |
| 91 | ENSG00000268805 | ENSG00000160285 |
| 92 | ENSG00000129566 | ENSG00000049540 |
| 93 | ENSG00000261052 | ENSG00000121671 |
| 94 | ENSG00000167536 | ENSG00000107798 |
| 95 | ENSG00000131652 | ENSG00000136026 |
| 96 | ENSG00000099864 | ENSG00000196712 |
| 97 | ENSG00000085265 | ENSG00000204175 |
| 98 | ENSG00000185088 | ENSG00000168924 |
| 99 | ENSG00000213015 | ENSG00000181852 |
| 100 | ENSG00000183624 | ENSG00000165905 |
| 101 | ENSG00000182851 | ENSG00000186919 |
| 102 | ENSG00000134757 | ENSG00000180592 |
| 103 | ENSG00000100379 | ENSG00000181938 |
| 104 | ENSG00000164077 | ENSG00000168509 |
| 105 | ENSG00000260170 | ENSG00000008710 |
| 106 | ENSG00000159259 | ENSG00000197943 |
| 107 | ENSG00000008283 | ENSG00000120129 |
| 108 | ENSG00000071282 | ENSG00000142784 |
| 109 | ENSG00000165527 | ENSG00000144711 |
| 110 | ENSG00000126500 | ENSG00000078124 |
| 111 | ENSG00000241127 | ENSG00000047315 |
| 112 | ENSG00000142089 | ENSG00000159335 |
| 113 | ENSG00000108468 | ENSG00000096996 |
| 114 | ENSG00000243709 | ENSG00000085872 |
| 115 | ENSG00000169495 | ENSG00000051341 |
| 116 | ENSG00000134250 | ENSG00000013503 |
| 117 | ENSG00000054523 | ENSG00000182319 |
| 118 | ENSG00000185176 | ENSG00000139323 |
| 119 | ENSG00000152582 | ENSG00000111639 |
| 120 | ENSG00000225663 | ENSG00000156675 |
| 121 | ENSG00000102057 | ENSG00000044115 |
| 122 | ENSG00000159579 | ENSG00000175745 |
| 123 | ENSG00000121067 | ENSG00000169006 |
| 124 | ENSG00000266258 | ENSG00000100196 |
| 125 | ENSG00000011260 | ENSG00000110987 |
| 126 | ENSG00000106070 | ENSG00000144791 |
| 127 | ENSG00000241563 | ENSG00000114480 |
| 128 | ENSG00000141968 | ENSG00000125148 |
| 129 | ENSG00000179776 | ENSG00000234511 |
| 130 | ENSG00000155760 | ENSG00000130751 |
| 131 | ENSG00000125266 | ENSG00000125695 |
| 132 | ENSG00000241370 | ENSG00000135898 |
| 133 | ENSG00000168274 | ENSG00000167900 |
| 134 | ENSG00000088888 | ENSG00000161381 |
| 135 | ENSG00000110721 | ENSG00000152601 |
| 136 | ENSG00000132669 | ENSG00000155926 |
| 137 | ENSG00000107404 | ENSG00000132938 |
| 138 | ENSG00000167965 | ENSG00000117595 |
| 139 | ENSG00000105829 | ENSG00000189046 |
| 140 | ENSG00000148334 | ENSG00000180979 |
| 141 | ENSG00000187796 | ENSG00000198892 |
| 142 | ENSG00000087086 | ENSG00000145012 |
| 143 | ENSG00000166278 | ENSG00000178585 |
| 144 | ENSG00000170296 | ENSG00000183765 |
| 145 | ENSG00000160111 | ENSG00000258989 |
| 146 | ENSG00000169026 | ENSG00000162931 |
| 147 | ENSG00000106541 | ENSG00000077312 |
| 148 | ENSG00000106113 | ENSG00000196169 |
| 149 | ENSG00000196220 | ENSG00000145022 |
| 150 | ENSG00000134809 | ENSG00000142347 |
| 151 | ENSG00000249109 | ENSG00000124449 |
| 152 | ENSG00000125755 | ENSG00000179841 |
| 153 | ENSG00000077463 | ENSG00000172216 |
| 154 | ENSG00000184007 | ENSG00000204160 |
| 155 | ENSG00000122378 | ENSG00000115112 |
| 156 | ENSG00000081019 | ENSG00000261857 |
| 157 | ENSG00000165512 | ENSG00000119669 |
| 158 | ENSG00000140564 | ENSG00000107807 |
| 159 | ENSG00000225362 | ENSG00000131408 |
| 160 | ENSG00000135835 | ENSG00000119919 |
| 161 | ENSG00000258792 | ENSG00000167778 |
| 162 | ENSG00000123636 | ENSG00000141696 |
| 163 | ENSG00000043462 | ENSG00000136908 |
| 164 | ENSG00000115963 | ENSG00000171840 |
| 165 | ENSG00000099330 | ENSG00000158296 |
| 166 | ENSG00000160199 | ENSG00000214900 |
| 167 | ENSG00000212901 | ENSG00000186834 |
| 168 | ENSG00000155792 | ENSG00000142920 |
| 169 | ENSG00000154642 | ENSG00000141448 |
| 170 | ENSG00000262660 | ENSG00000269223 |
| 171 | ENSG00000141378 | ENSG00000186687 |
| 172 | ENSG00000154556 | ENSG00000175662 |
| 173 | ENSG00000131153 | ENSG00000108100 |
| 174 | ENSG00000101146 | ENSG00000101336 |
| 175 | ENSG00000106993 | ENSG00000128268 |
| 176 | ENSG00000005073 | ENSG00000174004 |
| 177 | ENSG00000127084 | ENSG00000140534 |
| 178 | ENSG00000164330 | ENSG00000115738 |
| 179 | ENSG00000164597 | ENSG00000205927 |
| 180 | ENSG00000213533 | ENSG00000071626 |
| 181 | ENSG00000058453 | ENSG00000122971 |
| 182 | ENSG00000100056 | ENSG00000211455 |
| 183 | ENSG00000160285 | ENSG00000116459 |
| 184 | ENSG00000049540 | ENSG00000268413 |
| 185 | ENSG00000122335 | ENSG00000162894 |
| 186 | ENSG00000125449 | ENSG00000101076 |
| 187 | ENSG00000005381 | ENSG00000137699 |
| 188 | ENSG00000121671 | ENSG00000166411 |
| 189 | ENSG00000107798 | ENSG00000167264 |
| 190 | ENSG00000131686 | ENSG00000069345 |
| 191 | ENSG00000197279 | ENSG00000148229 |
| 192 | ENSG00000136026 | ENSG00000186235 |
| 193 | ENSG00000262814 | ENSG00000204850 |
| 194 | ENSG00000133665 | ENSG00000103184 |
| 195 | ENSG00000124570 | ENSG00000100565 |
| 196 | ENSG00000134215 | ENSG00000205867 |
| 197 | ENSG00000243649 | ENSG00000185264 |
| 198 | ENSG00000204175 | ENSG00000143545 |
| 199 | ENSG00000188313 | ENSG00000141255 |
| 200 | ENSG00000169291 | ENSG00000119688 |
| 201 | ENSG00000136352 | ENSG00000175906 |
| 202 | ENSG00000269155 | ENSG00000108381 |
| 203 | ENSG00000177045 | ENSG00000126768 |
| 204 | ENSG00000137877 | ENSG00000161955 |
| 205 | ENSG00000167553 | ENSG00000103653 |
| 206 | ENSG00000181852 | ENSG00000065054 |
| 207 | ENSG00000165905 | ENSG00000164733 |
| 208 | ENSG00000262526 | ENSG00000212864 |
| 209 | ENSG00000135269 | ENSG00000105642 |
| 210 | ENSG00000181938 | ENSG00000070614 |
| 211 | ENSG00000096433 | ENSG00000135677 |
| 212 | ENSG00000156261 | ENSG00000119616 |
| 213 | ENSG00000163283 | ENSG00000163781 |
| 214 | ENSG00000176381 | ENSG00000169951 |
| 215 | ENSG00000157388 | ENSG00000088256 |
| 216 | ENSG00000168509 | ENSG00000198887 |
| 217 | ENSG00000111666 | ENSG00000162599 |
| 218 | ENSG00000008710 | ENSG00000119411 |
| 219 | ENSG00000186577 | ENSG00000205730 |
| 220 | ENSG00000197943 | ENSG00000173175 |
| 221 | ENSG00000214367 | ENSG00000184678 |
| 222 | ENSG00000156050 | ENSG00000167513 |
| 223 | ENSG00000125505 | ENSG00000168148 |
| 224 | ENSG00000213341 | ENSG00000069493 |
| 225 | ENSG00000120129 | ENSG00000123146 |
| 226 | ENSG00000119285 | ENSG00000066336 |
| 227 | ENSG00000144711 | ENSG00000168453 |
| 228 | ENSG00000110619 | ENSG00000136982 |
| 229 | ENSG00000047315 | ENSG00000162873 |
| 230 | ENSG00000078124 | ENSG00000087250 |
| 231 | ENSG00000096996 | ENSG00000184792 |
| 232 | ENSG00000085872 | ENSG00000203804 |
| 233 | ENSG00000255561 | ENSG00000078053 |
| 234 | ENSG00000142235 | ENSG00000126251 |
| 235 | ENSG00000100147 | ENSG00000170873 |
| 236 | ENSG00000143217 | ENSG00000064042 |
| 237 | ENSG00000168676 | ENSG00000196975 |
| 238 | ENSG00000267824 | ENSG00000065613 |
| 239 | ENSG00000139323 | ENSG00000035403 |
| 240 | ENSG00000111639 | ENSG00000167693 |
| 241 | ENSG00000156675 | ENSG00000198113 |
| 242 | ENSG00000196176 | ENSG00000170442 |
| 243 | ENSG00000044115 | ENSG00000162066 |
| 244 | ENSG00000146109 | ENSG00000143199 |
| 245 | ENSG00000118245 | ENSG00000084764 |
| 246 | ENSG00000258729 | ENSG00000197756 |
| 247 | ENSG00000256660 | ENSG00000197405 |
| 248 | ENSG00000155287 | ENSG00000269279 |
| 249 | ENSG00000135966 | ENSG00000130635 |
| 250 | ENSG00000130700 | ENSG00000179889 |
| 251 | ENSG00000175745 | ENSG00000136295 |
| 252 | ENSG00000169006 | ENSG00000136279 |
| 253 | ENSG00000100196 | ENSG00000178982 |
| 254 | ENSG00000180096 | ENSG00000148358 |
| 255 | ENSG00000196966 | ENSG00000197182 |
| 256 | ENSG00000251201 | ENSG00000100987 |
| 257 | ENSG00000114480 | ENSG00000255587 |
| 258 | ENSG00000144791 | ENSG00000110514 |
| 259 | ENSG00000089041 | ENSG00000149634 |
| 260 | ENSG00000125148 | ENSG00000080823 |
| 261 | ENSG00000110987 | ENSG00000105402 |
| 262 | ENSG00000155189 | ENSG00000117091 |
| 263 | ENSG00000234511 | ENSG00000110841 |
| 264 | ENSG00000130751 | ENSG00000133065 |
| 265 | ENSG00000198211 | ENSG00000091490 |
| 266 | ENSG00000184060 | ENSG00000162148 |
| 267 | ENSG00000125695 | ENSG00000177732 |
| 268 | ENSG00000186193 | ENSG00000239672 |
| 269 | ENSG00000135898 | ENSG00000122966 |
| 270 | ENSG00000130724 | ENSG00000160408 |
| 271 | ENSG00000167900 | ENSG00000159110 |
| 272 | ENSG00000161381 | ENSG00000160683 |
| 273 | ENSG00000119725 | ENSG00000168004 |
| 274 | ENSG00000152601 | ENSG00000113263 |
| 275 | ENSG00000130270 | ENSG00000188986 |
| 276 | ENSG00000269305 | ENSG00000268927 |
| 277 | ENSG00000259399 | ENSG00000254550 |
| 278 | ENSG00000185269 | ENSG00000197181 |
| 279 | ENSG00000189046 | ENSG00000105707 |
| 280 | ENSG00000214226 | ENSG00000145819 |
| 281 | ENSG00000259642 | ENSG00000124466 |
| 282 | ENSG00000164402 | ENSG00000173546 |
| 283 | ENSG00000135740 | ENSG00000020181 |
| 284 | ENSG00000198892 | ENSG00000105643 |
| 285 | ENSG00000170214 | ENSG00000040933 |
| 286 | ENSG00000147697 | ENSG00000203852 |
| 287 | ENSG00000145868 | ENSG00000087338 |
| 288 | ENSG00000198901 | ENSG00000105695 |
| 289 | ENSG00000236320 | ENSG00000170242 |
| 290 | ENSG00000219200 | ENSG00000121851 |
| 291 | ENSG00000108671 | ENSG00000146192 |
| 292 | ENSG00000145012 | ENSG00000084754 |
| 293 | ENSG00000105374 | ENSG00000021355 |
| 294 | ENSG00000183765 | ENSG00000172765 |
| 295 | ENSG00000178585 | ENSG00000144712 |
| 296 | ENSG00000258989 | ENSG00000213203 |
| 297 | ENSG00000016082 | ENSG00000132026 |
| 298 | ENSG00000118513 | ENSG00000115266 |
| 299 | ENSG00000007312 | ENSG00000164896 |
| 300 | ENSG00000077312 | ENSG00000140382 |
| 301 | ENSG00000145022 | ENSG00000113558 |
| 302 | ENSG00000196169 | ENSG00000168411 |
| 303 | ENSG00000185070 | ENSG00000139613 |
| 304 | ENSG00000105135 | ENSG00000105948 |
| 305 | ENSG00000163466 | ENSG00000145191 |
| 306 | ENSG00000124449 | ENSG00000204219 |
| 307 | ENSG00000132275 | ENSG00000184260 |
| 308 | ENSG00000204160 | ENSG00000100425 |
| 309 | ENSG00000172216 | ENSG00000133612 |
| 310 | ENSG00000115112 | ENSG00000197191 |
| 311 | ENSG00000261857 | ENSG00000143409 |
| 312 | ENSG00000104894 | ENSG00000163762 |
| 313 | ENSG00000100711 | ENSG00000100997 |
| 314 | ENSG00000170876 | ENSG00000078814 |
| 315 | ENSG00000119669 | ENSG00000169688 |
| 316 | ENSG00000107807 | ENSG00000156831 |
| 317 | ENSG00000131408 | ENSG00000188672 |
| 318 | ENSG00000119919 | ENSG00000111652 |
| 319 | ENSG00000123989 | ENSG00000144674 |
| 320 | ENSG00000214188 | ENSG00000142949 |
| 321 | ENSG00000167778 | ENSG00000051825 |
| 322 | ENSG00000186063 | ENSG00000166710 |
| 323 | ENSG00000136908 | ENSG00000159592 |
| 324 | ENSG00000263620 | ENSG00000081923 |
| 325 | ENSG00000171840 | ENSG00000239264 |
| 326 | ENSG00000181291 | ENSG00000170921 |
| 327 | ENSG00000214900 | ENSG00000143379 |
| 328 | ENSG00000143466 | ENSG00000164877 |
| 329 | ENSG00000235098 | ENSG00000267909 |
| 330 | ENSG00000256053 | ENSG00000164985 |
| 331 | ENSG00000158458 | ENSG00000103540 |
| 332 | ENSG00000186834 | ENSG00000134308 |
| 333 | ENSG00000074356 | ENSG00000166797 |
| 334 | ENSG00000269223 | ENSG00000170439 |
| 335 | ENSG00000141448 | ENSG00000196132 |
| 336 | ENSG00000142920 | ENSG00000109787 |
| 337 | ENSG00000007384 | ENSG00000197345 |
| 338 | ENSG00000085552 | ENSG00000106804 |
| 339 | ENSG00000186687 | ENSG00000229615 |
| 340 | ENSG00000108100 | ENSG00000197429 |
| 341 | ENSG00000175662 | ENSG00000137857 |
| 342 | ENSG00000242515 | ENSG00000065717 |
| 343 | ENSG00000204393 | ENSG00000074657 |
| 344 | ENSG00000114378 | ENSG00000081059 |
| 345 | ENSG00000101336 | ENSG00000049323 |
| 346 | ENSG00000128268 | ENSG00000058668 |
| 347 | ENSG00000174004 | ENSG00000134571 |
| 348 | ENSG00000140534 | ENSG00000178035 |
| 349 | ENSG00000188807 | ENSG00000123143 |
| 350 | ENSG00000108753 | ENSG00000184232 |
| 351 | ENSG00000169627 | ENSG00000066056 |
| 352 | ENSG00000115738 | ENSG00000104907 |
| 353 | ENSG00000073146 | ENSG00000156113 |
| 354 | ENSG00000144228 | ENSG00000177483 |
| 355 | ENSG00000205927 | ENSG00000104213 |
| 356 | ENSG00000071626 | ENSG00000100445 |
| 357 | ENSG00000122971 | ENSG00000166689 |
| 358 | ENSG00000268413 | ENSG00000244242 |
| 359 | ENSG00000126233 | ENSG00000152292 |
| 360 | ENSG00000101076 | ENSG00000120051 |
| 361 | ENSG00000137699 | ENSG00000174238 |
| 362 | ENSG00000139656 | ENSG00000111305 |
| 363 | ENSG00000139190 | ENSG00000019995 |
| 364 | ENSG00000166411 | ENSG00000154217 |
| 365 | ENSG00000078808 | ENSG00000131187 |
| 366 | ENSG00000103510 | ENSG00000165655 |
| 367 | ENSG00000167264 | ENSG00000214921 |
| 368 | ENSG00000167619 | ENSG00000136932 |
| 369 | ENSG00000105835 | ENSG00000125844 |
| 370 | ENSG00000148229 | ENSG00000204278 |
| 371 | ENSG00000129152 | ENSG00000172059 |
| 372 | ENSG00000186235 | ENSG00000120992 |
| 373 | ENSG00000115419 | ENSG00000116525 |
| 374 | ENSG00000205867 | ENSG00000163521 |
| 375 | ENSG00000103184 | ENSG00000037241 |
| 376 | ENSG00000100565 | ENSG00000168389 |
| 377 | ENSG00000041880 | ENSG00000093167 |
| 378 | ENSG00000110719 | ENSG00000258740 |
| 379 | ENSG00000197213 | ENSG00000117713 |
| 380 | ENSG00000143545 | ENSG00000168298 |
| 381 | ENSG00000221978 | ENSG00000116151 |
| 382 | ENSG00000007168 | ENSG00000181781 |
| 383 | ENSG00000170191 | ENSG00000136827 |
| 384 | ENSG00000101084 | ENSG00000131370 |
| 385 | ENSG00000245017 | ENSG00000147789 |
| 386 | ENSG00000166143 | ENSG00000137522 |
| 387 | ENSG00000175906 | ENSG00000185972 |
| 388 | ENSG00000183134 | ENSG00000068383 |
| 389 | ENSG00000105364 | ENSG00000105261 |
| 390 | ENSG00000099365 | ENSG00000172508 |
| 391 | ENSG00000204291 | ENSG00000186329 |
| 392 | ENSG00000214872 | ENSG00000160055 |
| 393 | ENSG00000183251 | ENSG00000175866 |
| 394 | ENSG00000170989 | ENSG00000120162 |
| 395 | ENSG00000132199 | ENSG00000214645 |
| 396 | ENSG00000126768 | ENSG00000163513 |
| 397 | ENSG00000132470 | ENSG00000168734 |
| 398 | ENSG00000154710 | ENSG00000254996 |
| 399 | ENSG00000060491 | ENSG00000050438 |
| 400 | ENSG00000176715 | ENSG00000110917 |
| 401 | ENSG00000161955 | ENSG00000146376 |
| 402 | ENSG00000162739 | ENSG00000123136 |
| 403 | ENSG00000103653 | ENSG00000108557 |
| 404 | ENSG00000065054 | ENSG00000198597 |
| 405 | ENSG00000164733 | ENSG00000137266 |
| 406 | ENSG00000105642 | ENSG00000150753 |
| 407 | ENSG00000070614 | ENSG00000168884 |
| 408 | ENSG00000105514 | ENSG00000125944 |
| 409 | ENSG00000135677 | ENSG00000105254 |
| 410 | ENSG00000066813 | ENSG00000172531 |
| 411 | ENSG00000204308 | ENSG00000071054 |
| 412 | ENSG00000119616 | ENSG00000117266 |
| 413 | ENSG00000108439 | ENSG00000139714 |
| 414 | ENSG00000164609 | ENSG00000242247 |
| 415 | ENSG00000169951 | ENSG00000255103 |
| 416 | ENSG00000167094 | ENSG00000092931 |
| 417 | ENSG00000137273 | ENSG00000204704 |
| 418 | ENSG00000088256 | ENSG00000153310 |
| 419 | ENSG00000198887 | ENSG00000121653 |
| 420 | ENSG00000222005 | ENSG00000175707 |
| 421 | ENSG00000162599 | ENSG00000205148 |
| 422 | ENSG00000205730 | ENSG00000101265 |
| 423 | ENSG00000184678 | ENSG00000272442 |
| 424 | ENSG00000167513 | ENSG00000198837 |
| 425 | ENSG00000088320 | ENSG00000104728 |
| 426 | ENSG00000124406 | ENSG00000187147 |
| 427 | ENSG00000165959 | ENSG00000117013 |
| 428 | ENSG00000166974 | ENSG00000169302 |
| 429 | ENSG00000069493 | ENSG00000269753 |
| 430 | ENSG00000140650 | ENSG00000198756 |
| 431 | ENSG00000123146 | ENSG00000122025 |
| 432 | ENSG00000130856 | ENSG00000137507 |
| 433 | ENSG00000130299 | ENSG00000144824 |
| 434 | ENSG00000184787 | ENSG00000155629 |
| 435 | ENSG00000144648 | ENSG00000197312 |
| 436 | ENSG00000132475 | ENSG00000168077 |
| 437 | ENSG00000066336 | ENSG00000168746 |
| 438 | ENSG00000180694 | ENSG00000106665 |
| 439 | ENSG00000177301 | ENSG00000162441 |
| 440 | ENSG00000175305 | ENSG00000130309 |
| 441 | ENSG00000173826 | ENSG00000006837 |
| 442 | ENSG00000205850 | ENSG00000068305 |
| 443 | ENSG00000107566 | ENSG00000083099 |
| 444 | ENSG00000136982 | ENSG00000181031 |
| 445 | ENSG00000145919 | ENSG00000196923 |
| 446 | ENSG00000162873 | ENSG00000268628 |
| 447 | ENSG00000087250 | ENSG00000111319 |
| 448 | ENSG00000203804 | ENSG00000105223 |
| 449 | ENSG00000139767 | ENSG00000125864 |
| 450 | ENSG00000078053 | ENSG00000164124 |
| 451 | ENSG00000167994 | ENSG00000126353 |
| 452 | ENSG00000126251 | ENSG00000147905 |
| 453 | ENSG00000151746 | ENSG00000102125 |
| 454 | ENSG00000065613 | ENSG00000166444 |
| 455 | ENSG00000175065 | ENSG00000069122 |
| 456 | ENSG00000164440 | ENSG00000125319 |
| 457 | ENSG00000035403 | ENSG00000170515 |
| 458 | ENSG00000167656 | ENSG00000105656 |
| 459 | ENSG00000167693 | ENSG00000076555 |
| 460 | ENSG00000198113 | ENSG00000161526 |
| 461 | ENSG00000146426 | ENSG00000204590 |
| 462 | ENSG00000063438 | ENSG00000173699 |
| 463 | ENSG00000055332 | ENSG00000074276 |
| 464 | ENSG00000173221 | ENSG00000180891 |
| 465 | ENSG00000182896 | ENSG00000127743 |
| 466 | ENSG00000213585 | ENSG00000183032 |
| 467 | ENSG00000170442 | ENSG00000101255 |
| 468 | ENSG00000162066 | ENSG00000110328 |
| 469 | ENSG00000084764 | ENSG00000111011 |
| 470 | ENSG00000115282 | ENSG00000167468 |
| 471 | ENSG00000270149 | ENSG00000071242 |
| 472 | ENSG00000144045 | ENSG00000112715 |
| 473 | ENSG00000185527 | ENSG00000198624 |
| 474 | ENSG00000143458 | ENSG00000175634 |
| 475 | ENSG00000171680 | ENSG00000153395 |
| 476 | ENSG00000204913 | ENSG00000174059 |
| 477 | ENSG00000253304 | ENSG00000108797 |
| 478 | ENSG00000146223 | ENSG00000184602 |
| 479 | ENSG00000269279 | ENSG00000110955 |
| 480 | ENSG00000061987 | ENSG00000130383 |
| 481 | ENSG00000105605 | ENSG00000151360 |
| 482 | ENSG00000234237 | ENSG00000196408 |
| 483 | ENSG00000130635 | ENSG00000125531 |
| 484 | ENSG00000179889 | ENSG00000105205 |
| 485 | ENSG00000128713 | ENSG00000158985 |
| 486 | ENSG00000136279 | ENSG00000114491 |
| 487 | ENSG00000010404 | ENSG00000108823 |
| 488 | ENSG00000178982 | ENSG00000170456 |
| 489 | ENSG00000145740 | ENSG00000141542 |
| 490 | ENSG00000148358 | ENSG00000125746 |
| 491 | ENSG00000117505 | ENSG00000103710 |
| 492 | ENSG00000100987 | ENSG00000103495 |
| 493 | ENSG00000197182 | ENSG00000164087 |
| 494 | ENSG00000151718 | ENSG00000108848 |
| 495 | ENSG00000204001 | ENSG00000143294 |
| 496 | ENSG00000255587 | ENSG00000112339 |
| 497 | ENSG00000172785 | ENSG00000085511 |
| 498 | ENSG00000110514 | ENSG00000185477 |
| 499 | ENSG00000233538 | ENSG00000251184 |
| 500 | ENSG00000204188 | ENSG00000128159 |
| 501 | ENSG00000117091 | ENSG00000143862 |
| 502 | ENSG00000105402 | ENSG00000143315 |
| 503 | ENSG00000262628 | ENSG00000133980 |
| 504 | ENSG00000110841 | ENSG00000187514 |
| 505 | ENSG00000137449 | ENSG00000104823 |
| 506 | ENSG00000141505 | ENSG00000153066 |
| 507 | ENSG00000094914 | ENSG00000198860 |
| 508 | ENSG00000091490 | ENSG00000125744 |
| 509 | ENSG00000197471 | ENSG00000171102 |
| 510 | ENSG00000135476 | ENSG00000164627 |
| 511 | ENSG00000141569 | ENSG00000196979 |
| 512 | ENSG00000197912 | ENSG00000151948 |
| 513 | ENSG00000162148 | ENSG00000226976 |
| 514 | ENSG00000229415 | ENSG00000177383 |
| 515 | ENSG00000177732 | ENSG00000158717 |
| 516 | ENSG00000166589 | ENSG00000076356 |
| 517 | ENSG00000122966 | ENSG00000102030 |
| 518 | ENSG00000160408 | ENSG00000162482 |
| 519 | ENSG00000177728 | ENSG00000204120 |
| 520 | ENSG00000173890 | ENSG00000069188 |
| 521 | ENSG00000159110 | ENSG00000119888 |
| 522 | ENSG00000160683 | ENSG00000180089 |
| 523 | ENSG00000091656 | ENSG00000174791 |
| 524 | ENSG00000171804 | ENSG00000168936 |
| 525 | ENSG00000182393 | ENSG00000178057 |
| 526 | ENSG00000152558 | ENSG00000110536 |
| 527 | ENSG00000108406 | ENSG00000160888 |
| 528 | ENSG00000113263 | ENSG00000070729 |
| 529 | ENSG00000188986 | ENSG00000018408 |
| 530 | ENSG00000268927 | ENSG00000168765 |
| 531 | ENSG00000063176 | ENSG00000143570 |
| 532 | ENSG00000149923 | ENSG00000204176 |
| 533 | ENSG00000105707 | ENSG00000269570 |
| 534 | ENSG00000145819 | ENSG00000143179 |
| 535 | ENSG00000020181 | ENSG00000007541 |
| 536 | ENSG00000173546 | ENSG00000067066 |
| 537 | ENSG00000251139 | ENSG00000010610 |
| 538 | ENSG00000105643 | ENSG00000174177 |
| 539 | ENSG00000083845 | ENSG00000171862 |
| 540 | ENSG00000116754 | ENSG00000133328 |
| 541 | ENSG00000170340 | ENSG00000127804 |
| 542 | ENSG00000203852 | ENSG00000125998 |
| 543 | ENSG00000170178 | ENSG00000269858 |
| 544 | ENSG00000177119 | ENSG00000268626 |
| 545 | ENSG00000162526 | ENSG00000262003 |
| 546 | ENSG00000148426 | ENSG00000121578 |
| 547 | ENSG00000170242 | ENSG00000205356 |
| 548 | ENSG00000105695 | ENSG00000167992 |
| 549 | ENSG00000258664 | ENSG00000180806 |
| 550 | ENSG00000121851 | ENSG00000011600 |
| 551 | ENSG00000021355 | ENSG00000151704 |
| 552 | ENSG00000132382 | ENSG00000108523 |
| 553 | ENSG00000172765 | ENSG00000005243 |
| 554 | ENSG00000178966 | ENSG00000174100 |
| 555 | ENSG00000138035 | ENSG00000116337 |
| 556 | ENSG00000105967 | ENSG00000135441 |
| 557 | ENSG00000070961 | ENSG00000122696 |
| 558 | ENSG00000172568 | ENSG00000049541 |
| 559 | ENSG00000132026 | ENSG00000109906 |
| 560 | ENSG00000189057 | ENSG00000116809 |
| 561 | ENSG00000138668 | ENSG00000111321 |
| 562 | ENSG00000139668 | ENSG00000197043 |
| 563 | ENSG00000115266 | ENSG00000162551 |
| 564 | ENSG00000157240 | ENSG00000002586 |
| 565 | ENSG00000164896 | ENSG00000162391 |
| 566 | ENSG00000069667 | ENSG00000106089 |
| 567 | ENSG00000119537 | ENSG00000268538 |
| 568 | ENSG00000158022 | ENSG00000180008 |
| 569 | ENSG00000113558 | ENSG00000166896 |
| 570 | ENSG00000140382 | ENSG00000196743 |
| 571 | ENSG00000178222 | ENSG00000175604 |
| 572 | ENSG00000181396 | ENSG00000220008 |
| 573 | ENSG00000139613 | ENSG00000132915 |
| 574 | ENSG00000151846 | ENSG00000119866 |
| 575 | ENSG00000170374 | ENSG00000213889 |
| 576 | ENSG00000146005 | ENSG00000104863 |
| 577 | ENSG00000135824 | ENSG00000198925 |
| 578 | ENSG00000167182 | ENSG00000173065 |
| 579 | ENSG00000184260 | ENSG00000139572 |
| 580 | ENSG00000204219 | ENSG00000184221 |
| 581 | ENSG00000100425 | ENSG00000198336 |
| 582 | ENSG00000184566 | ENSG00000101464 |
| 583 | ENSG00000073150 | ENSG00000167578 |
| 584 | ENSG00000133612 | ENSG00000268069 |
| 585 | ENSG00000163762 | ENSG00000137496 |
| 586 | ENSG00000143409 | ENSG00000178828 |
| 587 | ENSG00000100997 | ENSG00000171056 |
| 588 | ENSG00000169688 | ENSG00000134817 |
| 589 | ENSG00000121797 | ENSG00000140548 |
| 590 | ENSG00000162543 | ENSG00000228278 |
| 591 | ENSG00000156831 | ENSG00000123411 |
| 592 | ENSG00000095794 | ENSG00000131899 |
| 593 | ENSG00000092421 | ENSG00000095981 |
| 594 | ENSG00000187266 | ENSG00000171055 |
| 595 | ENSG00000113580 | ENSG00000013725 |
| 596 | ENSG00000169122 | ENSG00000127528 |
| 597 | ENSG00000100505 | ENSG00000099797 |
| 598 | ENSG00000108294 | ENSG00000204682 |
| 599 | ENSG00000158864 | ENSG00000187288 |
| 600 | ENSG00000144674 | ENSG00000149633 |
| 601 | ENSG00000269871 | ENSG00000011304 |
| 602 | ENSG00000246223 | ENSG00000100060 |
| 603 | ENSG00000051825 | ENSG00000162458 |
| 604 | ENSG00000081923 | ENSG00000187720 |
| 605 | ENSG00000082438 | ENSG00000127578 |
| 606 | ENSG00000065883 | ENSG00000105647 |
| 607 | ENSG00000174151 | ENSG00000038532 |
| 608 | ENSG00000064726 | ENSG00000268798 |
| 609 | ENSG00000169217 | ENSG00000182771 |
| 610 | ENSG00000162461 | ENSG00000168005 |
| 611 | ENSG00000165417 | ENSG00000072736 |
| 612 | ENSG00000170921 | ENSG00000173113 |
| 613 | ENSG00000143379 | ENSG00000174851 |
| 614 | ENSG00000134419 | ENSG00000129194 |
| 615 | ENSG00000164877 | ENSG00000139131 |
| 616 | ENSG00000267909 | ENSG00000105392 |
| 617 | ENSG00000048740 | ENSG00000173262 |
| 618 | ENSG00000134308 | ENSG00000163697 |
| 619 | ENSG00000174276 | ENSG00000198417 |
| 620 | ENSG00000170439 | ENSG00000188051 |
| 621 | ENSG00000109787 | ENSG00000180448 |
| 622 | ENSG00000047579 | ENSG00000102858 |
| 623 | ENSG00000197345 | ENSG00000114779 |
| 624 | ENSG00000235645 | ENSG00000174407 |
| 625 | ENSG00000088756 | ENSG00000095587 |
| 626 | ENSG00000113384 | ENSG00000203747 |
| 627 | ENSG00000137857 | ENSG00000182858 |
| 628 | ENSG00000065717 | ENSG00000167202 |
| 629 | ENSG00000181541 | ENSG00000272772 |
| 630 | ENSG00000136261 | ENSG00000179456 |
| 631 | ENSG00000179922 | ENSG00000147475 |
| 632 | ENSG00000250264 | ENSG00000106636 |
| 633 | ENSG00000011566 | ENSG00000157911 |
| 634 | ENSG00000141101 | ENSG00000169184 |
| 635 | ENSG00000014216 | ENSG00000183657 |
| 636 | ENSG00000134571 | ENSG00000087087 |
| 637 | ENSG00000140943 | ENSG00000188095 |
| 638 | ENSG00000178035 | ENSG00000147488 |
| 639 | ENSG00000123143 | ENSG00000159714 |
| 640 | ENSG00000226174 | ENSG00000147571 |
| 641 | ENSG00000139874 | ENSG00000259784 |
| 642 | ENSG00000184232 | ENSG00000269490 |
| 643 | ENSG00000066056 | ENSG00000167977 |
| 644 | ENSG00000104907 | ENSG00000035862 |
| 645 | ENSG00000145741 | ENSG00000229183 |
| 646 | ENSG00000156113 | ENSG00000086548 |
| 647 | ENSG00000082898 | ENSG00000111077 |
| 648 | ENSG00000138413 | ENSG00000099999 |
| 649 | ENSG00000104213 | ENSG00000161395 |
| 650 | ENSG00000135929 | ENSG00000130701 |
| 651 | ENSG00000203859 | ENSG00000145934 |
| 652 | ENSG00000164403 | ENSG00000140474 |
| 653 | ENSG00000170396 | ENSG00000105467 |
| 654 | ENSG00000244242 | ENSG00000160014 |
| 655 | ENSG00000152292 | ENSG00000172156 |
| 656 | ENSG00000139304 | ENSG00000076248 |
| 657 | ENSG00000131378 | ENSG00000100219 |
| 658 | ENSG00000120051 | ENSG00000215187 |
| 659 | ENSG00000119714 | ENSG00000119396 |
| 660 | ENSG00000133740 | ENSG00000161180 |
| 661 | ENSG00000184389 | ENSG00000131503 |
| 662 | ENSG00000116106 | ENSG00000184481 |
| 663 | ENSG00000174238 | ENSG00000213619 |
| 664 | ENSG00000166035 | ENSG00000165935 |
| 665 | ENSG00000171310 | ENSG00000120705 |
| 666 | ENSG00000092096 | ENSG00000129515 |
| 667 | ENSG00000019995 | ENSG00000093010 |
| 668 | ENSG00000131187 | ENSG00000158220 |
| 669 | ENSG00000136100 | ENSG00000125347 |
| 670 | ENSG00000165655 | ENSG00000138029 |
| 671 | ENSG00000214921 | ENSG00000260851 |
| 672 | ENSG00000048392 | ENSG00000168883 |
| 673 | ENSG00000099617 | ENSG00000134453 |
| 674 | ENSG00000121716 | ENSG00000136274 |
| 675 | ENSG00000273291 | ENSG00000004455 |
| 676 | ENSG00000078369 | ENSG00000119760 |
| 677 | ENSG00000124788 | ENSG00000125462 |
| 678 | ENSG00000139637 | ENSG00000163354 |
| 679 | ENSG00000165072 | ENSG00000078304 |
| 680 | ENSG00000138138 | ENSG00000108846 |
| 681 | ENSG00000086967 | ENSG00000167632 |
| 682 | ENSG00000143632 | ENSG00000112599 |
| 683 | ENSG00000165644 | ENSG00000197081 |
| 684 | ENSG00000125844 | ENSG00000101213 |
| 685 | ENSG00000126249 | ENSG00000133424 |
| 686 | ENSG00000148795 | ENSG00000188229 |
| 687 | ENSG00000163170 | ENSG00000164076 |
| 688 | ENSG00000134460 | ENSG00000168481 |
| 689 | ENSG00000134057 | ENSG00000254413 |
| 690 | ENSG00000101439 | ENSG00000241598 |
| 691 | ENSG00000204278 | ENSG00000073060 |
| 692 | ENSG00000172059 | ENSG00000027075 |
| 693 | ENSG00000116525 | ENSG00000172775 |
| 694 | ENSG00000163521 | ENSG00000167720 |
| 695 | ENSG00000037241 | ENSG00000169564 |
| 696 | ENSG00000182782 | ENSG00000145495 |
| 697 | ENSG00000135090 | ENSG00000047578 |
| 698 | ENSG00000178796 | ENSG00000261427 |
| 699 | ENSG00000168389 | ENSG00000187695 |
| 700 | ENSG00000093167 | ENSG00000108669 |
| 701 | ENSG00000123307 | ENSG00000196154 |
| 702 | ENSG00000258740 | ENSG00000259202 |
| 703 | ENSG00000196284 | ENSG00000010539 |
| 704 | ENSG00000185899 | ENSG00000167460 |
| 705 | ENSG00000117713 | ENSG00000111181 |
| 706 | ENSG00000168298 | ENSG00000132164 |
| 707 | ENSG00000116151 | ENSG00000132819 |
| 708 | ENSG00000152520 | ENSG00000089225 |
| 709 | ENSG00000163644 | ENSG00000055163 |
| 710 | ENSG00000137274 | ENSG00000132591 |
| 711 | ENSG00000181781 | ENSG00000184517 |
| 712 | ENSG00000136827 | ENSG00000100811 |
| 713 | ENSG00000255154 | ENSG00000184012 |
| 714 | ENSG00000125779 | ENSG00000053372 |
| 715 | ENSG00000131370 | ENSG00000134684 |
| 716 | ENSG00000149564 | ENSG00000151702 |
| 717 | ENSG00000230778 | ENSG00000171940 |
| 718 | ENSG00000137522 | ENSG00000113048 |
| 719 | ENSG00000197079 | ENSG00000132323 |
| 720 | ENSG00000203722 | ENSG00000099250 |
| 721 | ENSG00000206047 | ENSG00000141526 |
| 722 | ENSG00000166822 | ENSG00000108691 |
| 723 | ENSG00000068383 | ENSG00000118137 |
| 724 | ENSG00000105261 | ENSG00000101489 |
| 725 | ENSG00000172508 | ENSG00000173702 |
| 726 | ENSG00000108423 | ENSG00000104419 |
| 727 | ENSG00000160055 | ENSG00000260238 |
| 728 | ENSG00000105219 | ENSG00000110756 |
| 729 | ENSG00000138434 | ENSG00000126259 |
| 730 | ENSG00000120162 | ENSG00000095397 |
| 731 | ENSG00000175866 | ENSG00000163703 |
| 732 | ENSG00000138483 | ENSG00000124226 |
| 733 | ENSG00000163513 | ENSG00000132405 |
| 734 | ENSG00000168734 | ENSG00000267987 |
| 735 | ENSG00000110917 | ENSG00000141506 |
| 736 | ENSG00000090382 | ENSG00000182162 |
| 737 | ENSG00000115365 | ENSG00000143869 |
| 738 | ENSG00000118946 | ENSG00000129116 |
| 739 | ENSG00000172995 | ENSG00000185651 |
| 740 | ENSG00000123136 | ENSG00000171621 |
| 741 | ENSG00000108557 | ENSG00000079432 |
| 742 | ENSG00000164106 | ENSG00000167657 |
| 743 | ENSG00000213934 | ENSG00000092871 |
| 744 | ENSG00000198597 | ENSG00000100122 |
| 745 | ENSG00000150753 | ENSG00000246922 |
| 746 | ENSG00000179195 | ENSG00000268024 |
| 747 | ENSG00000105254 | ENSG00000095002 |
| 748 | ENSG00000125944 | ENSG00000151148 |
| 749 | ENSG00000228368 | ENSG00000175544 |
| 750 | ENSG00000114767 | ENSG00000175920 |
| 751 | ENSG00000172531 | ENSG00000171791 |
| 752 | ENSG00000124588 | ENSG00000155330 |
| 753 | ENSG00000177989 | ENSG00000196526 |
| 754 | ENSG00000117266 | ENSG00000178597 |
| 755 | ENSG00000101558 | ENSG00000173918 |
| 756 | ENSG00000092931 | ENSG00000156398 |
| 757 | ENSG00000204704 | ENSG00000161405 |
| 758 | ENSG00000153310 | ENSG00000117090 |
| 759 | ENSG00000121653 | ENSG00000205147 |
| 760 | ENSG00000108839 | ENSG00000159166 |
| 761 | ENSG00000205148 | ENSG00000230989 |
| 762 | ENSG00000101265 | ENSG00000204252 |
| 763 | ENSG00000198837 | ENSG00000124216 |
| 764 | ENSG00000104728 | ENSG00000197142 |
| 765 | ENSG00000117013 | ENSG00000197965 |
| 766 | ENSG00000176986 | ENSG00000067560 |
| 767 | ENSG00000221949 | ENSG00000171992 |
| 768 | ENSG00000182379 | ENSG00000184922 |
| 769 | ENSG00000197329 | ENSG00000072163 |
| 770 | ENSG00000169302 | ENSG00000196628 |
| 771 | ENSG00000269753 | ENSG00000103194 |
| 772 | ENSG00000122025 | ENSG00000043591 |
| 773 | ENSG00000137507 | ENSG00000196666 |
| 774 | ENSG00000074219 | ENSG00000160785 |
| 775 | ENSG00000197312 | ENSG00000176387 |
| 776 | ENSG00000172575 | ENSG00000167487 |
| 777 | ENSG00000198498 | ENSG00000107104 |
| 778 | ENSG00000134061 | ENSG00000161082 |
| 779 | ENSG00000113083 | ENSG00000132613 |
| 780 | ENSG00000105281 | ENSG00000185897 |
| 781 | ENSG00000168746 | ENSG00000149476 |
| 782 | ENSG00000110013 | ENSG00000164535 |
| 783 | ENSG00000104833 | ENSG00000180992 |
| 784 | ENSG00000162441 | ENSG00000160209 |
| 785 | ENSG00000137073 | ENSG00000173153 |
| 786 | ENSG00000130309 | ENSG00000184293 |
| 787 | ENSG00000006837 | ENSG00000033011 |
| 788 | ENSG00000068305 | ENSG00000120725 |
| 789 | ENSG00000085514 | ENSG00000136848 |
| 790 | ENSG00000221821 | ENSG00000160182 |
| 791 | ENSG00000255307 | ENSG00000167779 |
| 792 | ENSG00000083099 | ENSG00000214670 |
| 793 | ENSG00000240682 | ENSG00000137474 |
| 794 | ENSG00000196923 | ENSG00000105700 |
| 795 | ENSG00000181031 | ENSG00000135924 |
| 796 | ENSG00000268628 | ENSG00000177697 |
| 797 | ENSG00000185559 | ENSG00000176692 |
| 798 | ENSG00000185359 | ENSG00000197665 |
| 799 | ENSG00000103876 | ENSG00000105339 |
| 800 | ENSG00000085832 | ENSG00000106617 |
| 801 | ENSG00000185792 | ENSG00000162804 |
| 802 | ENSG00000198846 | ENSG00000142186 |
| 803 | ENSG00000076706 | ENSG00000162616 |
| 804 | ENSG00000204099 | ENSG00000219481 |
| 805 | ENSG00000105223 | ENSG00000174807 |
| 806 | ENSG00000100802 | ENSG00000175287 |
| 807 | ENSG00000125864 | ENSG00000085465 |
| 808 | ENSG00000164124 | ENSG00000182107 |
| 809 | ENSG00000091140 | ENSG00000169991 |
| 810 | ENSG00000140675 | ENSG00000112041 |
| 811 | ENSG00000167701 | ENSG00000266173 |
| 812 | ENSG00000147905 | ENSG00000141748 |
| 813 | ENSG00000126353 | ENSG00000221983 |
| 814 | ENSG00000102125 | ENSG00000108679 |
| 815 | ENSG00000134259 | ENSG00000188888 |
| 816 | ENSG00000084110 | ENSG00000142661 |
| 817 | ENSG00000166444 | ENSG00000143164 |
| 818 | ENSG00000069122 | ENSG00000130475 |
| 819 | ENSG00000125319 | ENSG00000150347 |
| 820 | ENSG00000167925 | ENSG00000110375 |
| 821 | ENSG00000126464 | ENSG00000074660 |
| 822 | ENSG00000105656 | ENSG00000113194 |
| 823 | ENSG00000170515 | ENSG00000172349 |
| 824 | ENSG00000115053 | ENSG00000108641 |
| 825 | ENSG00000076555 | ENSG00000182208 |
| 826 | ENSG00000162366 | ENSG00000177551 |
| 827 | ENSG00000161526 | ENSG00000135482 |
| 828 | ENSG00000167600 | ENSG00000144028 |
| 829 | ENSG00000188886 | ENSG00000268083 |
| 830 | ENSG00000173699 | ENSG00000165929 |
| 831 | ENSG00000204590 | ENSG00000198324 |
| 832 | ENSG00000187531 | ENSG00000177685 |
| 833 | ENSG00000236882 | ENSG00000143543 |
| 834 | ENSG00000061337 | ENSG00000091527 |
| 835 | ENSG00000105289 | ENSG00000242574 |
| 836 | ENSG00000180891 | ENSG00000187607 |
| 837 | ENSG00000096088 | ENSG00000010626 |
| 838 | ENSG00000188037 | ENSG00000153714 |
| 839 | ENSG00000127743 | ENSG00000107140 |
| 840 | ENSG00000143753 | ENSG00000197483 |
| 841 | ENSG00000183032 | ENSG00000166250 |
| 842 | ENSG00000169105 | ENSG00000160803 |
| 843 | ENSG00000101255 | ENSG00000104368 |
| 844 | ENSG00000110328 | ENSG00000142611 |
| 845 | ENSG00000197653 | ENSG00000243147 |
| 846 | ENSG00000253457 | ENSG00000169957 |
| 847 | ENSG00000168772 | ENSG00000127824 |
| 848 | ENSG00000164953 | ENSG00000182687 |
| 849 | ENSG00000204673 | ENSG00000164086 |
| 850 | ENSG00000071242 | ENSG00000174788 |
| 851 | ENSG00000198624 | ENSG00000175727 |
| 852 | ENSG00000112715 | ENSG00000174343 |
| 853 | ENSG00000177628 | ENSG00000163032 |
| 854 | ENSG00000155438 | ENSG00000168090 |
| 855 | ENSG00000175634 | ENSG00000125386 |
| 856 | ENSG00000105376 | ENSG00000140859 |
| 857 | ENSG00000153395 | ENSG00000111671 |
| 858 | ENSG00000072210 | ENSG00000141579 |
| 859 | ENSG00000090674 | ENSG00000138311 |
| 860 | ENSG00000149609 | ENSG00000146535 |
| 861 | ENSG00000184432 | ENSG00000183508 |
| 862 | ENSG00000174059 | ENSG00000111653 |
| 863 | ENSG00000108797 | ENSG00000255104 |
| 864 | ENSG00000111669 | ENSG00000105429 |
| 865 | ENSG00000184602 | ENSG00000093072 |
| 866 | ENSG00000183397 | ENSG00000151532 |
| 867 | ENSG00000130383 | ENSG00000140519 |
| 868 | ENSG00000151360 | ENSG00000145779 |
| 869 | ENSG00000196408 | ENSG00000257923 |
| 870 | ENSG00000251357 | ENSG00000056097 |
| 871 | ENSG00000125531 | ENSG00000138162 |
| 872 | ENSG00000113811 | ENSG00000115306 |
| 873 | ENSG00000105205 | ENSG00000153563 |
| 874 | ENSG00000158985 | ENSG00000272305 |
| 875 | ENSG00000259384 | ENSG00000164849 |
| 876 | ENSG00000114491 | ENSG00000104140 |
| 877 | ENSG00000127951 | ENSG00000116285 |
| 878 | ENSG00000080822 | ENSG00000198077 |
| 879 | ENSG00000108823 | ENSG00000147168 |
| 880 | ENSG00000170456 | ENSG00000178922 |
| 881 | ENSG00000089060 | ENSG00000068654 |
| 882 | ENSG00000173141 | ENSG00000100012 |
| 883 | ENSG00000268280 | ENSG00000111276 |
| 884 | ENSG00000141542 | ENSG00000049759 |
| 885 | ENSG00000155085 | ENSG00000198909 |
| 886 | ENSG00000134291 | ENSG00000100604 |
| 887 | ENSG00000121410 | ENSG00000105323 |
| 888 | ENSG00000072756 | ENSG00000003756 |
| 889 | ENSG00000135974 | ENSG00000170027 |
| 890 | ENSG00000103710 | ENSG00000169016 |
| 891 | ENSG00000103495 | ENSG00000095261 |
| 892 | ENSG00000173705 | ENSG00000203879 |
| 893 | ENSG00000086666 | ENSG00000183971 |
| 894 | ENSG00000140691 | ENSG00000176200 |
| 895 | ENSG00000054938 | ENSG00000156510 |
| 896 | ENSG00000268218 | ENSG00000141971 |
| 897 | ENSG00000164087 | ENSG00000128311 |
| 898 | ENSG00000213145 | ENSG00000136603 |
| 899 | ENSG00000177602 | ENSG00000183569 |
| 900 | ENSG00000108848 | ENSG00000198471 |
| 901 | ENSG00000006607 | ENSG00000108175 |
| 902 | ENSG00000143294 | ENSG00000230567 |
| 903 | ENSG00000178307 | ENSG00000070366 |
| 904 | ENSG00000104760 | ENSG00000178149 |
| 905 | ENSG00000179344 | ENSG00000166741 |
| 906 | ENSG00000116161 | ENSG00000110958 |
| 907 | ENSG00000176731 | ENSG00000133401 |
| 908 | ENSG00000154451 | ENSG00000141279 |
| 909 | ENSG00000116793 | ENSG00000147526 |
| 910 | ENSG00000167397 | ENSG00000249590 |
| 911 | ENSG00000085511 | ENSG00000144560 |
| 912 | ENSG00000128159 | ENSG00000172530 |
| 913 | ENSG00000075391 | ENSG00000187959 |
| 914 | ENSG00000183474 | ENSG00000136205 |
| 915 | ENSG00000143862 | ENSG00000177058 |
| 916 | ENSG00000143315 | ENSG00000143190 |
| 917 | ENSG00000163497 | ENSG00000131188 |
| 918 | ENSG00000175470 | ENSG00000174951 |
| 919 | ENSG00000224361 | ENSG00000114853 |
| 920 | ENSG00000241119 | ENSG00000146006 |
| 921 | ENSG00000144407 | ENSG00000084628 |
| 922 | ENSG00000129353 | ENSG00000156869 |
| 923 | ENSG00000272333 | ENSG00000092820 |
| 924 | ENSG00000167077 | ENSG00000144749 |
| 925 | ENSG00000104823 | ENSG00000129219 |
| 926 | ENSG00000143226 | ENSG00000153531 |
| 927 | ENSG00000153066 | ENSG00000127946 |
| 928 | ENSG00000196979 | ENSG00000087077 |
| 929 | ENSG00000185507 | ENSG00000074416 |
| 930 | ENSG00000164627 | ENSG00000157570 |
| 931 | ENSG00000115825 | ENSG00000183160 |
| 932 | ENSG00000177383 | ENSG00000113068 |
| 933 | ENSG00000151948 | ENSG00000268313 |
| 934 | ENSG00000196917 | ENSG00000156414 |
| 935 | ENSG00000158717 | ENSG00000105388 |
| 936 | ENSG00000102030 | ENSG00000125841 |
| 937 | ENSG00000173812 | ENSG00000109917 |
| 938 | ENSG00000117519 | ENSG00000171124 |
| 939 | ENSG00000139697 | ENSG00000164587 |
| 940 | ENSG00000204120 | ENSG00000147601 |
| 941 | ENSG00000197852 | ENSG00000162520 |
| 942 | ENSG00000180089 | ENSG00000243056 |
| 943 | ENSG00000119888 | ENSG00000198755 |
| 944 | ENSG00000204311 | ENSG00000101384 |
| 945 | ENSG00000174791 | ENSG00000236311 |
| 946 | ENSG00000169609 | ENSG00000197430 |
| 947 | ENSG00000178057 | ENSG00000162542 |
| 948 | ENSG00000160888 | ENSG00000182217 |
| 949 | ENSG00000110536 | ENSG00000132702 |
| 950 | ENSG00000143333 | ENSG00000090013 |
| 951 | ENSG00000180901 | ENSG00000081913 |
| 952 | ENSG00000172171 | ENSG00000168010 |
| 953 | ENSG00000102879 | ENSG00000167173 |
| 954 | ENSG00000018408 | ENSG00000163352 |
| 955 | ENSG00000143570 | ENSG00000154127 |
| 956 | ENSG00000168765 | ENSG00000095139 |
| 957 | ENSG00000204176 | ENSG00000145911 |
| 958 | ENSG00000269570 | ENSG00000039600 |
| 959 | ENSG00000138386 | ENSG00000188305 |
| 960 | ENSG00000143179 | ENSG00000089094 |
| 961 | ENSG00000119922 | ENSG00000136068 |
| 962 | ENSG00000007541 | ENSG00000165714 |
| 963 | ENSG00000067066 | ENSG00000205476 |
| 964 | ENSG00000184857 | ENSG00000160218 |
| 965 | ENSG00000174243 | ENSG00000100426 |
| 966 | ENSG00000174177 | ENSG00000104852 |
| 967 | ENSG00000171862 | ENSG00000188878 |
| 968 | ENSG00000137818 | ENSG00000026025 |
| 969 | ENSG00000125998 | ENSG00000184743 |
| 970 | ENSG00000127804 | ENSG00000112304 |
| 971 | ENSG00000269858 | ENSG00000184502 |
| 972 | ENSG00000187109 | ENSG00000269103 |
| 973 | ENSG00000262003 | ENSG00000149636 |
| 974 | ENSG00000172878 | ENSG00000146122 |
| 975 | ENSG00000136630 | ENSG00000183682 |
| 976 | ENSG00000121578 | ENSG00000118729 |
| 977 | ENSG00000153404 | ENSG00000142227 |
| 978 | ENSG00000167992 | ENSG00000138175 |
| 979 | ENSG00000095015 | ENSG00000162975 |
| 980 | ENSG00000165494 | ENSG00000163435 |
| 981 | ENSG00000114529 | ENSG00000185254 |
| 982 | ENSG00000205089 | ENSG00000119950 |
| 983 | ENSG00000180806 | ENSG00000157045 |
| 984 | ENSG00000151704 | ENSG00000100994 |
| 985 | ENSG00000011600 | ENSG00000205710 |
| 986 | ENSG00000132716 | ENSG00000167625 |
| 987 | ENSG00000108523 | ENSG00000143420 |
| 988 | ENSG00000005243 | ENSG00000104964 |
| 989 | ENSG00000110148 | ENSG00000186298 |
| 990 | ENSG00000105679 | ENSG00000143384 |
| 991 | ENSG00000132522 | ENSG00000141977 |
| 992 | ENSG00000168065 | ENSG00000151150 |
| 993 | ENSG00000150527 | ENSG00000116473 |
| 994 | ENSG00000085788 | ENSG00000268400 |
| 995 | ENSG00000160401 | ENSG00000241360 |
| 996 | ENSG00000268098 | ENSG00000180353 |
| 997 | ENSG00000116337 | ENSG00000110768 |
| 998 | ENSG00000135441 | ENSG00000066027 |
| 999 | ENSG00000215644 | ENSG00000100335 |
| 1000 | ENSG00000168454 | ENSG00000105245 |
| 1001 | ENSG00000130997 | ENSG00000196730 |
| 1002 | ENSG00000168743 | ENSG00000135709 |
| 1003 | ENSG00000197043 | ENSG00000100532 |
| 1004 | ENSG00000022567 | ENSG00000112619 |
| 1005 | ENSG00000105298 | ENSG00000161270 |
| 1006 | ENSG00000162551 | ENSG00000173418 |
| 1007 | ENSG00000263417 | ENSG00000094804 |
| 1008 | ENSG00000197822 | ENSG00000168615 |
| 1009 | ENSG00000165661 | ENSG00000133193 |
| 1010 | ENSG00000180209 | ENSG00000127666 |
| 1011 | ENSG00000268538 | ENSG00000137809 |
| 1012 | ENSG00000160949 | ENSG00000188505 |
| 1013 | ENSG00000118271 | ENSG00000139318 |
| 1014 | ENSG00000165219 | ENSG00000161642 |
| 1015 | ENSG00000166896 | ENSG00000156011 |
| 1016 | ENSG00000214248 | ENSG00000089154 |
| 1017 | ENSG00000153064 | ENSG00000226321 |
| 1018 | ENSG00000196743 | ENSG00000179284 |
| 1019 | ENSG00000139546 | ENSG00000165660 |
| 1020 | ENSG00000183386 | ENSG00000160593 |
| 1021 | ENSG00000175604 | ENSG00000130332 |
| 1022 | ENSG00000119866 | ENSG00000130164 |
| 1023 | ENSG00000257962 | ENSG00000180596 |
| 1024 | ENSG00000078328 | ENSG00000075624 |
| 1025 | ENSG00000259332 | ENSG00000034152 |
| 1026 | ENSG00000121068 | ENSG00000168026 |
| 1027 | ENSG00000198925 | ENSG00000130147 |
| 1028 | ENSG00000164904 | ENSG00000154358 |
| 1029 | ENSG00000173065 | ENSG00000135365 |
| 1030 | ENSG00000111845 | ENSG00000164916 |
| 1031 | ENSG00000139572 | ENSG00000070081 |
| 1032 | ENSG00000154832 | ENSG00000182718 |
| 1033 | ENSG00000139549 | ENSG00000174339 |
| 1034 | ENSG00000204923 | ENSG00000128342 |
| 1035 | ENSG00000042753 | ENSG00000182871 |
| 1036 | ENSG00000184221 | ENSG00000198796 |
| 1037 | ENSG00000198336 | ENSG00000136732 |
| 1038 | ENSG00000134686 | ENSG00000167323 |
| 1039 | ENSG00000205277 | ENSG00000178363 |
| 1040 | ENSG00000132744 | ENSG00000081791 |
| 1041 | ENSG00000173581 | ENSG00000077157 |
| 1042 | ENSG00000123066 | ENSG00000217075 |
| 1043 | ENSG00000167578 | ENSG00000183463 |
| 1044 | ENSG00000172830 | ENSG00000100209 |
| 1045 | ENSG00000137496 | ENSG00000080031 |
| 1046 | ENSG00000268069 | ENSG00000155506 |
| 1047 | ENSG00000178828 | ENSG00000196465 |
| 1048 | ENSG00000241489 | ENSG00000121753 |
| 1049 | ENSG00000171056 | ENSG00000117475 |
| 1050 | ENSG00000105677 | ENSG00000240583 |
| 1051 | ENSG00000134817 | ENSG00000173068 |
| 1052 | ENSG00000131143 | ENSG00000268553 |
| 1053 | ENSG00000171794 | ENSG00000012124 |
| 1054 | ENSG00000140548 | ENSG00000181409 |
| 1055 | ENSG00000228278 | ENSG00000107863 |
| 1056 | ENSG00000123411 | ENSG00000102471 |
| 1057 | ENSG00000197530 | ENSG00000137393 |
| 1058 | ENSG00000131899 | ENSG00000162390 |
| 1059 | ENSG00000171055 | ENSG00000167363 |
| 1060 | ENSG00000198563 | ENSG00000100439 |
| 1061 | ENSG00000214597 | ENSG00000131055 |
| 1062 | ENSG00000243725 | ENSG00000260903 |
| 1063 | ENSG00000149243 | ENSG00000164543 |
| 1064 | ENSG00000197417 | ENSG00000075073 |
| 1065 | ENSG00000167971 | ENSG00000230510 |
| 1066 | ENSG00000013725 | ENSG00000166897 |
| 1067 | ENSG00000184056 | ENSG00000166246 |
| 1068 | ENSG00000113812 | ENSG00000183426 |
| 1069 | ENSG00000164924 | ENSG00000143479 |
| 1070 | ENSG00000178821 | ENSG00000165272 |
| 1071 | ENSG00000198740 | ENSG00000231880 |
| 1072 | ENSG00000127528 | ENSG00000131941 |
| 1073 | ENSG00000226180 | ENSG00000136425 |
| 1074 | ENSG00000215883 | ENSG00000165807 |
| 1075 | ENSG00000145476 | ENSG00000083857 |
| 1076 | ENSG00000142867 | ENSG00000178467 |
| 1077 | ENSG00000205116 | ENSG00000099625 |
| 1078 | ENSG00000214960 | ENSG00000144057 |
| 1079 | ENSG00000149633 | ENSG00000079459 |
| 1080 | ENSG00000185519 | ENSG00000178404 |
| 1081 | ENSG00000107159 | ENSG00000119408 |
| 1082 | ENSG00000011304 | ENSG00000267618 |
| 1083 | ENSG00000198824 | ENSG00000149043 |
| 1084 | ENSG00000100060 | ENSG00000043143 |
| 1085 | ENSG00000162458 | ENSG00000228008 |
| 1086 | ENSG00000013374 | ENSG00000183111 |
| 1087 | ENSG00000205318 | ENSG00000020577 |
| 1088 | ENSG00000182545 | ENSG00000178607 |
| 1089 | ENSG00000165724 | ENSG00000122958 |
| 1090 | ENSG00000105991 | ENSG00000179058 |
| 1091 | ENSG00000187720 | ENSG00000115183 |
| 1092 | ENSG00000161860 | ENSG00000196588 |
| 1093 | ENSG00000127578 | ENSG00000176248 |
| 1094 | ENSG00000038532 | ENSG00000186567 |
| 1095 | ENSG00000105647 | ENSG00000002330 |
| 1096 | ENSG00000268798 | ENSG00000067955 |
| 1097 | ENSG00000166478 | ENSG00000198246 |
| 1098 | ENSG00000072736 | ENSG00000172403 |
| 1099 | ENSG00000173113 | ENSG00000137288 |
| 1100 | ENSG00000046604 | ENSG00000197364 |
| 1101 | ENSG00000129194 | ENSG00000154124 |
| 1102 | ENSG00000174851 | ENSG00000015133 |
| 1103 | ENSG00000139131 | ENSG00000136059 |
| 1104 | ENSG00000105392 | ENSG00000088543 |
| 1105 | ENSG00000138675 | ENSG00000008311 |
| 1106 | ENSG00000170961 | ENSG00000140105 |
| 1107 | ENSG00000175792 | ENSG00000135537 |
| 1108 | ENSG00000169696 | ENSG00000174992 |
| 1109 | ENSG00000164898 | ENSG00000166823 |
| 1110 | ENSG00000267873 | ENSG00000173786 |
| 1111 | ENSG00000006282 | ENSG00000137337 |
| 1112 | ENSG00000198417 | ENSG00000221955 |
| 1113 | ENSG00000135318 | ENSG00000049239 |
| 1114 | ENSG00000188051 | ENSG00000266208 |
| 1115 | ENSG00000181036 | ENSG00000172935 |
| 1116 | ENSG00000267952 | ENSG00000185955 |
| 1117 | ENSG00000180448 | ENSG00000144837 |
| 1118 | ENSG00000179673 | ENSG00000151065 |
| 1119 | ENSG00000102858 | ENSG00000181523 |
| 1120 | ENSG00000114779 | ENSG00000105939 |
| 1121 | ENSG00000172724 | ENSG00000104067 |
| 1122 | ENSG00000174407 | ENSG00000100038 |
| 1123 | ENSG00000187186 | ENSG00000167851 |
| 1124 | ENSG00000095587 | ENSG00000008294 |
| 1125 | ENSG00000264324 | ENSG00000198625 |
| 1126 | ENSG00000203747 | ENSG00000149582 |
| 1127 | ENSG00000182858 | ENSG00000100314 |
| 1128 | ENSG00000272772 | ENSG00000120278 |
| 1129 | ENSG00000110066 | ENSG00000100413 |
| 1130 | ENSG00000167202 | ENSG00000166507 |
| 1131 | ENSG00000147475 | ENSG00000229474 |
| 1132 | ENSG00000179456 | ENSG00000149577 |
| 1133 | ENSG00000080819 | ENSG00000167654 |
| 1134 | ENSG00000100024 | ENSG00000063241 |
| 1135 | ENSG00000204531 | ENSG00000114738 |
| 1136 | ENSG00000162040 | ENSG00000105193 |
| 1137 | ENSG00000158773 | ENSG00000170348 |
| 1138 | ENSG00000117425 | ENSG00000115594 |
| 1139 | ENSG00000124198 | ENSG00000226397 |
| 1140 | ENSG00000147488 | ENSG00000216490 |
| 1141 | ENSG00000185100 | ENSG00000108684 |
| 1142 | ENSG00000115841 | ENSG00000259120 |
| 1143 | ENSG00000159714 | ENSG00000185339 |
| 1144 | ENSG00000147571 | ENSG00000122566 |
| 1145 | ENSG00000259784 | ENSG00000196782 |
| 1146 | ENSG00000143127 | ENSG00000111300 |
| 1147 | ENSG00000143375 | ENSG00000187049 |
| 1148 | ENSG00000125375 | ENSG00000091436 |
| 1149 | ENSG00000179111 | ENSG00000020633 |
| 1150 | ENSG00000167977 | ENSG00000104133 |
| 1151 | ENSG00000271698 | ENSG00000127952 |
| 1152 | ENSG00000035862 | ENSG00000165914 |
| 1153 | ENSG00000163812 | ENSG00000106991 |
| 1154 | ENSG00000086548 | ENSG00000125810 |
| 1155 | ENSG00000229183 | ENSG00000110090 |
| 1156 | ENSG00000133678 | ENSG00000137225 |
| 1157 | ENSG00000122783 | ENSG00000130382 |
| 1158 | ENSG00000111077 | ENSG00000142273 |
| 1159 | ENSG00000159884 | ENSG00000231672 |
| 1160 | ENSG00000182272 | ENSG00000163702 |
| 1161 | ENSG00000099999 | ENSG00000182054 |
| 1162 | ENSG00000161395 | ENSG00000221986 |
| 1163 | ENSG00000145934 | ENSG00000133863 |
| 1164 | ENSG00000130701 | ENSG00000128965 |
| 1165 | ENSG00000215704 | ENSG00000268217 |
| 1166 | ENSG00000161091 | ENSG00000100865 |
| 1167 | ENSG00000140474 | ENSG00000106009 |
| 1168 | ENSG00000259518 | ENSG00000258366 |
| 1169 | ENSG00000108518 | ENSG00000189171 |
| 1170 | ENSG00000273167 | ENSG00000155034 |
| 1171 | ENSG00000134470 | ENSG00000172742 |
| 1172 | ENSG00000160014 | ENSG00000158715 |
| 1173 | ENSG00000204577 | ENSG00000174567 |
| 1174 | ENSG00000222040 | ENSG00000175482 |
| 1175 | ENSG00000132793 | ENSG00000102103 |
| 1176 | ENSG00000076248 | ENSG00000100345 |
| 1177 | ENSG00000100219 | ENSG00000159713 |
| 1178 | ENSG00000215187 | ENSG00000099840 |
| 1179 | ENSG00000102931 | ENSG00000092531 |
| 1180 | ENSG00000119396 | ENSG00000130702 |
| 1181 | ENSG00000161180 | ENSG00000156735 |
| 1182 | ENSG00000162300 | ENSG00000107960 |
| 1183 | ENSG00000213619 | ENSG00000171227 |
| 1184 | ENSG00000165935 | ENSG00000164620 |
| 1185 | ENSG00000163467 | ENSG00000109099 |
| 1186 | ENSG00000129515 | ENSG00000169594 |
| 1187 | ENSG00000125347 | ENSG00000140992 |
| 1188 | ENSG00000123096 | ENSG00000008838 |
| 1189 | ENSG00000175221 | ENSG00000163933 |
| 1190 | ENSG00000091138 | ENSG00000204592 |
| 1191 | ENSG00000101187 | ENSG00000160862 |
| 1192 | ENSG00000260851 | ENSG00000063015 |
| 1193 | ENSG00000119929 | ENSG00000072786 |
| 1194 | ENSG00000128594 | ENSG00000198719 |
| 1195 | ENSG00000185803 | ENSG00000184216 |
| 1196 | ENSG00000125977 | ENSG00000187624 |
| 1197 | ENSG00000134453 | ENSG00000196323 |
| 1198 | ENSG00000004455 | ENSG00000167110 |
| 1199 | ENSG00000175315 | ENSG00000159625 |
| 1200 | ENSG00000077782 | ENSG00000154310 |
| 1201 | ENSG00000073670 | ENSG00000007306 |
| 1202 | ENSG00000151577 | ENSG00000185033 |
| 1203 | ENSG00000119760 | ENSG00000128228 |
| 1204 | ENSG00000125462 | ENSG00000234949 |
| 1205 | ENSG00000163354 | ENSG00000150991 |
| 1206 | ENSG00000138964 | ENSG00000130402 |
| 1207 | ENSG00000078304 | ENSG00000268991 |
| 1208 | ENSG00000178381 | ENSG00000268509 |
| 1209 | ENSG00000108846 | ENSG00000167721 |
| 1210 | ENSG00000167632 | ENSG00000134183 |
| 1211 | ENSG00000112599 | ENSG00000164707 |
| 1212 | ENSG00000197081 | ENSG00000081760 |
| 1213 | ENSG00000129480 | ENSG00000071677 |
| 1214 | ENSG00000133424 | ENSG00000185043 |
| 1215 | ENSG00000101213 | ENSG00000169994 |
| 1216 | ENSG00000068615 | ENSG00000168487 |
| 1217 | ENSG00000163486 | ENSG00000129951 |
| 1218 | ENSG00000164076 | ENSG00000214655 |
| 1219 | ENSG00000122877 | ENSG00000167641 |
| 1220 | ENSG00000187461 | ENSG00000126803 |
| 1221 | ENSG00000159674 | ENSG00000139610 |
| 1222 | ENSG00000124159 | ENSG00000182810 |
| 1223 | ENSG00000078246 | ENSG00000087903 |
| 1224 | ENSG00000166106 | ENSG00000144736 |
| 1225 | ENSG00000239282 | ENSG00000182866 |
| 1226 | ENSG00000127415 | ENSG00000124783 |
| 1227 | ENSG00000127399 | ENSG00000133059 |
| 1228 | ENSG00000106415 | ENSG00000099991 |
| 1229 | ENSG00000254413 | ENSG00000183751 |
| 1230 | ENSG00000241598 | ENSG00000111275 |
| 1231 | ENSG00000167685 | ENSG00000204957 |
| 1232 | ENSG00000073060 | ENSG00000147570 |
| 1233 | ENSG00000111266 | ENSG00000163956 |
| 1234 | ENSG00000258529 | ENSG00000100354 |
| 1235 | ENSG00000027075 | ENSG00000253426 |
| 1236 | ENSG00000196591 | ENSG00000153789 |
| 1237 | ENSG00000091640 | ENSG00000109991 |
| 1238 | ENSG00000172775 | ENSG00000255073 |
| 1239 | ENSG00000188026 | ENSG00000168118 |
| 1240 | ENSG00000170091 | ENSG00000162174 |
| 1241 | ENSG00000144959 | ENSG00000267740 |
| 1242 | ENSG00000258150 | ENSG00000175567 |
| 1243 | ENSG00000111737 | ENSG00000141905 |
| 1244 | ENSG00000174720 | ENSG00000245888 |
| 1245 | ENSG00000167720 | ENSG00000162896 |
| 1246 | ENSG00000169564 | ENSG00000167515 |
| 1247 | ENSG00000198668 | ENSG00000268965 |
| 1248 | ENSG00000067167 | ENSG00000111186 |
| 1249 | ENSG00000047578 | ENSG00000141858 |
| 1250 | ENSG00000100628 | ENSG00000259753 |
| 1251 | ENSG00000214046 | ENSG00000197380 |
| 1252 | ENSG00000261427 | ENSG00000126091 |
| 1253 | ENSG00000100395 | ENSG00000122705 |
| 1254 | ENSG00000108669 | ENSG00000037897 |
| 1255 | ENSG00000243708 | ENSG00000167604 |
| 1256 | ENSG00000136045 | ENSG00000162545 |
| 1257 | ENSG00000164366 | ENSG00000174903 |
| 1258 | ENSG00000188001 | ENSG00000188686 |
| 1259 | ENSG00000196154 | ENSG00000181577 |
| 1260 | ENSG00000214978 | ENSG00000182667 |
| 1261 | ENSG00000097021 | ENSG00000119682 |
| 1262 | ENSG00000108759 | ENSG00000156411 |
| 1263 | ENSG00000149489 | ENSG00000105723 |
| 1264 | ENSG00000139220 | ENSG00000205436 |
| 1265 | ENSG00000259202 | ENSG00000173917 |
| 1266 | ENSG00000166090 | ENSG00000088367 |
| 1267 | ENSG00000010539 | ENSG00000085721 |
| 1268 | ENSG00000070371 | ENSG00000228208 |
| 1269 | ENSG00000154165 | ENSG00000181449 |
| 1270 | ENSG00000167460 | ENSG00000120094 |
| 1271 | ENSG00000006047 | ENSG00000101150 |
| 1272 | ENSG00000012048 | ENSG00000173614 |
| 1273 | ENSG00000111181 | ENSG00000272762 |
| 1274 | ENSG00000132164 | ENSG00000138135 |
| 1275 | ENSG00000132819 | ENSG00000149599 |
| 1276 | ENSG00000089225 | ENSG00000143595 |
| 1277 | ENSG00000134758 | ENSG00000071051 |
| 1278 | ENSG00000120451 | ENSG00000198520 |
| 1279 | ENSG00000072195 | ENSG00000129221 |
| 1280 | ENSG00000132591 | ENSG00000173728 |
| 1281 | ENSG00000055163 | ENSG00000113916 |
| 1282 | ENSG00000205358 | ENSG00000176953 |
| 1283 | ENSG00000158079 | ENSG00000185000 |
| 1284 | ENSG00000184517 | ENSG00000183726 |
| 1285 | ENSG00000184012 | ENSG00000160185 |
| 1286 | ENSG00000100811 | ENSG00000165215 |
| 1287 | ENSG00000053372 | ENSG00000170776 |
| 1288 | ENSG00000134684 | ENSG00000179066 |
| 1289 | ENSG00000151702 | ENSG00000118094 |
| 1290 | ENSG00000171940 | ENSG00000135404 |
| 1291 | ENSG00000262304 | ENSG00000141873 |
| 1292 | ENSG00000113048 | ENSG00000142319 |
| 1293 | ENSG00000099364 | ENSG00000125888 |
| 1294 | ENSG00000132323 | ENSG00000120063 |
| 1295 | ENSG00000099250 | ENSG00000182551 |
| 1296 | ENSG00000205693 | ENSG00000173727 |
| 1297 | ENSG00000183020 | ENSG00000132326 |
| 1298 | ENSG00000108788 | ENSG00000148843 |
| 1299 | ENSG00000161980 | ENSG00000021762 |
| 1300 | ENSG00000136156 | ENSG00000073536 |
| 1301 | ENSG00000176160 | ENSG00000105559 |
| 1302 | ENSG00000151240 | ENSG00000221946 |
| 1303 | ENSG00000141526 | ENSG00000182327 |
| 1304 | ENSG00000108932 | ENSG00000092841 |
| 1305 | ENSG00000135298 | ENSG00000087152 |
| 1306 | ENSG00000118137 | ENSG00000166831 |
| 1307 | ENSG00000149292 | ENSG00000241978 |
| 1308 | ENSG00000173702 | ENSG00000174886 |
| 1309 | ENSG00000155090 | ENSG00000258311 |
| 1310 | ENSG00000160752 | ENSG00000172725 |
| 1311 | ENSG00000145990 | ENSG00000104164 |
| 1312 | ENSG00000101298 | ENSG00000101363 |
| 1313 | ENSG00000104419 | ENSG00000266728 |
| 1314 | ENSG00000189058 | ENSG00000143320 |
| 1315 | ENSG00000151632 | ENSG00000130706 |
| 1316 | ENSG00000104671 | ENSG00000143126 |
| 1317 | ENSG00000121057 | ENSG00000233493 |
| 1318 | ENSG00000126259 | ENSG00000101350 |
| 1319 | ENSG00000198363 | ENSG00000101493 |
| 1320 | ENSG00000165501 | ENSG00000182698 |
| 1321 | ENSG00000095397 | ENSG00000136918 |
| 1322 | ENSG00000220201 | ENSG00000089063 |
| 1323 | ENSG00000151023 | ENSG00000110244 |
| 1324 | ENSG00000120963 | ENSG00000114353 |
| 1325 | ENSG00000132405 | ENSG00000141560 |
| 1326 | ENSG00000267987 | ENSG00000131236 |
| 1327 | ENSG00000255823 | ENSG00000126787 |
| 1328 | ENSG00000141506 | ENSG00000196355 |
| 1329 | ENSG00000256316 | ENSG00000269605 |
| 1330 | ENSG00000143869 | ENSG00000145901 |
| 1331 | ENSG00000142025 | ENSG00000119599 |
| 1332 | ENSG00000129116 | ENSG00000188895 |
| 1333 | ENSG00000105146 | ENSG00000168890 |
| 1334 | ENSG00000258653 | ENSG00000082516 |
| 1335 | ENSG00000103496 | ENSG00000167767 |
| 1336 | ENSG00000171621 | ENSG00000267206 |
| 1337 | ENSG00000079432 | ENSG00000227729 |
| 1338 | ENSG00000099814 | ENSG00000168899 |
| 1339 | ENSG00000185627 | ENSG00000171865 |
| 1340 | ENSG00000167657 | ENSG00000142507 |
| 1341 | ENSG00000092871 | ENSG00000099785 |
| 1342 | ENSG00000234965 | ENSG00000120318 |
| 1343 | ENSG00000084693 | ENSG00000256530 |
| 1344 | ENSG00000134107 | ENSG00000203791 |
| 1345 | ENSG00000154134 | ENSG00000153443 |
| 1346 | ENSG00000155269 | ENSG00000129518 |
| 1347 | ENSG00000159445 | ENSG00000167139 |
| 1348 | ENSG00000071894 | ENSG00000167895 |
| 1349 | ENSG00000100122 | ENSG00000043355 |
| 1350 | ENSG00000164603 | ENSG00000183615 |
| 1351 | ENSG00000165506 | ENSG00000108588 |
| 1352 | ENSG00000100075 | ENSG00000177108 |
| 1353 | ENSG00000183542 | ENSG00000221926 |
| 1354 | ENSG00000246922 | ENSG00000130204 |
| 1355 | ENSG00000095002 | ENSG00000141753 |
| 1356 | ENSG00000151148 | ENSG00000151414 |
| 1357 | ENSG00000175544 | ENSG00000065970 |
| 1358 | ENSG00000160746 | ENSG00000137193 |
| 1359 | ENSG00000171791 | ENSG00000087088 |
| 1360 | ENSG00000107929 | ENSG00000104826 |
| 1361 | ENSG00000170540 | ENSG00000103932 |
| 1362 | ENSG00000121101 | ENSG00000136574 |
| 1363 | ENSG00000100221 | ENSG00000154122 |
| 1364 | ENSG00000171604 | ENSG00000126247 |
| 1365 | ENSG00000146425 | ENSG00000177464 |
| 1366 | ENSG00000196526 | ENSG00000178882 |
| 1367 | ENSG00000131849 | ENSG00000079337 |
| 1368 | ENSG00000169851 | ENSG00000163626 |
| 1369 | ENSG00000121905 | ENSG00000156515 |
| 1370 | ENSG00000205740 | ENSG00000176842 |
| 1371 | ENSG00000175485 | ENSG00000125753 |
| 1372 | ENSG00000103355 | ENSG00000173950 |
| 1373 | ENSG00000156398 | ENSG00000114279 |
| 1374 | ENSG00000173918 | ENSG00000157851 |
| 1375 | ENSG00000117090 | ENSG00000130560 |
| 1376 | ENSG00000179361 | ENSG00000163217 |
| 1377 | ENSG00000258465 | ENSG00000186583 |
| 1378 | ENSG00000205147 | ENSG00000153944 |
| 1379 | ENSG00000273398 | ENSG00000262601 |
| 1380 | ENSG00000095951 | ENSG00000180834 |
| 1381 | ENSG00000159166 | ENSG00000105971 |
| 1382 | ENSG00000205517 | ENSG00000130304 |
| 1383 | ENSG00000019505 | ENSG00000161664 |
| 1384 | ENSG00000204252 | ENSG00000116833 |
| 1385 | ENSG00000124216 | ENSG00000169154 |
| 1386 | ENSG00000198746 | ENSG00000173821 |
| 1387 | ENSG00000181027 | ENSG00000134871 |
| 1388 | ENSG00000083812 | ENSG00000116176 |
| 1389 | ENSG00000064692 | ENSG00000196189 |
| 1390 | ENSG00000127774 | ENSG00000135111 |
| 1391 | ENSG00000100385 | ENSG00000136280 |
| 1392 | ENSG00000198851 | ENSG00000204628 |
| 1393 | ENSG00000197142 | ENSG00000143303 |
| 1394 | ENSG00000232941 | ENSG00000171643 |
| 1395 | ENSG00000198879 | ENSG00000135100 |
| 1396 | ENSG00000197965 | ENSG00000204518 |
| 1397 | ENSG00000182154 | ENSG00000182853 |
| 1398 | ENSG00000067560 | ENSG00000089685 |
| 1399 | ENSG00000118322 | ENSG00000198554 |
| 1400 | ENSG00000171992 | ENSG00000255872 |
| 1401 | ENSG00000065809 | ENSG00000141933 |
| 1402 | ENSG00000184922 | ENSG00000164107 |
| 1403 | ENSG00000072163 | ENSG00000070423 |
| 1404 | ENSG00000155016 | ENSG00000182934 |
| 1405 | ENSG00000196628 | ENSG00000122787 |
| 1406 | ENSG00000138286 | ENSG00000196230 |
| 1407 | ENSG00000103194 | ENSG00000204560 |
| 1408 | ENSG00000156206 | ENSG00000163638 |
| 1409 | ENSG00000180539 | ENSG00000106351 |
| 1410 | ENSG00000224854 | ENSG00000118898 |
| 1411 | ENSG00000167543 | ENSG00000103248 |
| 1412 | ENSG00000196666 | ENSG00000137509 |
| 1413 | ENSG00000073737 | ENSG00000073598 |
| 1414 | ENSG00000131591 | ENSG00000248098 |
| 1415 | ENSG00000137494 | ENSG00000187164 |
| 1416 | ENSG00000176387 | ENSG00000177225 |
| 1417 | ENSG00000170144 | ENSG00000048707 |
| 1418 | ENSG00000037637 | ENSG00000196739 |
| 1419 | ENSG00000099849 | ENSG00000182606 |
| 1420 | ENSG00000107104 | ENSG00000198792 |
| 1421 | ENSG00000161082 | ENSG00000127463 |
| 1422 | ENSG00000132613 | ENSG00000167565 |
| 1423 | ENSG00000185897 | ENSG00000100311 |
| 1424 | ENSG00000149476 | ENSG00000004399 |
| 1425 | ENSG00000110400 | ENSG00000184154 |
| 1426 | ENSG00000180992 | ENSG00000177030 |
| 1427 | ENSG00000267426 | ENSG00000137821 |
| 1428 | ENSG00000205670 | ENSG00000171282 |
| 1429 | ENSG00000154813 | ENSG00000148498 |
| 1430 | ENSG00000142405 | ENSG00000134440 |
| 1431 | ENSG00000132661 | ENSG00000139926 |
| 1432 | ENSG00000173153 | ENSG00000100902 |
| 1433 | ENSG00000184293 | ENSG00000090932 |
| 1434 | ENSG00000033011 | ENSG00000131848 |
| 1435 | ENSG00000185686 | ENSG00000187244 |
| 1436 | ENSG00000168958 | ENSG00000135932 |
| 1437 | ENSG00000175779 | ENSG00000179954 |
| 1438 | ENSG00000167779 | ENSG00000119280 |
| 1439 | ENSG00000214670 | ENSG00000269879 |
| 1440 | ENSG00000167183 | ENSG00000099795 |
| 1441 | ENSG00000121807 | ENSG00000101161 |
| 1442 | ENSG00000155659 | ENSG00000168781 |
| 1443 | ENSG00000110042 | ENSG00000173714 |
| 1444 | ENSG00000105700 | ENSG00000160271 |
| 1445 | ENSG00000111667 | ENSG00000089737 |
| 1446 | ENSG00000179840 | ENSG00000033030 |
| 1447 | ENSG00000135924 | ENSG00000164961 |
| 1448 | ENSG00000177697 | ENSG00000159176 |
| 1449 | ENSG00000141052 | ENSG00000136003 |
| 1450 | ENSG00000176692 | ENSG00000161888 |
| 1451 | ENSG00000197665 | ENSG00000111057 |
| 1452 | ENSG00000103249 | ENSG00000125900 |
| 1453 | ENSG00000150526 | ENSG00000268412 |
| 1454 | ENSG00000105339 | ENSG00000171631 |
| 1455 | ENSG00000151090 | ENSG00000126804 |
| 1456 | ENSG00000106617 | ENSG00000110799 |
| 1457 | ENSG00000178734 | ENSG00000103326 |
| 1458 | ENSG00000162804 | ENSG00000101290 |
| 1459 | ENSG00000101335 | ENSG00000106789 |
| 1460 | ENSG00000162616 | ENSG00000130340 |
| 1461 | ENSG00000142186 | ENSG00000267001 |
| 1462 | ENSG00000204978 | ENSG00000158104 |
| 1463 | ENSG00000219481 | ENSG00000134046 |
| 1464 | ENSG00000186074 | ENSG00000197880 |
| 1465 | ENSG00000137804 | ENSG00000127603 |
| 1466 | ENSG00000232070 | ENSG00000163803 |
| 1467 | ENSG00000174807 | ENSG00000100319 |
| 1468 | ENSG00000124789 | ENSG00000214063 |
| 1469 | ENSG00000163806 | ENSG00000108829 |
| 1470 | ENSG00000117151 | ENSG00000264813 |
| 1471 | ENSG00000182107 | ENSG00000108001 |
| 1472 | ENSG00000165478 | ENSG00000147471 |
| 1473 | ENSG00000170616 | ENSG00000248099 |
| 1474 | ENSG00000175274 | ENSG00000065361 |
| 1475 | ENSG00000186150 | ENSG00000063322 |
| 1476 | ENSG00000196166 | ENSG00000171570 |
| 1477 | ENSG00000156976 | ENSG00000107731 |
| 1478 | ENSG00000162913 | ENSG00000233198 |
| 1479 | ENSG00000266173 | ENSG00000022267 |
| 1480 | ENSG00000147813 | ENSG00000148335 |
| 1481 | ENSG00000140030 | ENSG00000166501 |
| 1482 | ENSG00000141748 | ENSG00000104635 |
| 1483 | ENSG00000080298 | ENSG00000185338 |
| 1484 | ENSG00000221983 | ENSG00000167671 |
| 1485 | ENSG00000108679 | ENSG00000011105 |
| 1486 | ENSG00000183473 | ENSG00000114737 |
| 1487 | ENSG00000142661 | ENSG00000249240 |
| 1488 | ENSG00000161654 | ENSG00000189067 |
| 1489 | ENSG00000110375 | ENSG00000213780 |
| 1490 | ENSG00000116981 | ENSG00000010379 |
| 1491 | ENSG00000204038 | ENSG00000203630 |
| 1492 | ENSG00000109063 | ENSG00000148180 |
| 1493 | ENSG00000273047 | ENSG00000204323 |
| 1494 | ENSG00000172349 | ENSG00000160050 |
| 1495 | ENSG00000108641 | ENSG00000134317 |
| 1496 | ENSG00000177889 | ENSG00000137168 |
| 1497 | ENSG00000182208 | ENSG00000129028 |
| 1498 | ENSG00000100055 | ENSG00000118369 |
| 1499 | ENSG00000135482 | ENSG00000120738 |
| 1500 | ENSG00000179397 | ENSG00000206562 |
| 1501 | ENSG00000087253 | ENSG00000187713 |
| 1502 | ENSG00000144028 | ENSG00000150687 |
| 1503 | ENSG00000179163 | ENSG00000268457 |
| 1504 | ENSG00000217825 | ENSG00000204178 |
| 1505 | ENSG00000161610 | ENSG00000084112 |
| 1506 | ENSG00000268083 | ENSG00000197746 |
| 1507 | ENSG00000179010 | ENSG00000145020 |
| 1508 | ENSG00000198324 | ENSG00000029153 |
| 1509 | ENSG00000131653 | ENSG00000101407 |
| 1510 | ENSG00000123091 | ENSG00000107485 |
| 1511 | ENSG00000177685 | ENSG00000197162 |
| 1512 | ENSG00000125447 | ENSG00000019582 |
| 1513 | ENSG00000158850 | ENSG00000060566 |
| 1514 | ENSG00000087299 | ENSG00000163545 |
| 1515 | ENSG00000143543 | ENSG00000108821 |
| 1516 | ENSG00000175325 | ENSG00000160953 |
| 1517 | ENSG00000242574 | ENSG00000167703 |
| 1518 | ENSG00000187607 | ENSG00000213922 |
| 1519 | ENSG00000010626 | ENSG00000198626 |
| 1520 | ENSG00000153714 | ENSG00000140522 |
| 1521 | ENSG00000184811 | ENSG00000007372 |
| 1522 | ENSG00000120256 | ENSG00000105808 |
| 1523 | ENSG00000120306 | ENSG00000072110 |
| 1524 | ENSG00000107140 | ENSG00000161204 |
| 1525 | ENSG00000197483 | ENSG00000130037 |
| 1526 | ENSG00000175121 | ENSG00000104825 |
| 1527 | ENSG00000160803 | ENSG00000117676 |
| 1528 | ENSG00000105379 | ENSG00000170345 |
| 1529 | ENSG00000166250 | ENSG00000185640 |
| 1530 | ENSG00000234545 | ENSG00000048545 |
| 1531 | ENSG00000149658 | ENSG00000255468 |
| 1532 | ENSG00000198805 | ENSG00000233927 |
| 1533 | ENSG00000104368 | ENSG00000105576 |
| 1534 | ENSG00000137819 | ENSG00000177311 |
| 1535 | ENSG00000142611 | ENSG00000248235 |
| 1536 | ENSG00000243147 | ENSG00000175643 |
| 1537 | ENSG00000108055 | ENSG00000112624 |
| 1538 | ENSG00000054611 | ENSG00000162496 |
| 1539 | ENSG00000169957 | ENSG00000165917 |
| 1540 | ENSG00000127824 | ENSG00000139055 |
| 1541 | ENSG00000151743 | ENSG00000128294 |
| 1542 | ENSG00000179564 | ENSG00000148120 |
| 1543 | ENSG00000175727 | ENSG00000106976 |
| 1544 | ENSG00000177335 | ENSG00000131446 |
| 1545 | ENSG00000064270 | ENSG00000108551 |
| 1546 | ENSG00000224132 | ENSG00000124212 |
| 1547 | ENSG00000177984 | ENSG00000197595 |
| 1548 | ENSG00000174343 | ENSG00000159256 |
| 1549 | ENSG00000111796 | ENSG00000171823 |
| 1550 | ENSG00000152061 | ENSG00000103544 |
| 1551 | ENSG00000055957 | ENSG00000159761 |
| 1552 | ENSG00000269657 | ENSG00000115970 |
| 1553 | ENSG00000197580 | ENSG00000128309 |
| 1554 | ENSG00000140859 | ENSG00000122733 |
| 1555 | ENSG00000134917 | ENSG00000109654 |
| 1556 | ENSG00000111671 | ENSG00000127152 |
| 1557 | ENSG00000141579 | ENSG00000149218 |
| 1558 | ENSG00000139636 | ENSG00000101425 |
| 1559 | ENSG00000141295 | ENSG00000167461 |
| 1560 | ENSG00000183508 | ENSG00000143314 |
| 1561 | ENSG00000175336 | ENSG00000120057 |
| 1562 | ENSG00000133800 | ENSG00000073067 |
| 1563 | ENSG00000103066 | ENSG00000157110 |
| 1564 | ENSG00000102362 | ENSG00000169756 |
| 1565 | ENSG00000116852 | ENSG00000233502 |
| 1566 | ENSG00000059691 | ENSG00000130158 |
| 1567 | ENSG00000255104 | ENSG00000072062 |
| 1568 | ENSG00000138650 | ENSG00000137747 |
| 1569 | ENSG00000105429 | ENSG00000105464 |
| 1570 | ENSG00000093072 | ENSG00000123388 |
| 1571 | ENSG00000258945 | ENSG00000169554 |
| 1572 | ENSG00000151532 | ENSG00000133063 |
| 1573 | ENSG00000115641 | ENSG00000129932 |
| 1574 | ENSG00000140519 | ENSG00000155666 |
| 1575 | ENSG00000197714 | ENSG00000136859 |
| 1576 | ENSG00000257923 | ENSG00000154920 |
| 1577 | ENSG00000115306 | ENSG00000100979 |
| 1578 | ENSG00000109089 | ENSG00000128805 |
| 1579 | ENSG00000063244 | ENSG00000099817 |
| 1580 | ENSG00000164849 | ENSG00000105889 |
| 1581 | ENSG00000104140 | ENSG00000137491 |
| 1582 | ENSG00000116285 | ENSG00000110713 |
| 1583 | ENSG00000132376 | ENSG00000187867 |
| 1584 | ENSG00000198077 | ENSG00000164307 |
| 1585 | ENSG00000100012 | ENSG00000183856 |
| 1586 | ENSG00000154856 | ENSG00000183077 |
| 1587 | ENSG00000111276 | ENSG00000238244 |
| 1588 | ENSG00000255038 | ENSG00000197502 |
| 1589 | ENSG00000049759 | ENSG00000165948 |
| 1590 | ENSG00000100146 | ENSG00000101577 |
| 1591 | ENSG00000198909 | ENSG00000127831 |
| 1592 | ENSG00000100604 | ENSG00000008083 |
| 1593 | ENSG00000235750 | ENSG00000169129 |
| 1594 | ENSG00000105323 | ENSG00000163386 |
| 1595 | ENSG00000003756 | ENSG00000232774 |
| 1596 | ENSG00000174502 | ENSG00000160282 |
| 1597 | ENSG00000179088 | ENSG00000133393 |
| 1598 | ENSG00000198858 | ENSG00000149346 |
| 1599 | ENSG00000235718 | ENSG00000011021 |
| 1600 | ENSG00000145780 | ENSG00000182087 |
| 1601 | ENSG00000137267 | ENSG00000145555 |
| 1602 | ENSG00000171150 | ENSG00000144655 |
| 1603 | ENSG00000100842 | ENSG00000186642 |
| 1604 | ENSG00000169016 | ENSG00000160813 |
| 1605 | ENSG00000203879 | ENSG00000143363 |
| 1606 | ENSG00000183971 | ENSG00000244607 |
| 1607 | ENSG00000156510 | ENSG00000005844 |
| 1608 | ENSG00000176200 | ENSG00000100101 |
| 1609 | ENSG00000141971 | ENSG00000160741 |
| 1610 | ENSG00000188389 | ENSG00000075643 |
| 1611 | ENSG00000136603 | ENSG00000182095 |
| 1612 | ENSG00000069943 | ENSG00000167011 |
| 1613 | ENSG00000183569 | ENSG00000164574 |
| 1614 | ENSG00000058262 | ENSG00000123810 |
| 1615 | ENSG00000072274 | ENSG00000196296 |
| 1616 | ENSG00000124587 | ENSG00000179751 |
| 1617 | ENSG00000108175 | ENSG00000099866 |
| 1618 | ENSG00000155324 | ENSG00000145912 |
| 1619 | ENSG00000162231 | ENSG00000168906 |
| 1620 | ENSG00000167700 | ENSG00000161996 |
| 1621 | ENSG00000070366 | ENSG00000119042 |
| 1622 | ENSG00000180909 | ENSG00000204569 |
| 1623 | ENSG00000116698 | ENSG00000241837 |
| 1624 | ENSG00000178149 | ENSG00000128283 |
| 1625 | ENSG00000186792 | ENSG00000156381 |
| 1626 | ENSG00000133401 | ENSG00000131944 |
| 1627 | ENSG00000141279 | ENSG00000144677 |
| 1628 | ENSG00000249590 | ENSG00000161594 |
| 1629 | ENSG00000144560 | ENSG00000167483 |
| 1630 | ENSG00000147526 | ENSG00000119632 |
| 1631 | ENSG00000134508 | ENSG00000088833 |
| 1632 | ENSG00000172530 | ENSG00000179271 |
| 1633 | ENSG00000124201 | ENSG00000160223 |
| 1634 | ENSG00000136205 | ENSG00000153187 |
| 1635 | ENSG00000118515 | ENSG00000140459 |
| 1636 | ENSG00000117594 | ENSG00000121957 |
| 1637 | ENSG00000205129 | ENSG00000221962 |
| 1638 | ENSG00000175471 | ENSG00000160691 |
| 1639 | ENSG00000152705 | ENSG00000268781 |
| 1640 | ENSG00000188452 | ENSG00000100991 |
| 1641 | ENSG00000116701 | ENSG00000146067 |
| 1642 | ENSG00000143190 | ENSG00000196396 |
| 1643 | ENSG00000205809 | ENSG00000157554 |
| 1644 | ENSG00000197557 | ENSG00000111674 |
| 1645 | ENSG00000131188 | ENSG00000163041 |
| 1646 | ENSG00000213983 | ENSG00000174307 |
| 1647 | ENSG00000114853 | ENSG00000133808 |
| 1648 | ENSG00000164825 | ENSG00000213199 |
| 1649 | ENSG00000085831 | ENSG00000185880 |
| 1650 | ENSG00000143401 | ENSG00000228768 |
| 1651 | ENSG00000186318 | ENSG00000157119 |
| 1652 | ENSG00000198818 | ENSG00000067113 |
| 1653 | ENSG00000092820 | ENSG00000141985 |
| 1654 | ENSG00000182993 | ENSG00000180626 |
| 1655 | ENSG00000120658 | ENSG00000076944 |
| 1656 | ENSG00000127946 | ENSG00000089159 |
| 1657 | ENSG00000166716 | ENSG00000131116 |
| 1658 | ENSG00000188343 | ENSG00000172366 |
| 1659 | ENSG00000234719 | ENSG00000119655 |
| 1660 | ENSG00000187730 | ENSG00000110497 |
| 1661 | ENSG00000206053 | ENSG00000142627 |
| 1662 | ENSG00000123584 | ENSG00000099804 |
| 1663 | ENSG00000157570 | ENSG00000120071 |
| 1664 | ENSG00000129277 | ENSG00000124257 |
| 1665 | ENSG00000183160 | ENSG00000172382 |
| 1666 | ENSG00000092054 | ENSG00000001617 |
| 1667 | ENSG00000113068 | ENSG00000139436 |
| 1668 | ENSG00000156414 | ENSG00000147439 |
| 1669 | ENSG00000108270 | ENSG00000268898 |
| 1670 | ENSG00000125648 | ENSG00000125691 |
| 1671 | ENSG00000106537 | ENSG00000110887 |
| 1672 | ENSG00000125798 | ENSG00000172936 |
| 1673 | ENSG00000171124 | ENSG00000060642 |
| 1674 | ENSG00000125841 | ENSG00000133477 |
| 1675 | ENSG00000109917 | ENSG00000067221 |
| 1676 | ENSG00000164587 | ENSG00000141068 |
| 1677 | ENSG00000168234 | ENSG00000185122 |
| 1678 | ENSG00000162520 | ENSG00000133048 |
| 1679 | ENSG00000162460 | ENSG00000178150 |
| 1680 | ENSG00000101384 | ENSG00000113525 |
| 1681 | ENSG00000248592 | ENSG00000243660 |
| 1682 | ENSG00000213927 | ENSG00000170271 |
| 1683 | ENSG00000173673 | ENSG00000177830 |
| 1684 | ENSG00000120833 | ENSG00000050344 |
| 1685 | ENSG00000236311 | ENSG00000124772 |
| 1686 | ENSG00000173926 | ENSG00000185101 |
| 1687 | ENSG00000117115 | ENSG00000183250 |
| 1688 | ENSG00000197430 | ENSG00000131061 |
| 1689 | ENSG00000162542 | ENSG00000175489 |
| 1690 | ENSG00000152689 | ENSG00000070526 |
| 1691 | ENSG00000182217 | ENSG00000143845 |
| 1692 | ENSG00000130518 | ENSG00000138606 |
| 1693 | ENSG00000132702 | ENSG00000189339 |
| 1694 | ENSG00000174628 | ENSG00000154102 |
| 1695 | ENSG00000090013 | ENSG00000100030 |
| 1696 | ENSG00000168010 | ENSG00000182185 |
| 1697 | ENSG00000167173 | ENSG00000241322 |
| 1698 | ENSG00000139865 | ENSG00000213689 |
| 1699 | ENSG00000158402 | ENSG00000137216 |
| 1700 | ENSG00000140374 | ENSG00000105519 |
| 1701 | ENSG00000163352 | ENSG00000160256 |
| 1702 | ENSG00000184984 | ENSG00000136754 |
| 1703 | ENSG00000137693 | ENSG00000176108 |
| 1704 | ENSG00000172409 | ENSG00000101246 |
| 1705 | ENSG00000187024 | ENSG00000169562 |
| 1706 | ENSG00000114113 | ENSG00000182827 |
| 1707 | ENSG00000185842 | ENSG00000072864 |
| 1708 | ENSG00000157191 | ENSG00000077147 |
| 1709 | ENSG00000095139 | ENSG00000112249 |
| 1710 | ENSG00000039600 | ENSG00000154328 |
| 1711 | ENSG00000168066 | ENSG00000099994 |
| 1712 | ENSG00000156222 | ENSG00000263136 |
| 1713 | ENSG00000089094 | ENSG00000197846 |
| 1714 | ENSG00000069812 | ENSG00000141682 |
| 1715 | ENSG00000148377 | ENSG00000149357 |
| 1716 | ENSG00000072954 | ENSG00000198435 |
| 1717 | ENSG00000249115 | ENSG00000110921 |
| 1718 | ENSG00000136068 | ENSG00000177971 |
| 1719 | ENSG00000180061 | ENSG00000097007 |
| 1720 | ENSG00000165714 | ENSG00000183337 |
| 1721 | ENSG00000167964 | ENSG00000032444 |
| 1722 | ENSG00000163683 | ENSG00000178078 |
| 1723 | ENSG00000196950 | ENSG00000163645 |
| 1724 | ENSG00000100852 | ENSG00000132329 |
| 1725 | ENSG00000136108 | ENSG00000105613 |
| 1726 | ENSG00000205476 | ENSG00000095319 |
| 1727 | ENSG00000064201 | ENSG00000143162 |
| 1728 | ENSG00000160218 | ENSG00000075043 |
| 1729 | ENSG00000105341 | ENSG00000197768 |
| 1730 | ENSG00000135596 | ENSG00000137731 |
| 1731 | ENSG00000149548 | ENSG00000124780 |
| 1732 | ENSG00000119812 | ENSG00000160284 |
| 1733 | ENSG00000124215 | ENSG00000130203 |
| 1734 | ENSG00000100426 | ENSG00000168538 |
| 1735 | ENSG00000149781 | ENSG00000011347 |
| 1736 | ENSG00000147889 | ENSG00000166145 |
| 1737 | ENSG00000179965 | ENSG00000087116 |
| 1738 | ENSG00000188878 | ENSG00000004939 |
| 1739 | ENSG00000026025 | ENSG00000100359 |
| 1740 | ENSG00000112304 | ENSG00000112782 |
| 1741 | ENSG00000183011 | ENSG00000123444 |
| 1742 | ENSG00000142224 | ENSG00000105492 |
| 1743 | ENSG00000078237 | ENSG00000065000 |
| 1744 | ENSG00000213648 | ENSG00000131044 |
| 1745 | ENSG00000269103 | ENSG00000142178 |
| 1746 | ENSG00000184502 | ENSG00000148341 |
| 1747 | ENSG00000146122 | ENSG00000243509 |
| 1748 | ENSG00000183682 | ENSG00000096395 |
| 1749 | ENSG00000132823 | ENSG00000170807 |
| 1750 | ENSG00000162975 | ENSG00000011275 |
| 1751 | ENSG00000119917 | ENSG00000258984 |
| 1752 | ENSG00000138175 | ENSG00000130703 |
| 1753 | ENSG00000183747 | ENSG00000143418 |
| 1754 | ENSG00000183706 | ENSG00000269048 |
| 1755 | ENSG00000163435 | ENSG00000139053 |
| 1756 | ENSG00000132207 | ENSG00000067191 |
| 1757 | ENSG00000197616 | ENSG00000120068 |
| 1758 | ENSG00000148848 | ENSG00000163701 |
| 1759 | ENSG00000108405 | ENSG00000183638 |
| 1760 | ENSG00000157045 | ENSG00000173805 |
| 1761 | ENSG00000119950 | ENSG00000108639 |
| 1762 | ENSG00000170788 | ENSG00000157782 |
| 1763 | ENSG00000100994 | ENSG00000153179 |
| 1764 | ENSG00000167625 | ENSG00000075651 |
| 1765 | ENSG00000065882 | ENSG00000178209 |
| 1766 | ENSG00000143420 | ENSG00000170310 |
| 1767 | ENSG00000215014 | ENSG00000132740 |
| 1768 | ENSG00000104964 | ENSG00000243989 |
| 1769 | ENSG00000189308 | ENSG00000188428 |
| 1770 | ENSG00000186480 | ENSG00000196235 |
| 1771 | ENSG00000171136 | ENSG00000146112 |
| 1772 | ENSG00000160226 | ENSG00000162641 |
| 1773 | ENSG00000167208 | ENSG00000164713 |
| 1774 | ENSG00000134198 | ENSG00000171914 |
| 1775 | ENSG00000118707 | ENSG00000196754 |
| 1776 | ENSG00000143384 | ENSG00000090020 |
| 1777 | ENSG00000130638 | ENSG00000187997 |
| 1778 | ENSG00000141977 | ENSG00000138495 |
| 1779 | ENSG00000151150 | ENSG00000166359 |
| 1780 | ENSG00000116473 | ENSG00000039068 |
| 1781 | ENSG00000125398 | ENSG00000140479 |
| 1782 | ENSG00000068120 | ENSG00000263155 |
| 1783 | ENSG00000151376 | ENSG00000181350 |
| 1784 | ENSG00000083454 | ENSG00000148175 |
| 1785 | ENSG00000241360 | ENSG00000257767 |
| 1786 | ENSG00000128482 | ENSG00000167280 |
| 1787 | ENSG00000188396 | ENSG00000006071 |
| 1788 | ENSG00000110768 | ENSG00000131981 |
| 1789 | ENSG00000197459 | ENSG00000173281 |
| 1790 | ENSG00000066027 | ENSG00000137817 |
| 1791 | ENSG00000100335 | ENSG00000150201 |
| 1792 | ENSG00000149212 | ENSG00000103591 |
| 1793 | ENSG00000196730 | ENSG00000122711 |
| 1794 | ENSG00000100156 | ENSG00000154678 |
| 1795 | ENSG00000135709 | ENSG00000266964 |
| 1796 | ENSG00000118432 | ENSG00000178741 |
| 1797 | ENSG00000173418 | ENSG00000149177 |
| 1798 | ENSG00000161270 | ENSG00000178852 |
| 1799 | ENSG00000094804 | ENSG00000168461 |
| 1800 | ENSG00000196226 | ENSG00000173264 |
| 1801 | ENSG00000168615 | ENSG00000083444 |
| 1802 | ENSG00000137809 | ENSG00000259471 |
| 1803 | ENSG00000127666 | ENSG00000061455 |
| 1804 | ENSG00000118292 | ENSG00000099282 |
| 1805 | ENSG00000188505 | ENSG00000140993 |
| 1806 | ENSG00000139318 | ENSG00000175564 |
| 1807 | ENSG00000156011 | ENSG00000134775 |
| 1808 | ENSG00000136933 | ENSG00000134668 |
| 1809 | ENSG00000161642 | ENSG00000121764 |
| 1810 | ENSG00000196935 | ENSG00000143553 |
| 1811 | ENSG00000122218 | ENSG00000171408 |
| 1812 | ENSG00000174521 | ENSG00000122694 |
| 1813 | ENSG00000163359 | ENSG00000008735 |
| 1814 | ENSG00000089154 | ENSG00000197561 |
| 1815 | ENSG00000255439 | ENSG00000182264 |
| 1816 | ENSG00000226321 | ENSG00000100316 |
| 1817 | ENSG00000165660 | ENSG00000158055 |
| 1818 | ENSG00000179284 | ENSG00000171475 |
| 1819 | ENSG00000007047 | ENSG00000198700 |
| 1820 | ENSG00000121060 | ENSG00000266714 |
| 1821 | ENSG00000124610 | ENSG00000155744 |
| 1822 | ENSG00000141540 | ENSG00000221845 |
| 1823 | ENSG00000180902 | ENSG00000198807 |
| 1824 | ENSG00000160593 | ENSG00000189377 |
| 1825 | ENSG00000247121 | ENSG00000175646 |
| 1826 | ENSG00000182352 | ENSG00000187021 |
| 1827 | ENSG00000130164 | ENSG00000149115 |
| 1828 | ENSG00000180596 | ENSG00000099194 |
| 1829 | ENSG00000095370 | ENSG00000141258 |
| 1830 | ENSG00000197601 | ENSG00000205364 |
| 1831 | ENSG00000090104 | ENSG00000140718 |
| 1832 | ENSG00000075624 | ENSG00000130876 |
| 1833 | ENSG00000034152 | ENSG00000168071 |
| 1834 | ENSG00000168026 | ENSG00000132182 |
| 1835 | ENSG00000152784 | ENSG00000182326 |
| 1836 | ENSG00000188827 | ENSG00000160613 |
| 1837 | ENSG00000154358 | ENSG00000158445 |
| 1838 | ENSG00000031003 | ENSG00000168631 |
| 1839 | ENSG00000082512 | ENSG00000166086 |
| 1840 | ENSG00000121579 | ENSG00000176595 |
| 1841 | ENSG00000099992 | ENSG00000179134 |
| 1842 | ENSG00000157870 | ENSG00000149635 |
| 1843 | ENSG00000135365 | ENSG00000172348 |
| 1844 | ENSG00000167716 | ENSG00000181038 |
| 1845 | ENSG00000174339 | ENSG00000124256 |
| 1846 | ENSG00000128342 | ENSG00000070501 |
| 1847 | ENSG00000261949 | ENSG00000105325 |
| 1848 | ENSG00000117625 | ENSG00000177679 |
| 1849 | ENSG00000198796 | ENSG00000140577 |
| 1850 | ENSG00000171316 | ENSG00000173991 |
| 1851 | ENSG00000182871 | ENSG00000157613 |
| 1852 | ENSG00000178363 | ENSG00000185963 |
| 1853 | ENSG00000167323 | ENSG00000204257 |
| 1854 | ENSG00000226979 | ENSG00000257108 |
| 1855 | ENSG00000131969 | ENSG00000145794 |
| 1856 | ENSG00000081791 | ENSG00000148288 |
| 1857 | ENSG00000113648 | ENSG00000149136 |
| 1858 | ENSG00000142459 | ENSG00000188112 |
| 1859 | ENSG00000077157 | ENSG00000120093 |
| 1860 | ENSG00000217075 | ENSG00000184471 |
| 1861 | ENSG00000258728 | ENSG00000177427 |
| 1862 | ENSG00000183463 | ENSG00000074047 |
| 1863 | ENSG00000100209 | ENSG00000184313 |
| 1864 | ENSG00000080031 | ENSG00000107902 |
| 1865 | ENSG00000138756 | ENSG00000087258 |
| 1866 | ENSG00000196465 | ENSG00000250733 |
| 1867 | ENSG00000121753 | ENSG00000068724 |
| 1868 | ENSG00000173068 | ENSG00000077420 |
| 1869 | ENSG00000165795 | ENSG00000148541 |
| 1870 | ENSG00000268553 | ENSG00000103257 |
| 1871 | ENSG00000240583 | ENSG00000036549 |
| 1872 | ENSG00000012124 | ENSG00000065534 |
| 1873 | ENSG00000181409 | ENSG00000010072 |
| 1874 | ENSG00000106330 | ENSG00000172938 |
| 1875 | ENSG00000102471 | ENSG00000077809 |
| 1876 | ENSG00000149480 | ENSG00000157593 |
| 1877 | ENSG00000134490 | ENSG00000215790 |
| 1878 | ENSG00000138796 | ENSG00000182492 |
| 1879 | ENSG00000186115 | ENSG00000108511 |
| 1880 | ENSG00000159231 | ENSG00000035664 |
| 1881 | ENSG00000269449 | ENSG00000152377 |
| 1882 | ENSG00000166592 | ENSG00000077152 |
| 1883 | ENSG00000133466 | ENSG00000091622 |
| 1884 | ENSG00000111846 | ENSG00000156709 |
| 1885 | ENSG00000101417 | ENSG00000092964 |
| 1886 | ENSG00000167363 | ENSG00000167580 |
| 1887 | ENSG00000100439 | ENSG00000267645 |
| 1888 | ENSG00000131055 | ENSG00000174038 |
| 1889 | ENSG00000167549 | ENSG00000197757 |
| 1890 | ENSG00000260903 | ENSG00000146083 |
| 1891 | ENSG00000230510 | ENSG00000141401 |
| 1892 | ENSG00000087448 | ENSG00000126266 |
| 1893 | ENSG00000121774 | ENSG00000254788 |
| 1894 | ENSG00000166897 | ENSG00000237847 |
| 1895 | ENSG00000132514 | ENSG00000167566 |
| 1896 | ENSG00000166246 | ENSG00000134326 |
| 1897 | ENSG00000067606 | ENSG00000080618 |
| 1898 | ENSG00000173269 | ENSG00000100288 |
| 1899 | ENSG00000143479 | ENSG00000183570 |
| 1900 | ENSG00000097033 | ENSG00000123908 |
| 1901 | ENSG00000231880 | ENSG00000111199 |
| 1902 | ENSG00000113328 | ENSG00000174804 |
| 1903 | ENSG00000172640 | ENSG00000186635 |
| 1904 | ENSG00000181830 | ENSG00000186994 |
| 1905 | ENSG00000136425 | ENSG00000167770 |
| 1906 | ENSG00000178467 | ENSG00000141026 |
| 1907 | ENSG00000166984 | ENSG00000104221 |
| 1908 | ENSG00000099625 | ENSG00000005156 |
| 1909 | ENSG00000144057 | ENSG00000161960 |
| 1910 | ENSG00000149016 | ENSG00000168101 |
| 1911 | ENSG00000119865 | ENSG00000166839 |
| 1912 | ENSG00000079459 | ENSG00000119801 |
| 1913 | ENSG00000170889 | ENSG00000137962 |
| 1914 | ENSG00000178404 | ENSG00000178252 |
| 1915 | ENSG00000144597 | ENSG00000144579 |
| 1916 | ENSG00000119408 | ENSG00000175318 |
| 1917 | ENSG00000167601 | ENSG00000198517 |
| 1918 | ENSG00000170677 | ENSG00000027697 |
| 1919 | ENSG00000149269 | ENSG00000177721 |
| 1920 | ENSG00000267618 | ENSG00000134333 |
| 1921 | ENSG00000167123 | ENSG00000137106 |
| 1922 | ENSG00000162073 | ENSG00000172828 |
| 1923 | ENSG00000185875 | ENSG00000267913 |
| 1924 | ENSG00000149043 | ENSG00000110448 |
| 1925 | ENSG00000146216 | ENSG00000152700 |
| 1926 | ENSG00000043143 | ENSG00000091136 |
| 1927 | ENSG00000047644 | ENSG00000139505 |
| 1928 | ENSG00000228008 | ENSG00000110455 |
| 1929 | ENSG00000159184 | ENSG00000257949 |
| 1930 | ENSG00000148688 | ENSG00000106211 |
| 1931 | ENSG00000262039 | ENSG00000262246 |
| 1932 | ENSG00000183111 | ENSG00000175911 |
| 1933 | ENSG00000178607 | ENSG00000149948 |
| 1934 | ENSG00000122958 | ENSG00000090975 |
| 1935 | ENSG00000179058 | ENSG00000134109 |
| 1936 | ENSG00000150722 | ENSG00000149532 |
| 1937 | ENSG00000196588 | ENSG00000198569 |
| 1938 | ENSG00000186567 | ENSG00000248993 |
| 1939 | ENSG00000002330 | ENSG00000156931 |
| 1940 | ENSG00000180828 | ENSG00000267848 |
| 1941 | ENSG00000067955 | ENSG00000136450 |
| 1942 | ENSG00000185271 | ENSG00000142599 |
| 1943 | ENSG00000124813 | ENSG00000157103 |
| 1944 | ENSG00000251503 | ENSG00000167618 |
| 1945 | ENSG00000198246 | ENSG00000167103 |
| 1946 | ENSG00000172403 | ENSG00000010278 |
| 1947 | ENSG00000198074 | ENSG00000181381 |
| 1948 | ENSG00000197364 | ENSG00000104808 |
| 1949 | ENSG00000154124 | ENSG00000172116 |
| 1950 | ENSG00000015133 | ENSG00000066294 |
| 1951 | ENSG00000167085 | ENSG00000030419 |
| 1952 | ENSG00000158008 | ENSG00000258512 |
| 1953 | ENSG00000088543 | ENSG00000110777 |
| 1954 | ENSG00000168078 | ENSG00000179604 |
| 1955 | ENSG00000166734 | ENSG00000163814 |
| 1956 | ENSG00000186049 | ENSG00000146809 |
| 1957 | ENSG00000140105 | ENSG00000143321 |
| 1958 | ENSG00000174992 | ENSG00000242114 |
| 1959 | ENSG00000150907 | ENSG00000174945 |
| 1960 | ENSG00000141499 | ENSG00000143995 |
| 1961 | ENSG00000101452 | ENSG00000169813 |
| 1962 | ENSG00000162367 | ENSG00000079308 |
| 1963 | ENSG00000137337 | ENSG00000077238 |
| 1964 | ENSG00000266208 | ENSG00000119013 |
| 1965 | ENSG00000049239 | ENSG00000135775 |
| 1966 | ENSG00000051009 | ENSG00000174705 |
| 1967 | ENSG00000162078 | ENSG00000243678 |
| 1968 | ENSG00000172935 | ENSG00000104824 |
| 1969 | ENSG00000182544 | ENSG00000173207 |
| 1970 | ENSG00000144837 | ENSG00000087008 |
| 1971 | ENSG00000185955 | ENSG00000013810 |
| 1972 | ENSG00000151065 | ENSG00000001461 |
| 1973 | ENSG00000150787 | ENSG00000155508 |
| 1974 | ENSG00000021826 | ENSG00000048828 |
| 1975 | ENSG00000181523 | ENSG00000152234 |
| 1976 | ENSG00000068097 | ENSG00000108622 |
| 1977 | ENSG00000104067 | ENSG00000104228 |
| 1978 | ENSG00000100038 | ENSG00000102119 |
| 1979 | ENSG00000224186 | ENSG00000037042 |
| 1980 | ENSG00000130177 | ENSG00000255168 |
| 1981 | ENSG00000115318 | ENSG00000089053 |
| 1982 | ENSG00000129235 | ENSG00000174514 |
| 1983 | ENSG00000008294 | ENSG00000136238 |
| 1984 | ENSG00000106299 | ENSG00000268397 |
| 1985 | ENSG00000134970 | ENSG00000113719 |
| 1986 | ENSG00000149582 | ENSG00000099860 |
| 1987 | ENSG00000100314 | ENSG00000124766 |
| 1988 | ENSG00000167863 | ENSG00000114745 |
| 1989 | ENSG00000175746 | ENSG00000166025 |
| 1990 | ENSG00000120278 | ENSG00000176438 |
| 1991 | ENSG00000166507 | ENSG00000162244 |
| 1992 | ENSG00000148399 | ENSG00000105255 |
| 1993 | ENSG00000034713 | ENSG00000168209 |
| 1994 | ENSG00000185668 | ENSG00000169241 |
| 1995 | ENSG00000162775 | ENSG00000243156 |
| 1996 | ENSG00000109111 | ENSG00000115661 |
| 1997 | ENSG00000101199 | ENSG00000125522 |
| 1998 | ENSG00000076554 | ENSG00000100023 |
| 1999 | ENSG00000179256 | ENSG00000206527 |
| 2000 | ENSG00000149577 | ENSG00000267942 |
| 2001 | ENSG00000063241 | ENSG00000124440 |
| 2002 | ENSG00000157470 | ENSG00000089486 |
| 2003 | ENSG00000111371 | ENSG00000096093 |
| 2004 | ENSG00000114738 | ENSG00000001460 |
| 2005 | ENSG00000167112 | ENSG00000269422 |
| 2006 | ENSG00000170348 | ENSG00000139370 |
| 2007 | ENSG00000104883 | ENSG00000197956 |
| 2008 | ENSG00000138083 | ENSG00000197728 |
| 2009 | ENSG00000205531 | ENSG00000161217 |
| 2010 | ENSG00000185294 | ENSG00000100029 |
| 2011 | ENSG00000226397 | ENSG00000105357 |
| 2012 | ENSG00000151725 | ENSG00000142798 |
| 2013 | ENSG00000216490 | ENSG00000136938 |
| 2014 | ENSG00000259120 | ENSG00000169515 |
| 2015 | ENSG00000163166 | ENSG00000105810 |
| 2016 | ENSG00000169689 | ENSG00000111913 |
| 2017 | ENSG00000123473 | ENSG00000174775 |
| 2018 | ENSG00000159214 | ENSG00000170613 |
| 2019 | ENSG00000111300 | ENSG00000165269 |
| 2020 | ENSG00000135968 | ENSG00000139793 |
| 2021 | ENSG00000091436 | ENSG00000143412 |
| 2022 | ENSG00000020633 | ENSG00000146856 |
| 2023 | ENSG00000187049 | ENSG00000178719 |
| 2024 | ENSG00000172867 | ENSG00000174990 |
| 2025 | ENSG00000186827 | ENSG00000080293 |
| 2026 | ENSG00000165912 | ENSG00000148985 |
| 2027 | ENSG00000165914 | ENSG00000160678 |
| 2028 | ENSG00000268822 | ENSG00000166963 |
| 2029 | ENSG00000106991 | ENSG00000142039 |
| 2030 | ENSG00000125810 | ENSG00000006704 |
| 2031 | ENSG00000129514 | ENSG00000176533 |
| 2032 | ENSG00000163295 | ENSG00000171105 |
| 2033 | ENSG00000110090 | ENSG00000121577 |
| 2034 | ENSG00000225190 | ENSG00000118257 |
| 2035 | ENSG00000130382 | ENSG00000266086 |
| 2036 | ENSG00000269169 | ENSG00000183597 |
| 2037 | ENSG00000006530 | ENSG00000131094 |
| 2038 | ENSG00000142273 | ENSG00000164053 |
| 2039 | ENSG00000231672 | ENSG00000178502 |
| 2040 | ENSG00000182054 | ENSG00000129933 |
| 2041 | ENSG00000126467 | ENSG00000136448 |
| 2042 | ENSG00000125520 | ENSG00000174437 |
| 2043 | ENSG00000128965 | ENSG00000168260 |
| 2044 | ENSG00000166272 | ENSG00000110063 |
| 2045 | ENSG00000258366 | ENSG00000077348 |
| 2046 | ENSG00000176749 | ENSG00000105696 |
| 2047 | ENSG00000017621 | ENSG00000131069 |
| 2048 | ENSG00000152217 | ENSG00000184860 |
| 2049 | ENSG00000177150 | ENSG00000126368 |
| 2050 | ENSG00000100626 | ENSG00000196415 |
| 2051 | ENSG00000204882 | ENSG00000143621 |
| 2052 | ENSG00000155034 | ENSG00000185928 |
| 2053 | ENSG00000172742 | ENSG00000183246 |
| 2054 | ENSG00000187145 | ENSG00000269035 |
| 2055 | ENSG00000118965 | ENSG00000167178 |
| 2056 | ENSG00000112578 | ENSG00000172869 |
| 2057 | ENSG00000125551 | ENSG00000173706 |
| 2058 | ENSG00000174567 | ENSG00000180573 |
| 2059 | ENSG00000141510 | ENSG00000107672 |
| 2060 | ENSG00000175482 | ENSG00000178980 |
| 2061 | ENSG00000102103 | ENSG00000124225 |
| 2062 | ENSG00000100345 | ENSG00000145029 |
| 2063 | ENSG00000134201 | ENSG00000141127 |
| 2064 | ENSG00000177082 | ENSG00000100097 |
| 2065 | ENSG00000159713 | ENSG00000143110 |
| 2066 | ENSG00000137574 | ENSG00000137413 |
| 2067 | ENSG00000156735 | ENSG00000167670 |
| 2068 | ENSG00000130702 | ENSG00000129657 |
| 2069 | ENSG00000107960 | ENSG00000198742 |
| 2070 | ENSG00000171227 | ENSG00000152952 |
| 2071 | ENSG00000164620 | ENSG00000100342 |
| 2072 | ENSG00000170866 | ENSG00000104885 |
| 2073 | ENSG00000109099 | ENSG00000157510 |
| 2074 | ENSG00000149294 | ENSG00000197457 |
| 2075 | ENSG00000140992 | ENSG00000132466 |
| 2076 | ENSG00000008838 | ENSG00000142619 |
| 2077 | ENSG00000163464 | ENSG00000051108 |
| 2078 | ENSG00000140521 | ENSG00000107551 |
| 2079 | ENSG00000204592 | ENSG00000138119 |
| 2080 | ENSG00000105856 | ENSG00000144136 |
| 2081 | ENSG00000081320 | ENSG00000134318 |
| 2082 | ENSG00000063015 | ENSG00000103415 |
| 2083 | ENSG00000198719 | ENSG00000090924 |
| 2084 | ENSG00000006042 | ENSG00000076864 |
| 2085 | ENSG00000184216 | ENSG00000090061 |
| 2086 | ENSG00000244734 | ENSG00000069956 |
| 2087 | ENSG00000243667 | ENSG00000187942 |
| 2088 | ENSG00000187624 | ENSG00000196405 |
| 2089 | ENSG00000196323 | ENSG00000250067 |
| 2090 | ENSG00000120875 | ENSG00000179029 |
| 2091 | ENSG00000228474 | ENSG00000136167 |
| 2092 | ENSG00000110104 | ENSG00000197747 |
| 2093 | ENSG00000156042 | ENSG00000105063 |
| 2094 | ENSG00000159625 | ENSG00000205334 |
| 2095 | ENSG00000154310 | ENSG00000164125 |
| 2096 | ENSG00000196532 | ENSG00000084234 |
| 2097 | ENSG00000104129 | ENSG00000172548 |
| 2098 | ENSG00000128228 | ENSG00000157778 |
| 2099 | ENSG00000184454 | ENSG00000167771 |
| 2100 | ENSG00000178127 | ENSG00000089327 |
| 2101 | ENSG00000107937 | ENSG00000159648 |
| 2102 | ENSG00000183955 | ENSG00000196420 |
| 2103 | ENSG00000234949 | ENSG00000117877 |
| 2104 | ENSG00000183431 | ENSG00000121964 |
| 2105 | ENSG00000134627 | ENSG00000164548 |
| 2106 | ENSG00000150991 | ENSG00000168484 |
| 2107 | ENSG00000135870 | ENSG00000105127 |
| 2108 | ENSG00000130402 | ENSG00000107263 |
| 2109 | ENSG00000119900 | ENSG00000197321 |
| 2110 | ENSG00000183684 | ENSG00000136960 |
| 2111 | ENSG00000182108 | ENSG00000185666 |
| 2112 | ENSG00000268509 | ENSG00000138629 |
| 2113 | ENSG00000268991 | ENSG00000168491 |
| 2114 | ENSG00000134183 | ENSG00000187678 |
| 2115 | ENSG00000164398 | ENSG00000103423 |
| 2116 | ENSG00000167721 | ENSG00000123159 |
| 2117 | ENSG00000081760 | ENSG00000106628 |
| 2118 | ENSG00000111540 | ENSG00000130173 |
| 2119 | ENSG00000172915 | ENSG00000135093 |
| 2120 | ENSG00000071677 | ENSG00000172236 |
| 2121 | ENSG00000169994 | ENSG00000174498 |
| 2122 | ENSG00000134463 | ENSG00000152223 |
| 2123 | ENSG00000204498 | ENSG00000006453 |
| 2124 | ENSG00000139192 | ENSG00000112308 |
| 2125 | ENSG00000184774 | ENSG00000044446 |
| 2126 | ENSG00000158555 | ENSG00000141570 |
| 2127 | ENSG00000129951 | ENSG00000089012 |
| 2128 | ENSG00000196565 | ENSG00000257411 |
| 2129 | ENSG00000214655 | ENSG00000006432 |
| 2130 | ENSG00000167641 | ENSG00000135862 |
| 2131 | ENSG00000114030 | ENSG00000113070 |
| 2132 | ENSG00000248167 | ENSG00000117318 |
| 2133 | ENSG00000167646 | ENSG00000135414 |
| 2134 | ENSG00000134028 | ENSG00000008382 |
| 2135 | ENSG00000182810 | ENSG00000100092 |
| 2136 | ENSG00000086506 | ENSG00000010818 |
| 2137 | ENSG00000120802 | ENSG00000119938 |
| 2138 | ENSG00000087903 | ENSG00000257921 |
| 2139 | ENSG00000033050 | ENSG00000095713 |
| 2140 | ENSG00000066629 | ENSG00000110074 |
| 2141 | ENSG00000168070 | ENSG00000214212 |
| 2142 | ENSG00000182866 | ENSG00000108469 |
| 2143 | ENSG00000119723 | ENSG00000160588 |
| 2144 | ENSG00000124783 | ENSG00000069275 |
| 2145 | ENSG00000133059 | ENSG00000122547 |
| 2146 | ENSG00000040633 | ENSG00000172572 |
| 2147 | ENSG00000114850 | ENSG00000177156 |
| 2148 | ENSG00000183751 | ENSG00000058085 |
| 2149 | ENSG00000061938 | ENSG00000141002 |
| 2150 | ENSG00000204957 | ENSG00000197122 |
| 2151 | ENSG00000147570 | ENSG00000170689 |
| 2152 | ENSG00000167978 | ENSG00000164708 |
| 2153 | ENSG00000179627 | ENSG00000183773 |
| 2154 | ENSG00000253426 | ENSG00000154035 |
| 2155 | ENSG00000100354 | ENSG00000128564 |
| 2156 | ENSG00000109991 | ENSG00000123297 |
| 2157 | ENSG00000153789 | ENSG00000255994 |
| 2158 | ENSG00000205177 | ENSG00000128915 |
| 2159 | ENSG00000255073 | ENSG00000103241 |
| 2160 | ENSG00000167291 | ENSG00000179144 |
| 2161 | ENSG00000168118 | ENSG00000230493 |
| 2162 | ENSG00000162174 | ENSG00000177380 |
| 2163 | ENSG00000198585 | ENSG00000188191 |
| 2164 | ENSG00000187097 | ENSG00000239900 |
| 2165 | ENSG00000267740 | ENSG00000187688 |
| 2166 | ENSG00000175567 | ENSG00000103091 |
| 2167 | ENSG00000134574 | ENSG00000157985 |
| 2168 | ENSG00000168152 | ENSG00000124074 |
| 2169 | ENSG00000187905 | ENSG00000166825 |
| 2170 | ENSG00000141905 | ENSG00000108556 |
| 2171 | ENSG00000162664 | ENSG00000163349 |
| 2172 | ENSG00000245888 | ENSG00000067182 |
| 2173 | ENSG00000140995 | ENSG00000061273 |
| 2174 | ENSG00000116014 | ENSG00000095932 |
| 2175 | ENSG00000167515 | ENSG00000197562 |
| 2176 | ENSG00000126778 | ENSG00000102572 |
| 2177 | ENSG00000268965 | ENSG00000139641 |
| 2178 | ENSG00000182986 | ENSG00000143772 |
| 2179 | ENSG00000141858 | ENSG00000089847 |
| 2180 | ENSG00000196072 | ENSG00000186185 |
| 2181 | ENSG00000213057 | ENSG00000101343 |
| 2182 | ENSG00000180264 | ENSG00000142621 |
| 2183 | ENSG00000108950 | ENSG00000185104 |
| 2184 | ENSG00000123374 | ENSG00000115919 |
| 2185 | ENSG00000259753 | ENSG00000196083 |
| 2186 | ENSG00000197380 | ENSG00000106125 |
| 2187 | ENSG00000126091 | ENSG00000160255 |
| 2188 | ENSG00000221886 | ENSG00000213471 |
| 2189 | ENSG00000168159 | ENSG00000166484 |
| 2190 | ENSG00000180383 | ENSG00000196866 |
| 2191 | ENSG00000268403 | ENSG00000198373 |
| 2192 | ENSG00000213085 | ENSG00000204377 |
| 2193 | ENSG00000179632 | ENSG00000239605 |
| 2194 | ENSG00000037897 | ENSG00000133392 |
| 2195 | ENSG00000167604 | ENSG00000137203 |
| 2196 | ENSG00000162545 | ENSG00000255394 |
| 2197 | ENSG00000174903 | ENSG00000107447 |
| 2198 | ENSG00000149654 | ENSG00000087460 |
| 2199 | ENSG00000188686 | ENSG00000174898 |
| 2200 | ENSG00000181577 | ENSG00000255730 |
| 2201 | ENSG00000119682 | ENSG00000100650 |
| 2202 | ENSG00000182667 | ENSG00000158195 |
| 2203 | ENSG00000269375 | ENSG00000100483 |
| 2204 | ENSG00000156411 | ENSG00000213402 |
| 2205 | ENSG00000065325 | ENSG00000135336 |
| 2206 | ENSG00000105723 | ENSG00000163932 |
| 2207 | ENSG00000146904 | ENSG00000143578 |
| 2208 | ENSG00000205436 | ENSG00000185236 |
| 2209 | ENSG00000162924 | ENSG00000087586 |
| 2210 | ENSG00000173917 | ENSG00000215012 |
| 2211 | ENSG00000142330 | ENSG00000168404 |
| 2212 | ENSG00000135297 | ENSG00000143183 |
| 2213 | ENSG00000126858 | ENSG00000153815 |
| 2214 | ENSG00000088367 | ENSG00000134369 |
| 2215 | ENSG00000085721 | ENSG00000175206 |
| 2216 | ENSG00000165502 | ENSG00000088726 |
| 2217 | ENSG00000213654 | ENSG00000177548 |
| 2218 | ENSG00000076604 | ENSG00000146872 |
| 2219 | ENSG00000203864 | ENSG00000127948 |
| 2220 | ENSG00000198937 | ENSG00000213578 |
| 2221 | ENSG00000181449 | ENSG00000269766 |
| 2222 | ENSG00000258292 | ENSG00000082641 |
| 2223 | ENSG00000204511 | ENSG00000184634 |
| 2224 | ENSG00000172270 | ENSG00000175193 |
| 2225 | ENSG00000120094 | ENSG00000167491 |
| 2226 | ENSG00000110717 | ENSG00000106367 |
| 2227 | ENSG00000101150 | ENSG00000258572 |
| 2228 | ENSG00000173614 | ENSG00000141867 |
| 2229 | ENSG00000272762 | ENSG00000166796 |
| 2230 | ENSG00000138135 | ENSG00000197256 |
| 2231 | ENSG00000128872 | ENSG00000151715 |
| 2232 | ENSG00000154330 | ENSG00000253368 |
| 2233 | ENSG00000071051 | ENSG00000205213 |
| 2234 | ENSG00000198520 | ENSG00000142910 |
| 2235 | ENSG00000173728 | ENSG00000059588 |
| 2236 | ENSG00000185000 | ENSG00000115648 |
| 2237 | ENSG00000170776 | ENSG00000167371 |
| 2238 | ENSG00000004897 | ENSG00000139722 |
| 2239 | ENSG00000213380 | ENSG00000168556 |
| 2240 | ENSG00000025800 | ENSG00000184574 |
| 2241 | ENSG00000179066 | ENSG00000159723 |
| 2242 | ENSG00000128652 | ENSG00000189306 |
| 2243 | ENSG00000165609 | ENSG00000137713 |
| 2244 | ENSG00000184599 | ENSG00000077984 |
| 2245 | ENSG00000129214 | ENSG00000188163 |
| 2246 | ENSG00000118094 | ENSG00000187902 |
| 2247 | ENSG00000198690 | ENSG00000133318 |
| 2248 | ENSG00000135404 | ENSG00000197119 |
| 2249 | ENSG00000141873 | ENSG00000144589 |
| 2250 | ENSG00000147592 | ENSG00000114857 |
| 2251 | ENSG00000142319 | ENSG00000101216 |
| 2252 | ENSG00000173846 | ENSG00000244020 |
| 2253 | ENSG00000074370 | ENSG00000253320 |
| 2254 | ENSG00000069248 | ENSG00000108352 |
| 2255 | ENSG00000120063 | ENSG00000166548 |
| 2256 | ENSG00000250588 | ENSG00000178860 |
| 2257 | ENSG00000158683 | ENSG00000228532 |
| 2258 | ENSG00000156599 | ENSG00000122180 |
| 2259 | ENSG00000182551 | ENSG00000186684 |
| 2260 | ENSG00000162992 | ENSG00000142677 |
| 2261 | ENSG00000173727 | ENSG00000184545 |
| 2262 | ENSG00000132326 | ENSG00000111961 |
| 2263 | ENSG00000118971 | ENSG00000157423 |
| 2264 | ENSG00000148843 | ENSG00000111405 |
| 2265 | ENSG00000021762 | ENSG00000049860 |
| 2266 | ENSG00000073536 | ENSG00000100968 |
| 2267 | ENSG00000175115 | ENSG00000008853 |
| 2268 | ENSG00000205057 | ENSG00000156925 |
| 2269 | ENSG00000135686 | ENSG00000126561 |
| 2270 | ENSG00000221946 | ENSG00000205593 |
| 2271 | ENSG00000255537 | ENSG00000075914 |
| 2272 | ENSG00000092841 | ENSG00000152767 |
| 2273 | ENSG00000087152 | ENSG00000108244 |
| 2274 | ENSG00000137802 | ENSG00000162407 |
| 2275 | ENSG00000183304 | ENSG00000177200 |
| 2276 | ENSG00000162571 | ENSG00000186198 |
| 2277 | ENSG00000183979 | ENSG00000177800 |
| 2278 | ENSG00000241978 | ENSG00000077044 |
| 2279 | ENSG00000257093 | ENSG00000232112 |
| 2280 | ENSG00000167741 | ENSG00000113739 |
| 2281 | ENSG00000023892 | ENSG00000174529 |
| 2282 | ENSG00000122299 | ENSG00000065600 |
| 2283 | ENSG00000160959 | ENSG00000122304 |
| 2284 | ENSG00000158163 | ENSG00000167635 |
| 2285 | ENSG00000169020 | ENSG00000184381 |
| 2286 | ENSG00000174886 | ENSG00000096384 |
| 2287 | ENSG00000126391 | ENSG00000160145 |
| 2288 | ENSG00000258311 | ENSG00000196700 |
| 2289 | ENSG00000172725 | ENSG00000187775 |
| 2290 | ENSG00000104164 | ENSG00000269074 |
| 2291 | ENSG00000013306 | ENSG00000163958 |
| 2292 | ENSG00000149506 | ENSG00000107077 |
| 2293 | ENSG00000166886 | ENSG00000225683 |
| 2294 | ENSG00000143320 | ENSG00000116138 |
| 2295 | ENSG00000133104 | ENSG00000140416 |
| 2296 | ENSG00000171766 | ENSG00000100403 |
| 2297 | ENSG00000196388 | ENSG00000111641 |
| 2298 | ENSG00000130706 | ENSG00000215695 |
| 2299 | ENSG00000052126 | ENSG00000171435 |
| 2300 | ENSG00000101350 | ENSG00000011422 |
| 2301 | ENSG00000233493 | ENSG00000139835 |
| 2302 | ENSG00000204152 | ENSG00000198917 |
| 2303 | ENSG00000101493 | ENSG00000068903 |
| 2304 | ENSG00000182698 | ENSG00000122515 |
| 2305 | ENSG00000110244 | ENSG00000197724 |
| 2306 | ENSG00000162949 | ENSG00000138623 |
| 2307 | ENSG00000110076 | ENSG00000100600 |
| 2308 | ENSG00000114353 | ENSG00000133250 |
| 2309 | ENSG00000141560 | ENSG00000165672 |
| 2310 | ENSG00000173431 | ENSG00000160219 |
| 2311 | ENSG00000146859 | ENSG00000233436 |
| 2312 | ENSG00000187391 | ENSG00000205978 |
| 2313 | ENSG00000186723 | ENSG00000196208 |
| 2314 | ENSG00000166377 | ENSG00000234438 |
| 2315 | ENSG00000196355 | ENSG00000171773 |
| 2316 | ENSG00000182584 | ENSG00000013364 |
| 2317 | ENSG00000269605 | ENSG00000168264 |
| 2318 | ENSG00000145901 | ENSG00000182177 |
| 2319 | ENSG00000129159 | ENSG00000183255 |
| 2320 | ENSG00000070019 | ENSG00000133026 |
| 2321 | ENSG00000117479 | ENSG00000010810 |
| 2322 | ENSG00000184203 | ENSG00000042062 |
| 2323 | ENSG00000170955 | ENSG00000163660 |
| 2324 | ENSG00000188895 | ENSG00000186666 |
| 2325 | ENSG00000184363 | ENSG00000183941 |
| 2326 | ENSG00000147804 | ENSG00000100906 |
| 2327 | ENSG00000167767 | ENSG00000267168 |
| 2328 | ENSG00000101188 | ENSG00000165556 |
| 2329 | ENSG00000111196 | ENSG00000153885 |
| 2330 | ENSG00000168237 | ENSG00000203870 |
| 2331 | ENSG00000025708 | ENSG00000160570 |
| 2332 | ENSG00000175793 | ENSG00000205869 |
| 2333 | ENSG00000158669 | ENSG00000124126 |
| 2334 | ENSG00000159322 | ENSG00000170423 |
| 2335 | ENSG00000267206 | ENSG00000111676 |
| 2336 | ENSG00000227729 | ENSG00000042445 |
| 2337 | ENSG00000171865 | ENSG00000135472 |
| 2338 | ENSG00000176887 | ENSG00000188522 |
| 2339 | ENSG00000124370 | ENSG00000115598 |
| 2340 | ENSG00000120318 | ENSG00000118702 |
| 2341 | ENSG00000099785 | ENSG00000108219 |
| 2342 | ENSG00000169410 | ENSG00000213240 |
| 2343 | ENSG00000132005 | ENSG00000164237 |
| 2344 | ENSG00000141424 | ENSG00000141293 |
| 2345 | ENSG00000256530 | ENSG00000109851 |
| 2346 | ENSG00000074266 | ENSG00000204842 |
| 2347 | ENSG00000267120 | ENSG00000138771 |
| 2348 | ENSG00000128254 | ENSG00000127554 |
| 2349 | ENSG00000203791 | ENSG00000163517 |
| 2350 | ENSG00000131043 | ENSG00000178878 |
| 2351 | ENSG00000127452 | ENSG00000198783 |
| 2352 | ENSG00000153443 | ENSG00000143158 |
| 2353 | ENSG00000129518 | ENSG00000144668 |
| 2354 | ENSG00000135124 | ENSG00000173548 |
| 2355 | ENSG00000167139 | ENSG00000141551 |
| 2356 | ENSG00000090054 | ENSG00000084636 |
| 2357 | ENSG00000127418 | ENSG00000255692 |
| 2358 | ENSG00000130312 | ENSG00000130720 |
| 2359 | ENSG00000004838 | ENSG00000089157 |
| 2360 | ENSG00000167895 | ENSG00000107611 |
| 2361 | ENSG00000198723 | ENSG00000148719 |
| 2362 | ENSG00000043355 | ENSG00000186591 |
| 2363 | ENSG00000183615 | ENSG00000261147 |
| 2364 | ENSG00000108588 | ENSG00000173757 |
| 2365 | ENSG00000177108 | ENSG00000213366 |
| 2366 | ENSG00000138356 | ENSG00000008441 |
| 2367 | ENSG00000221926 | ENSG00000198561 |
| 2368 | ENSG00000141753 | ENSG00000117691 |
| 2369 | ENSG00000078269 | ENSG00000007237 |
| 2370 | ENSG00000065970 | ENSG00000113758 |
| 2371 | ENSG00000100216 | ENSG00000165188 |
| 2372 | ENSG00000101144 | ENSG00000006712 |
| 2373 | ENSG00000185761 | ENSG00000177807 |
| 2374 | ENSG00000105397 | ENSG00000129245 |
| 2375 | ENSG00000131650 | ENSG00000101665 |
| 2376 | ENSG00000087088 | ENSG00000102996 |
| 2377 | ENSG00000104826 | ENSG00000122545 |
| 2378 | ENSG00000154162 | ENSG00000128274 |
| 2379 | ENSG00000177842 | ENSG00000183770 |
| 2380 | ENSG00000186222 | ENSG00000149679 |
| 2381 | ENSG00000136574 | ENSG00000140854 |
| 2382 | ENSG00000154122 | ENSG00000189337 |
| 2383 | ENSG00000126247 | ENSG00000125775 |
| 2384 | ENSG00000187033 | ENSG00000091651 |
| 2385 | ENSG00000156885 | ENSG00000168092 |
| 2386 | ENSG00000188536 | ENSG00000256097 |
| 2387 | ENSG00000085491 | ENSG00000163995 |
| 2388 | ENSG00000178882 | ENSG00000128272 |
| 2389 | ENSG00000166920 | ENSG00000142765 |
| 2390 | ENSG00000079337 | ENSG00000184164 |
| 2391 | ENSG00000119640 | ENSG00000184371 |
| 2392 | ENSG00000185800 | ENSG00000235978 |
| 2393 | ENSG00000143374 | ENSG00000264194 |
| 2394 | ENSG00000156515 | ENSG00000134294 |
| 2395 | ENSG00000176842 | ENSG00000134954 |
| 2396 | ENSG00000164494 | ENSG00000255046 |
| 2397 | ENSG00000140263 | ENSG00000131323 |
| 2398 | ENSG00000167588 | ENSG00000179772 |
| 2399 | ENSG00000173950 | ENSG00000160208 |
| 2400 | ENSG00000033627 | ENSG00000141574 |
| 2401 | ENSG00000130560 | ENSG00000143858 |
| 2402 | ENSG00000081014 | ENSG00000197958 |
| 2403 | ENSG00000100151 | ENSG00000049192 |
| 2404 | ENSG00000146521 | ENSG00000148841 |
| 2405 | ENSG00000163217 | ENSG00000163431 |
| 2406 | ENSG00000186583 | ENSG00000123080 |
| 2407 | ENSG00000135587 | ENSG00000184897 |
| 2408 | ENSG00000180869 | ENSG00000134824 |
| 2409 | ENSG00000167286 | ENSG00000113522 |
| 2410 | ENSG00000008405 | ENSG00000153060 |
| 2411 | ENSG00000262601 | ENSG00000167705 |
| 2412 | ENSG00000153944 | ENSG00000228804 |
| 2413 | ENSG00000180834 | ENSG00000137814 |
| 2414 | ENSG00000105971 | ENSG00000162729 |
| 2415 | ENSG00000170222 | ENSG00000109758 |
| 2416 | ENSG00000130304 | ENSG00000110583 |
| 2417 | ENSG00000161664 | ENSG00000101019 |
| 2418 | ENSG00000151773 | ENSG00000119138 |
| 2419 | ENSG00000127580 | ENSG00000157368 |
| 2420 | ENSG00000182230 | ENSG00000105649 |
| 2421 | ENSG00000178449 | ENSG00000086589 |
| 2422 | ENSG00000169154 | ENSG00000196357 |
| 2423 | ENSG00000116176 | ENSG00000063046 |
| 2424 | ENSG00000134871 | ENSG00000026036 |
| 2425 | ENSG00000183336 | ENSG00000205464 |
| 2426 | ENSG00000260092 | ENSG00000106003 |
| 2427 | ENSG00000132305 | ENSG00000143870 |
| 2428 | ENSG00000167862 | ENSG00000174996 |
| 2429 | ENSG00000068394 | ENSG00000149527 |
| 2430 | ENSG00000244122 | ENSG00000126822 |
| 2431 | ENSG00000136280 | ENSG00000160460 |
| 2432 | ENSG00000204628 | ENSG00000198125 |
| 2433 | ENSG00000130762 | ENSG00000143622 |
| 2434 | ENSG00000056736 | ENSG00000167962 |
| 2435 | ENSG00000143303 | ENSG00000163681 |
| 2436 | ENSG00000166326 | ENSG00000101294 |
| 2437 | ENSG00000135100 | ENSG00000105538 |
| 2438 | ENSG00000162695 | ENSG00000127838 |
| 2439 | ENSG00000204518 | ENSG00000198964 |
| 2440 | ENSG00000121075 | ENSG00000006016 |
| 2441 | ENSG00000165434 | ENSG00000159217 |
| 2442 | ENSG00000167236 | ENSG00000154553 |
| 2443 | ENSG00000089685 | ENSG00000173210 |
| 2444 | ENSG00000141519 | ENSG00000214530 |
| 2445 | ENSG00000119777 | ENSG00000101307 |
| 2446 | ENSG00000141933 | ENSG00000069702 |
| 2447 | ENSG00000070423 | ENSG00000146701 |
| 2448 | ENSG00000164107 | ENSG00000116299 |
| 2449 | ENSG00000157107 | ENSG00000229859 |
| 2450 | ENSG00000261740 | ENSG00000168878 |
| 2451 | ENSG00000182934 | ENSG00000160688 |
| 2452 | ENSG00000248905 | ENSG00000160051 |
| 2453 | ENSG00000142657 | ENSG00000204464 |
| 2454 | ENSG00000196968 | ENSG00000140807 |
| 2455 | ENSG00000175137 | ENSG00000076641 |
| 2456 | ENSG00000122787 | ENSG00000178562 |
| 2457 | ENSG00000257327 | ENSG00000070159 |
| 2458 | ENSG00000196230 | ENSG00000074603 |
| 2459 | ENSG00000144161 | ENSG00000165443 |
| 2460 | ENSG00000119685 | ENSG00000024422 |
| 2461 | ENSG00000147364 | ENSG00000183208 |
| 2462 | ENSG00000073331 | ENSG00000154493 |
| 2463 | ENSG00000204560 | ENSG00000128609 |
| 2464 | ENSG00000163638 | ENSG00000211584 |
| 2465 | ENSG00000106351 | ENSG00000132698 |
| 2466 | ENSG00000174586 | ENSG00000006659 |
| 2467 | ENSG00000118898 | ENSG00000089692 |
| 2468 | ENSG00000103248 | ENSG00000006118 |
| 2469 | ENSG00000161618 | ENSG00000124243 |
| 2470 | ENSG00000137509 | ENSG00000125740 |
| 2471 | ENSG00000105486 | ENSG00000134852 |
| 2472 | ENSG00000128165 | ENSG00000205018 |
| 2473 | ENSG00000135837 | ENSG00000119403 |
| 2474 | ENSG00000248098 | ENSG00000273088 |
| 2475 | ENSG00000073598 | ENSG00000137502 |
| 2476 | ENSG00000148926 | ENSG00000268130 |
| 2477 | ENSG00000187164 | ENSG00000137960 |
| 2478 | ENSG00000177225 | ENSG00000102096 |
| 2479 | ENSG00000115902 | ENSG00000087266 |
| 2480 | ENSG00000205300 | ENSG00000150967 |
| 2481 | ENSG00000171163 | ENSG00000113712 |
| 2482 | ENSG00000048707 | ENSG00000120885 |
| 2483 | ENSG00000152208 | ENSG00000197892 |
| 2484 | ENSG00000244291 | ENSG00000099203 |
| 2485 | ENSG00000196739 | ENSG00000159314 |
| 2486 | ENSG00000269018 | ENSG00000169605 |
| 2487 | ENSG00000183963 | ENSG00000269417 |
| 2488 | ENSG00000174226 | ENSG00000162522 |
| 2489 | ENSG00000159352 | ENSG00000196839 |
| 2490 | ENSG00000198792 | ENSG00000196544 |
| 2491 | ENSG00000127463 | ENSG00000140497 |
| 2492 | ENSG00000167565 | ENSG00000269259 |
| 2493 | ENSG00000100311 | ENSG00000117616 |
| 2494 | ENSG00000179363 | ENSG00000167106 |
| 2495 | ENSG00000186075 | ENSG00000136634 |
| 2496 | ENSG00000184154 | ENSG00000087274 |
| 2497 | ENSG00000152475 | ENSG00000088808 |
| 2498 | ENSG00000177030 | ENSG00000079691 |
| 2499 | ENSG00000132965 | ENSG00000168710 |
| 2500 | ENSG00000137821 | ENSG00000148337 |
| 2501 | ENSG00000254772 | ENSG00000136270 |
| 2502 | ENSG00000171793 | ENSG00000268396 |
| 2503 | ENSG00000108924 | ENSG00000177105 |
| 2504 | ENSG00000171282 | ENSG00000173064 |
| 2505 | ENSG00000204634 | ENSG00000120549 |
| 2506 | ENSG00000166783 | ENSG00000068400 |
| 2507 | ENSG00000138640 | ENSG00000234776 |
| 2508 | ENSG00000134440 | ENSG00000103485 |
| 2509 | ENSG00000136286 | ENSG00000203811 |
| 2510 | ENSG00000144713 | ENSG00000112039 |
| 2511 | ENSG00000100249 | ENSG00000242372 |
| 2512 | ENSG00000129493 | ENSG00000183258 |
| 2513 | ENSG00000100298 | ENSG00000176087 |
| 2514 | ENSG00000196139 | ENSG00000121104 |
| 2515 | ENSG00000136877 | ENSG00000125966 |
| 2516 | ENSG00000203485 | ENSG00000153292 |
| 2517 | ENSG00000139926 | ENSG00000172939 |
| 2518 | ENSG00000100902 | ENSG00000176302 |
| 2519 | ENSG00000090932 | ENSG00000123999 |
| 2520 | ENSG00000100033 | ENSG00000187045 |
| 2521 | ENSG00000131848 | ENSG00000184182 |
| 2522 | ENSG00000119383 | ENSG00000107957 |
| 2523 | ENSG00000187244 | ENSG00000176473 |
| 2524 | ENSG00000197208 | ENSG00000137411 |
| 2525 | ENSG00000148396 | ENSG00000140297 |
| 2526 | ENSG00000213921 | ENSG00000162747 |
| 2527 | ENSG00000179954 | ENSG00000118162 |
| 2528 | ENSG00000109670 | ENSG00000103647 |
| 2529 | ENSG00000269879 | ENSG00000106772 |
| 2530 | ENSG00000258130 | ENSG00000157916 |
| 2531 | ENSG00000119280 | ENSG00000111912 |
| 2532 | ENSG00000175899 | ENSG00000117298 |
| 2533 | ENSG00000137275 | ENSG00000184076 |
| 2534 | ENSG00000101161 | ENSG00000188897 |
| 2535 | ENSG00000163879 | ENSG00000152022 |
| 2536 | ENSG00000172660 | ENSG00000180398 |
| 2537 | ENSG00000168621 | ENSG00000249459 |
| 2538 | ENSG00000144642 | ENSG00000163191 |
| 2539 | ENSG00000173714 | ENSG00000101474 |
| 2540 | ENSG00000089737 | ENSG00000172893 |
| 2541 | ENSG00000145623 | ENSG00000140750 |
| 2542 | ENSG00000163993 | ENSG00000100321 |
| 2543 | ENSG00000180525 | ENSG00000070718 |
| 2544 | ENSG00000162438 | ENSG00000164920 |
| 2545 | ENSG00000159176 | ENSG00000112576 |
| 2546 | ENSG00000108479 | ENSG00000267937 |
| 2547 | ENSG00000161800 | ENSG00000173567 |
| 2548 | ENSG00000136003 | ENSG00000139438 |
| 2549 | ENSG00000161888 | ENSG00000076826 |
| 2550 | ENSG00000204532 | ENSG00000268858 |
| 2551 | ENSG00000188011 | ENSG00000188997 |
| 2552 | ENSG00000107984 | ENSG00000122678 |
| 2553 | ENSG00000130881 | ENSG00000143514 |
| 2554 | ENSG00000244405 | ENSG00000122986 |
| 2555 | ENSG00000165588 | ENSG00000159479 |
| 2556 | ENSG00000212743 | ENSG00000187994 |
| 2557 | ENSG00000173085 | ENSG00000118407 |
| 2558 | ENSG00000171631 | ENSG00000118640 |
| 2559 | ENSG00000122641 | ENSG00000102977 |
| 2560 | ENSG00000110799 | ENSG00000092969 |
| 2561 | ENSG00000111729 | ENSG00000084731 |
| 2562 | ENSG00000103326 | ENSG00000100353 |
| 2563 | ENSG00000227507 | ENSG00000182613 |
| 2564 | ENSG00000100804 | ENSG00000106366 |
| 2565 | ENSG00000079101 | ENSG00000011485 |
| 2566 | ENSG00000130340 | ENSG00000261594 |
| 2567 | ENSG00000267001 | ENSG00000126264 |
| 2568 | ENSG00000158104 | ENSG00000213523 |
| 2569 | ENSG00000115461 | ENSG00000171097 |
| 2570 | ENSG00000198754 | ENSG00000254087 |
| 2571 | ENSG00000108255 | ENSG00000104804 |
| 2572 | ENSG00000113407 | ENSG00000176678 |
| 2573 | ENSG00000013583 | ENSG00000137343 |
| 2574 | ENSG00000140463 | ENSG00000162517 |
| 2575 | ENSG00000178175 | ENSG00000167986 |
| 2576 | ENSG00000169247 | ENSG00000171747 |
| 2577 | ENSG00000134046 | ENSG00000118705 |
| 2578 | ENSG00000197880 | ENSG00000124831 |
| 2579 | ENSG00000127603 | ENSG00000254732 |
| 2580 | ENSG00000163803 | ENSG00000165949 |
| 2581 | ENSG00000100319 | ENSG00000168887 |
| 2582 | ENSG00000197381 | ENSG00000130830 |
| 2583 | ENSG00000214063 | ENSG00000101337 |
| 2584 | ENSG00000213949 | ENSG00000171858 |
| 2585 | ENSG00000264813 | ENSG00000129946 |
| 2586 | ENSG00000108829 | ENSG00000142733 |
| 2587 | ENSG00000104866 | ENSG00000105329 |
| 2588 | ENSG00000213760 | ENSG00000175535 |
| 2589 | ENSG00000147471 | ENSG00000130669 |
| 2590 | ENSG00000065361 | ENSG00000173517 |
| 2591 | ENSG00000182979 | ENSG00000229314 |
| 2592 | ENSG00000063322 | ENSG00000171159 |
| 2593 | ENSG00000171570 | ENSG00000184900 |
| 2594 | ENSG00000135720 | ENSG00000173678 |
| 2595 | ENSG00000204446 | ENSG00000131183 |
| 2596 | ENSG00000268863 | ENSG00000081189 |
| 2597 | ENSG00000107731 | ENSG00000157322 |
| 2598 | ENSG00000180432 | ENSG00000185551 |
| 2599 | ENSG00000184009 | ENSG00000138018 |
| 2600 | ENSG00000148335 | ENSG00000107821 |
| 2601 | ENSG00000262481 | ENSG00000133884 |
| 2602 | ENSG00000166501 | ENSG00000166595 |
| 2603 | ENSG00000178229 | ENSG00000101040 |
| 2604 | ENSG00000166170 | ENSG00000151553 |
| 2605 | ENSG00000104635 | ENSG00000182512 |
| 2606 | ENSG00000050820 | ENSG00000188822 |
| 2607 | ENSG00000108961 | ENSG00000102032 |
| 2608 | ENSG00000226777 | ENSG00000138180 |
| 2609 | ENSG00000185338 | ENSG00000163430 |
| 2610 | ENSG00000167671 | ENSG00000102891 |
| 2611 | ENSG00000011105 | ENSG00000198518 |
| 2612 | ENSG00000114737 | ENSG00000069329 |
| 2613 | ENSG00000204264 | ENSG00000160472 |
| 2614 | ENSG00000114503 | ENSG00000122176 |
| 2615 | ENSG00000144401 | ENSG00000136002 |
| 2616 | ENSG00000164729 | ENSG00000154370 |
| 2617 | ENSG00000185347 | ENSG00000136111 |
| 2618 | ENSG00000162959 | ENSG00000188735 |
| 2619 | ENSG00000189067 | ENSG00000118096 |
| 2620 | ENSG00000174106 | ENSG00000131669 |
| 2621 | ENSG00000179886 | ENSG00000168904 |
| 2622 | ENSG00000119547 | ENSG00000166128 |
| 2623 | ENSG00000213780 | ENSG00000174744 |
| 2624 | ENSG00000010379 | ENSG00000196859 |
| 2625 | ENSG00000167434 | ENSG00000137834 |
| 2626 | ENSG00000187513 | ENSG00000145283 |
| 2627 | ENSG00000148180 | ENSG00000105072 |
| 2628 | ENSG00000203630 | ENSG00000197150 |
| 2629 | ENSG00000126773 | ENSG00000135773 |
| 2630 | ENSG00000128709 | ENSG00000172345 |
| 2631 | ENSG00000204323 | ENSG00000154016 |
| 2632 | ENSG00000155542 | ENSG00000159842 |
| 2633 | ENSG00000183307 | ENSG00000167723 |
| 2634 | ENSG00000269104 | ENSG00000102935 |
| 2635 | ENSG00000074590 | ENSG00000135931 |
| 2636 | ENSG00000254873 | ENSG00000148343 |
| 2637 | ENSG00000162063 | ENSG00000145506 |
| 2638 | ENSG00000179715 | ENSG00000177042 |
| 2639 | ENSG00000165782 | ENSG00000177674 |
| 2640 | ENSG00000160050 | ENSG00000188124 |
| 2641 | ENSG00000134317 | ENSG00000221914 |
| 2642 | ENSG00000054598 | ENSG00000115286 |
| 2643 | ENSG00000106511 | ENSG00000185245 |
| 2644 | ENSG00000129028 | ENSG00000137767 |
| 2645 | ENSG00000118369 | ENSG00000136451 |
| 2646 | ENSG00000206562 | ENSG00000180389 |
| 2647 | ENSG00000249853 | ENSG00000171596 |
| 2648 | ENSG00000196436 | ENSG00000143156 |
| 2649 | ENSG00000150687 | ENSG00000256713 |
| 2650 | ENSG00000171700 | ENSG00000170421 |
| 2651 | ENSG00000129007 | ENSG00000105953 |
| 2652 | ENSG00000130772 | ENSG00000169783 |
| 2653 | ENSG00000268457 | ENSG00000104687 |
| 2654 | ENSG00000189001 | ENSG00000166477 |
| 2655 | ENSG00000084112 | ENSG00000172247 |
| 2656 | ENSG00000197746 | ENSG00000214290 |
| 2657 | ENSG00000126214 | ENSG00000181513 |
| 2658 | ENSG00000140391 | ENSG00000067225 |
| 2659 | ENSG00000204314 | ENSG00000122861 |
| 2660 | ENSG00000145020 | ENSG00000103769 |
| 2661 | ENSG00000186564 | ENSG00000128563 |
| 2662 | ENSG00000152620 | ENSG00000269783 |
| 2663 | ENSG00000101407 | ENSG00000205236 |
| 2664 | ENSG00000166922 | ENSG00000175764 |
| 2665 | ENSG00000107485 | ENSG00000163815 |
| 2666 | ENSG00000019582 | ENSG00000108061 |
| 2667 | ENSG00000197162 | ENSG00000075413 |
| 2668 | ENSG00000060566 | ENSG00000176209 |
| 2669 | ENSG00000163545 | ENSG00000009790 |
| 2670 | ENSG00000108821 | ENSG00000099622 |
| 2671 | ENSG00000116977 | ENSG00000157637 |
| 2672 | ENSG00000138032 | ENSG00000116604 |
| 2673 | ENSG00000256500 | ENSG00000171617 |
| 2674 | ENSG00000160953 | ENSG00000037965 |
| 2675 | ENSG00000151778 | ENSG00000152240 |
| 2676 | ENSG00000163492 | ENSG00000269295 |
| 2677 | ENSG00000113448 | ENSG00000173230 |
| 2678 | ENSG00000184254 | ENSG00000214854 |
| 2679 | ENSG00000140522 | ENSG00000114631 |
| 2680 | ENSG00000197324 | ENSG00000184470 |
| 2681 | ENSG00000007372 | ENSG00000168135 |
| 2682 | ENSG00000160973 | ENSG00000164818 |
| 2683 | ENSG00000188828 | ENSG00000112759 |
| 2684 | ENSG00000185917 | ENSG00000172380 |
| 2685 | ENSG00000119333 | ENSG00000105131 |
| 2686 | ENSG00000072110 | ENSG00000263588 |
| 2687 | ENSG00000130037 | ENSG00000125508 |
| 2688 | ENSG00000133275 | ENSG00000099957 |
| 2689 | ENSG00000104825 | ENSG00000117222 |
| 2690 | ENSG00000214706 | ENSG00000171962 |
| 2691 | ENSG00000100139 | ENSG00000130377 |
| 2692 | ENSG00000131779 | ENSG00000070831 |
| 2693 | ENSG00000170345 | ENSG00000176058 |
| 2694 | ENSG00000048545 | ENSG00000136828 |
| 2695 | ENSG00000016391 | ENSG00000139364 |
| 2696 | ENSG00000160097 | ENSG00000135480 |
| 2697 | ENSG00000161940 | ENSG00000175877 |
| 2698 | ENSG00000177992 | ENSG00000213923 |
| 2699 | ENSG00000172803 | ENSG00000141556 |
| 2700 | ENSG00000255468 | ENSG00000135956 |
| 2701 | ENSG00000176928 | ENSG00000110446 |
| 2702 | ENSG00000204619 | ENSG00000183833 |
| 2703 | ENSG00000233927 | ENSG00000154277 |
| 2704 | ENSG00000160948 | ENSG00000116455 |
| 2705 | ENSG00000105576 | ENSG00000186716 |
| 2706 | ENSG00000151651 | ENSG00000228049 |
| 2707 | ENSG00000177311 | ENSG00000142166 |
| 2708 | ENSG00000139880 | ENSG00000168038 |
| 2709 | ENSG00000248235 | ENSG00000269205 |
| 2710 | ENSG00000108395 | ENSG00000169704 |
| 2711 | ENSG00000175643 | ENSG00000060971 |
| 2712 | ENSG00000165917 | ENSG00000158545 |
| 2713 | ENSG00000162496 | ENSG00000104884 |
| 2714 | ENSG00000001084 | ENSG00000165915 |
| 2715 | ENSG00000172757 | ENSG00000167613 |
| 2716 | ENSG00000143891 | ENSG00000111684 |
| 2717 | ENSG00000128294 | ENSG00000142182 |
| 2718 | ENSG00000148120 | ENSG00000100083 |
| 2719 | ENSG00000131446 | ENSG00000118526 |
| 2720 | ENSG00000139263 | ENSG00000116962 |
| 2721 | ENSG00000108551 | ENSG00000175329 |
| 2722 | ENSG00000159871 | ENSG00000161267 |
| 2723 | ENSG00000197595 | ENSG00000167476 |
| 2724 | ENSG00000164951 | ENSG00000182257 |
| 2725 | ENSG00000112337 | ENSG00000141738 |
| 2726 | ENSG00000159256 | ENSG00000167414 |
| 2727 | ENSG00000119906 | ENSG00000134313 |
| 2728 | ENSG00000165996 | ENSG00000064787 |
| 2729 | ENSG00000179580 | ENSG00000110080 |
| 2730 | ENSG00000214402 | ENSG00000160951 |
| 2731 | ENSG00000103544 | ENSG00000149021 |
| 2732 | ENSG00000115970 | ENSG00000154930 |
| 2733 | ENSG00000166523 | ENSG00000182372 |
| 2734 | ENSG00000115935 | ENSG00000269810 |
| 2735 | ENSG00000108984 | ENSG00000099875 |
| 2736 | ENSG00000133657 | ENSG00000172789 |
| 2737 | ENSG00000204610 | ENSG00000129255 |
| 2738 | ENSG00000161265 | ENSG00000173080 |
| 2739 | ENSG00000109775 | ENSG00000161202 |
| 2740 | ENSG00000122733 | ENSG00000143554 |
| 2741 | ENSG00000127152 | ENSG00000134222 |
| 2742 | ENSG00000149218 | ENSG00000245848 |
| 2743 | ENSG00000101425 | ENSG00000167615 |
| 2744 | ENSG00000243627 | ENSG00000185863 |
| 2745 | ENSG00000268941 | ENSG00000100243 |
| 2746 | ENSG00000215845 | ENSG00000185933 |
| 2747 | ENSG00000184937 | ENSG00000197872 |
| 2748 | ENSG00000035687 | ENSG00000060982 |
| 2749 | ENSG00000167461 | ENSG00000233670 |
| 2750 | ENSG00000143314 | ENSG00000022976 |
| 2751 | ENSG00000120057 | ENSG00000122643 |
| 2752 | ENSG00000111144 | ENSG00000204256 |
| 2753 | ENSG00000172650 | ENSG00000114742 |
| 2754 | ENSG00000164647 | ENSG00000105767 |
| 2755 | ENSG00000073067 | ENSG00000270136 |
| 2756 | ENSG00000160753 | ENSG00000159788 |
| 2757 | ENSG00000157110 | ENSG00000253787 |
| 2758 | ENSG00000213221 | ENSG00000259009 |
| 2759 | ENSG00000234745 | ENSG00000167281 |
| 2760 | ENSG00000065413 | ENSG00000172845 |
| 2761 | ENSG00000233502 | ENSG00000057593 |
| 2762 | ENSG00000169756 | ENSG00000103037 |
| 2763 | ENSG00000061936 | ENSG00000184908 |
| 2764 | ENSG00000140320 | ENSG00000187741 |
| 2765 | ENSG00000156486 | ENSG00000005486 |
| 2766 | ENSG00000130158 | ENSG00000055483 |
| 2767 | ENSG00000182752 | ENSG00000158748 |
| 2768 | ENSG00000072062 | ENSG00000105705 |
| 2769 | ENSG00000198250 | ENSG00000269047 |
| 2770 | ENSG00000157557 | ENSG00000184557 |
| 2771 | ENSG00000170315 | ENSG00000162144 |
| 2772 | ENSG00000079387 | ENSG00000148225 |
| 2773 | ENSG00000115956 | ENSG00000132646 |
| 2774 | ENSG00000156508 | ENSG00000143486 |
| 2775 | ENSG00000123388 | ENSG00000219073 |
| 2776 | ENSG00000169554 | ENSG00000125637 |
| 2777 | ENSG00000166535 | ENSG00000134160 |
| 2778 | ENSG00000180155 | ENSG00000176697 |
| 2779 | ENSG00000167800 | ENSG00000196224 |
| 2780 | ENSG00000104517 | ENSG00000180720 |
| 2781 | ENSG00000094661 | ENSG00000136717 |
| 2782 | ENSG00000135205 | ENSG00000172379 |
| 2783 | ENSG00000133063 | ENSG00000113140 |
| 2784 | ENSG00000129932 | ENSG00000143569 |
| 2785 | ENSG00000155666 | ENSG00000171206 |
| 2786 | ENSG00000092036 | ENSG00000168028 |
| 2787 | ENSG00000181885 | ENSG00000132680 |
| 2788 | ENSG00000185475 | ENSG00000075618 |
| 2789 | ENSG00000136244 | ENSG00000183760 |
| 2790 | ENSG00000138411 | ENSG00000179913 |
| 2791 | ENSG00000212900 | ENSG00000143167 |
| 2792 | ENSG00000258928 | ENSG00000139800 |
| 2793 | ENSG00000154920 | ENSG00000166833 |
| 2794 | ENSG00000108733 | ENSG00000148154 |
| 2795 | ENSG00000110367 | ENSG00000118655 |
| 2796 | ENSG00000128805 | ENSG00000173762 |
| 2797 | ENSG00000099817 | ENSG00000122557 |
| 2798 | ENSG00000105889 | ENSG00000141582 |
| 2799 | ENSG00000002746 | ENSG00000140009 |
| 2800 | ENSG00000094963 | ENSG00000198931 |
| 2801 | ENSG00000111752 | ENSG00000132881 |
| 2802 | ENSG00000187867 | ENSG00000268040 |
| 2803 | ENSG00000110713 | ENSG00000146054 |
| 2804 | ENSG00000116675 | ENSG00000141699 |
| 2805 | ENSG00000164307 | ENSG00000269058 |
| 2806 | ENSG00000183856 | ENSG00000171431 |
| 2807 | ENSG00000148704 | ENSG00000154845 |
| 2808 | ENSG00000183077 | ENSG00000104835 |
| 2809 | ENSG00000177398 | ENSG00000121933 |
| 2810 | ENSG00000257987 | ENSG00000122970 |
| 2811 | ENSG00000140968 | ENSG00000152977 |
| 2812 | ENSG00000152556 | ENSG00000241945 |
| 2813 | ENSG00000100644 | ENSG00000213892 |
| 2814 | ENSG00000249961 | ENSG00000099985 |
| 2815 | ENSG00000248727 | ENSG00000101342 |
| 2816 | ENSG00000104901 | ENSG00000268172 |
| 2817 | ENSG00000130921 | ENSG00000135299 |
| 2818 | ENSG00000160606 | ENSG00000110880 |
| 2819 | ENSG00000180574 | ENSG00000167470 |
| 2820 | ENSG00000238244 | ENSG00000185905 |
| 2821 | ENSG00000008516 | ENSG00000186103 |
| 2822 | ENSG00000214736 | ENSG00000164054 |
| 2823 | ENSG00000182676 | ENSG00000182132 |
| 2824 | ENSG00000132024 | ENSG00000164828 |
| 2825 | ENSG00000101577 | ENSG00000124181 |
| 2826 | ENSG00000127831 | ENSG00000164885 |
| 2827 | ENSG00000146618 | ENSG00000186660 |
| 2828 | ENSG00000008083 | ENSG00000155096 |
| 2829 | ENSG00000169129 | ENSG00000116771 |
| 2830 | ENSG00000143641 | ENSG00000267368 |
| 2831 | ENSG00000186806 | ENSG00000103313 |
| 2832 | ENSG00000163386 | ENSG00000100714 |
| 2833 | ENSG00000069974 | ENSG00000161281 |
| 2834 | ENSG00000166436 | ENSG00000162086 |
| 2835 | ENSG00000185633 | ENSG00000259495 |
| 2836 | ENSG00000166398 | ENSG00000185522 |
| 2837 | ENSG00000232774 | ENSG00000138166 |
| 2838 | ENSG00000153814 | ENSG00000173566 |
| 2839 | ENSG00000091073 | ENSG00000186187 |
| 2840 | ENSG00000160282 | ENSG00000114019 |
| 2841 | ENSG00000240224 | ENSG00000042832 |
| 2842 | ENSG00000170500 | ENSG00000229117 |
| 2843 | ENSG00000118939 | ENSG00000163558 |
| 2844 | ENSG00000149346 | ENSG00000135437 |
| 2845 | ENSG00000133393 | ENSG00000183318 |
| 2846 | ENSG00000182087 | ENSG00000150093 |
| 2847 | ENSG00000169169 | ENSG00000171060 |
| 2848 | ENSG00000144655 | ENSG00000111252 |
| 2849 | ENSG00000186642 | ENSG00000184270 |
| 2850 | ENSG00000160813 | ENSG00000114982 |
| 2851 | ENSG00000244607 | ENSG00000168610 |
| 2852 | ENSG00000143363 | ENSG00000104953 |
| 2853 | ENSG00000110315 | ENSG00000149571 |
| 2854 | ENSG00000005844 | ENSG00000069399 |
| 2855 | ENSG00000254806 | ENSG00000178971 |
| 2856 | ENSG00000160741 | ENSG00000139433 |
| 2857 | ENSG00000100101 | ENSG00000169490 |
| 2858 | ENSG00000075643 | ENSG00000108179 |
| 2859 | ENSG00000144868 | ENSG00000174306 |
| 2860 | ENSG00000089351 | ENSG00000168439 |
| 2861 | ENSG00000227268 | ENSG00000133027 |
| 2862 | ENSG00000177663 | ENSG00000130787 |
| 2863 | ENSG00000182095 | ENSG00000149591 |
| 2864 | ENSG00000163686 | ENSG00000138303 |
| 2865 | ENSG00000186260 | ENSG00000150455 |
| 2866 | ENSG00000183287 | ENSG00000153786 |
| 2867 | ENSG00000115317 | ENSG00000167699 |
| 2868 | ENSG00000198734 | ENSG00000111725 |
| 2869 | ENSG00000109814 | ENSG00000177542 |
| 2870 | ENSG00000240021 | ENSG00000268317 |
| 2871 | ENSG00000123810 | ENSG00000110906 |
| 2872 | ENSG00000163297 | ENSG00000125611 |
| 2873 | ENSG00000173334 | ENSG00000135424 |
| 2874 | ENSG00000264668 | ENSG00000071205 |
| 2875 | ENSG00000179751 | ENSG00000242802 |
| 2876 | ENSG00000105697 | ENSG00000008324 |
| 2877 | ENSG00000268800 | ENSG00000023191 |
| 2878 | ENSG00000099866 | ENSG00000111802 |
| 2879 | ENSG00000119042 | ENSG00000091428 |
| 2880 | ENSG00000161996 | ENSG00000175155 |
| 2881 | ENSG00000070190 | ENSG00000173638 |
| 2882 | ENSG00000204569 | ENSG00000105327 |
| 2883 | ENSG00000087303 | ENSG00000255062 |
| 2884 | ENSG00000184207 | ENSG00000104415 |
| 2885 | ENSG00000241837 | ENSG00000104903 |
| 2886 | ENSG00000128283 | ENSG00000160293 |
| 2887 | ENSG00000237102 | ENSG00000158941 |
| 2888 | ENSG00000138660 | ENSG00000071246 |
| 2889 | ENSG00000109667 | ENSG00000004534 |
| 2890 | ENSG00000108602 | ENSG00000267314 |
| 2891 | ENSG00000100503 | ENSG00000126746 |
| 2892 | ENSG00000156381 | ENSG00000163520 |
| 2893 | ENSG00000232434 | ENSG00000158636 |
| 2894 | ENSG00000085978 | ENSG00000169418 |
| 2895 | ENSG00000173588 | ENSG00000149418 |
| 2896 | ENSG00000176974 | ENSG00000143153 |
| 2897 | ENSG00000128604 | ENSG00000071553 |
| 2898 | ENSG00000104973 | ENSG00000078487 |
| 2899 | ENSG00000144677 | ENSG00000213995 |
| 2900 | ENSG00000257218 | ENSG00000164331 |
| 2901 | ENSG00000256018 | ENSG00000117122 |
| 2902 | ENSG00000175198 | ENSG00000136942 |
| 2903 | ENSG00000006025 | ENSG00000177595 |
| 2904 | ENSG00000177202 | ENSG00000158023 |
| 2905 | ENSG00000260001 | ENSG00000149573 |
| 2906 | ENSG00000167483 | ENSG00000157653 |
| 2907 | ENSG00000213424 | ENSG00000174358 |
| 2908 | ENSG00000163684 | ENSG00000056972 |
| 2909 | ENSG00000139625 | ENSG00000255284 |
| 2910 | ENSG00000179271 | ENSG00000088298 |
| 2911 | ENSG00000180259 | ENSG00000153904 |
| 2912 | ENSG00000170004 | ENSG00000204991 |
| 2913 | ENSG00000099821 | ENSG00000203711 |
| 2914 | ENSG00000140941 | ENSG00000140506 |
| 2915 | ENSG00000221947 | ENSG00000054356 |
| 2916 | ENSG00000047457 | ENSG00000186334 |
| 2917 | ENSG00000188100 | ENSG00000107738 |
| 2918 | ENSG00000160223 | ENSG00000163914 |
| 2919 | ENSG00000249160 | ENSG00000171853 |
| 2920 | ENSG00000153187 | ENSG00000258539 |
| 2921 | ENSG00000140459 | ENSG00000174093 |
| 2922 | ENSG00000068489 | ENSG00000179761 |
| 2923 | ENSG00000146090 | ENSG00000100266 |
| 2924 | ENSG00000106066 | ENSG00000134138 |
| 2925 | ENSG00000167165 | ENSG00000132824 |
| 2926 | ENSG00000268885 | ENSG00000197301 |
| 2927 | ENSG00000221962 | ENSG00000160161 |
| 2928 | ENSG00000204060 | ENSG00000079616 |
| 2929 | ENSG00000160691 | ENSG00000151474 |
| 2930 | ENSG00000215421 | ENSG00000161533 |
| 2931 | ENSG00000251493 | ENSG00000188501 |
| 2932 | ENSG00000146067 | ENSG00000186174 |
| 2933 | ENSG00000064651 | ENSG00000100422 |
| 2934 | ENSG00000148488 | ENSG00000070731 |
| 2935 | ENSG00000185664 | ENSG00000135334 |
| 2936 | ENSG00000126603 | ENSG00000114656 |
| 2937 | ENSG00000196396 | ENSG00000186854 |
| 2938 | ENSG00000163239 | ENSG00000053254 |
| 2939 | ENSG00000111674 | ENSG00000072958 |
| 2940 | ENSG00000259900 | ENSG00000214413 |
| 2941 | ENSG00000198814 | ENSG00000125409 |
| 2942 | ENSG00000163041 | ENSG00000087074 |
| 2943 | ENSG00000186891 | ENSG00000150990 |
| 2944 | ENSG00000174307 | ENSG00000179241 |
| 2945 | ENSG00000111670 | ENSG00000104827 |
| 2946 | ENSG00000171763 | ENSG00000148834 |
| 2947 | ENSG00000079215 | ENSG00000105771 |
| 2948 | ENSG00000102870 | ENSG00000183476 |
| 2949 | ENSG00000127445 | ENSG00000249222 |
| 2950 | ENSG00000133808 | ENSG00000198821 |
| 2951 | ENSG00000213199 | ENSG00000197818 |
| 2952 | ENSG00000174236 | ENSG00000088832 |
| 2953 | ENSG00000179598 | ENSG00000148943 |
| 2954 | ENSG00000103067 | ENSG00000148814 |
| 2955 | ENSG00000157119 | ENSG00000104812 |
| 2956 | ENSG00000228768 | ENSG00000187051 |
| 2957 | ENSG00000156170 | ENSG00000196570 |
| 2958 | ENSG00000170855 | ENSG00000112977 |
| 2959 | ENSG00000119844 | ENSG00000182646 |
| 2960 | ENSG00000106526 | ENSG00000104918 |
| 2961 | ENSG00000067113 | ENSG00000124782 |
| 2962 | ENSG00000180354 | ENSG00000196663 |
| 2963 | ENSG00000010704 | ENSG00000070182 |
| 2964 | ENSG00000197006 | ENSG00000137709 |
| 2965 | ENSG00000141985 | ENSG00000185386 |
| 2966 | ENSG00000136040 | ENSG00000172175 |
| 2967 | ENSG00000180626 | ENSG00000140848 |
| 2968 | ENSG00000143669 | ENSG00000188783 |
| 2969 | ENSG00000089159 | ENSG00000139899 |
| 2970 | ENSG00000172366 | ENSG00000143847 |
| 2971 | ENSG00000197705 | ENSG00000196209 |
| 2972 | ENSG00000119655 | ENSG00000166173 |
| 2973 | ENSG00000142627 | ENSG00000130511 |
| 2974 | ENSG00000055208 | ENSG00000204574 |
| 2975 | ENSG00000168067 | ENSG00000130723 |
| 2976 | ENSG00000099804 | ENSG00000133315 |
| 2977 | ENSG00000120071 | ENSG00000214732 |
| 2978 | ENSG00000001617 | ENSG00000015532 |
| 2979 | ENSG00000172382 | ENSG00000120733 |
| 2980 | ENSG00000139436 | ENSG00000136478 |
| 2981 | ENSG00000147439 | ENSG00000105227 |
| 2982 | ENSG00000268898 | ENSG00000109458 |
| 2983 | ENSG00000198910 | ENSG00000153879 |
| 2984 | ENSG00000125691 | ENSG00000112699 |
| 2985 | ENSG00000189269 | ENSG00000054967 |
| 2986 | ENSG00000135426 | ENSG00000166794 |
| 2987 | ENSG00000110887 | ENSG00000183908 |
| 2988 | ENSG00000124693 | ENSG00000260861 |
| 2989 | ENSG00000060642 | ENSG00000168350 |
| 2990 | ENSG00000168826 | ENSG00000159596 |
| 2991 | ENSG00000077942 | ENSG00000139437 |
| 2992 | ENSG00000133477 | ENSG00000183605 |
| 2993 | ENSG00000196569 | ENSG00000111642 |
| 2994 | ENSG00000067221 | ENSG00000197948 |
| 2995 | ENSG00000136383 | ENSG00000130202 |
| 2996 | ENSG00000162735 | ENSG00000102024 |
| 2997 | ENSG00000186765 | ENSG00000188032 |
| 2998 | ENSG00000078687 | ENSG00000131242 |
| 2999 | ENSG00000185122 | ENSG00000263053 |
| 3000 | ENSG00000100028 | ENSG00000203812 |
| 3001 | ENSG00000178150 | ENSG00000164300 |
| 3002 | ENSG00000133048 | ENSG00000153406 |
| 3003 | ENSG00000142606 | ENSG00000152518 |
| 3004 | ENSG00000243660 | ENSG00000177106 |
| 3005 | ENSG00000113525 | ENSG00000117984 |
| 3006 | ENSG00000177830 | ENSG00000170727 |
| 3007 | ENSG00000170860 | ENSG00000117280 |
| 3008 | ENSG00000185101 | ENSG00000108296 |
| 3009 | ENSG00000096060 | ENSG00000104375 |
| 3010 | ENSG00000167046 | ENSG00000078403 |
| 3011 | ENSG00000183250 | ENSG00000167772 |
| 3012 | ENSG00000144445 | ENSG00000000938 |
| 3013 | ENSG00000165175 | ENSG00000159063 |
| 3014 | ENSG00000131061 | ENSG00000130779 |
| 3015 | ENSG00000174885 | ENSG00000128335 |
| 3016 | ENSG00000023228 | ENSG00000167658 |
| 3017 | ENSG00000068024 | ENSG00000174915 |
| 3018 | ENSG00000125952 | ENSG00000269165 |
| 3019 | ENSG00000175489 | ENSG00000204677 |
| 3020 | ENSG00000070526 | ENSG00000172081 |
| 3021 | ENSG00000143845 | ENSG00000161904 |
| 3022 | ENSG00000115289 | ENSG00000088538 |
| 3023 | ENSG00000138606 | ENSG00000112246 |
| 3024 | ENSG00000189339 | ENSG00000215021 |
| 3025 | ENSG00000100030 | ENSG00000062822 |
| 3026 | ENSG00000154102 | ENSG00000111291 |
| 3027 | ENSG00000121903 | ENSG00000100100 |
| 3028 | ENSG00000182185 | ENSG00000189320 |
| 3029 | ENSG00000214300 | ENSG00000148660 |
| 3030 | ENSG00000141376 | ENSG00000115590 |
| 3031 | ENSG00000085433 | ENSG00000198752 |
| 3032 | ENSG00000102854 | ENSG00000135744 |
| 3033 | ENSG00000197355 | ENSG00000136819 |
| 3034 | ENSG00000204482 | ENSG00000204347 |
| 3035 | ENSG00000136653 | ENSG00000079805 |
| 3036 | ENSG00000241322 | ENSG00000128626 |
| 3037 | ENSG00000137216 | ENSG00000111348 |
| 3038 | ENSG00000154146 | ENSG00000105355 |
| 3039 | ENSG00000089280 | ENSG00000163947 |
| 3040 | ENSG00000163975 | ENSG00000176531 |
| 3041 | ENSG00000135549 | ENSG00000115884 |
| 3042 | ENSG00000105519 | ENSG00000178217 |
| 3043 | ENSG00000160256 | ENSG00000118160 |
| 3044 | ENSG00000130193 | ENSG00000145916 |
| 3045 | ENSG00000180881 | ENSG00000183260 |
| 3046 | ENSG00000125458 | ENSG00000099783 |
| 3047 | ENSG00000112242 | ENSG00000007968 |
| 3048 | ENSG00000197296 | ENSG00000100320 |
| 3049 | ENSG00000153147 | ENSG00000157551 |
| 3050 | ENSG00000102921 | ENSG00000211450 |
| 3051 | ENSG00000238227 | ENSG00000164989 |
| 3052 | ENSG00000179335 | ENSG00000143924 |
| 3053 | ENSG00000176108 | ENSG00000171471 |
| 3054 | ENSG00000078399 | ENSG00000268865 |
| 3055 | ENSG00000163909 | ENSG00000203876 |
| 3056 | ENSG00000101246 | ENSG00000089693 |
| 3057 | ENSG00000126698 | ENSG00000112531 |
| 3058 | ENSG00000128346 | ENSG00000103335 |
| 3059 | ENSG00000244731 | ENSG00000028277 |
| 3060 | ENSG00000182827 | ENSG00000111481 |
| 3061 | ENSG00000215440 | ENSG00000165495 |
| 3062 | ENSG00000072864 | ENSG00000269676 |
| 3063 | ENSG00000137310 | ENSG00000229809 |
| 3064 | ENSG00000125245 | ENSG00000154027 |
| 3065 | ENSG00000140525 | ENSG00000162728 |
| 3066 | ENSG00000154328 | ENSG00000115756 |
| 3067 | ENSG00000143819 | ENSG00000100299 |
| 3068 | ENSG00000175591 | ENSG00000170956 |
| 3069 | ENSG00000197846 | ENSG00000151729 |
| 3070 | ENSG00000263136 | ENSG00000107882 |
| 3071 | ENSG00000149357 | ENSG00000204709 |
| 3072 | ENSG00000115232 | ENSG00000068308 |
| 3073 | ENSG00000198435 | ENSG00000135631 |
| 3074 | ENSG00000172809 | ENSG00000183087 |
| 3075 | ENSG00000183034 | ENSG00000111325 |
| 3076 | ENSG00000181378 | ENSG00000205832 |
| 3077 | ENSG00000177971 | ENSG00000131126 |
| 3078 | ENSG00000183337 | ENSG00000213029 |
| 3079 | ENSG00000032444 | ENSG00000105373 |
| 3080 | ENSG00000174500 | ENSG00000076650 |
| 3081 | ENSG00000178078 | ENSG00000162341 |
| 3082 | ENSG00000163645 | ENSG00000136929 |
| 3083 | ENSG00000169660 | ENSG00000158480 |
| 3084 | ENSG00000105613 | ENSG00000205126 |
| 3085 | ENSG00000120333 | ENSG00000124253 |
| 3086 | ENSG00000116539 | ENSG00000182472 |
| 3087 | ENSG00000075043 | ENSG00000161036 |
| 3088 | ENSG00000143162 | ENSG00000002834 |
| 3089 | ENSG00000095319 | ENSG00000106683 |
| 3090 | ENSG00000197768 | ENSG00000187239 |
| 3091 | ENSG00000137731 | ENSG00000186501 |
| 3092 | ENSG00000130311 | ENSG00000119139 |
| 3093 | ENSG00000258256 | ENSG00000130731 |
| 3094 | ENSG00000181061 | ENSG00000160679 |
| 3095 | ENSG00000117650 | ENSG00000129422 |
| 3096 | ENSG00000057019 | ENSG00000133030 |
| 3097 | ENSG00000160284 | ENSG00000164338 |
| 3098 | ENSG00000117362 | ENSG00000111261 |
| 3099 | ENSG00000011347 | ENSG00000198053 |
| 3100 | ENSG00000138185 | ENSG00000204713 |
| 3101 | ENSG00000235478 | ENSG00000049449 |
| 3102 | ENSG00000166145 | ENSG00000136573 |
| 3103 | ENSG00000139624 | ENSG00000085185 |
| 3104 | ENSG00000006075 | ENSG00000143850 |
| 3105 | ENSG00000145860 | ENSG00000172005 |
| 3106 | ENSG00000004939 | ENSG00000173838 |
| 3107 | ENSG00000127054 | ENSG00000100170 |
| 3108 | ENSG00000100359 | ENSG00000140948 |
| 3109 | ENSG00000036672 | ENSG00000157827 |
| 3110 | ENSG00000041802 | ENSG00000178999 |
| 3111 | ENSG00000259494 | ENSG00000213339 |
| 3112 | ENSG00000164430 | ENSG00000166526 |
| 3113 | ENSG00000136842 | ENSG00000092445 |
| 3114 | ENSG00000147408 | ENSG00000121966 |
| 3115 | ENSG00000155087 | ENSG00000077009 |
| 3116 | ENSG00000151208 | ENSG00000165511 |
| 3117 | ENSG00000105737 | ENSG00000033327 |
| 3118 | ENSG00000123444 | ENSG00000100429 |
| 3119 | ENSG00000050405 | ENSG00000178764 |
| 3120 | ENSG00000230610 | ENSG00000152894 |
| 3121 | ENSG00000175879 | ENSG00000130313 |
| 3122 | ENSG00000146278 | ENSG00000177673 |
| 3123 | ENSG00000105171 | ENSG00000104899 |
| 3124 | ENSG00000111596 | ENSG00000175463 |
| 3125 | ENSG00000188706 | ENSG00000167765 |
| 3126 | ENSG00000119414 | ENSG00000169499 |
| 3127 | ENSG00000142178 | ENSG00000095203 |
| 3128 | ENSG00000100258 | ENSG00000123415 |
| 3129 | ENSG00000166347 | ENSG00000023041 |
| 3130 | ENSG00000148341 | ENSG00000104365 |
| 3131 | ENSG00000182704 | ENSG00000159873 |
| 3132 | ENSG00000243509 | ENSG00000141744 |
| 3133 | ENSG00000166532 | ENSG00000093183 |
| 3134 | ENSG00000168653 | ENSG00000064012 |
| 3135 | ENSG00000142082 | ENSG00000157680 |
| 3136 | ENSG00000170807 | ENSG00000139428 |
| 3137 | ENSG00000175354 | ENSG00000183072 |
| 3138 | ENSG00000123933 | ENSG00000028137 |
| 3139 | ENSG00000011275 | ENSG00000085276 |
| 3140 | ENSG00000130703 | ENSG00000087157 |
| 3141 | ENSG00000143418 | ENSG00000118960 |
| 3142 | ENSG00000123407 | ENSG00000183023 |
| 3143 | ENSG00000132622 | ENSG00000125945 |
| 3144 | ENSG00000269048 | ENSG00000069020 |
| 3145 | ENSG00000154252 | ENSG00000106633 |
| 3146 | ENSG00000164821 | ENSG00000172232 |
| 3147 | ENSG00000155657 | ENSG00000163633 |
| 3148 | ENSG00000214026 | ENSG00000150712 |
| 3149 | ENSG00000067191 | ENSG00000118689 |
| 3150 | ENSG00000268194 | ENSG00000118503 |
| 3151 | ENSG00000153234 | ENSG00000120896 |
| 3152 | ENSG00000120068 | ENSG00000158169 |
| 3153 | ENSG00000143630 | ENSG00000159921 |
| 3154 | ENSG00000183638 | ENSG00000100410 |
| 3155 | ENSG00000257127 | ENSG00000108349 |
| 3156 | ENSG00000173805 | ENSG00000057294 |
| 3157 | ENSG00000157782 | ENSG00000197971 |
| 3158 | ENSG00000108639 | ENSG00000011028 |
| 3159 | ENSG00000153179 | ENSG00000182541 |
| 3160 | ENSG00000136114 | ENSG00000162897 |
| 3161 | ENSG00000115998 | ENSG00000120647 |
| 3162 | ENSG00000182224 | ENSG00000176485 |
| 3163 | ENSG00000178209 | ENSG00000112787 |
| 3164 | ENSG00000083799 | ENSG00000099995 |
| 3165 | ENSG00000132740 | ENSG00000161671 |
| 3166 | ENSG00000170310 | ENSG00000169093 |
| 3167 | ENSG00000138674 | ENSG00000151062 |
| 3168 | ENSG00000164694 | ENSG00000129562 |
| 3169 | ENSG00000243989 | ENSG00000110243 |
| 3170 | ENSG00000103160 | ENSG00000128917 |
| 3171 | ENSG00000167522 | ENSG00000142156 |
| 3172 | ENSG00000142207 | ENSG00000267157 |
| 3173 | ENSG00000145945 | ENSG00000181222 |
| 3174 | ENSG00000074527 | ENSG00000224383 |
| 3175 | ENSG00000162572 | ENSG00000269363 |
| 3176 | ENSG00000240972 | ENSG00000090686 |
| 3177 | ENSG00000146112 | ENSG00000100350 |
| 3178 | ENSG00000103264 | ENSG00000196689 |
| 3179 | ENSG00000092758 | ENSG00000065675 |
| 3180 | ENSG00000196517 | ENSG00000127080 |
| 3181 | ENSG00000171914 | ENSG00000170935 |
| 3182 | ENSG00000142609 | ENSG00000122692 |
| 3183 | ENSG00000196754 | ENSG00000213397 |
| 3184 | ENSG00000268085 | ENSG00000168310 |
| 3185 | ENSG00000105855 | ENSG00000133226 |
| 3186 | ENSG00000187997 | ENSG00000064195 |
| 3187 | ENSG00000204839 | ENSG00000113575 |
| 3188 | ENSG00000269308 | ENSG00000153707 |
| 3189 | ENSG00000176903 | ENSG00000100417 |
| 3190 | ENSG00000130449 | ENSG00000077522 |
| 3191 | ENSG00000155097 | ENSG00000130528 |
| 3192 | ENSG00000237452 | ENSG00000268028 |
| 3193 | ENSG00000188542 | ENSG00000153250 |
| 3194 | ENSG00000068971 | ENSG00000160221 |
| 3195 | ENSG00000187189 | ENSG00000188293 |
| 3196 | ENSG00000039068 | ENSG00000132002 |
| 3197 | ENSG00000179388 | ENSG00000187990 |
| 3198 | ENSG00000140479 | ENSG00000183154 |
| 3199 | ENSG00000109016 | ENSG00000130513 |
| 3200 | ENSG00000174327 | ENSG00000268975 |
| 3201 | ENSG00000272896 | ENSG00000071859 |
| 3202 | ENSG00000263155 | ENSG00000101751 |
| 3203 | ENSG00000125388 | ENSG00000116774 |
| 3204 | ENSG00000181350 | ENSG00000157734 |
| 3205 | ENSG00000141627 | ENSG00000203867 |
| 3206 | ENSG00000137801 | ENSG00000250644 |
| 3207 | ENSG00000148175 | ENSG00000143537 |
| 3208 | ENSG00000140986 | ENSG00000211445 |
| 3209 | ENSG00000167280 | ENSG00000131759 |
| 3210 | ENSG00000186407 | ENSG00000164363 |
| 3211 | ENSG00000198369 | ENSG00000205821 |
| 3212 | ENSG00000179399 | ENSG00000177731 |
| 3213 | ENSG00000137817 | ENSG00000175946 |
| 3214 | ENSG00000173281 | ENSG00000115468 |
| 3215 | ENSG00000175279 | ENSG00000125826 |
| 3216 | ENSG00000204520 | ENSG00000203685 |
| 3217 | ENSG00000150201 | ENSG00000147065 |
| 3218 | ENSG00000129910 | ENSG00000105011 |
| 3219 | ENSG00000103591 | ENSG00000171928 |
| 3220 | ENSG00000122711 | ENSG00000116685 |
| 3221 | ENSG00000134200 | ENSG00000116584 |
| 3222 | ENSG00000174136 | ENSG00000148356 |
| 3223 | ENSG00000206172 | ENSG00000188710 |
| 3224 | ENSG00000161609 | ENSG00000074181 |
| 3225 | ENSG00000266964 | ENSG00000173349 |
| 3226 | ENSG00000149177 | ENSG00000159388 |
| 3227 | ENSG00000178852 | ENSG00000106012 |
| 3228 | ENSG00000168461 | ENSG00000126351 |
| 3229 | ENSG00000243696 | ENSG00000100324 |
| 3230 | ENSG00000173264 | ENSG00000164638 |
| 3231 | ENSG00000137841 | ENSG00000131771 |
| 3232 | ENSG00000129675 | ENSG00000118156 |
| 3233 | ENSG00000061455 | ENSG00000197935 |
| 3234 | ENSG00000215182 | ENSG00000151692 |
| 3235 | ENSG00000140993 | ENSG00000229086 |
| 3236 | ENSG00000162004 | ENSG00000140464 |
| 3237 | ENSG00000171298 | ENSG00000103994 |
| 3238 | ENSG00000267261 | ENSG00000258674 |
| 3239 | ENSG00000152766 | ENSG00000143575 |
| 3240 | ENSG00000162298 | ENSG00000167987 |
| 3241 | ENSG00000175564 | ENSG00000105497 |
| 3242 | ENSG00000134668 | ENSG00000140254 |
| 3243 | ENSG00000267906 | ENSG00000248487 |
| 3244 | ENSG00000124749 | ENSG00000178257 |
| 3245 | ENSG00000149596 | ENSG00000084444 |
| 3246 | ENSG00000153774 | ENSG00000099910 |
| 3247 | ENSG00000258947 | ENSG00000187091 |
| 3248 | ENSG00000121764 | ENSG00000130939 |
| 3249 | ENSG00000092929 | ENSG00000134900 |
| 3250 | ENSG00000171408 | ENSG00000134030 |
| 3251 | ENSG00000268856 | ENSG00000014138 |
| 3252 | ENSG00000138068 | ENSG00000197375 |
| 3253 | ENSG00000243789 | ENSG00000084733 |
| 3254 | ENSG00000185736 | ENSG00000259571 |
| 3255 | ENSG00000140543 | ENSG00000104805 |
| 3256 | ENSG00000197561 | ENSG00000026508 |
| 3257 | ENSG00000124578 | ENSG00000182170 |
| 3258 | ENSG00000114388 | ENSG00000163453 |
| 3259 | ENSG00000151650 | ENSG00000161203 |
| 3260 | ENSG00000100316 | ENSG00000182459 |
| 3261 | ENSG00000169203 | ENSG00000204580 |
| 3262 | ENSG00000160087 | ENSG00000172986 |
| 3263 | ENSG00000138061 | ENSG00000031698 |
| 3264 | ENSG00000255054 | ENSG00000020256 |
| 3265 | ENSG00000222028 | ENSG00000187242 |
| 3266 | ENSG00000112343 | ENSG00000255529 |
| 3267 | ENSG00000158055 | ENSG00000178685 |
| 3268 | ENSG00000132677 | ENSG00000132563 |
| 3269 | ENSG00000171475 | ENSG00000082781 |
| 3270 | ENSG00000071082 | ENSG00000254726 |
| 3271 | ENSG00000266714 | ENSG00000107438 |
| 3272 | ENSG00000198700 | ENSG00000003400 |
| 3273 | ENSG00000155744 | ENSG00000168255 |
| 3274 | ENSG00000221845 | ENSG00000159461 |
| 3275 | ENSG00000198807 | ENSG00000140092 |
| 3276 | ENSG00000140274 | ENSG00000139515 |
| 3277 | ENSG00000189377 | ENSG00000161547 |
| 3278 | ENSG00000175646 | ENSG00000198445 |
| 3279 | ENSG00000188910 | ENSG00000126432 |
| 3280 | ENSG00000049130 | ENSG00000080561 |
| 3281 | ENSG00000151665 | ENSG00000068137 |
| 3282 | ENSG00000109065 | ENSG00000107742 |
| 3283 | ENSG00000149115 | ENSG00000107242 |
| 3284 | ENSG00000099194 | ENSG00000151503 |
| 3285 | ENSG00000112799 | ENSG00000169876 |
| 3286 | ENSG00000205364 | ENSG00000105722 |
| 3287 | ENSG00000141258 | ENSG00000179292 |
| 3288 | ENSG00000140718 | ENSG00000175283 |
| 3289 | ENSG00000188846 | ENSG00000132681 |
| 3290 | ENSG00000187605 | ENSG00000214944 |
| 3291 | ENSG00000130876 | ENSG00000119681 |
| 3292 | ENSG00000168071 | ENSG00000184524 |
| 3293 | ENSG00000177143 | ENSG00000158747 |
| 3294 | ENSG00000132182 | ENSG00000105974 |
| 3295 | ENSG00000170606 | ENSG00000167186 |
| 3296 | ENSG00000188976 | ENSG00000221963 |
| 3297 | ENSG00000169621 | ENSG00000160181 |
| 3298 | ENSG00000182326 | ENSG00000086696 |
| 3299 | ENSG00000160613 | ENSG00000133789 |
| 3300 | ENSG00000168631 | ENSG00000116266 |
| 3301 | ENSG00000166086 | ENSG00000250317 |
| 3302 | ENSG00000141543 | ENSG00000249624 |
| 3303 | ENSG00000176595 | ENSG00000141524 |
| 3304 | ENSG00000179134 | ENSG00000254959 |
| 3305 | ENSG00000181638 | ENSG00000100079 |
| 3306 | ENSG00000105875 | ENSG00000184144 |
| 3307 | ENSG00000175087 | ENSG00000101331 |
| 3308 | ENSG00000157617 | ENSG00000075426 |
| 3309 | ENSG00000198795 | ENSG00000141837 |
| 3310 | ENSG00000198711 | ENSG00000066084 |
| 3311 | ENSG00000172348 | ENSG00000130940 |
| 3312 | ENSG00000269804 | ENSG00000163959 |
| 3313 | ENSG00000181038 | ENSG00000206560 |
| 3314 | ENSG00000228120 | ENSG00000155868 |
| 3315 | ENSG00000171262 | ENSG00000187479 |
| 3316 | ENSG00000204070 | ENSG00000205923 |
| 3317 | ENSG00000130675 | ENSG00000145882 |
| 3318 | ENSG00000244274 | ENSG00000135766 |
| 3319 | ENSG00000070501 | ENSG00000115523 |
| 3320 | ENSG00000255804 | ENSG00000065308 |
| 3321 | ENSG00000105325 | ENSG00000012061 |
| 3322 | ENSG00000115297 | ENSG00000099204 |
| 3323 | ENSG00000060762 | ENSG00000149260 |
| 3324 | ENSG00000140577 | ENSG00000114349 |
| 3325 | ENSG00000173991 | ENSG00000144485 |
| 3326 | ENSG00000157613 | ENSG00000131711 |
| 3327 | ENSG00000170775 | ENSG00000268173 |
| 3328 | ENSG00000204257 | ENSG00000136141 |
| 3329 | ENSG00000130589 | ENSG00000196378 |
| 3330 | ENSG00000257108 | ENSG00000152127 |
| 3331 | ENSG00000183778 | ENSG00000120937 |
| 3332 | ENSG00000164292 | ENSG00000103089 |
| 3333 | ENSG00000149136 | ENSG00000164530 |
| 3334 | ENSG00000053438 | ENSG00000164880 |
| 3335 | ENSG00000177791 | ENSG00000183864 |
| 3336 | ENSG00000197576 | ENSG00000146858 |
| 3337 | ENSG00000123243 | ENSG00000120616 |
| 3338 | ENSG00000123171 | ENSG00000196924 |
| 3339 | ENSG00000188112 | ENSG00000166483 |
| 3340 | ENSG00000120093 | ENSG00000130590 |
| 3341 | ENSG00000133895 | ENSG00000159023 |
| 3342 | ENSG00000166402 | ENSG00000196498 |
| 3343 | ENSG00000184471 | ENSG00000162009 |
| 3344 | ENSG00000175938 | ENSG00000164051 |
| 3345 | ENSG00000177427 | ENSG00000225485 |
| 3346 | ENSG00000121440 | ENSG00000182950 |
| 3347 | ENSG00000196642 | ENSG00000267855 |
| 3348 | ENSG00000270099 | ENSG00000187866 |
| 3349 | ENSG00000104763 | ENSG00000138835 |
| 3350 | ENSG00000144785 | ENSG00000177103 |
| 3351 | ENSG00000125538 | ENSG00000172183 |
| 3352 | ENSG00000116221 | ENSG00000100225 |
| 3353 | ENSG00000074047 | ENSG00000177556 |
| 3354 | ENSG00000250571 | ENSG00000112559 |
| 3355 | ENSG00000107902 | ENSG00000156453 |
| 3356 | ENSG00000170703 | ENSG00000105258 |
| 3357 | ENSG00000082701 | ENSG00000171729 |
| 3358 | ENSG00000139496 | ENSG00000100441 |
| 3359 | ENSG00000198315 | ENSG00000163950 |
| 3360 | ENSG00000087258 | ENSG00000170558 |
| 3361 | ENSG00000068724 | ENSG00000186106 |
| 3362 | ENSG00000161544 | ENSG00000175505 |
| 3363 | ENSG00000105617 | ENSG00000138613 |
| 3364 | ENSG00000143324 | ENSG00000103197 |
| 3365 | ENSG00000103257 | ENSG00000169871 |
| 3366 | ENSG00000148541 | ENSG00000176155 |
| 3367 | ENSG00000126456 | ENSG00000198198 |
| 3368 | ENSG00000174007 | ENSG00000066136 |
| 3369 | ENSG00000178722 | ENSG00000167100 |
| 3370 | ENSG00000065534 | ENSG00000108819 |
| 3371 | ENSG00000118197 | ENSG00000166529 |
| 3372 | ENSG00000172938 | ENSG00000108106 |
| 3373 | ENSG00000103275 | ENSG00000173366 |
| 3374 | ENSG00000269514 | ENSG00000205426 |
| 3375 | ENSG00000077809 | ENSG00000172890 |
| 3376 | ENSG00000215790 | ENSG00000183888 |
| 3377 | ENSG00000108511 | ENSG00000008323 |
| 3378 | ENSG00000035664 | ENSG00000150756 |
| 3379 | ENSG00000131018 | ENSG00000149091 |
| 3380 | ENSG00000155893 | ENSG00000198169 |
| 3381 | ENSG00000102359 | ENSG00000160216 |
| 3382 | ENSG00000206069 | ENSG00000125618 |
| 3383 | ENSG00000196476 | ENSG00000160789 |
| 3384 | ENSG00000166126 | ENSG00000137312 |
| 3385 | ENSG00000131351 | ENSG00000064601 |
| 3386 | ENSG00000148158 | ENSG00000268484 |
| 3387 | ENSG00000214022 | ENSG00000268656 |
| 3388 | ENSG00000183495 | ENSG00000168014 |
| 3389 | ENSG00000091664 | ENSG00000126217 |
| 3390 | ENSG00000181218 | ENSG00000131748 |
| 3391 | ENSG00000115307 | ENSG00000127616 |
| 3392 | ENSG00000167580 | ENSG00000255245 |
| 3393 | ENSG00000181656 | ENSG00000163251 |
| 3394 | ENSG00000171595 | ENSG00000203667 |
| 3395 | ENSG00000240065 | ENSG00000179930 |
| 3396 | ENSG00000174038 | ENSG00000008128 |
| 3397 | ENSG00000197757 | ENSG00000181039 |
| 3398 | ENSG00000140682 | ENSG00000086062 |
| 3399 | ENSG00000064102 | ENSG00000067369 |
| 3400 | ENSG00000141401 | ENSG00000124171 |
| 3401 | ENSG00000167702 | ENSG00000135407 |
| 3402 | ENSG00000126266 | ENSG00000145907 |
| 3403 | ENSG00000254788 | ENSG00000205791 |
| 3404 | ENSG00000105993 | ENSG00000106565 |
| 3405 | ENSG00000237847 | ENSG00000196557 |
| 3406 | ENSG00000167566 | ENSG00000155265 |
| 3407 | ENSG00000145075 | ENSG00000204950 |
| 3408 | ENSG00000074071 | ENSG00000249087 |
| 3409 | ENSG00000134326 | ENSG00000171302 |
| 3410 | ENSG00000186532 | ENSG00000180929 |
| 3411 | ENSG00000139219 | ENSG00000198355 |
| 3412 | ENSG00000080618 | ENSG00000124659 |
| 3413 | ENSG00000100288 | ENSG00000124839 |
| 3414 | ENSG00000183570 | ENSG00000117118 |
| 3415 | ENSG00000167395 | ENSG00000133069 |
| 3416 | ENSG00000125430 | ENSG00000076924 |
| 3417 | ENSG00000123908 | ENSG00000164850 |
| 3418 | ENSG00000173391 | ENSG00000161714 |
| 3419 | ENSG00000064703 | ENSG00000173641 |
| 3420 | ENSG00000112902 | ENSG00000162241 |
| 3421 | ENSG00000205810 | ENSG00000126709 |
| 3422 | ENSG00000111199 | ENSG00000134020 |
| 3423 | ENSG00000174804 | ENSG00000172534 |
| 3424 | ENSG00000168679 | ENSG00000261459 |
| 3425 | ENSG00000186635 | ENSG00000172346 |
| 3426 | ENSG00000137691 | ENSG00000139083 |
| 3427 | ENSG00000150337 | ENSG00000132688 |
| 3428 | ENSG00000166199 | ENSG00000105552 |
| 3429 | ENSG00000130561 | ENSG00000119559 |
| 3430 | ENSG00000186994 | ENSG00000214844 |
| 3431 | ENSG00000167770 | ENSG00000146063 |
| 3432 | ENSG00000197322 | ENSG00000166925 |
| 3433 | ENSG00000131876 | ENSG00000075275 |
| 3434 | ENSG00000141026 | ENSG00000068650 |
| 3435 | ENSG00000173976 | ENSG00000268146 |
| 3436 | ENSG00000104221 | ENSG00000142623 |
| 3437 | ENSG00000130725 | ENSG00000206262 |
| 3438 | ENSG00000198454 | ENSG00000035141 |
| 3439 | ENSG00000161960 | ENSG00000177700 |
| 3440 | ENSG00000005156 | ENSG00000168291 |
| 3441 | ENSG00000257184 | ENSG00000164897 |
| 3442 | ENSG00000107036 | ENSG00000134262 |
| 3443 | ENSG00000168101 | ENSG00000162426 |
| 3444 | ENSG00000185436 | ENSG00000072310 |
| 3445 | ENSG00000203896 | ENSG00000171931 |
| 3446 | ENSG00000187583 | ENSG00000251258 |
| 3447 | ENSG00000119801 | ENSG00000183598 |
| 3448 | ENSG00000100297 | ENSG00000179862 |
| 3449 | ENSG00000137962 | ENSG00000182218 |
| 3450 | ENSG00000110881 | ENSG00000186007 |
| 3451 | ENSG00000178252 | ENSG00000071462 |
| 3452 | ENSG00000204618 | ENSG00000103426 |
| 3453 | ENSG00000156886 | ENSG00000187824 |
| 3454 | ENSG00000175318 | ENSG00000064932 |
| 3455 | ENSG00000187772 | ENSG00000084090 |
| 3456 | ENSG00000144579 | ENSG00000196576 |
| 3457 | ENSG00000162383 | ENSG00000136490 |
| 3458 | ENSG00000198517 | ENSG00000206190 |
| 3459 | ENSG00000267964 | ENSG00000010295 |
| 3460 | ENSG00000042980 | ENSG00000206260 |
| 3461 | ENSG00000177721 | ENSG00000143590 |
| 3462 | ENSG00000134333 | ENSG00000110848 |
| 3463 | ENSG00000103966 | ENSG00000181045 |
| 3464 | ENSG00000248540 | ENSG00000126218 |
| 3465 | ENSG00000175093 | ENSG00000186468 |
| 3466 | ENSG00000126460 | ENSG00000183576 |
| 3467 | ENSG00000005961 | ENSG00000163516 |
| 3468 | ENSG00000137106 | ENSG00000104695 |
| 3469 | ENSG00000108389 | ENSG00000143851 |
| 3470 | ENSG00000269407 | ENSG00000157540 |
| 3471 | ENSG00000172828 | ENSG00000156990 |
| 3472 | ENSG00000137076 | ENSG00000156535 |
| 3473 | ENSG00000040531 | ENSG00000180066 |
| 3474 | ENSG00000167799 | ENSG00000179085 |
| 3475 | ENSG00000042813 | ENSG00000109452 |
| 3476 | ENSG00000053747 | ENSG00000158470 |
| 3477 | ENSG00000267913 | ENSG00000185442 |
| 3478 | ENSG00000174417 | ENSG00000065057 |
| 3479 | ENSG00000149131 | ENSG00000065320 |
| 3480 | ENSG00000166164 | ENSG00000133321 |
| 3481 | ENSG00000110448 | ENSG00000166482 |
| 3482 | ENSG00000167194 | ENSG00000133816 |
| 3483 | ENSG00000152700 | ENSG00000196167 |
| 3484 | ENSG00000091136 | ENSG00000167617 |
| 3485 | ENSG00000113369 | ENSG00000171861 |
| 3486 | ENSG00000139505 | ENSG00000165898 |
| 3487 | ENSG00000196821 | ENSG00000167157 |
| 3488 | ENSG00000101193 | ENSG00000108528 |
| 3489 | ENSG00000066926 | ENSG00000018625 |
| 3490 | ENSG00000112659 | ENSG00000135842 |
| 3491 | ENSG00000262246 | ENSG00000095752 |
| 3492 | ENSG00000175911 | ENSG00000121073 |
| 3493 | ENSG00000176907 | ENSG00000154945 |
| 3494 | ENSG00000179813 | ENSG00000205022 |
| 3495 | ENSG00000142534 | ENSG00000150593 |
| 3496 | ENSG00000162227 | ENSG00000117335 |
| 3497 | ENSG00000120265 | ENSG00000178951 |
| 3498 | ENSG00000105516 | ENSG00000182749 |
| 3499 | ENSG00000156076 | ENSG00000187475 |
| 3500 | ENSG00000149948 | ENSG00000188372 |
| 3501 | ENSG00000166562 | ENSG00000205076 |
| 3502 | ENSG00000156127 | ENSG00000107341 |
| 3503 | ENSG00000090975 | ENSG00000160214 |
| 3504 | ENSG00000149532 | ENSG00000165271 |
| 3505 | ENSG00000134109 | ENSG00000101138 |
| 3506 | ENSG00000248993 | ENSG00000068078 |
| 3507 | ENSG00000100150 | ENSG00000095383 |
| 3508 | ENSG00000141252 | ENSG00000110237 |
| 3509 | ENSG00000103522 | ENSG00000164867 |
| 3510 | ENSG00000127903 | ENSG00000175711 |
| 3511 | ENSG00000155363 | ENSG00000233276 |
| 3512 | ENSG00000081692 | ENSG00000125534 |
| 3513 | ENSG00000156931 | ENSG00000107554 |
| 3514 | ENSG00000268470 | ENSG00000117408 |
| 3515 | ENSG00000143373 | ENSG00000138172 |
| 3516 | ENSG00000267848 | ENSG00000168016 |
| 3517 | ENSG00000185432 | ENSG00000205323 |
| 3518 | ENSG00000250799 | ENSG00000116497 |
| 3519 | ENSG00000104872 | ENSG00000187860 |
| 3520 | ENSG00000131508 | ENSG00000109321 |
| 3521 | ENSG00000148384 | ENSG00000131473 |
| 3522 | ENSG00000196116 | ENSG00000102981 |
| 3523 | ENSG00000136450 | ENSG00000117410 |
| 3524 | ENSG00000142599 | ENSG00000079974 |
| 3525 | ENSG00000157103 | ENSG00000146540 |
| 3526 | ENSG00000158473 | ENSG00000188130 |
| 3527 | ENSG00000167618 | ENSG00000105655 |
| 3528 | ENSG00000010278 | ENSG00000141994 |
| 3529 | ENSG00000167103 | ENSG00000178279 |
| 3530 | ENSG00000164520 | ENSG00000198663 |
| 3531 | ENSG00000141485 | ENSG00000031081 |
| 3532 | ENSG00000203286 | ENSG00000108799 |
| 3533 | ENSG00000196683 | ENSG00000167774 |
| 3534 | ENSG00000165731 | ENSG00000136816 |
| 3535 | ENSG00000196427 | ENSG00000111012 |
| 3536 | ENSG00000169047 | ENSG00000174231 |
| 3537 | ENSG00000104808 | ENSG00000175215 |
| 3538 | ENSG00000066294 | ENSG00000183479 |
| 3539 | ENSG00000182952 | ENSG00000177606 |
| 3540 | ENSG00000136463 | ENSG00000147862 |
| 3541 | ENSG00000100162 | ENSG00000184828 |
| 3542 | ENSG00000030419 | ENSG00000038382 |
| 3543 | ENSG00000106399 | ENSG00000122565 |
| 3544 | ENSG00000258512 | ENSG00000162236 |
| 3545 | ENSG00000169905 | ENSG00000118004 |
| 3546 | ENSG00000143627 | ENSG00000161677 |
| 3547 | ENSG00000110777 | ENSG00000105321 |
| 3548 | ENSG00000166558 | ENSG00000254858 |
| 3549 | ENSG00000146809 | ENSG00000168096 |
| 3550 | ENSG00000100567 | ENSG00000136689 |
| 3551 | ENSG00000143321 | ENSG00000133067 |
| 3552 | ENSG00000242114 | ENSG00000154511 |
| 3553 | ENSG00000254019 | ENSG00000227184 |
| 3554 | ENSG00000165490 | ENSG00000152229 |
| 3555 | ENSG00000143995 | ENSG00000089057 |
| 3556 | ENSG00000119335 | ENSG00000160294 |
| 3557 | ENSG00000169813 | ENSG00000159289 |
| 3558 | ENSG00000079308 | ENSG00000176463 |
| 3559 | ENSG00000102738 | ENSG00000104889 |
| 3560 | ENSG00000132603 | ENSG00000118046 |
| 3561 | ENSG00000119013 | ENSG00000113742 |
| 3562 | ENSG00000077238 | ENSG00000105229 |
| 3563 | ENSG00000255508 | ENSG00000018280 |
| 3564 | ENSG00000104824 | ENSG00000204653 |
| 3565 | ENSG00000258388 | ENSG00000005075 |
| 3566 | ENSG00000138604 | ENSG00000124299 |
| 3567 | ENSG00000171490 | ENSG00000185332 |
| 3568 | ENSG00000173207 | ENSG00000258839 |
| 3569 | ENSG00000100399 | ENSG00000100412 |
| 3570 | ENSG00000026103 | ENSG00000039523 |
| 3571 | ENSG00000163501 | ENSG00000158428 |
| 3572 | ENSG00000030110 | ENSG00000149187 |
| 3573 | ENSG00000267918 | ENSG00000182500 |
| 3574 | ENSG00000160224 | ENSG00000170835 |
| 3575 | ENSG00000146143 | ENSG00000185379 |
| 3576 | ENSG00000108622 | ENSG00000103489 |
| 3577 | ENSG00000102119 | ENSG00000103227 |
| 3578 | ENSG00000135916 | ENSG00000124615 |
| 3579 | ENSG00000105137 | ENSG00000134108 |
| 3580 | ENSG00000139278 | ENSG00000137936 |
| 3581 | ENSG00000037042 | ENSG00000185883 |
| 3582 | ENSG00000250173 | ENSG00000198920 |
| 3583 | ENSG00000205544 | ENSG00000089820 |
| 3584 | ENSG00000064886 | ENSG00000203697 |
| 3585 | ENSG00000255168 | ENSG00000181652 |
| 3586 | ENSG00000185028 | ENSG00000089902 |
| 3587 | ENSG00000268397 | ENSG00000198218 |
| 3588 | ENSG00000179941 | ENSG00000124493 |
| 3589 | ENSG00000113719 | ENSG00000092148 |
| 3590 | ENSG00000086598 | ENSG00000128487 |
| 3591 | ENSG00000099860 | ENSG00000161179 |
| 3592 | ENSG00000124766 | ENSG00000179588 |
| 3593 | ENSG00000166025 | ENSG00000269650 |
| 3594 | ENSG00000197620 | ENSG00000141219 |
| 3595 | ENSG00000176438 | ENSG00000155093 |
| 3596 | ENSG00000114745 | ENSG00000160584 |
| 3597 | ENSG00000162244 | ENSG00000189403 |
| 3598 | ENSG00000167861 | ENSG00000155749 |
| 3599 | ENSG00000168209 | ENSG00000184933 |
| 3600 | ENSG00000105255 | ENSG00000170075 |
| 3601 | ENSG00000169241 | ENSG00000010282 |
| 3602 | ENSG00000130303 | ENSG00000143614 |
| 3603 | ENSG00000243156 | ENSG00000185404 |
| 3604 | ENSG00000240849 | ENSG00000183150 |
| 3605 | ENSG00000090857 | ENSG00000111859 |
| 3606 | ENSG00000047365 | ENSG00000163075 |
| 3607 | ENSG00000125522 | ENSG00000157483 |
| 3608 | ENSG00000115661 | ENSG00000235568 |
| 3609 | ENSG00000126583 | ENSG00000143977 |
| 3610 | ENSG00000100023 | ENSG00000127423 |
| 3611 | ENSG00000131037 | ENSG00000174871 |
| 3612 | ENSG00000136931 | ENSG00000174669 |
| 3613 | ENSG00000104388 | ENSG00000110876 |
| 3614 | ENSG00000206527 | ENSG00000160396 |
| 3615 | ENSG00000267942 | ENSG00000106236 |
| 3616 | ENSG00000183653 | ENSG00000074964 |
| 3617 | ENSG00000109625 | ENSG00000214556 |
| 3618 | ENSG00000089486 | ENSG00000117597 |
| 3619 | ENSG00000145217 | ENSG00000111640 |
| 3620 | ENSG00000096093 | ENSG00000181788 |
| 3621 | ENSG00000160058 | ENSG00000171552 |
| 3622 | ENSG00000001460 | ENSG00000158604 |
| 3623 | ENSG00000168827 | ENSG00000126705 |
| 3624 | ENSG00000269422 | ENSG00000163913 |
| 3625 | ENSG00000139370 | ENSG00000042493 |
| 3626 | ENSG00000047346 | ENSG00000174083 |
| 3627 | ENSG00000197956 | ENSG00000007866 |
| 3628 | ENSG00000169429 | ENSG00000130368 |
| 3629 | ENSG00000165630 | ENSG00000185022 |
| 3630 | ENSG00000170486 | ENSG00000141580 |
| 3631 | ENSG00000197728 | ENSG00000235878 |
| 3632 | ENSG00000122644 | ENSG00000169908 |
| 3633 | ENSG00000179902 | ENSG00000010438 |
| 3634 | ENSG00000162552 | ENSG00000185798 |
| 3635 | ENSG00000105357 | ENSG00000164323 |
| 3636 | ENSG00000266958 | ENSG00000154319 |
| 3637 | ENSG00000136938 | ENSG00000087237 |
| 3638 | ENSG00000128000 | ENSG00000184985 |
| 3639 | ENSG00000088356 | ENSG00000129226 |
| 3640 | ENSG00000115275 | ENSG00000141232 |
| 3641 | ENSG00000146676 | ENSG00000187498 |
| 3642 | ENSG00000105810 | ENSG00000137497 |
| 3643 | ENSG00000155367 | ENSG00000126106 |
| 3644 | ENSG00000174775 | ENSG00000113073 |
| 3645 | ENSG00000111913 | ENSG00000110108 |
| 3646 | ENSG00000170613 | ENSG00000257704 |
| 3647 | ENSG00000171864 | ENSG00000103707 |
| 3648 | ENSG00000160404 | ENSG00000169692 |
| 3649 | ENSG00000143412 | ENSG00000168517 |
| 3650 | ENSG00000082269 | ENSG00000128016 |
| 3651 | ENSG00000127954 | ENSG00000103502 |
| 3652 | ENSG00000178719 | ENSG00000185499 |
| 3653 | ENSG00000163157 | ENSG00000126749 |
| 3654 | ENSG00000174990 | ENSG00000140505 |
| 3655 | ENSG00000196659 | ENSG00000102974 |
| 3656 | ENSG00000080293 | ENSG00000187783 |
| 3657 | ENSG00000179855 | ENSG00000187735 |
| 3658 | ENSG00000204140 | ENSG00000212123 |
| 3659 | ENSG00000119457 | ENSG00000111678 |
| 3660 | ENSG00000148985 | ENSG00000119431 |
| 3661 | ENSG00000006704 | ENSG00000182196 |
| 3662 | ENSG00000176533 | ENSG00000176490 |
| 3663 | ENSG00000176542 | ENSG00000143624 |
| 3664 | ENSG00000131470 | ENSG00000072134 |
| 3665 | ENSG00000118257 | ENSG00000170525 |
| 3666 | ENSG00000266086 | ENSG00000091542 |
| 3667 | ENSG00000131094 | ENSG00000101421 |
| 3668 | ENSG00000106006 | ENSG00000099812 |
| 3669 | ENSG00000129933 | ENSG00000067082 |
| 3670 | ENSG00000136448 | ENSG00000183421 |
| 3671 | ENSG00000134504 | ENSG00000171989 |
| 3672 | ENSG00000101443 | ENSG00000188529 |
| 3673 | ENSG00000005189 | ENSG00000157303 |
| 3674 | ENSG00000168260 | ENSG00000008513 |
| 3675 | ENSG00000110063 | ENSG00000197019 |
| 3676 | ENSG00000077348 | ENSG00000116586 |
| 3677 | ENSG00000135823 | ENSG00000147465 |
| 3678 | ENSG00000108786 | ENSG00000114698 |
| 3679 | ENSG00000182885 | ENSG00000142192 |
| 3680 | ENSG00000213445 | ENSG00000116478 |
| 3681 | ENSG00000111269 | ENSG00000168476 |
| 3682 | ENSG00000063177 | ENSG00000228919 |
| 3683 | ENSG00000124383 | ENSG00000158615 |
| 3684 | ENSG00000232671 | ENSG00000159224 |
| 3685 | ENSG00000083807 | ENSG00000147459 |
| 3686 | ENSG00000111696 | ENSG00000180535 |
| 3687 | ENSG00000181090 | ENSG00000143776 |
| 3688 | ENSG00000184860 | ENSG00000177479 |
| 3689 | ENSG00000078795 | ENSG00000104814 |
| 3690 | ENSG00000128340 | ENSG00000114268 |
| 3691 | ENSG00000126368 | ENSG00000128585 |
| 3692 | ENSG00000196415 | ENSG00000177455 |
| 3693 | ENSG00000123119 | ENSG00000111679 |
| 3694 | ENSG00000132359 | ENSG00000267882 |
| 3695 | ENSG00000185928 | ENSG00000232056 |
| 3696 | ENSG00000130755 | ENSG00000100003 |
| 3697 | ENSG00000103018 | ENSG00000081237 |
| 3698 | ENSG00000140280 | ENSG00000189143 |
| 3699 | ENSG00000181965 | ENSG00000064309 |
| 3700 | ENSG00000167178 | ENSG00000076067 |
| 3701 | ENSG00000269035 | ENSG00000110492 |
| 3702 | ENSG00000143398 | ENSG00000116016 |
| 3703 | ENSG00000113583 | ENSG00000175029 |
| 3704 | ENSG00000125812 | ENSG00000235268 |
| 3705 | ENSG00000186847 | ENSG00000120913 |
| 3706 | ENSG00000164399 | ENSG00000172262 |
| 3707 | ENSG00000132549 | ENSG00000127527 |
| 3708 | ENSG00000126215 | ENSG00000198353 |
| 3709 | ENSG00000163714 | ENSG00000116903 |
| 3710 | ENSG00000163508 | ENSG00000204852 |
| 3711 | ENSG00000123552 | ENSG00000029725 |
| 3712 | ENSG00000198758 | ENSG00000146477 |
| 3713 | ENSG00000091536 | ENSG00000114923 |
| 3714 | ENSG00000180573 | ENSG00000183558 |
| 3715 | ENSG00000178980 | ENSG00000267881 |
| 3716 | ENSG00000124225 | ENSG00000170803 |
| 3717 | ENSG00000134759 | ENSG00000150750 |
| 3718 | ENSG00000100365 | ENSG00000178096 |
| 3719 | ENSG00000145029 | ENSG00000104915 |
| 3720 | ENSG00000133313 | ENSG00000103196 |
| 3721 | ENSG00000141127 | ENSG00000173692 |
| 3722 | ENSG00000128581 | ENSG00000166816 |
| 3723 | ENSG00000132313 | ENSG00000187840 |
| 3724 | ENSG00000198242 | ENSG00000184436 |
| 3725 | ENSG00000100097 | ENSG00000099949 |
| 3726 | ENSG00000204909 | ENSG00000182898 |
| 3727 | ENSG00000184840 | ENSG00000007314 |
| 3728 | ENSG00000188707 | ENSG00000090971 |
| 3729 | ENSG00000137413 | ENSG00000267710 |
| 3730 | ENSG00000258365 | ENSG00000251537 |
| 3731 | ENSG00000100292 | ENSG00000104886 |
| 3732 | ENSG00000100031 | ENSG00000105197 |
| 3733 | ENSG00000129657 | ENSG00000134184 |
| 3734 | ENSG00000136011 | ENSG00000072694 |
| 3735 | ENSG00000167670 | ENSG00000198356 |
| 3736 | ENSG00000240247 | ENSG00000259003 |
| 3737 | ENSG00000100342 | ENSG00000104897 |
| 3738 | ENSG00000157510 | ENSG00000120889 |
| 3739 | ENSG00000119523 | ENSG00000140443 |
| 3740 | ENSG00000115850 | ENSG00000127481 |
| 3741 | ENSG00000106258 | ENSG00000141741 |
| 3742 | ENSG00000108947 | ENSG00000143556 |
| 3743 | ENSG00000197457 | ENSG00000182552 |
| 3744 | ENSG00000130595 | ENSG00000168591 |
| 3745 | ENSG00000132466 | ENSG00000211456 |
| 3746 | ENSG00000142619 | ENSG00000146221 |
| 3747 | ENSG00000171401 | ENSG00000106261 |
| 3748 | ENSG00000051108 | ENSG00000011332 |
| 3749 | ENSG00000205336 | ENSG00000105438 |
| 3750 | ENSG00000107551 | ENSG00000176261 |
| 3751 | ENSG00000132507 | ENSG00000132768 |
| 3752 | ENSG00000144136 | ENSG00000196338 |
| 3753 | ENSG00000128944 | ENSG00000250506 |
| 3754 | ENSG00000134318 | ENSG00000108039 |
| 3755 | ENSG00000103415 | ENSG00000198842 |
| 3756 | ENSG00000090924 | ENSG00000148346 |
| 3757 | ENSG00000076864 | ENSG00000099889 |
| 3758 | ENSG00000112658 | ENSG00000099942 |
| 3759 | ENSG00000126895 | ENSG00000140575 |
| 3760 | ENSG00000106018 | ENSG00000130584 |
| 3761 | ENSG00000069956 | ENSG00000178104 |
| 3762 | ENSG00000090061 | ENSG00000100348 |
| 3763 | ENSG00000139648 | ENSG00000144029 |
| 3764 | ENSG00000101972 | ENSG00000131462 |
| 3765 | ENSG00000250067 | ENSG00000113657 |
| 3766 | ENSG00000175449 | ENSG00000250424 |
| 3767 | ENSG00000204283 | ENSG00000178921 |
| 3768 | ENSG00000196405 | ENSG00000139629 |
| 3769 | ENSG00000069966 | ENSG00000135074 |
| 3770 | ENSG00000105204 | ENSG00000163485 |
| 3771 | ENSG00000129009 | ENSG00000161558 |
| 3772 | ENSG00000136167 | ENSG00000141854 |
| 3773 | ENSG00000179029 | ENSG00000154359 |
| 3774 | ENSG00000197747 | ENSG00000176407 |
| 3775 | ENSG00000168242 | ENSG00000172493 |
| 3776 | ENSG00000131791 | ENSG00000006194 |
| 3777 | ENSG00000129354 | ENSG00000171813 |
| 3778 | ENSG00000169507 | ENSG00000162909 |
| 3779 | ENSG00000105063 | ENSG00000185585 |
| 3780 | ENSG00000102908 | ENSG00000177000 |
| 3781 | ENSG00000137807 | ENSG00000160180 |
| 3782 | ENSG00000138081 | ENSG00000111271 |
| 3783 | ENSG00000205334 | ENSG00000158042 |
| 3784 | ENSG00000212673 | ENSG00000148411 |
| 3785 | ENSG00000090447 | ENSG00000269808 |
| 3786 | ENSG00000164125 | ENSG00000187135 |
| 3787 | ENSG00000108666 | ENSG00000165525 |
| 3788 | ENSG00000172548 | ENSG00000142046 |
| 3789 | ENSG00000107643 | ENSG00000100815 |
| 3790 | ENSG00000121058 | ENSG00000172992 |
| 3791 | ENSG00000157778 | ENSG00000170145 |
| 3792 | ENSG00000213563 | ENSG00000106948 |
| 3793 | ENSG00000089327 | ENSG00000169955 |
| 3794 | ENSG00000196420 | ENSG00000100605 |
| 3795 | ENSG00000105371 | ENSG00000162892 |
| 3796 | ENSG00000268632 | ENSG00000167136 |
| 3797 | ENSG00000121964 | ENSG00000141736 |
| 3798 | ENSG00000197111 | ENSG00000141503 |
| 3799 | ENSG00000110344 | ENSG00000174950 |
| 3800 | ENSG00000137261 | ENSG00000248871 |
| 3801 | ENSG00000197321 | ENSG00000132334 |
| 3802 | ENSG00000188375 | ENSG00000196372 |
| 3803 | ENSG00000183010 | ENSG00000023330 |
| 3804 | ENSG00000269496 | ENSG00000100065 |
| 3805 | ENSG00000185666 | ENSG00000187627 |
| 3806 | ENSG00000215788 | ENSG00000130208 |
| 3807 | ENSG00000018610 | ENSG00000163874 |
| 3808 | ENSG00000172322 | ENSG00000148737 |
| 3809 | ENSG00000150457 | ENSG00000197798 |
| 3810 | ENSG00000138629 | ENSG00000102100 |
| 3811 | ENSG00000168491 | ENSG00000157601 |
| 3812 | ENSG00000120729 | ENSG00000139832 |
| 3813 | ENSG00000187678 | ENSG00000158373 |
| 3814 | ENSG00000128989 | ENSG00000108107 |
| 3815 | ENSG00000188869 | ENSG00000133056 |
| 3816 | ENSG00000267802 | ENSG00000140932 |
| 3817 | ENSG00000103423 | ENSG00000168175 |
| 3818 | ENSG00000221988 | ENSG00000133243 |
| 3819 | ENSG00000134146 | ENSG00000168124 |
| 3820 | ENSG00000171587 | ENSG00000169180 |
| 3821 | ENSG00000267303 | ENSG00000048471 |
| 3822 | ENSG00000258986 | ENSG00000126003 |
| 3823 | ENSG00000011590 | ENSG00000147684 |
| 3824 | ENSG00000106628 | ENSG00000198894 |
| 3825 | ENSG00000159733 | ENSG00000063169 |
| 3826 | ENSG00000157227 | ENSG00000060237 |
| 3827 | ENSG00000169306 | ENSG00000100351 |
| 3828 | ENSG00000104472 | ENSG00000269182 |
| 3829 | ENSG00000181754 | ENSG00000187185 |
| 3830 | ENSG00000115020 | ENSG00000005882 |
| 3831 | ENSG00000130173 | ENSG00000167257 |
| 3832 | ENSG00000179846 | ENSG00000136950 |
| 3833 | ENSG00000168398 | ENSG00000150995 |
| 3834 | ENSG00000151465 | ENSG00000159173 |
| 3835 | ENSG00000135093 | ENSG00000175110 |
| 3836 | ENSG00000130770 | ENSG00000064687 |
| 3837 | ENSG00000066405 | ENSG00000255974 |
| 3838 | ENSG00000172236 | ENSG00000247596 |
| 3839 | ENSG00000124678 | ENSG00000242689 |
| 3840 | ENSG00000171033 | ENSG00000141756 |
| 3841 | ENSG00000169299 | ENSG00000133985 |
| 3842 | ENSG00000267976 | ENSG00000243646 |
| 3843 | ENSG00000108375 | ENSG00000136810 |
| 3844 | ENSG00000112308 | ENSG00000152661 |
| 3845 | ENSG00000213676 | ENSG00000116815 |
| 3846 | ENSG00000101224 | ENSG00000172331 |
| 3847 | ENSG00000138772 | ENSG00000178397 |
| 3848 | ENSG00000204301 | ENSG00000089163 |
| 3849 | ENSG00000044446 | ENSG00000198873 |
| 3850 | ENSG00000141570 | ENSG00000099624 |
| 3851 | ENSG00000089012 | ENSG00000116688 |
| 3852 | ENSG00000257411 | ENSG00000013523 |
| 3853 | ENSG00000063515 | ENSG00000122863 |
| 3854 | ENSG00000006432 | ENSG00000149485 |
| 3855 | ENSG00000135862 | ENSG00000114626 |
| 3856 | ENSG00000117318 | ENSG00000070444 |
| 3857 | ENSG00000163637 | ENSG00000163872 |
| 3858 | ENSG00000228594 | ENSG00000123405 |
| 3859 | ENSG00000135414 | ENSG00000103034 |
| 3860 | ENSG00000133247 | ENSG00000107593 |
| 3861 | ENSG00000234769 | ENSG00000243364 |
| 3862 | ENSG00000008382 | ENSG00000169682 |
| 3863 | ENSG00000184809 | ENSG00000076513 |
| 3864 | ENSG00000183401 | ENSG00000165548 |
| 3865 | ENSG00000153071 | ENSG00000160991 |
| 3866 | ENSG00000167113 | ENSG00000107130 |
| 3867 | ENSG00000100092 | ENSG00000100201 |
| 3868 | ENSG00000010818 | ENSG00000160685 |
| 3869 | ENSG00000112941 | ENSG00000100228 |
| 3870 | ENSG00000198911 | ENSG00000066468 |
| 3871 | ENSG00000198816 | ENSG00000203392 |
| 3872 | ENSG00000204710 | ENSG00000052795 |
| 3873 | ENSG00000145757 | ENSG00000118785 |
| 3874 | ENSG00000257921 | ENSG00000156413 |
| 3875 | ENSG00000095713 | ENSG00000101413 |
| 3876 | ENSG00000175536 | ENSG00000185811 |
| 3877 | ENSG00000110074 | ENSG00000089775 |
| 3878 | ENSG00000108469 | ENSG00000180957 |
| 3879 | ENSG00000137831 | ENSG00000099998 |
| 3880 | ENSG00000165689 | ENSG00000272916 |
| 3881 | ENSG00000254995 | ENSG00000166546 |
| 3882 | ENSG00000160588 | ENSG00000165752 |
| 3883 | ENSG00000118777 | ENSG00000076242 |
| 3884 | ENSG00000122547 | ENSG00000124882 |
| 3885 | ENSG00000172572 | ENSG00000141012 |
| 3886 | ENSG00000177156 | ENSG00000237190 |
| 3887 | ENSG00000058085 | ENSG00000124098 |
| 3888 | ENSG00000161835 | ENSG00000128973 |
| 3889 | ENSG00000243284 | ENSG00000174456 |
| 3890 | ENSG00000141002 | ENSG00000116990 |
| 3891 | ENSG00000115592 | ENSG00000166295 |
| 3892 | ENSG00000132746 | ENSG00000198488 |
| 3893 | ENSG00000164708 | ENSG00000134321 |
| 3894 | ENSG00000170689 | ENSG00000090432 |
| 3895 | ENSG00000174348 | ENSG00000133265 |
| 3896 | ENSG00000183773 | ENSG00000196431 |
| 3897 | ENSG00000154035 | ENSG00000121680 |
| 3898 | ENSG00000197629 | ENSG00000086848 |
| 3899 | ENSG00000123094 | ENSG00000120949 |
| 3900 | ENSG00000170638 | ENSG00000085982 |
| 3901 | ENSG00000255994 | ENSG00000204564 |
| 3902 | ENSG00000123297 | ENSG00000147573 |
| 3903 | ENSG00000221990 | ENSG00000197697 |
| 3904 | ENSG00000128915 | ENSG00000177854 |
| 3905 | ENSG00000154099 | ENSG00000136840 |
| 3906 | ENSG00000214511 | ENSG00000178802 |
| 3907 | ENSG00000103241 | ENSG00000126243 |
| 3908 | ENSG00000147419 | ENSG00000143319 |
| 3909 | ENSG00000162402 | ENSG00000187268 |
| 3910 | ENSG00000179151 | ENSG00000140022 |
| 3911 | ENSG00000043514 | ENSG00000106992 |
| 3912 | ENSG00000230493 | ENSG00000133316 |
| 3913 | ENSG00000188191 | ENSG00000078668 |
| 3914 | ENSG00000213931 | ENSG00000240505 |
| 3915 | ENSG00000085662 | ENSG00000090470 |
| 3916 | ENSG00000120158 | ENSG00000130479 |
| 3917 | ENSG00000105499 | ENSG00000171119 |
| 3918 | ENSG00000239900 | ENSG00000170412 |
| 3919 | ENSG00000198258 | ENSG00000111664 |
| 3920 | ENSG00000187688 | ENSG00000188493 |
| 3921 | ENSG00000103091 | ENSG00000225614 |
| 3922 | ENSG00000133997 | ENSG00000144230 |
| 3923 | ENSG00000157985 | ENSG00000110031 |
| 3924 | ENSG00000007516 | ENSG00000103642 |
| 3925 | ENSG00000108387 | ENSG00000005513 |
| 3926 | ENSG00000115415 | ENSG00000151914 |
| 3927 | ENSG00000105483 | ENSG00000131149 |
| 3928 | ENSG00000132481 | ENSG00000148218 |
| 3929 | ENSG00000163349 | ENSG00000162882 |
| 3930 | ENSG00000181481 | ENSG00000157837 |
| 3931 | ENSG00000239779 | ENSG00000177425 |
| 3932 | ENSG00000061273 | ENSG00000138814 |
| 3933 | ENSG00000095932 | ENSG00000167780 |
| 3934 | ENSG00000124942 | ENSG00000075240 |
| 3935 | ENSG00000039560 | ENSG00000116288 |
| 3936 | ENSG00000197562 | ENSG00000158246 |
| 3937 | ENSG00000102572 | ENSG00000102034 |
| 3938 | ENSG00000139641 | ENSG00000128039 |
| 3939 | ENSG00000134247 | ENSG00000002933 |
| 3940 | ENSG00000143772 | ENSG00000090621 |
| 3941 | ENSG00000089847 | ENSG00000196670 |
| 3942 | ENSG00000125354 | ENSG00000167881 |
| 3943 | ENSG00000186185 | ENSG00000164591 |
| 3944 | ENSG00000186265 | ENSG00000113108 |
| 3945 | ENSG00000103479 | ENSG00000060709 |
| 3946 | ENSG00000213246 | ENSG00000149798 |
| 3947 | ENSG00000101343 | ENSG00000205595 |
| 3948 | ENSG00000117016 | ENSG00000179218 |
| 3949 | ENSG00000142621 | ENSG00000110048 |
| 3950 | ENSG00000196890 | ENSG00000120075 |
| 3951 | ENSG00000166743 | ENSG00000198691 |
| 3952 | ENSG00000170262 | ENSG00000089505 |
| 3953 | ENSG00000106125 | ENSG00000116984 |
| 3954 | ENSG00000125457 | ENSG00000150401 |
| 3955 | ENSG00000160255 | ENSG00000183153 |
| 3956 | ENSG00000259132 | ENSG00000162878 |
| 3957 | ENSG00000152137 | ENSG00000099308 |
| 3958 | ENSG00000188060 | ENSG00000147437 |
| 3959 | ENSG00000187855 | ENSG00000169435 |
| 3960 | ENSG00000166484 | ENSG00000117528 |
| 3961 | ENSG00000196866 | ENSG00000133216 |
| 3962 | ENSG00000198373 | ENSG00000142632 |
| 3963 | ENSG00000197299 | ENSG00000173825 |
| 3964 | ENSG00000204377 | ENSG00000100302 |
| 3965 | ENSG00000204217 | ENSG00000008226 |
| 3966 | ENSG00000239605 | ENSG00000100312 |
| 3967 | ENSG00000133392 | ENSG00000137094 |
| 3968 | ENSG00000137203 | ENSG00000146834 |
| 3969 | ENSG00000255394 | ENSG00000268797 |
| 3970 | ENSG00000127220 | ENSG00000160783 |
| 3971 | ENSG00000141979 | ENSG00000170634 |
| 3972 | ENSG00000125414 | ENSG00000172831 |
| 3973 | ENSG00000107447 | ENSG00000172824 |
| 3974 | ENSG00000168300 | ENSG00000259458 |
| 3975 | ENSG00000120656 | ENSG00000010292 |
| 3976 | ENSG00000154783 | ENSG00000101152 |
| 3977 | ENSG00000087460 | ENSG00000108021 |
| 3978 | ENSG00000174898 | ENSG00000168785 |
| 3979 | ENSG00000167315 | ENSG00000163531 |
| 3980 | ENSG00000255730 | ENSG00000205238 |
| 3981 | ENSG00000268614 | ENSG00000163694 |
| 3982 | ENSG00000258691 | ENSG00000189319 |
| 3983 | ENSG00000124593 | ENSG00000131747 |
| 3984 | ENSG00000118454 | ENSG00000269510 |
| 3985 | ENSG00000106038 | ENSG00000130529 |
| 3986 | ENSG00000100650 | ENSG00000157823 |
| 3987 | ENSG00000139405 | ENSG00000168575 |
| 3988 | ENSG00000176105 | ENSG00000176984 |
| 3989 | ENSG00000222038 | ENSG00000025293 |
| 3990 | ENSG00000178896 | ENSG00000214360 |
| 3991 | ENSG00000158195 | ENSG00000188761 |
| 3992 | ENSG00000116514 | ENSG00000162889 |
| 3993 | ENSG00000223953 | ENSG00000188566 |
| 3994 | ENSG00000131152 | ENSG00000161992 |
| 3995 | ENSG00000214140 | ENSG00000174527 |
| 3996 | ENSG00000173928 | ENSG00000165716 |
| 3997 | ENSG00000100483 | ENSG00000108298 |
| 3998 | ENSG00000105447 | ENSG00000072071 |
| 3999 | ENSG00000213402 | ENSG00000148331 |
| 4000 | ENSG00000127337 | ENSG00000205784 |
| 4001 | ENSG00000182809 | ENSG00000007350 |
| 4002 | ENSG00000182180 | ENSG00000185630 |
| 4003 | ENSG00000267885 | ENSG00000143382 |
| 4004 | ENSG00000143578 | ENSG00000103528 |
| 4005 | ENSG00000138378 | ENSG00000197681 |
| 4006 | ENSG00000185236 | ENSG00000133317 |
| 4007 | ENSG00000039650 | ENSG00000134375 |
| 4008 | ENSG00000180530 | ENSG00000117289 |
| 4009 | ENSG00000172456 | ENSG00000169925 |
| 4010 | ENSG00000143183 | ENSG00000111644 |
| 4011 | ENSG00000203993 | ENSG00000176177 |
| 4012 | ENSG00000186866 | ENSG00000163884 |
| 4013 | ENSG00000153815 | ENSG00000179262 |
| 4014 | ENSG00000142208 | ENSG00000106631 |
| 4015 | ENSG00000180304 | ENSG00000090097 |
| 4016 | ENSG00000145979 | ENSG00000146648 |
| 4017 | ENSG00000134369 | ENSG00000120899 |
| 4018 | ENSG00000100401 | ENSG00000106785 |
| 4019 | ENSG00000123700 | ENSG00000176393 |
| 4020 | ENSG00000133961 | ENSG00000137404 |
| 4021 | ENSG00000173013 | ENSG00000164855 |
| 4022 | ENSG00000197694 | ENSG00000163346 |
| 4023 | ENSG00000146872 | ENSG00000163479 |
| 4024 | ENSG00000159228 | ENSG00000102802 |
| 4025 | ENSG00000213741 | ENSG00000142748 |
| 4026 | ENSG00000134352 | ENSG00000135077 |
| 4027 | ENSG00000213578 | ENSG00000268310 |
| 4028 | ENSG00000106686 | ENSG00000125868 |
| 4029 | ENSG00000145824 | ENSG00000131400 |
| 4030 | ENSG00000021461 | ENSG00000031823 |
| 4031 | ENSG00000173335 | ENSG00000203814 |
| 4032 | ENSG00000115263 | ENSG00000169715 |
| 4033 | ENSG00000269766 | ENSG00000172889 |
| 4034 | ENSG00000082641 | ENSG00000239857 |
| 4035 | ENSG00000162585 | ENSG00000171532 |
| 4036 | ENSG00000149922 | ENSG00000137878 |
| 4037 | ENSG00000175229 | ENSG00000117407 |
| 4038 | ENSG00000129521 | ENSG00000124762 |
| 4039 | ENSG00000167491 | ENSG00000055118 |
| 4040 | ENSG00000175193 | ENSG00000196781 |
| 4041 | ENSG00000256762 | ENSG00000063660 |
| 4042 | ENSG00000258572 | ENSG00000100239 |
| 4043 | ENSG00000187017 | ENSG00000156502 |
| 4044 | ENSG00000141867 | ENSG00000167775 |
| 4045 | ENSG00000133812 | ENSG00000196391 |
| 4046 | ENSG00000166796 | ENSG00000131620 |
| 4047 | ENSG00000197256 | ENSG00000188211 |
| 4048 | ENSG00000124208 | ENSG00000162521 |
| 4049 | ENSG00000198270 | ENSG00000164284 |
| 4050 | ENSG00000165066 | ENSG00000065621 |
| 4051 | ENSG00000205084 | ENSG00000161973 |
| 4052 | ENSG00000156787 | ENSG00000105662 |
| 4053 | ENSG00000243566 | ENSG00000140526 |
| 4054 | ENSG00000179921 | ENSG00000075223 |
| 4055 | ENSG00000177614 | ENSG00000129351 |
| 4056 | ENSG00000008517 | ENSG00000011376 |
| 4057 | ENSG00000176194 | ENSG00000176101 |
| 4058 | ENSG00000205213 | ENSG00000125968 |
| 4059 | ENSG00000059588 | ENSG00000124006 |
| 4060 | ENSG00000142910 | ENSG00000099260 |
| 4061 | ENSG00000069206 | ENSG00000260869 |
| 4062 | ENSG00000236287 | ENSG00000198959 |
| 4063 | ENSG00000115648 | ENSG00000151693 |
| 4064 | ENSG00000111412 | ENSG00000257008 |
| 4065 | ENSG00000151690 | ENSG00000155275 |
| 4066 | ENSG00000139722 | ENSG00000183853 |
| 4067 | ENSG00000167371 | ENSG00000241839 |
| 4068 | ENSG00000128607 | ENSG00000178093 |
| 4069 | ENSG00000204348 | ENSG00000146094 |
| 4070 | ENSG00000115649 | ENSG00000089356 |
| 4071 | ENSG00000159723 | ENSG00000159640 |
| 4072 | ENSG00000189306 | ENSG00000103174 |
| 4073 | ENSG00000137713 | ENSG00000188282 |
| 4074 | ENSG00000077984 | ENSG00000136854 |
| 4075 | ENSG00000188163 | ENSG00000182909 |
| 4076 | ENSG00000204540 | ENSG00000185215 |
| 4077 | ENSG00000155366 | ENSG00000142188 |
| 4078 | ENSG00000187902 | ENSG00000080854 |
| 4079 | ENSG00000087245 | ENSG00000177954 |
| 4080 | ENSG00000163915 | ENSG00000163249 |
| 4081 | ENSG00000178226 | ENSG00000114315 |
| 4082 | ENSG00000237330 | ENSG00000140795 |
| 4083 | ENSG00000131871 | ENSG00000181322 |
| 4084 | ENSG00000197119 | ENSG00000013563 |
| 4085 | ENSG00000188931 | ENSG00000101306 |
| 4086 | ENSG00000144589 | ENSG00000171497 |
| 4087 | ENSG00000114857 | ENSG00000256655 |
| 4088 | ENSG00000057663 | ENSG00000268423 |
| 4089 | ENSG00000132424 | ENSG00000151067 |
| 4090 | ENSG00000101216 | ENSG00000086544 |
| 4091 | ENSG00000253320 | ENSG00000229833 |
| 4092 | ENSG00000188290 | ENSG00000126934 |
| 4093 | ENSG00000065268 | ENSG00000144040 |
| 4094 | ENSG00000108352 | ENSG00000181773 |
| 4095 | ENSG00000169714 | ENSG00000123427 |
| 4096 | ENSG00000103942 | ENSG00000189334 |
| 4097 | ENSG00000185024 | ENSG00000039987 |
| 4098 | ENSG00000142937 | ENSG00000104142 |
| 4099 | ENSG00000166548 | ENSG00000131795 |
| 4100 | ENSG00000179091 | ENSG00000176884 |
| 4101 | ENSG00000139644 | ENSG00000198771 |
| 4102 | ENSG00000105501 | ENSG00000034053 |
| 4103 | ENSG00000241399 | ENSG00000139514 |
| 4104 | ENSG00000083307 | ENSG00000122642 |
| 4105 | ENSG00000152684 | ENSG00000108826 |
| 4106 | ENSG00000178860 | ENSG00000058404 |
| 4107 | ENSG00000187773 | ENSG00000213782 |
| 4108 | ENSG00000228532 | ENSG00000265096 |
| 4109 | ENSG00000122180 | ENSG00000107819 |
| 4110 | ENSG00000147485 | ENSG00000167074 |
| 4111 | ENSG00000203883 | ENSG00000103494 |
| 4112 | ENSG00000142677 | ENSG00000163833 |
| 4113 | ENSG00000157423 | ENSG00000137166 |
| 4114 | ENSG00000184545 | ENSG00000187010 |
| 4115 | ENSG00000111405 | ENSG00000168874 |
| 4116 | ENSG00000008853 | ENSG00000188015 |
| 4117 | ENSG00000156925 | ENSG00000167105 |
| 4118 | ENSG00000126561 | ENSG00000165171 |
| 4119 | ENSG00000174279 | ENSG00000197540 |
| 4120 | ENSG00000205593 | ENSG00000150867 |
| 4121 | ENSG00000152767 | ENSG00000067842 |
| 4122 | ENSG00000178188 | ENSG00000125741 |
| 4123 | ENSG00000124575 | ENSG00000168539 |
| 4124 | ENSG00000132003 | ENSG00000095066 |
| 4125 | ENSG00000163810 | ENSG00000169604 |
| 4126 | ENSG00000108244 | ENSG00000104881 |
| 4127 | ENSG00000000003 | ENSG00000134851 |
| 4128 | ENSG00000186198 | ENSG00000249209 |
| 4129 | ENSG00000177800 | ENSG00000113296 |
| 4130 | ENSG00000187231 | ENSG00000198055 |
| 4131 | ENSG00000244588 | ENSG00000120910 |
| 4132 | ENSG00000077044 | ENSG00000137409 |
| 4133 | ENSG00000065600 | ENSG00000161328 |
| 4134 | ENSG00000122304 | ENSG00000197363 |
| 4135 | ENSG00000167635 | ENSG00000105427 |
| 4136 | ENSG00000164385 | ENSG00000144021 |
| 4137 | ENSG00000147535 | ENSG00000146700 |
| 4138 | ENSG00000087302 | ENSG00000117525 |
| 4139 | ENSG00000184381 | ENSG00000153113 |
| 4140 | ENSG00000131196 | ENSG00000163348 |
| 4141 | ENSG00000132016 | ENSG00000141098 |
| 4142 | ENSG00000196700 | ENSG00000110934 |
| 4143 | ENSG00000269074 | ENSG00000167815 |
| 4144 | ENSG00000187775 | ENSG00000111424 |
| 4145 | ENSG00000185920 | ENSG00000188937 |
| 4146 | ENSG00000107077 | ENSG00000114735 |
| 4147 | ENSG00000136104 | ENSG00000260027 |
| 4148 | ENSG00000163958 | ENSG00000103742 |
| 4149 | ENSG00000225683 | ENSG00000134324 |
| 4150 | ENSG00000005001 | ENSG00000212710 |
| 4151 | ENSG00000123064 | ENSG00000100034 |
| 4152 | ENSG00000089639 | ENSG00000139718 |
| 4153 | ENSG00000116138 | ENSG00000135218 |
| 4154 | ENSG00000140416 | ENSG00000120645 |
| 4155 | ENSG00000100403 | ENSG00000087076 |
| 4156 | ENSG00000105894 | ENSG00000164091 |
| 4157 | ENSG00000124713 | ENSG00000183207 |
| 4158 | ENSG00000188677 | ENSG00000100982 |
| 4159 | ENSG00000187260 | ENSG00000115758 |
| 4160 | ENSG00000125144 | ENSG00000198276 |
| 4161 | ENSG00000105699 | ENSG00000164050 |
| 4162 | ENSG00000170581 | ENSG00000136802 |
| 4163 | ENSG00000135220 | ENSG00000117632 |
| 4164 | ENSG00000111641 | ENSG00000155903 |
| 4165 | ENSG00000215695 | ENSG00000182197 |
| 4166 | ENSG00000174292 | ENSG00000105479 |
| 4167 | ENSG00000068903 | ENSG00000131746 |
| 4168 | ENSG00000138623 | ENSG00000107614 |
| 4169 | ENSG00000183549 | ENSG00000187650 |
| 4170 | ENSG00000116667 | ENSG00000184451 |
| 4171 | ENSG00000109323 | ENSG00000146828 |
| 4172 | ENSG00000162494 | ENSG00000110245 |
| 4173 | ENSG00000197013 | ENSG00000131375 |
| 4174 | ENSG00000149179 | ENSG00000169018 |
| 4175 | ENSG00000137270 | ENSG00000220032 |
| 4176 | ENSG00000182628 | ENSG00000196456 |
| 4177 | ENSG00000170819 | ENSG00000130005 |
| 4178 | ENSG00000115325 | ENSG00000129680 |
| 4179 | ENSG00000165672 | ENSG00000178445 |
| 4180 | ENSG00000206043 | ENSG00000156574 |
| 4181 | ENSG00000161798 | ENSG00000145675 |
| 4182 | ENSG00000164325 | ENSG00000136271 |
| 4183 | ENSG00000168703 | ENSG00000140853 |
| 4184 | ENSG00000234438 | ENSG00000006611 |
| 4185 | ENSG00000171773 | ENSG00000259207 |
| 4186 | ENSG00000013364 | ENSG00000113504 |
| 4187 | ENSG00000187105 | ENSG00000101210 |
| 4188 | ENSG00000168264 | ENSG00000249679 |
| 4189 | ENSG00000185250 | ENSG00000089101 |
| 4190 | ENSG00000182150 | ENSG00000136231 |
| 4191 | ENSG00000182177 | ENSG00000196843 |
| 4192 | ENSG00000102886 | ENSG00000102245 |
| 4193 | ENSG00000183255 | ENSG00000142789 |
| 4194 | ENSG00000163746 | ENSG00000166924 |
| 4195 | ENSG00000143390 | ENSG00000160213 |
| 4196 | ENSG00000182968 | ENSG00000011052 |
| 4197 | ENSG00000104450 | ENSG00000172663 |
| 4198 | ENSG00000248469 | ENSG00000084623 |
| 4199 | ENSG00000129170 | ENSG00000163462 |
| 4200 | ENSG00000164220 | ENSG00000171135 |
| 4201 | ENSG00000010810 | ENSG00000170322 |
| 4202 | ENSG00000042062 | ENSG00000198914 |
| 4203 | ENSG00000124134 | ENSG00000139044 |
| 4204 | ENSG00000186666 | ENSG00000104904 |
| 4205 | ENSG00000163660 | ENSG00000163623 |
| 4206 | ENSG00000125510 | ENSG00000148200 |
| 4207 | ENSG00000110436 | ENSG00000213132 |
| 4208 | ENSG00000109066 | ENSG00000087495 |
| 4209 | ENSG00000255874 | ENSG00000273266 |
| 4210 | ENSG00000269855 | ENSG00000177666 |
| 4211 | ENSG00000183941 | ENSG00000159128 |
| 4212 | ENSG00000267531 | ENSG00000181610 |
| 4213 | ENSG00000100906 | ENSG00000196411 |
| 4214 | ENSG00000165556 | ENSG00000187922 |
| 4215 | ENSG00000267168 | ENSG00000269690 |
| 4216 | ENSG00000141527 | ENSG00000113749 |
| 4217 | ENSG00000162769 | ENSG00000181789 |
| 4218 | ENSG00000168795 | ENSG00000183486 |
| 4219 | ENSG00000141522 | ENSG00000180818 |
| 4220 | ENSG00000136698 | ENSG00000168314 |
| 4221 | ENSG00000160570 | ENSG00000072518 |
| 4222 | ENSG00000205869 | ENSG00000249884 |
| 4223 | ENSG00000124126 | ENSG00000183718 |
| 4224 | ENSG00000156172 | ENSG00000171720 |
| 4225 | ENSG00000212998 | ENSG00000100647 |
| 4226 | ENSG00000162771 | ENSG00000157514 |
| 4227 | ENSG00000140386 | ENSG00000188987 |
| 4228 | ENSG00000111676 | ENSG00000139725 |
| 4229 | ENSG00000042445 | ENSG00000107736 |
| 4230 | ENSG00000135472 | ENSG00000143878 |
| 4231 | ENSG00000126777 | ENSG00000131409 |
| 4232 | ENSG00000155465 | ENSG00000160190 |
| 4233 | ENSG00000188522 | ENSG00000073849 |
| 4234 | ENSG00000134882 | ENSG00000130222 |
| 4235 | ENSG00000152147 | ENSG00000262484 |
| 4236 | ENSG00000108219 | ENSG00000100664 |
| 4237 | ENSG00000173915 | ENSG00000125107 |
| 4238 | ENSG00000164237 | ENSG00000189152 |
| 4239 | ENSG00000213240 | ENSG00000203880 |
| 4240 | ENSG00000171129 | ENSG00000124920 |
| 4241 | ENSG00000141293 | ENSG00000119899 |
| 4242 | ENSG00000109851 | ENSG00000163399 |
| 4243 | ENSG00000204842 | ENSG00000099917 |
| 4244 | ENSG00000102882 | ENSG00000159363 |
| 4245 | ENSG00000197445 | ENSG00000163050 |
| 4246 | ENSG00000104957 | ENSG00000164088 |
| 4247 | ENSG00000163528 | ENSG00000101346 |
| 4248 | ENSG00000259075 | ENSG00000185372 |
| 4249 | ENSG00000143811 | ENSG00000132141 |
| 4250 | ENSG00000178028 | ENSG00000026652 |
| 4251 | ENSG00000138771 | ENSG00000100207 |
| 4252 | ENSG00000205339 | ENSG00000186862 |
| 4253 | ENSG00000135547 | ENSG00000164251 |
| 4254 | ENSG00000004776 | ENSG00000090273 |
| 4255 | ENSG00000127554 | ENSG00000169398 |
| 4256 | ENSG00000163517 | ENSG00000160007 |
| 4257 | ENSG00000178878 | ENSG00000136830 |
| 4258 | ENSG00000135409 | ENSG00000251322 |
| 4259 | ENSG00000198783 | ENSG00000059804 |
| 4260 | ENSG00000126070 | ENSG00000184608 |
| 4261 | ENSG00000123384 | ENSG00000204104 |
| 4262 | ENSG00000244482 | ENSG00000187079 |
| 4263 | ENSG00000173548 | ENSG00000019144 |
| 4264 | ENSG00000141551 | ENSG00000002919 |
| 4265 | ENSG00000269089 | ENSG00000250510 |
| 4266 | ENSG00000188167 | ENSG00000141568 |
| 4267 | ENSG00000100142 | ENSG00000133246 |
| 4268 | ENSG00000126457 | ENSG00000188643 |
| 4269 | ENSG00000138430 | ENSG00000197409 |
| 4270 | ENSG00000084636 | ENSG00000147403 |
| 4271 | ENSG00000163393 | ENSG00000115525 |
| 4272 | ENSG00000255692 | ENSG00000167614 |
| 4273 | ENSG00000143337 | ENSG00000171115 |
| 4274 | ENSG00000089157 | ENSG00000155849 |
| 4275 | ENSG00000268465 | ENSG00000123815 |
| 4276 | ENSG00000107611 | ENSG00000161638 |
| 4277 | ENSG00000269469 | ENSG00000060688 |
| 4278 | ENSG00000177669 | ENSG00000144843 |
| 4279 | ENSG00000127564 | ENSG00000108370 |
| 4280 | ENSG00000171450 | ENSG00000233404 |
| 4281 | ENSG00000184887 | ENSG00000189266 |
| 4282 | ENSG00000140990 | ENSG00000108448 |
| 4283 | ENSG00000148719 | ENSG00000150938 |
| 4284 | ENSG00000172578 | ENSG00000118263 |
| 4285 | ENSG00000186591 | ENSG00000161016 |
| 4286 | ENSG00000117859 | ENSG00000106624 |
| 4287 | ENSG00000204267 | ENSG00000140836 |
| 4288 | ENSG00000149735 | ENSG00000175970 |
| 4289 | ENSG00000173757 | ENSG00000118762 |
| 4290 | ENSG00000181019 | ENSG00000197604 |
| 4291 | ENSG00000132471 | ENSG00000088280 |
| 4292 | ENSG00000213366 | ENSG00000168297 |
| 4293 | ENSG00000008441 | ENSG00000177692 |
| 4294 | ENSG00000119242 | ENSG00000185198 |
| 4295 | ENSG00000117691 | ENSG00000177302 |
| 4296 | ENSG00000113758 | ENSG00000100599 |
| 4297 | ENSG00000006712 | ENSG00000196358 |
| 4298 | ENSG00000177807 | ENSG00000160188 |
| 4299 | ENSG00000129245 | ENSG00000162891 |
| 4300 | ENSG00000101665 | ENSG00000171223 |
| 4301 | ENSG00000182685 | ENSG00000239697 |
| 4302 | ENSG00000122545 | ENSG00000254521 |
| 4303 | ENSG00000143369 | ENSG00000134594 |
| 4304 | ENSG00000183770 | ENSG00000108688 |
| 4305 | ENSG00000179348 | ENSG00000131389 |
| 4306 | ENSG00000149679 | ENSG00000163875 |
| 4307 | ENSG00000006074 | ENSG00000170871 |
| 4308 | ENSG00000140854 | ENSG00000124120 |
| 4309 | ENSG00000109689 | ENSG00000171863 |
| 4310 | ENSG00000125775 | ENSG00000269832 |
| 4311 | ENSG00000168092 | ENSG00000170542 |
| 4312 | ENSG00000104960 | ENSG00000130783 |
| 4313 | ENSG00000163995 | ENSG00000163964 |
| 4314 | ENSG00000128272 | ENSG00000167642 |
| 4315 | ENSG00000136807 | ENSG00000088325 |
| 4316 | ENSG00000142765 | ENSG00000178928 |
| 4317 | ENSG00000184371 | ENSG00000267757 |
| 4318 | ENSG00000184164 | ENSG00000054654 |
| 4319 | ENSG00000105982 | ENSG00000141564 |
| 4320 | ENSG00000063245 | ENSG00000165507 |
| 4321 | ENSG00000235978 | ENSG00000115718 |
| 4322 | ENSG00000259133 | ENSG00000205795 |
| 4323 | ENSG00000267819 | ENSG00000120160 |
| 4324 | ENSG00000134294 | ENSG00000215910 |
| 4325 | ENSG00000134954 | ENSG00000159720 |
| 4326 | ENSG00000255046 | ENSG00000133055 |
| 4327 | ENSG00000131323 | ENSG00000168496 |
| 4328 | ENSG00000168418 | ENSG00000159210 |
| 4329 | ENSG00000122592 | ENSG00000174197 |
| 4330 | ENSG00000173083 | ENSG00000163357 |
| 4331 | ENSG00000141574 | ENSG00000158711 |
| 4332 | ENSG00000142751 | ENSG00000106077 |
| 4333 | ENSG00000143858 | ENSG00000159753 |
| 4334 | ENSG00000057657 | ENSG00000143842 |
| 4335 | ENSG00000064547 | ENSG00000176472 |
| 4336 | ENSG00000148841 | ENSG00000107249 |
| 4337 | ENSG00000125814 | ENSG00000235531 |
| 4338 | ENSG00000163431 | ENSG00000006831 |
| 4339 | ENSG00000070413 | ENSG00000160183 |
| 4340 | ENSG00000120709 | ENSG00000183763 |
| 4341 | ENSG00000184897 | ENSG00000101347 |
| 4342 | ENSG00000225885 | ENSG00000164889 |
| 4343 | ENSG00000134824 | ENSG00000173988 |
| 4344 | ENSG00000076662 | ENSG00000170498 |
| 4345 | ENSG00000166192 | ENSG00000175324 |
| 4346 | ENSG00000166441 | ENSG00000153902 |
| 4347 | ENSG00000113522 | ENSG00000172613 |
| 4348 | ENSG00000153060 | ENSG00000269883 |
| 4349 | ENSG00000140749 | ENSG00000106603 |
| 4350 | ENSG00000124557 | ENSG00000113645 |
| 4351 | ENSG00000102934 | ENSG00000196562 |
| 4352 | ENSG00000198604 | ENSG00000119048 |
| 4353 | ENSG00000198915 | ENSG00000213658 |
| 4354 | ENSG00000162729 | ENSG00000205560 |
| 4355 | ENSG00000250565 | ENSG00000165916 |
| 4356 | ENSG00000110583 | ENSG00000143515 |
| 4357 | ENSG00000055955 | ENSG00000070404 |
| 4358 | ENSG00000176358 | ENSG00000115268 |
| 4359 | ENSG00000101019 | ENSG00000268852 |
| 4360 | ENSG00000157368 | ENSG00000108312 |
| 4361 | ENSG00000105649 | ENSG00000165238 |
| 4362 | ENSG00000064763 | ENSG00000181026 |
| 4363 | ENSG00000138080 | ENSG00000128266 |
| 4364 | ENSG00000196357 | ENSG00000241852 |
| 4365 | ENSG00000086589 | ENSG00000137942 |
| 4366 | ENSG00000106333 | ENSG00000133121 |
| 4367 | ENSG00000063046 | ENSG00000239732 |
| 4368 | ENSG00000026036 | ENSG00000268643 |
| 4369 | ENSG00000176124 | ENSG00000182022 |
| 4370 | ENSG00000143870 | ENSG00000105550 |
| 4371 | ENSG00000129250 | ENSG00000204952 |
| 4372 | ENSG00000174996 | ENSG00000173801 |
| 4373 | ENSG00000149527 | ENSG00000168003 |
| 4374 | ENSG00000256407 | ENSG00000166046 |
| 4375 | ENSG00000160460 | ENSG00000134243 |
| 4376 | ENSG00000213809 | ENSG00000134825 |
| 4377 | ENSG00000198125 | ENSG00000141698 |
| 4378 | ENSG00000170890 | ENSG00000205683 |
| 4379 | ENSG00000171533 | ENSG00000127561 |
| 4380 | ENSG00000166503 | ENSG00000128567 |
| 4381 | ENSG00000110711 | ENSG00000168056 |
| 4382 | ENSG00000177946 | ENSG00000221916 |
| 4383 | ENSG00000143622 | ENSG00000154025 |
| 4384 | ENSG00000167962 | ENSG00000196329 |
| 4385 | ENSG00000118307 | ENSG00000163219 |
| 4386 | ENSG00000101294 | ENSG00000198026 |
| 4387 | ENSG00000079385 | ENSG00000205629 |
| 4388 | ENSG00000153391 | ENSG00000161896 |
| 4389 | ENSG00000127838 | ENSG00000117399 |
| 4390 | ENSG00000184486 | ENSG00000087589 |
| 4391 | ENSG00000198964 | ENSG00000140465 |
| 4392 | ENSG00000159217 | ENSG00000152818 |
| 4393 | ENSG00000216921 | ENSG00000108854 |
| 4394 | ENSG00000173210 | ENSG00000164379 |
| 4395 | ENSG00000154553 | ENSG00000149575 |
| 4396 | ENSG00000005249 | ENSG00000124343 |
| 4397 | ENSG00000214530 | ENSG00000168334 |
| 4398 | ENSG00000101307 | ENSG00000117411 |
| 4399 | ENSG00000068028 | ENSG00000119638 |
| 4400 | ENSG00000116299 | ENSG00000128908 |
| 4401 | ENSG00000179528 | ENSG00000204851 |
| 4402 | ENSG00000229859 | ENSG00000182013 |
| 4403 | ENSG00000116521 | ENSG00000166188 |
| 4404 | ENSG00000177994 | ENSG00000136504 |
| 4405 | ENSG00000205250 | ENSG00000147421 |
| 4406 | ENSG00000160051 | ENSG00000160469 |
| 4407 | ENSG00000171017 | ENSG00000129038 |
| 4408 | ENSG00000160688 | ENSG00000119698 |
| 4409 | ENSG00000110321 | ENSG00000186010 |
| 4410 | ENSG00000204464 | ENSG00000123144 |
| 4411 | ENSG00000186111 | ENSG00000178919 |
| 4412 | ENSG00000268635 | ENSG00000169504 |
| 4413 | ENSG00000213614 | ENSG00000139990 |
| 4414 | ENSG00000189376 | ENSG00000076108 |
| 4415 | ENSG00000112761 | ENSG00000064313 |
| 4416 | ENSG00000164465 | ENSG00000166949 |
| 4417 | ENSG00000140807 | ENSG00000109118 |
| 4418 | ENSG00000076641 | ENSG00000147586 |
| 4419 | ENSG00000144815 | ENSG00000260272 |
| 4420 | ENSG00000135540 | ENSG00000128242 |
| 4421 | ENSG00000236939 | ENSG00000161956 |
| 4422 | ENSG00000162396 | ENSG00000188778 |
| 4423 | ENSG00000165443 | ENSG00000178761 |
| 4424 | ENSG00000187116 | ENSG00000217555 |
| 4425 | ENSG00000118680 | ENSG00000165233 |
| 4426 | ENSG00000024422 | ENSG00000132842 |
| 4427 | ENSG00000154493 | ENSG00000022277 |
| 4428 | ENSG00000168970 | ENSG00000183785 |
| 4429 | ENSG00000114841 | ENSG00000215193 |
| 4430 | ENSG00000128609 | ENSG00000107719 |
| 4431 | ENSG00000129968 | ENSG00000259164 |
| 4432 | ENSG00000211584 | ENSG00000101605 |
| 4433 | ENSG00000006659 | ENSG00000163463 |
| 4434 | ENSG00000124243 | ENSG00000105650 |
| 4435 | ENSG00000006118 | ENSG00000103043 |
| 4436 | ENSG00000134852 | ENSG00000204866 |
| 4437 | ENSG00000122122 | ENSG00000214357 |
| 4438 | ENSG00000205018 | ENSG00000196260 |
| 4439 | ENSG00000183092 | ENSG00000117640 |
| 4440 | ENSG00000184909 | ENSG00000114861 |
| 4441 | ENSG00000273088 | ENSG00000254531 |
| 4442 | ENSG00000137502 | ENSG00000198185 |
| 4443 | ENSG00000163017 | ENSG00000161929 |
| 4444 | ENSG00000268130 | ENSG00000114316 |
| 4445 | ENSG00000168824 | ENSG00000114786 |
| 4446 | ENSG00000104064 | ENSG00000115459 |
| 4447 | ENSG00000102096 | ENSG00000138594 |
| 4448 | ENSG00000137960 | ENSG00000064666 |
| 4449 | ENSG00000169583 | ENSG00000174485 |
| 4450 | ENSG00000152104 | ENSG00000148308 |
| 4451 | ENSG00000150967 | ENSG00000213853 |
| 4452 | ENSG00000113712 | ENSG00000095380 |
| 4453 | ENSG00000176890 | ENSG00000273049 |
| 4454 | ENSG00000005102 | ENSG00000168386 |
| 4455 | ENSG00000170325 | ENSG00000174963 |
| 4456 | ENSG00000180257 | ENSG00000151575 |
| 4457 | ENSG00000090581 | ENSG00000079435 |
| 4458 | ENSG00000115274 | ENSG00000056558 |
| 4459 | ENSG00000118849 | ENSG00000197461 |
| 4460 | ENSG00000197892 | ENSG00000135930 |
| 4461 | ENSG00000162613 | ENSG00000146232 |
| 4462 | ENSG00000160957 | ENSG00000263812 |
| 4463 | ENSG00000183779 | ENSG00000204003 |
| 4464 | ENSG00000139112 | ENSG00000140307 |
| 4465 | ENSG00000159314 | ENSG00000112769 |
| 4466 | ENSG00000166233 | ENSG00000164485 |
| 4467 | ENSG00000162522 | ENSG00000166579 |
| 4468 | ENSG00000124232 | ENSG00000089006 |
| 4469 | ENSG00000183496 | ENSG00000157933 |
| 4470 | ENSG00000125734 | ENSG00000115561 |
| 4471 | ENSG00000145113 | ENSG00000013588 |
| 4472 | ENSG00000196839 | ENSG00000105738 |
| 4473 | ENSG00000140497 | ENSG00000105290 |
| 4474 | ENSG00000177374 | ENSG00000268818 |
| 4475 | ENSG00000196544 | ENSG00000197536 |
| 4476 | ENSG00000117707 | ENSG00000124092 |
| 4477 | ENSG00000269259 | ENSG00000222007 |
| 4478 | ENSG00000167106 | ENSG00000008130 |
| 4479 | ENSG00000117616 | ENSG00000197170 |
| 4480 | ENSG00000166348 | ENSG00000205309 |
| 4481 | ENSG00000087274 | ENSG00000100227 |
| 4482 | ENSG00000136634 | ENSG00000129993 |
| 4483 | ENSG00000088808 | ENSG00000167880 |
| 4484 | ENSG00000164754 | ENSG00000140451 |
| 4485 | ENSG00000237541 | ENSG00000137726 |
| 4486 | ENSG00000167985 | ENSG00000149639 |
| 4487 | ENSG00000128829 | ENSG00000137331 |
| 4488 | ENSG00000117983 | ENSG00000100983 |
| 4489 | ENSG00000148337 | ENSG00000006015 |
| 4490 | ENSG00000197102 | ENSG00000214595 |
| 4491 | ENSG00000185989 | ENSG00000142149 |
| 4492 | ENSG00000151694 | ENSG00000028528 |
| 4493 | ENSG00000268396 | ENSG00000168612 |
| 4494 | ENSG00000034677 | ENSG00000077549 |
| 4495 | ENSG00000177105 | ENSG00000062524 |
| 4496 | ENSG00000126261 | ENSG00000241644 |
| 4497 | ENSG00000116133 | ENSG00000198853 |
| 4498 | ENSG00000166793 | ENSG00000108342 |
| 4499 | ENSG00000120549 | ENSG00000162877 |
| 4500 | ENSG00000150961 | ENSG00000110931 |
| 4501 | ENSG00000172586 | ENSG00000214866 |
| 4502 | ENSG00000234776 | ENSG00000181827 |
| 4503 | ENSG00000068400 | ENSG00000182534 |
| 4504 | ENSG00000103485 | ENSG00000249861 |
| 4505 | ENSG00000203811 | ENSG00000181722 |
| 4506 | ENSG00000177875 | ENSG00000272214 |
| 4507 | ENSG00000166275 | ENSG00000168961 |
| 4508 | ENSG00000144802 | ENSG00000117400 |
| 4509 | ENSG00000137876 | ENSG00000187486 |
| 4510 | ENSG00000184945 | ENSG00000132589 |
| 4511 | ENSG00000138795 | ENSG00000169242 |
| 4512 | ENSG00000242372 | ENSG00000151320 |
| 4513 | ENSG00000183258 | ENSG00000040487 |
| 4514 | ENSG00000121104 | ENSG00000187726 |
| 4515 | ENSG00000125966 | ENSG00000197265 |
| 4516 | ENSG00000153292 | ENSG00000163798 |
| 4517 | ENSG00000168769 | ENSG00000186510 |
| 4518 | ENSG00000149927 | ENSG00000185838 |
| 4519 | ENSG00000178243 | ENSG00000136878 |
| 4520 | ENSG00000108963 | ENSG00000103460 |
| 4521 | ENSG00000150281 | ENSG00000268427 |
| 4522 | ENSG00000160791 | ENSG00000160993 |
| 4523 | ENSG00000176302 | ENSG00000162757 |
| 4524 | ENSG00000167874 | ENSG00000162302 |
| 4525 | ENSG00000167914 | ENSG00000184640 |
| 4526 | ENSG00000160124 | ENSG00000169189 |
| 4527 | ENSG00000123999 | ENSG00000066322 |
| 4528 | ENSG00000187045 | ENSG00000112584 |
| 4529 | ENSG00000003147 | ENSG00000213626 |
| 4530 | ENSG00000107957 | ENSG00000204128 |
| 4531 | ENSG00000176473 | ENSG00000130826 |
| 4532 | ENSG00000137411 | ENSG00000164626 |
| 4533 | ENSG00000137309 | ENSG00000187609 |
| 4534 | ENSG00000159958 | ENSG00000003402 |
| 4535 | ENSG00000176171 | ENSG00000188959 |
| 4536 | ENSG00000162747 | ENSG00000162065 |
| 4537 | ENSG00000162511 | ENSG00000271723 |
| 4538 | ENSG00000118162 | ENSG00000224963 |
| 4539 | ENSG00000268950 | ENSG00000166123 |
| 4540 | ENSG00000168530 | ENSG00000160877 |
| 4541 | ENSG00000104976 | ENSG00000182742 |
| 4542 | ENSG00000204315 | ENSG00000214491 |
| 4543 | ENSG00000106069 | ENSG00000174013 |
| 4544 | ENSG00000157916 | ENSG00000153446 |
| 4545 | ENSG00000268790 | ENSG00000198646 |
| 4546 | ENSG00000111912 | ENSG00000175514 |
| 4547 | ENSG00000184076 | ENSG00000088387 |
| 4548 | ENSG00000117298 | ENSG00000196684 |
| 4549 | ENSG00000073584 | ENSG00000182158 |
| 4550 | ENSG00000188897 | ENSG00000204576 |
| 4551 | ENSG00000152022 | ENSG00000161652 |
| 4552 | ENSG00000137078 | ENSG00000222047 |
| 4553 | ENSG00000180398 | ENSG00000168675 |
| 4554 | ENSG00000104529 | ENSG00000171603 |
| 4555 | ENSG00000153936 | ENSG00000153561 |
| 4556 | ENSG00000169612 | ENSG00000112159 |
| 4557 | ENSG00000180878 | ENSG00000170667 |
| 4558 | ENSG00000071539 | ENSG00000105352 |
| 4559 | ENSG00000176532 | ENSG00000177600 |
| 4560 | ENSG00000163191 | ENSG00000065526 |
| 4561 | ENSG00000101474 | ENSG00000129195 |
| 4562 | ENSG00000186458 | ENSG00000173848 |
| 4563 | ENSG00000205581 | ENSG00000222046 |
| 4564 | ENSG00000172893 | ENSG00000132692 |
| 4565 | ENSG00000095917 | ENSG00000131398 |
| 4566 | ENSG00000140750 | ENSG00000089022 |
| 4567 | ENSG00000228032 | ENSG00000062038 |
| 4568 | ENSG00000219545 | ENSG00000005483 |
| 4569 | ENSG00000100321 | ENSG00000129691 |
| 4570 | ENSG00000125817 | ENSG00000050165 |
| 4571 | ENSG00000070718 | ENSG00000164078 |
| 4572 | ENSG00000118058 | ENSG00000228157 |
| 4573 | ENSG00000164920 | ENSG00000239961 |
| 4574 | ENSG00000272104 | ENSG00000078804 |
| 4575 | ENSG00000073605 | ENSG00000074054 |
| 4576 | ENSG00000112576 | ENSG00000219435 |
| 4577 | ENSG00000112245 | ENSG00000131504 |
| 4578 | ENSG00000125869 |  |
| 4579 | ENSG00000166923 |  |
| 4580 | ENSG00000267937 |  |
| 4581 | ENSG00000139438 |  |
| 4582 | ENSG00000076826 |  |
| 4583 | ENSG00000165626 |  |
| 4584 | ENSG00000268858 |  |
| 4585 | ENSG00000188997 |  |
| 4586 | ENSG00000167195 |  |
| 4587 | ENSG00000197879 |  |
| 4588 | ENSG00000122678 |  |
| 4589 | ENSG00000168505 |  |
| 4590 | ENSG00000160886 |  |
| 4591 | ENSG00000128011 |  |
| 4592 | ENSG00000135469 |  |
| 4593 | ENSG00000109436 |  |
| 4594 | ENSG00000092847 |  |
| 4595 | ENSG00000223601 |  |
| 4596 | ENSG00000159479 |  |
| 4597 | ENSG00000187994 |  |
| 4598 | ENSG00000118407 |  |
| 4599 | ENSG00000092969 |  |
| 4600 | ENSG00000095485 |  |
| 4601 | ENSG00000100353 |  |
| 4602 | ENSG00000264058 |  |
| 4603 | ENSG00000108946 |  |
| 4604 | ENSG00000182613 |  |
| 4605 | ENSG00000100226 |  |
| 4606 | ENSG00000125657 |  |
| 4607 | ENSG00000137259 |  |
| 4608 | ENSG00000061794 |  |
| 4609 | ENSG00000072571 |  |
| 4610 | ENSG00000261594 |  |
| 4611 | ENSG00000126264 |  |
| 4612 | ENSG00000196366 |  |
| 4613 | ENSG00000162888 |  |
| 4614 | ENSG00000188223 |  |
| 4615 | ENSG00000254087 |  |
| 4616 | ENSG00000161905 |  |
| 4617 | ENSG00000104804 |  |
| 4618 | ENSG00000176678 |  |
| 4619 | ENSG00000090339 |  |
| 4620 | ENSG00000162517 |  |
| 4621 | ENSG00000167986 |  |
| 4622 | ENSG00000171747 |  |
| 4623 | ENSG00000008282 |  |
| 4624 | ENSG00000185015 |  |
| 4625 | ENSG00000124831 |  |
| 4626 | ENSG00000089818 |  |
| 4627 | ENSG00000224420 |  |
| 4628 | ENSG00000113368 |  |
| 4629 | ENSG00000273395 |  |
| 4630 | ENSG00000262664 |  |
| 4631 | ENSG00000126461 |  |
| 4632 | ENSG00000206561 |  |
| 4633 | ENSG00000101337 |  |
| 4634 | ENSG00000079257 |  |
| 4635 | ENSG00000171858 |  |
| 4636 | ENSG00000129946 |  |
| 4637 | ENSG00000134802 |  |
| 4638 | ENSG00000142733 |  |
| 4639 | ENSG00000185813 |  |
| 4640 | ENSG00000105329 |  |
| 4641 | ENSG00000130669 |  |
| 4642 | ENSG00000173517 |  |
| 4643 | ENSG00000229314 |  |
| 4644 | ENSG00000176396 |  |
| 4645 | ENSG00000171159 |  |
| 4646 | ENSG00000184900 |  |
| 4647 | ENSG00000178988 |  |
| 4648 | ENSG00000099256 |  |
| 4649 | ENSG00000129673 |  |
| 4650 | ENSG00000185619 |  |
| 4651 | ENSG00000081189 |  |
| 4652 | ENSG00000111707 |  |
| 4653 | ENSG00000131183 |  |
| 4654 | ENSG00000163001 |  |
| 4655 | ENSG00000185551 |  |
| 4656 | ENSG00000103260 |  |
| 4657 | ENSG00000060558 |  |
| 4658 | ENSG00000133884 |  |
| 4659 | ENSG00000107821 |  |
| 4660 | ENSG00000111897 |  |
| 4661 | ENSG00000156273 |  |
| 4662 | ENSG00000197157 |  |
| 4663 | ENSG00000166595 |  |
| 4664 | ENSG00000184209 |  |
| 4665 | ENSG00000151553 |  |
| 4666 | ENSG00000159685 |  |
| 4667 | ENSG00000101040 |  |
| 4668 | ENSG00000167207 |  |
| 4669 | ENSG00000151491 |  |
| 4670 | ENSG00000182512 |  |
| 4671 | ENSG00000188822 |  |
| 4672 | ENSG00000170006 |  |
| 4673 | ENSG00000102032 |  |
| 4674 | ENSG00000102891 |  |
| 4675 | ENSG00000186795 |  |
| 4676 | ENSG00000198518 |  |
| 4677 | ENSG00000130592 |  |
| 4678 | ENSG00000198736 |  |
| 4679 | ENSG00000160472 |  |
| 4680 | ENSG00000122176 |  |
| 4681 | ENSG00000136002 |  |
| 4682 | ENSG00000163145 |  |
| 4683 | ENSG00000126458 |  |
| 4684 | ENSG00000136111 |  |
| 4685 | ENSG00000213551 |  |
| 4686 | ENSG00000104413 |  |
| 4687 | ENSG00000188735 |  |
| 4688 | ENSG00000232859 |  |
| 4689 | ENSG00000189182 |  |
| 4690 | ENSG00000118096 |  |
| 4691 | ENSG00000131669 |  |
| 4692 | ENSG00000053501 |  |
| 4693 | ENSG00000168904 |  |
| 4694 | ENSG00000256950 |  |
| 4695 | ENSG00000134533 |  |
| 4696 | ENSG00000198720 |  |
| 4697 | ENSG00000178573 |  |
| 4698 | ENSG00000174744 |  |
| 4699 | ENSG00000125912 |  |
| 4700 | ENSG00000205155 |  |
| 4701 | ENSG00000160318 |  |
| 4702 | ENSG00000137834 |  |
| 4703 | ENSG00000196187 |  |
| 4704 | ENSG00000196859 |  |
| 4705 | ENSG00000153048 |  |
| 4706 | ENSG00000164929 |  |
| 4707 | ENSG00000165684 |  |
| 4708 | ENSG00000103549 |  |
| 4709 | ENSG00000172361 |  |
| 4710 | ENSG00000185009 |  |
| 4711 | ENSG00000105072 |  |
| 4712 | ENSG00000205269 |  |
| 4713 | ENSG00000062716 |  |
| 4714 | ENSG00000135773 |  |
| 4715 | ENSG00000162576 |  |
| 4716 | ENSG00000197150 |  |
| 4717 | ENSG00000081277 |  |
| 4718 | ENSG00000172345 |  |
| 4719 | ENSG00000159842 |  |
| 4720 | ENSG00000154016 |  |
| 4721 | ENSG00000139946 |  |
| 4722 | ENSG00000111790 |  |
| 4723 | ENSG00000270316 |  |
| 4724 | ENSG00000102935 |  |
| 4725 | ENSG00000135931 |  |
| 4726 | ENSG00000148343 |  |
| 4727 | ENSG00000145506 |  |
| 4728 | ENSG00000180447 |  |
| 4729 | ENSG00000100721 |  |
| 4730 | ENSG00000158882 |  |
| 4731 | ENSG00000197114 |  |
| 4732 | ENSG00000105880 |  |
| 4733 | ENSG00000143774 |  |
| 4734 | ENSG00000177042 |  |
| 4735 | ENSG00000149090 |  |
| 4736 | ENSG00000104783 |  |
| 4737 | ENSG00000115286 |  |
| 4738 | ENSG00000185245 |  |
| 4739 | ENSG00000188786 |  |
| 4740 | ENSG00000117899 |  |
| 4741 | ENSG00000179820 |  |
| 4742 | ENSG00000164318 |  |
| 4743 | ENSG00000163866 |  |
| 4744 | ENSG00000182325 |  |
| 4745 | ENSG00000257365 |  |
| 4746 | ENSG00000255835 |  |
| 4747 | ENSG00000137767 |  |
| 4748 | ENSG00000185105 |  |
| 4749 | ENSG00000144366 |  |
| 4750 | ENSG00000158714 |  |
| 4751 | ENSG00000196946 |  |
| 4752 | ENSG00000136451 |  |
| 4753 | ENSG00000125246 |  |
| 4754 | ENSG00000180389 |  |
| 4755 | ENSG00000186710 |  |
| 4756 | ENSG00000143156 |  |
| 4757 | ENSG00000256713 |  |
| 4758 | ENSG00000196092 |  |
| 4759 | ENSG00000165055 |  |
| 4760 | ENSG00000106348 |  |
| 4761 | ENSG00000138685 |  |
| 4762 | ENSG00000130559 |  |
| 4763 | ENSG00000169972 |  |
| 4764 | ENSG00000049883 |  |
| 4765 | ENSG00000169783 |  |
| 4766 | ENSG00000171320 |  |
| 4767 | ENSG00000161939 |  |
| 4768 | ENSG00000197982 |  |
| 4769 | ENSG00000166477 |  |
| 4770 | ENSG00000075856 |  |
| 4771 | ENSG00000172247 |  |
| 4772 | ENSG00000156097 |  |
| 4773 | ENSG00000267950 |  |
| 4774 | ENSG00000214290 |  |
| 4775 | ENSG00000172071 |  |
| 4776 | ENSG00000181513 |  |
| 4777 | ENSG00000107331 |  |
| 4778 | ENSG00000127585 |  |
| 4779 | ENSG00000067225 |  |
| 4780 | ENSG00000116260 |  |
| 4781 | ENSG00000122861 |  |
| 4782 | ENSG00000103769 |  |
| 4783 | ENSG00000128563 |  |
| 4784 | ENSG00000272325 |  |
| 4785 | ENSG00000101236 |  |
| 4786 | ENSG00000269783 |  |
| 4787 | ENSG00000178623 |  |
| 4788 | ENSG00000108840 |  |
| 4789 | ENSG00000140839 |  |
| 4790 | ENSG00000144857 |  |
| 4791 | ENSG00000165300 |  |
| 4792 | ENSG00000010244 |  |
| 4793 | ENSG00000173598 |  |
| 4794 | ENSG00000103269 |  |
| 4795 | ENSG00000108061 |  |
| 4796 | ENSG00000168928 |  |
| 4797 | ENSG00000269679 |  |
| 4798 | ENSG00000075413 |  |
| 4799 | ENSG00000180053 |  |
| 4800 | ENSG00000166317 |  |
| 4801 | ENSG00000141404 |  |
| 4802 | ENSG00000118260 |  |
| 4803 | ENSG00000176209 |  |
| 4804 | ENSG00000123095 |  |
| 4805 | ENSG00000136367 |  |
| 4806 | ENSG00000171492 |  |
| 4807 | ENSG00000137218 |  |
| 4808 | ENSG00000099622 |  |
| 4809 | ENSG00000154305 |  |
| 4810 | ENSG00000162755 |  |
| 4811 | ENSG00000157637 |  |
| 4812 | ENSG00000116604 |  |
| 4813 | ENSG00000037965 |  |
| 4814 | ENSG00000115137 |  |
| 4815 | ENSG00000258941 |  |
| 4816 | ENSG00000148153 |  |
| 4817 | ENSG00000214854 |  |
| 4818 | ENSG00000131910 |  |
| 4819 | ENSG00000116032 |  |
| 4820 | ENSG00000168135 |  |
| 4821 | ENSG00000110046 |  |
| 4822 | ENSG00000168282 |  |
| 4823 | ENSG00000164967 |  |
| 4824 | ENSG00000170473 |  |
| 4825 | ENSG00000100304 |  |
| 4826 | ENSG00000162869 |  |
| 4827 | ENSG00000114573 |  |
| 4828 | ENSG00000147883 |  |
| 4829 | ENSG00000172380 |  |
| 4830 | ENSG00000143036 |  |
| 4831 | ENSG00000105131 |  |
| 4832 | ENSG00000263588 |  |
| 4833 | ENSG00000125508 |  |
| 4834 | ENSG00000099957 |  |
| 4835 | ENSG00000268179 |  |
| 4836 | ENSG00000165029 |  |
| 4837 | ENSG00000198648 |  |
| 4838 | ENSG00000119772 |  |
| 4839 | ENSG00000171962 |  |
| 4840 | ENSG00000090238 |  |
| 4841 | ENSG00000105717 |  |
| 4842 | ENSG00000078902 |  |
| 4843 | ENSG00000183155 |  |
| 4844 | ENSG00000269175 |  |
| 4845 | ENSG00000130377 |  |
| 4846 | ENSG00000070831 |  |
| 4847 | ENSG00000158552 |  |
| 4848 | ENSG00000198835 |  |
| 4849 | ENSG00000267796 |  |
| 4850 | ENSG00000182446 |  |
| 4851 | ENSG00000180043 |  |
| 4852 | ENSG00000139364 |  |
| 4853 | ENSG00000135480 |  |
| 4854 | ENSG00000084092 |  |
| 4855 | ENSG00000141556 |  |
| 4856 | ENSG00000213923 |  |
| 4857 | ENSG00000135956 |  |
| 4858 | ENSG00000110446 |  |
| 4859 | ENSG00000161847 |  |
| 4860 | ENSG00000108094 |  |
| 4861 | ENSG00000197921 |  |
| 4862 | ENSG00000269205 |  |
| 4863 | ENSG00000169704 |  |
| 4864 | ENSG00000164104 |  |
| 4865 | ENSG00000158545 |  |
| 4866 | ENSG00000103657 |  |
| 4867 | ENSG00000165915 |  |
| 4868 | ENSG00000167613 |  |
| 4869 | ENSG00000111684 |  |
| 4870 | ENSG00000142182 |  |
| 4871 | ENSG00000085117 |  |
| 4872 | ENSG00000100083 |  |
| 4873 | ENSG00000118526 |  |
| 4874 | ENSG00000134780 |  |
| 4875 | ENSG00000108883 |  |
| 4876 | ENSG00000175329 |  |
| 4877 | ENSG00000109511 |  |
| 4878 | ENSG00000162961 |  |
| 4879 | ENSG00000138658 |  |
| 4880 | ENSG00000164406 |  |
| 4881 | ENSG00000140262 |  |
| 4882 | ENSG00000161267 |  |
| 4883 | ENSG00000204599 |  |
| 4884 | ENSG00000182257 |  |
| 4885 | ENSG00000005175 |  |
| 4886 | ENSG00000197487 |  |
| 4887 | ENSG00000106105 |  |
| 4888 | ENSG00000167414 |  |
| 4889 | ENSG00000095585 |  |
| 4890 | ENSG00000165119 |  |
| 4891 | ENSG00000134313 |  |
| 4892 | ENSG00000139193 |  |
| 4893 | ENSG00000064787 |  |
| 4894 | ENSG00000110080 |  |
| 4895 | ENSG00000105963 |  |
| 4896 | ENSG00000123395 |  |
| 4897 | ENSG00000188687 |  |
| 4898 | ENSG00000189114 |  |
| 4899 | ENSG00000137269 |  |
| 4900 | ENSG00000198064 |  |
| 4901 | ENSG00000167792 |  |
| 4902 | ENSG00000111962 |  |
| 4903 | ENSG00000149021 |  |
| 4904 | ENSG00000182372 |  |
| 4905 | ENSG00000064961 |  |
| 4906 | ENSG00000154930 |  |
| 4907 | ENSG00000094755 |  |
| 4908 | ENSG00000269810 |  |
| 4909 | ENSG00000162819 |  |
| 4910 | ENSG00000177885 |  |
| 4911 | ENSG00000162430 |  |
| 4912 | ENSG00000169758 |  |
| 4913 | ENSG00000240230 |  |
| 4914 | ENSG00000113441 |  |
| 4915 | ENSG00000186417 |  |
| 4916 | ENSG00000185453 |  |
| 4917 | ENSG00000172789 |  |
| 4918 | ENSG00000183655 |  |
| 4919 | ENSG00000118298 |  |
| 4920 | ENSG00000129255 |  |
| 4921 | ENSG00000071073 |  |
| 4922 | ENSG00000173080 |  |
| 4923 | ENSG00000166333 |  |
| 4924 | ENSG00000103888 |  |
| 4925 | ENSG00000188779 |  |
| 4926 | ENSG00000268466 |  |
| 4927 | ENSG00000143554 |  |
| 4928 | ENSG00000245848 |  |
| 4929 | ENSG00000177192 |  |
| 4930 | ENSG00000167615 |  |
| 4931 | ENSG00000176444 |  |
| 4932 | ENSG00000174326 |  |
| 4933 | ENSG00000100243 |  |
| 4934 | ENSG00000101204 |  |
| 4935 | ENSG00000185933 |  |
| 4936 | ENSG00000187980 |  |
| 4937 | ENSG00000261221 |  |
| 4938 | ENSG00000060982 |  |
| 4939 | ENSG00000181104 |  |
| 4940 | ENSG00000269094 |  |
| 4941 | ENSG00000233670 |  |
| 4942 | ENSG00000107833 |  |
| 4943 | ENSG00000204256 |  |
| 4944 | ENSG00000206503 |  |
| 4945 | ENSG00000162104 |  |
| 4946 | ENSG00000114742 |  |
| 4947 | ENSG00000105085 |  |
| 4948 | ENSG00000012211 |  |
| 4949 | ENSG00000270136 |  |
| 4950 | ENSG00000198467 |  |
| 4951 | ENSG00000253787 |  |
| 4952 | ENSG00000259009 |  |
| 4953 | ENSG00000167281 |  |
| 4954 | ENSG00000172845 |  |
| 4955 | ENSG00000057593 |  |
| 4956 | ENSG00000120337 |  |
| 4957 | ENSG00000168672 |  |
| 4958 | ENSG00000204536 |  |
| 4959 | ENSG00000180370 |  |
| 4960 | ENSG00000159069 |  |
| 4961 | ENSG00000205143 |  |
| 4962 | ENSG00000188157 |  |
| 4963 | ENSG00000055483 |  |
| 4964 | ENSG00000158748 |  |
| 4965 | ENSG00000052344 |  |
| 4966 | ENSG00000145965 |  |
| 4967 | ENSG00000105705 |  |
| 4968 | ENSG00000167114 |  |
| 4969 | ENSG00000269047 |  |
| 4970 | ENSG00000162144 |  |
| 4971 | ENSG00000184557 |  |
| 4972 | ENSG00000105711 |  |
| 4973 | ENSG00000130182 |  |
| 4974 | ENSG00000105610 |  |
| 4975 | ENSG00000185187 |  |
| 4976 | ENSG00000105370 |  |
| 4977 | ENSG00000160972 |  |
| 4978 | ENSG00000145996 |  |
| 4979 | ENSG00000143486 |  |
| 4980 | ENSG00000100612 |  |
| 4981 | ENSG00000125637 |  |
| 4982 | ENSG00000224051 |  |
| 4983 | ENSG00000219073 |  |
| 4984 | ENSG00000162384 |  |
| 4985 | ENSG00000134160 |  |
| 4986 | ENSG00000090554 |  |
| 4987 | ENSG00000176697 |  |
| 4988 | ENSG00000196224 |  |
| 4989 | ENSG00000180720 |  |
| 4990 | ENSG00000007038 |  |
| 4991 | ENSG00000083290 |  |
| 4992 | ENSG00000144468 |  |
| 4993 | ENSG00000187672 |  |
| 4994 | ENSG00000133805 |  |
| 4995 | ENSG00000172379 |  |
| 4996 | ENSG00000113140 |  |
| 4997 | ENSG00000254470 |  |
| 4998 | ENSG00000136738 |  |
| 4999 | ENSG00000203666 |  |
| 5000 | ENSG00000182199 |  |
| 5001 | ENSG00000164031 |  |
| 5002 | ENSG00000137434 |  |
| 5003 | ENSG00000101198 |  |
| 5004 | ENSG00000171206 |  |
| 5005 | ENSG00000132680 |  |
| 5006 | ENSG00000162910 |  |
| 5007 | ENSG00000168994 |  |
| 5008 | ENSG00000075618 |  |
| 5009 | ENSG00000183760 |  |
| 5010 | ENSG00000139800 |  |
| 5011 | ENSG00000110107 |  |
| 5012 | ENSG00000166833 |  |
| 5013 | ENSG00000148154 |  |
| 5014 | ENSG00000118655 |  |
| 5015 | ENSG00000173762 |  |
| 5016 | ENSG00000136275 |  |
| 5017 | ENSG00000183628 |  |
| 5018 | ENSG00000122557 |  |
| 5019 | ENSG00000177706 |  |
| 5020 | ENSG00000141582 |  |
| 5021 | ENSG00000198931 |  |
| 5022 | ENSG00000174194 |  |
| 5023 | ENSG00000123200 |  |
| 5024 | ENSG00000196118 |  |
| 5025 | ENSG00000171219 |  |
| 5026 | ENSG00000186047 |  |
| 5027 | ENSG00000269831 |  |
| 5028 | ENSG00000242485 |  |
| 5029 | ENSG00000166888 |  |
| 5030 | ENSG00000205922 |  |
| 5031 | ENSG00000132881 |  |
| 5032 | ENSG00000268040 |  |
| 5033 | ENSG00000141699 |  |
| 5034 | ENSG00000146054 |  |
| 5035 | ENSG00000100462 |  |
| 5036 | ENSG00000066735 |  |
| 5037 | ENSG00000269058 |  |
| 5038 | ENSG00000171431 |  |
| 5039 | ENSG00000164344 |  |
| 5040 | ENSG00000154845 |  |
| 5041 | ENSG00000030582 |  |
| 5042 | ENSG00000137843 |  |
| 5043 | ENSG00000104835 |  |
| 5044 | ENSG00000154133 |  |
| 5045 | ENSG00000108590 |  |
| 5046 | ENSG00000121933 |  |
| 5047 | ENSG00000156265 |  |
| 5048 | ENSG00000122970 |  |
| 5049 | ENSG00000152977 |  |
| 5050 | ENSG00000269620 |  |
| 5051 | ENSG00000135828 |  |
| 5052 | ENSG00000241945 |  |
| 5053 | ENSG00000197780 |  |
| 5054 | ENSG00000213892 |  |
| 5055 | ENSG00000197555 |  |
| 5056 | ENSG00000099985 |  |
| 5057 | ENSG00000144152 |  |
| 5058 | ENSG00000145016 |  |
| 5059 | ENSG00000268172 |  |
| 5060 | ENSG00000014164 |  |
| 5061 | ENSG00000102003 |  |
| 5062 | ENSG00000105520 |  |
| 5063 | ENSG00000135299 |  |
| 5064 | ENSG00000205268 |  |
| 5065 | ENSG00000110880 |  |
| 5066 | ENSG00000243444 |  |
| 5067 | ENSG00000164611 |  |
| 5068 | ENSG00000167470 |  |
| 5069 | ENSG00000185905 |  |
| 5070 | ENSG00000182132 |  |
| 5071 | ENSG00000163788 |  |
| 5072 | ENSG00000173404 |  |
| 5073 | ENSG00000164828 |  |
| 5074 | ENSG00000137478 |  |
| 5075 | ENSG00000145220 |  |
| 5076 | ENSG00000124181 |  |
| 5077 | ENSG00000186660 |  |
| 5078 | ENSG00000155096 |  |
| 5079 | ENSG00000164885 |  |
| 5080 | ENSG00000141141 |  |
| 5081 | ENSG00000135473 |  |
| 5082 | ENSG00000141965 |  |
| 5083 | ENSG00000116771 |  |
| 5084 | ENSG00000103313 |  |
| 5085 | ENSG00000100731 |  |
| 5086 | ENSG00000205045 |  |
| 5087 | ENSG00000161281 |  |
| 5088 | ENSG00000184990 |  |
| 5089 | ENSG00000162086 |  |
| 5090 | ENSG00000259495 |  |
| 5091 | ENSG00000185522 |  |
| 5092 | ENSG00000129474 |  |
| 5093 | ENSG00000138166 |  |
| 5094 | ENSG00000111615 |  |
| 5095 | ENSG00000166582 |  |
| 5096 | ENSG00000215902 |  |
| 5097 | ENSG00000148408 |  |
| 5098 | ENSG00000186187 |  |
| 5099 | ENSG00000114019 |  |
| 5100 | ENSG00000229117 |  |
| 5101 | ENSG00000003056 |  |
| 5102 | ENSG00000138867 |  |
| 5103 | ENSG00000163558 |  |
| 5104 | ENSG00000135437 |  |
| 5105 | ENSG00000173327 |  |
| 5106 | ENSG00000150093 |  |
| 5107 | ENSG00000175084 |  |
| 5108 | ENSG00000171060 |  |
| 5109 | ENSG00000111252 |  |
| 5110 | ENSG00000185201 |  |
| 5111 | ENSG00000184270 |  |
| 5112 | ENSG00000114982 |  |
| 5113 | ENSG00000168610 |  |
| 5114 | ENSG00000104953 |  |
| 5115 | ENSG00000069399 |  |
| 5116 | ENSG00000149571 |  |
| 5117 | ENSG00000178971 |  |
| 5118 | ENSG00000139433 |  |
| 5119 | ENSG00000254647 |  |
| 5120 | ENSG00000169490 |  |
| 5121 | ENSG00000139344 |  |
| 5122 | ENSG00000108179 |  |
| 5123 | ENSG00000174306 |  |
| 5124 | ENSG00000168439 |  |
| 5125 | ENSG00000273452 |  |
| 5126 | ENSG00000157106 |  |
| 5127 | ENSG00000116191 |  |
| 5128 | ENSG00000133027 |  |
| 5129 | ENSG00000206203 |  |
| 5130 | ENSG00000268923 |  |
| 5131 | ENSG00000130787 |  |
| 5132 | ENSG00000187961 |  |
| 5133 | ENSG00000133639 |  |
| 5134 | ENSG00000149591 |  |
| 5135 | ENSG00000138303 |  |
| 5136 | ENSG00000150455 |  |
| 5137 | ENSG00000141552 |  |
| 5138 | ENSG00000153786 |  |
| 5139 | ENSG00000217930 |  |
| 5140 | ENSG00000167699 |  |
| 5141 | ENSG00000155130 |  |
| 5142 | ENSG00000111725 |  |
| 5143 | ENSG00000104856 |  |
| 5144 | ENSG00000254656 |  |
| 5145 | ENSG00000149273 |  |
| 5146 | ENSG00000177542 |  |
| 5147 | ENSG00000149743 |  |
| 5148 | ENSG00000268317 |  |
| 5149 | ENSG00000212734 |  |
| 5150 | ENSG00000132535 |  |
| 5151 | ENSG00000110906 |  |
| 5152 | ENSG00000174839 |  |
| 5153 | ENSG00000125611 |  |
| 5154 | ENSG00000135424 |  |
| 5155 | ENSG00000179403 |  |
| 5156 | ENSG00000023191 |  |
| 5157 | ENSG00000008324 |  |
| 5158 | ENSG00000204614 |  |
| 5159 | ENSG00000091428 |  |
| 5160 | ENSG00000111802 |  |
| 5161 | ENSG00000099331 |  |
| 5162 | ENSG00000173338 |  |
| 5163 | ENSG00000175155 |  |
| 5164 | ENSG00000128422 |  |
| 5165 | ENSG00000173638 |  |
| 5166 | ENSG00000268791 |  |
| 5167 | ENSG00000104415 |  |
| 5168 | ENSG00000255062 |  |
| 5169 | ENSG00000077514 |  |
| 5170 | ENSG00000104903 |  |
| 5171 | ENSG00000160293 |  |
| 5172 | ENSG00000158941 |  |
| 5173 | ENSG00000160932 |  |
| 5174 | ENSG00000071246 |  |
| 5175 | ENSG00000004534 |  |
| 5176 | ENSG00000133110 |  |
| 5177 | ENSG00000169762 |  |
| 5178 | ENSG00000057608 |  |
| 5179 | ENSG00000267314 |  |
| 5180 | ENSG00000133019 |  |
| 5181 | ENSG00000100697 |  |
| 5182 | ENSG00000163520 |  |
| 5183 | ENSG00000158869 |  |
| 5184 | ENSG00000105202 |  |
| 5185 | ENSG00000158636 |  |
| 5186 | ENSG00000169418 |  |
| 5187 | ENSG00000112320 |  |
| 5188 | ENSG00000143153 |  |
| 5189 | ENSG00000163286 |  |
| 5190 | ENSG00000071553 |  |
| 5191 | ENSG00000143499 |  |
| 5192 | ENSG00000078487 |  |
| 5193 | ENSG00000198730 |  |
| 5194 | ENSG00000118007 |  |
| 5195 | ENSG00000138459 |  |
| 5196 | ENSG00000213995 |  |
| 5197 | ENSG00000117122 |  |
| 5198 | ENSG00000106803 |  |
| 5199 | ENSG00000177595 |  |
| 5200 | ENSG00000149150 |  |
| 5201 | ENSG00000158023 |  |
| 5202 | ENSG00000149573 |  |
| 5203 | ENSG00000157653 |  |
| 5204 | ENSG00000156171 |  |
| 5205 | ENSG00000174358 |  |
| 5206 | ENSG00000184508 |  |
| 5207 | ENSG00000056972 |  |
| 5208 | ENSG00000255284 |  |
| 5209 | ENSG00000088298 |  |
| 5210 | ENSG00000204991 |  |
| 5211 | ENSG00000115677 |  |
| 5212 | ENSG00000153904 |  |
| 5213 | ENSG00000145214 |  |
| 5214 | ENSG00000102243 |  |
| 5215 | ENSG00000143643 |  |
| 5216 | ENSG00000203711 |  |
| 5217 | ENSG00000140506 |  |
| 5218 | ENSG00000198826 |  |
| 5219 | ENSG00000054356 |  |
| 5220 | ENSG00000211460 |  |
| 5221 | ENSG00000160870 |  |
| 5222 | ENSG00000186088 |  |
| 5223 | ENSG00000272195 |  |
| 5224 | ENSG00000125877 |  |
| 5225 | ENSG00000162642 |  |
| 5226 | ENSG00000105879 |  |
| 5227 | ENSG00000104870 |  |
| 5228 | ENSG00000107738 |  |
| 5229 | ENSG00000148671 |  |
| 5230 | ENSG00000171853 |  |
| 5231 | ENSG00000073734 |  |
| 5232 | ENSG00000258539 |  |
| 5233 | ENSG00000169903 |  |
| 5234 | ENSG00000196547 |  |
| 5235 | ENSG00000147454 |  |
| 5236 | ENSG00000205726 |  |
| 5237 | ENSG00000105518 |  |
| 5238 | ENSG00000132205 |  |
| 5239 | ENSG00000179761 |  |
| 5240 | ENSG00000198898 |  |
| 5241 | ENSG00000132824 |  |
| 5242 | ENSG00000134138 |  |
| 5243 | ENSG00000197301 |  |
| 5244 | ENSG00000082397 |  |
| 5245 | ENSG00000079616 |  |
| 5246 | ENSG00000160161 |  |
| 5247 | ENSG00000151474 |  |
| 5248 | ENSG00000161533 |  |
| 5249 | ENSG00000186174 |  |
| 5250 | ENSG00000196872 |  |
| 5251 | ENSG00000100422 |  |
| 5252 | ENSG00000070731 |  |
| 5253 | ENSG00000268500 |  |
| 5254 | ENSG00000188242 |  |
| 5255 | ENSG00000204822 |  |
| 5256 | ENSG00000127334 |  |
| 5257 | ENSG00000268674 |  |
| 5258 | ENSG00000137868 |  |
| 5259 | ENSG00000128641 |  |
| 5260 | ENSG00000053254 |  |
| 5261 | ENSG00000204616 |  |
| 5262 | ENSG00000072958 |  |
| 5263 | ENSG00000062598 |  |
| 5264 | ENSG00000214413 |  |
| 5265 | ENSG00000146670 |  |
| 5266 | ENSG00000007520 |  |
| 5267 | ENSG00000184682 |  |
| 5268 | ENSG00000104859 |  |
| 5269 | ENSG00000166439 |  |
| 5270 | ENSG00000167595 |  |
| 5271 | ENSG00000125409 |  |
| 5272 | ENSG00000166900 |  |
| 5273 | ENSG00000068831 |  |
| 5274 | ENSG00000256797 |  |
| 5275 | ENSG00000104827 |  |
| 5276 | ENSG00000179241 |  |
| 5277 | ENSG00000105968 |  |
| 5278 | ENSG00000148834 |  |
| 5279 | ENSG00000105771 |  |
| 5280 | ENSG00000172818 |  |
| 5281 | ENSG00000183476 |  |
| 5282 | ENSG00000118922 |  |
| 5283 | ENSG00000198821 |  |
| 5284 | ENSG00000249222 |  |
| 5285 | ENSG00000148943 |  |
| 5286 | ENSG00000104812 |  |
| 5287 | ENSG00000088832 |  |
| 5288 | ENSG00000170917 |  |
| 5289 | ENSG00000148814 |  |
| 5290 | ENSG00000064205 |  |
| 5291 | ENSG00000144504 |  |
| 5292 | ENSG00000187051 |  |
| 5293 | ENSG00000143318 |  |
| 5294 | ENSG00000112977 |  |
| 5295 | ENSG00000124422 |  |
| 5296 | ENSG00000196570 |  |
| 5297 | ENSG00000156256 |  |
| 5298 | ENSG00000064115 |  |
| 5299 | ENSG00000205352 |  |
| 5300 | ENSG00000184544 |  |
| 5301 | ENSG00000124782 |  |
| 5302 | ENSG00000196663 |  |
| 5303 | ENSG00000229974 |  |
| 5304 | ENSG00000256222 |  |
| 5305 | ENSG00000166166 |  |
| 5306 | ENSG00000137709 |  |
| 5307 | ENSG00000119392 |  |
| 5308 | ENSG00000239665 |  |
| 5309 | ENSG00000185386 |  |
| 5310 | ENSG00000179583 |  |
| 5311 | ENSG00000139645 |  |
| 5312 | ENSG00000172175 |  |
| 5313 | ENSG00000102781 |  |
| 5314 | ENSG00000267281 |  |
| 5315 | ENSG00000140848 |  |
| 5316 | ENSG00000244187 |  |
| 5317 | ENSG00000134330 |  |
| 5318 | ENSG00000144285 |  |
| 5319 | ENSG00000145975 |  |
| 5320 | ENSG00000142233 |  |
| 5321 | ENSG00000143847 |  |
| 5322 | ENSG00000000005 |  |
| 5323 | ENSG00000138134 |  |
| 5324 | ENSG00000166173 |  |
| 5325 | ENSG00000111780 |  |
| 5326 | ENSG00000112667 |  |
| 5327 | ENSG00000171132 |  |
| 5328 | ENSG00000130511 |  |
| 5329 | ENSG00000204574 |  |
| 5330 | ENSG00000253293 |  |
| 5331 | ENSG00000184916 |  |
| 5332 | ENSG00000076984 |  |
| 5333 | ENSG00000197891 |  |
| 5334 | ENSG00000133315 |  |
| 5335 | ENSG00000167107 |  |
| 5336 | ENSG00000214732 |  |
| 5337 | ENSG00000015532 |  |
| 5338 | ENSG00000120733 |  |
| 5339 | ENSG00000136478 |  |
| 5340 | ENSG00000116132 |  |
| 5341 | ENSG00000163156 |  |
| 5342 | ENSG00000105227 |  |
| 5343 | ENSG00000189120 |  |
| 5344 | ENSG00000109458 |  |
| 5345 | ENSG00000112699 |  |
| 5346 | ENSG00000108465 |  |
| 5347 | ENSG00000135525 |  |
| 5348 | ENSG00000054967 |  |
| 5349 | ENSG00000255093 |  |
| 5350 | ENSG00000140598 |  |
| 5351 | ENSG00000169375 |  |
| 5352 | ENSG00000260861 |  |
| 5353 | ENSG00000112640 |  |
| 5354 | ENSG00000168350 |  |
| 5355 | ENSG00000262633 |  |
| 5356 | ENSG00000165637 |  |
| 5357 | ENSG00000087269 |  |
| 5358 | ENSG00000125733 |  |
| 5359 | ENSG00000139437 |  |
| 5360 | ENSG00000243135 |  |
| 5361 | ENSG00000183605 |  |
| 5362 | ENSG00000141349 |  |
| 5363 | ENSG00000255181 |  |
| 5364 | ENSG00000111642 |  |
| 5365 | ENSG00000154096 |  |
| 5366 | ENSG00000197948 |  |
| 5367 | ENSG00000102024 |  |
| 5368 | ENSG00000188032 |  |
| 5369 | ENSG00000131242 |  |
| 5370 | ENSG00000263053 |  |
| 5371 | ENSG00000164300 |  |
| 5372 | ENSG00000203812 |  |
| 5373 | ENSG00000152518 |  |
| 5374 | ENSG00000153406 |  |
| 5375 | ENSG00000136485 |  |
| 5376 | ENSG00000143416 |  |
| 5377 | ENSG00000177106 |  |
| 5378 | ENSG00000268154 |  |
| 5379 | ENSG00000170727 |  |
| 5380 | ENSG00000117984 |  |
| 5381 | ENSG00000179532 |  |
| 5382 | ENSG00000179833 |  |
| 5383 | ENSG00000181649 |  |
| 5384 | ENSG00000099385 |  |
| 5385 | ENSG00000197183 |  |
| 5386 | ENSG00000108296 |  |
| 5387 | ENSG00000104375 |  |
| 5388 | ENSG00000000938 |  |
| 5389 | ENSG00000167772 |  |
| 5390 | ENSG00000159063 |  |
| 5391 | ENSG00000130779 |  |
| 5392 | ENSG00000196998 |  |
| 5393 | ENSG00000166913 |  |
| 5394 | ENSG00000128335 |  |
| 5395 | ENSG00000173894 |  |
| 5396 | ENSG00000178033 |  |
| 5397 | ENSG00000167658 |  |
| 5398 | ENSG00000073169 |  |
| 5399 | ENSG00000174915 |  |
| 5400 | ENSG00000269165 |  |
| 5401 | ENSG00000086159 |  |
| 5402 | ENSG00000122367 |  |
| 5403 | ENSG00000147955 |  |
| 5404 | ENSG00000173124 |  |
| 5405 | ENSG00000164299 |  |
| 5406 | ENSG00000152969 |  |
| 5407 | ENSG00000088538 |  |
| 5408 | ENSG00000160602 |  |
| 5409 | ENSG00000100241 |  |
| 5410 | ENSG00000006377 |  |
| 5411 | ENSG00000128710 |  |
| 5412 | ENSG00000261794 |  |
| 5413 | ENSG00000136997 |  |
| 5414 | ENSG00000151006 |  |
| 5415 | ENSG00000088053 |  |
| 5416 | ENSG00000032219 |  |
| 5417 | ENSG00000198598 |  |
| 5418 | ENSG00000215021 |  |
| 5419 | ENSG00000062822 |  |
| 5420 | ENSG00000117308 |  |
| 5421 | ENSG00000111291 |  |
| 5422 | ENSG00000148429 |  |
| 5423 | ENSG00000100100 |  |
| 5424 | ENSG00000250709 |  |
| 5425 | ENSG00000170577 |  |
| 5426 | ENSG00000103266 |  |
| 5427 | ENSG00000249471 |  |
| 5428 | ENSG00000182831 |  |
| 5429 | ENSG00000167904 |  |
| 5430 | ENSG00000196748 |  |
| 5431 | ENSG00000204351 |  |
| 5432 | ENSG00000115590 |  |
| 5433 | ENSG00000198752 |  |
| 5434 | ENSG00000148660 |  |
| 5435 | ENSG00000137077 |  |
| 5436 | ENSG00000136819 |  |
| 5437 | ENSG00000136250 |  |
| 5438 | ENSG00000258875 |  |
| 5439 | ENSG00000128626 |  |
| 5440 | ENSG00000133872 |  |
| 5441 | ENSG00000066117 |  |
| 5442 | ENSG00000156234 |  |
| 5443 | ENSG00000105509 |  |
| 5444 | ENSG00000105355 |  |
| 5445 | ENSG00000140931 |  |
| 5446 | ENSG00000164040 |  |
| 5447 | ENSG00000115884 |  |
| 5448 | ENSG00000178217 |  |
| 5449 | ENSG00000118160 |  |
| 5450 | ENSG00000112851 |  |
| 5451 | ENSG00000170584 |  |
| 5452 | ENSG00000187535 |  |
| 5453 | ENSG00000105220 |  |
| 5454 | ENSG00000183260 |  |
| 5455 | ENSG00000117643 |  |
| 5456 | ENSG00000099783 |  |
| 5457 | ENSG00000007968 |  |
| 5458 | ENSG00000164506 |  |
| 5459 | ENSG00000102606 |  |
| 5460 | ENSG00000100320 |  |
| 5461 | ENSG00000112137 |  |
| 5462 | ENSG00000157551 |  |
| 5463 | ENSG00000148400 |  |
| 5464 | ENSG00000104731 |  |
| 5465 | ENSG00000168477 |  |
| 5466 | ENSG00000186487 |  |
| 5467 | ENSG00000088899 |  |
| 5468 | ENSG00000184163 |  |
| 5469 | ENSG00000198832 |  |
| 5470 | ENSG00000135637 |  |
| 5471 | ENSG00000156853 |  |
| 5472 | ENSG00000124614 |  |
| 5473 | ENSG00000171471 |  |
| 5474 | ENSG00000268865 |  |
| 5475 | ENSG00000105443 |  |
| 5476 | ENSG00000147799 |  |
| 5477 | ENSG00000173456 |  |
| 5478 | ENSG00000203876 |  |
| 5479 | ENSG00000257242 |  |
| 5480 | ENSG00000112531 |  |
| 5481 | ENSG00000172780 |  |
| 5482 | ENSG00000163344 |  |
| 5483 | ENSG00000103335 |  |
| 5484 | ENSG00000028277 |  |
| 5485 | ENSG00000184925 |  |
| 5486 | ENSG00000149782 |  |
| 5487 | ENSG00000111481 |  |
| 5488 | ENSG00000163634 |  |
| 5489 | ENSG00000139289 |  |
| 5490 | ENSG00000165495 |  |
| 5491 | ENSG00000269676 |  |
| 5492 | ENSG00000116254 |  |
| 5493 | ENSG00000229809 |  |
| 5494 | ENSG00000154027 |  |
| 5495 | ENSG00000141644 |  |
| 5496 | ENSG00000186340 |  |
| 5497 | ENSG00000162728 |  |
| 5498 | ENSG00000115756 |  |
| 5499 | ENSG00000109736 |  |
| 5500 | ENSG00000106031 |  |
| 5501 | ENSG00000170956 |  |
| 5502 | ENSG00000185298 |  |
| 5503 | ENSG00000107882 |  |
| 5504 | ENSG00000204709 |  |
| 5505 | ENSG00000259431 |  |
| 5506 | ENSG00000068308 |  |
| 5507 | ENSG00000135631 |  |
| 5508 | ENSG00000084463 |  |
| 5509 | ENSG00000183087 |  |
| 5510 | ENSG00000184988 |  |
| 5511 | ENSG00000111325 |  |
| 5512 | ENSG00000205832 |  |
| 5513 | ENSG00000198408 |  |
| 5514 | ENSG00000099326 |  |
| 5515 | ENSG00000166391 |  |
| 5516 | ENSG00000241186 |  |
| 5517 | ENSG00000089558 |  |
| 5518 | ENSG00000166340 |  |
| 5519 | ENSG00000166676 |  |
| 5520 | ENSG00000108443 |  |
| 5521 | ENSG00000213029 |  |
| 5522 | ENSG00000181873 |  |
| 5523 | ENSG00000180884 |  |
| 5524 | ENSG00000134539 |  |
| 5525 | ENSG00000080845 |  |
| 5526 | ENSG00000105373 |  |
| 5527 | ENSG00000106004 |  |
| 5528 | ENSG00000162341 |  |
| 5529 | ENSG00000136929 |  |
| 5530 | ENSG00000084207 |  |
| 5531 | ENSG00000173511 |  |
| 5532 | ENSG00000100234 |  |
| 5533 | ENSG00000083312 |  |
| 5534 | ENSG00000115361 |  |
| 5535 | ENSG00000166446 |  |
| 5536 | ENSG00000197361 |  |
| 5537 | ENSG00000162650 |  |
| 5538 | ENSG00000139187 |  |
| 5539 | ENSG00000179148 |  |
| 5540 | ENSG00000182472 |  |
| 5541 | ENSG00000250091 |  |
| 5542 | ENSG00000162777 |  |
| 5543 | ENSG00000137161 |  |
| 5544 | ENSG00000161036 |  |
| 5545 | ENSG00000168701 |  |
| 5546 | ENSG00000224870 |  |
| 5547 | ENSG00000143434 |  |
| 5548 | ENSG00000128645 |  |
| 5549 | ENSG00000173011 |  |
| 5550 | ENSG00000002834 |  |
| 5551 | ENSG00000106683 |  |
| 5552 | ENSG00000106336 |  |
| 5553 | ENSG00000187239 |  |
| 5554 | ENSG00000005187 |  |
| 5555 | ENSG00000128918 |  |
| 5556 | ENSG00000118894 |  |
| 5557 | ENSG00000130731 |  |
| 5558 | ENSG00000158796 |  |
| 5559 | ENSG00000129422 |  |
| 5560 | ENSG00000260286 |  |
| 5561 | ENSG00000132510 |  |
| 5562 | ENSG00000133030 |  |
| 5563 | ENSG00000111261 |  |
| 5564 | ENSG00000123106 |  |
| 5565 | ENSG00000147576 |  |
| 5566 | ENSG00000183914 |  |
| 5567 | ENSG00000146842 |  |
| 5568 | ENSG00000198053 |  |
| 5569 | ENSG00000204713 |  |
| 5570 | ENSG00000049449 |  |
| 5571 | ENSG00000170484 |  |
| 5572 | ENSG00000136573 |  |
| 5573 | ENSG00000172005 |  |
| 5574 | ENSG00000143850 |  |
| 5575 | ENSG00000170684 |  |
| 5576 | ENSG00000173838 |  |
| 5577 | ENSG00000185504 |  |
| 5578 | ENSG00000100362 |  |
| 5579 | ENSG00000140948 |  |
| 5580 | ENSG00000178999 |  |
| 5581 | ENSG00000213339 |  |
| 5582 | ENSG00000139746 |  |
| 5583 | ENSG00000122121 |  |
| 5584 | ENSG00000165511 |  |
| 5585 | ENSG00000137070 |  |
| 5586 | ENSG00000170166 |  |
| 5587 | ENSG00000033327 |  |
| 5588 | ENSG00000100429 |  |
| 5589 | ENSG00000116786 |  |
| 5590 | ENSG00000089597 |  |
| 5591 | ENSG00000178764 |  |
| 5592 | ENSG00000170627 |  |
| 5593 | ENSG00000162614 |  |
| 5594 | ENSG00000152894 |  |
| 5595 | ENSG00000130313 |  |
| 5596 | ENSG00000182580 |  |
| 5597 | ENSG00000181135 |  |
| 5598 | ENSG00000175463 |  |
| 5599 | ENSG00000167765 |  |
| 5600 | ENSG00000169499 |  |
| 5601 | ENSG00000149499 |  |
| 5602 | ENSG00000123415 |  |
| 5603 | ENSG00000023041 |  |
| 5604 | ENSG00000103152 |  |
| 5605 | ENSG00000104365 |  |
| 5606 | ENSG00000148344 |  |
| 5607 | ENSG00000176912 |  |
| 5608 | ENSG00000159873 |  |
| 5609 | ENSG00000141744 |  |
| 5610 | ENSG00000168925 |  |
| 5611 | ENSG00000116983 |  |
| 5612 | ENSG00000093183 |  |
| 5613 | ENSG00000178297 |  |
| 5614 | ENSG00000187954 |  |
| 5615 | ENSG00000188771 |  |
| 5616 | ENSG00000189362 |  |
| 5617 | ENSG00000157680 |  |
| 5618 | ENSG00000173457 |  |
| 5619 | ENSG00000268809 |  |
| 5620 | ENSG00000197774 |  |
| 5621 | ENSG00000147896 |  |
| 5622 | ENSG00000005379 |  |
| 5623 | ENSG00000085276 |  |
| 5624 | ENSG00000109586 |  |
| 5625 | ENSG00000205189 |  |
| 5626 | ENSG00000087157 |  |
| 5627 | ENSG00000118960 |  |
| 5628 | ENSG00000136449 |  |
| 5629 | ENSG00000124155 |  |
| 5630 | ENSG00000164500 |  |
| 5631 | ENSG00000125945 |  |
| 5632 | ENSG00000069020 |  |
| 5633 | ENSG00000196535 |  |
| 5634 | ENSG00000176973 |  |
| 5635 | ENSG00000172232 |  |
| 5636 | ENSG00000134013 |  |
| 5637 | ENSG00000196091 |  |
| 5638 | ENSG00000137486 |  |
| 5639 | ENSG00000248751 |  |
| 5640 | ENSG00000150712 |  |
| 5641 | ENSG00000143061 |  |
| 5642 | ENSG00000244752 |  |
| 5643 | ENSG00000270466 |  |
| 5644 | ENSG00000147689 |  |
| 5645 | ENSG00000115464 |  |
| 5646 | ENSG00000188994 |  |
| 5647 | ENSG00000268503 |  |
| 5648 | ENSG00000162734 |  |
| 5649 | ENSG00000101004 |  |
| 5650 | ENSG00000111145 |  |
| 5651 | ENSG00000120896 |  |
| 5652 | ENSG00000118503 |  |
| 5653 | ENSG00000158169 |  |
| 5654 | ENSG00000196735 |  |
| 5655 | ENSG00000205307 |  |
| 5656 | ENSG00000272573 |  |
| 5657 | ENSG00000158270 |  |
| 5658 | ENSG00000100410 |  |
| 5659 | ENSG00000100726 |  |
| 5660 | ENSG00000108349 |  |
| 5661 | ENSG00000057294 |  |
| 5662 | ENSG00000011028 |  |
| 5663 | ENSG00000077235 |  |
| 5664 | ENSG00000197971 |  |
| 5665 | ENSG00000146197 |  |
| 5666 | ENSG00000182541 |  |
| 5667 | ENSG00000105058 |  |
| 5668 | ENSG00000110888 |  |
| 5669 | ENSG00000243335 |  |
| 5670 | ENSG00000112787 |  |
| 5671 | ENSG00000171161 |  |
| 5672 | ENSG00000137198 |  |
| 5673 | ENSG00000222009 |  |
| 5674 | ENSG00000099995 |  |
| 5675 | ENSG00000161671 |  |
| 5676 | ENSG00000158786 |  |
| 5677 | ENSG00000151062 |  |
| 5678 | ENSG00000152495 |  |
| 5679 | ENSG00000134884 |  |
| 5680 | ENSG00000132950 |  |
| 5681 | ENSG00000170291 |  |
| 5682 | ENSG00000182324 |  |
| 5683 | ENSG00000135324 |  |
| 5684 | ENSG00000157064 |  |
| 5685 | ENSG00000129562 |  |
| 5686 | ENSG00000159784 |  |
| 5687 | ENSG00000110243 |  |
| 5688 | ENSG00000119650 |  |
| 5689 | ENSG00000005022 |  |
| 5690 | ENSG00000186838 |  |
| 5691 | ENSG00000180233 |  |
| 5692 | ENSG00000128917 |  |
| 5693 | ENSG00000259159 |  |
| 5694 | ENSG00000224383 |  |
| 5695 | ENSG00000267157 |  |
| 5696 | ENSG00000181222 |  |
| 5697 | ENSG00000142156 |  |
| 5698 | ENSG00000108344 |  |
| 5699 | ENSG00000271949 |  |
| 5700 | ENSG00000090686 |  |
| 5701 | ENSG00000100350 |  |
| 5702 | ENSG00000261717 |  |
| 5703 | ENSG00000164308 |  |
| 5704 | ENSG00000065675 |  |
| 5705 | ENSG00000231852 |  |
| 5706 | ENSG00000074855 |  |
| 5707 | ENSG00000080546 |  |
| 5708 | ENSG00000122692 |  |
| 5709 | ENSG00000168310 |  |
| 5710 | ENSG00000166750 |  |
| 5711 | ENSG00000064195 |  |
| 5712 | ENSG00000133226 |  |
| 5713 | ENSG00000118600 |  |
| 5714 | ENSG00000113575 |  |
| 5715 | ENSG00000048342 |  |
| 5716 | ENSG00000153707 |  |
| 5717 | ENSG00000145391 |  |
| 5718 | ENSG00000075884 |  |
| 5719 | ENSG00000140988 |  |
| 5720 | ENSG00000189129 |  |
| 5721 | ENSG00000182557 |  |
| 5722 | ENSG00000183665 |  |
| 5723 | ENSG00000162365 |  |
| 5724 | ENSG00000268028 |  |
| 5725 | ENSG00000160221 |  |
| 5726 | ENSG00000188293 |  |
| 5727 | ENSG00000019169 |  |
| 5728 | ENSG00000134575 |  |
| 5729 | ENSG00000133256 |  |
| 5730 | ENSG00000166394 |  |
| 5731 | ENSG00000214050 |  |
| 5732 | ENSG00000138101 |  |
| 5733 | ENSG00000101190 |  |
| 5734 | ENSG00000198788 |  |
| 5735 | ENSG00000187990 |  |
| 5736 | ENSG00000166902 |  |
| 5737 | ENSG00000183154 |  |
| 5738 | ENSG00000204889 |  |
| 5739 | ENSG00000130513 |  |
| 5740 | ENSG00000268975 |  |
| 5741 | ENSG00000228835 |  |
| 5742 | ENSG00000101751 |  |
| 5743 | ENSG00000071859 |  |
| 5744 | ENSG00000025796 |  |
| 5745 | ENSG00000116774 |  |
| 5746 | ENSG00000203867 |  |
| 5747 | ENSG00000143740 |  |
| 5748 | ENSG00000164451 |  |
| 5749 | ENSG00000057757 |  |
| 5750 | ENSG00000250644 |  |
| 5751 | ENSG00000267958 |  |
| 5752 | ENSG00000143537 |  |
| 5753 | ENSG00000211445 |  |
| 5754 | ENSG00000206177 |  |
| 5755 | ENSG00000164363 |  |
| 5756 | ENSG00000101082 |  |
| 5757 | ENSG00000131759 |  |
| 5758 | ENSG00000205821 |  |
| 5759 | ENSG00000177731 |  |
| 5760 | ENSG00000162694 |  |
| 5761 | ENSG00000115468 |  |
| 5762 | ENSG00000140950 |  |
| 5763 | ENSG00000127419 |  |
| 5764 | ENSG00000125826 |  |
| 5765 | ENSG00000203685 |  |
| 5766 | ENSG00000105011 |  |
| 5767 | ENSG00000268556 |  |
| 5768 | ENSG00000176809 |  |
| 5769 | ENSG00000116584 |  |
| 5770 | ENSG00000056661 |  |
| 5771 | ENSG00000162851 |  |
| 5772 | ENSG00000162723 |  |
| 5773 | ENSG00000074181 |  |
| 5774 | ENSG00000143140 |  |
| 5775 | ENSG00000173349 |  |
| 5776 | ENSG00000159388 |  |
| 5777 | ENSG00000108774 |  |
| 5778 | ENSG00000140406 |  |
| 5779 | ENSG00000126351 |  |
| 5780 | ENSG00000100324 |  |
| 5781 | ENSG00000164638 |  |
| 5782 | ENSG00000065029 |  |
| 5783 | ENSG00000179115 |  |
| 5784 | ENSG00000167711 |  |
| 5785 | ENSG00000131771 |  |
| 5786 | ENSG00000118156 |  |
| 5787 | ENSG00000197935 |  |
| 5788 | ENSG00000151692 |  |
| 5789 | ENSG00000229086 |  |
| 5790 | ENSG00000176619 |  |
| 5791 | ENSG00000140464 |  |
| 5792 | ENSG00000258674 |  |
| 5793 | ENSG00000117148 |  |
| 5794 | ENSG00000169964 |  |
| 5795 | ENSG00000105497 |  |
| 5796 | ENSG00000167987 |  |
| 5797 | ENSG00000115816 |  |
| 5798 | ENSG00000140254 |  |
| 5799 | ENSG00000120664 |  |
| 5800 | ENSG00000170365 |  |
| 5801 | ENSG00000178257 |  |
| 5802 | ENSG00000142173 |  |
| 5803 | ENSG00000248487 |  |
| 5804 | ENSG00000084444 |  |
| 5805 | ENSG00000181085 |  |
| 5806 | ENSG00000099910 |  |
| 5807 | ENSG00000142959 |  |
| 5808 | ENSG00000174840 |  |
| 5809 | ENSG00000141664 |  |
| 5810 | ENSG00000102890 |  |
| 5811 | ENSG00000134030 |  |
| 5812 | ENSG00000134900 |  |
| 5813 | ENSG00000197375 |  |
| 5814 | ENSG00000104112 |  |
| 5815 | ENSG00000104805 |  |
| 5816 | ENSG00000259571 |  |
| 5817 | ENSG00000026508 |  |
| 5818 | ENSG00000163877 |  |
| 5819 | ENSG00000261567 |  |
| 5820 | ENSG00000163453 |  |
| 5821 | ENSG00000163825 |  |
| 5822 | ENSG00000182459 |  |
| 5823 | ENSG00000181778 |  |
| 5824 | ENSG00000204580 |  |
| 5825 | ENSG00000101222 |  |
| 5826 | ENSG00000255529 |  |
| 5827 | ENSG00000187242 |  |
| 5828 | ENSG00000178685 |  |
| 5829 | ENSG00000112365 |  |
| 5830 | ENSG00000178075 |  |
| 5831 | ENSG00000204843 |  |
| 5832 | ENSG00000181274 |  |
| 5833 | ENSG00000119661 |  |
| 5834 | ENSG00000099960 |  |
| 5835 | ENSG00000130489 |  |
| 5836 | ENSG00000082781 |  |
| 5837 | ENSG00000154719 |  |
| 5838 | ENSG00000107438 |  |
| 5839 | ENSG00000003400 |  |
| 5840 | ENSG00000138834 |  |
| 5841 | ENSG00000185133 |  |
| 5842 | ENSG00000131584 |  |
| 5843 | ENSG00000232850 |  |
| 5844 | ENSG00000149182 |  |
| 5845 | ENSG00000159461 |  |
| 5846 | ENSG00000268182 |  |
| 5847 | ENSG00000186918 |  |
| 5848 | ENSG00000197696 |  |
| 5849 | ENSG00000139515 |  |
| 5850 | ENSG00000122386 |  |
| 5851 | ENSG00000131668 |  |
| 5852 | ENSG00000161547 |  |
| 5853 | ENSG00000198445 |  |
| 5854 | ENSG00000070770 |  |
| 5855 | ENSG00000126432 |  |
| 5856 | ENSG00000080189 |  |
| 5857 | ENSG00000128536 |  |
| 5858 | ENSG00000186907 |  |
| 5859 | ENSG00000068137 |  |
| 5860 | ENSG00000204542 |  |
| 5861 | ENSG00000107742 |  |
| 5862 | ENSG00000005448 |  |
| 5863 | ENSG00000110057 |  |
| 5864 | ENSG00000169876 |  |
| 5865 | ENSG00000268000 |  |
| 5866 | ENSG00000105607 |  |
| 5867 | ENSG00000213859 |  |
| 5868 | ENSG00000105722 |  |
| 5869 | ENSG00000175283 |  |
| 5870 | ENSG00000179292 |  |
| 5871 | ENSG00000101441 |  |
| 5872 | ENSG00000143393 |  |
| 5873 | ENSG00000180176 |  |
| 5874 | ENSG00000104946 |  |
| 5875 | ENSG00000023608 |  |
| 5876 | ENSG00000132681 |  |
| 5877 | ENSG00000091181 |  |
| 5878 | ENSG00000166147 |  |
| 5879 | ENSG00000135925 |  |
| 5880 | ENSG00000188393 |  |
| 5881 | ENSG00000119681 |  |
| 5882 | ENSG00000104979 |  |
| 5883 | ENSG00000254614 |  |
| 5884 | ENSG00000184524 |  |
| 5885 | ENSG00000158747 |  |
| 5886 | ENSG00000187556 |  |
| 5887 | ENSG00000185885 |  |
| 5888 | ENSG00000105974 |  |
| 5889 | ENSG00000256100 |  |
| 5890 | ENSG00000159261 |  |
| 5891 | ENSG00000167186 |  |
| 5892 | ENSG00000221963 |  |
| 5893 | ENSG00000268302 |  |
| 5894 | ENSG00000132906 |  |
| 5895 | ENSG00000101327 |  |
| 5896 | ENSG00000166140 |  |
| 5897 | ENSG00000086696 |  |
| 5898 | ENSG00000133789 |  |
| 5899 | ENSG00000250317 |  |
| 5900 | ENSG00000249624 |  |
| 5901 | ENSG00000160075 |  |
| 5902 | ENSG00000141524 |  |
| 5903 | ENSG00000123562 |  |
| 5904 | ENSG00000149823 |  |
| 5905 | ENSG00000113302 |  |
| 5906 | ENSG00000254959 |  |
| 5907 | ENSG00000136404 |  |
| 5908 | ENSG00000134827 |  |
| 5909 | ENSG00000100079 |  |
| 5910 | ENSG00000176014 |  |
| 5911 | ENSG00000101331 |  |
| 5912 | ENSG00000205744 |  |
| 5913 | ENSG00000075426 |  |
| 5914 | ENSG00000101966 |  |
| 5915 | ENSG00000148459 |  |
| 5916 | ENSG00000169085 |  |
| 5917 | ENSG00000242715 |  |
| 5918 | ENSG00000127837 |  |
| 5919 | ENSG00000103852 |  |
| 5920 | ENSG00000121335 |  |
| 5921 | ENSG00000215298 |  |
| 5922 | ENSG00000141837 |  |
| 5923 | ENSG00000115255 |  |
| 5924 | ENSG00000260456 |  |
| 5925 | ENSG00000167637 |  |
| 5926 | ENSG00000118985 |  |
| 5927 | ENSG00000130940 |  |
| 5928 | ENSG00000163959 |  |
| 5929 | ENSG00000168993 |  |
| 5930 | ENSG00000058600 |  |
| 5931 | ENSG00000154447 |  |
| 5932 | ENSG00000213231 |  |
| 5933 | ENSG00000127191 |  |
| 5934 | ENSG00000172689 |  |
| 5935 | ENSG00000180921 |  |
| 5936 | ENSG00000268387 |  |
| 5937 | ENSG00000009335 |  |
| 5938 | ENSG00000177463 |  |
| 5939 | ENSG00000204475 |  |
| 5940 | ENSG00000205923 |  |
| 5941 | ENSG00000187479 |  |
| 5942 | ENSG00000155868 |  |
| 5943 | ENSG00000144567 |  |
| 5944 | ENSG00000215018 |  |
| 5945 | ENSG00000145882 |  |
| 5946 | ENSG00000104147 |  |
| 5947 | ENSG00000115523 |  |
| 5948 | ENSG00000102796 |  |
| 5949 | ENSG00000145920 |  |
| 5950 | ENSG00000065308 |  |
| 5951 | ENSG00000099204 |  |
| 5952 | ENSG00000114349 |  |
| 5953 | ENSG00000144485 |  |
| 5954 | ENSG00000131873 |  |
| 5955 | ENSG00000074800 |  |
| 5956 | ENSG00000223474 |  |
| 5957 | ENSG00000166965 |  |
| 5958 | ENSG00000132671 |  |
| 5959 | ENSG00000114023 |  |
| 5960 | ENSG00000268279 |  |
| 5961 | ENSG00000268173 |  |
| 5962 | ENSG00000171703 |  |
| 5963 | ENSG00000255346 |  |
| 5964 | ENSG00000139574 |  |
| 5965 | ENSG00000184428 |  |
| 5966 | ENSG00000007255 |  |
| 5967 | ENSG00000188004 |  |
| 5968 | ENSG00000154803 |  |
| 5969 | ENSG00000107796 |  |
| 5970 | ENSG00000218739 |  |
| 5971 | ENSG00000123364 |  |
| 5972 | ENSG00000142546 |  |
| 5973 | ENSG00000129911 |  |
| 5974 | ENSG00000124194 |  |
| 5975 | ENSG00000255819 |  |
| 5976 | ENSG00000164880 |  |
| 5977 | ENSG00000183864 |  |
| 5978 | ENSG00000120616 |  |
| 5979 | ENSG00000196924 |  |
| 5980 | ENSG00000117614 |  |
| 5981 | ENSG00000144130 |  |
| 5982 | ENSG00000078674 |  |
| 5983 | ENSG00000130590 |  |
| 5984 | ENSG00000162009 |  |
| 5985 | ENSG00000184995 |  |
| 5986 | ENSG00000180758 |  |
| 5987 | ENSG00000159023 |  |
| 5988 | ENSG00000165046 |  |
| 5989 | ENSG00000196498 |  |
| 5990 | ENSG00000225485 |  |
| 5991 | ENSG00000006638 |  |
| 5992 | ENSG00000182950 |  |
| 5993 | ENSG00000175832 |  |
| 5994 | ENSG00000267855 |  |
| 5995 | ENSG00000166165 |  |
| 5996 | ENSG00000138835 |  |
| 5997 | ENSG00000163918 |  |
| 5998 | ENSG00000177103 |  |
| 5999 | ENSG00000170312 |  |
| 6000 | ENSG00000176753 |  |
| 6001 | ENSG00000161570 |  |
| 6002 | ENSG00000100225 |  |
| 6003 | ENSG00000241404 |  |
| 6004 | ENSG00000221874 |  |
| 6005 | ENSG00000154978 |  |
| 6006 | ENSG00000182168 |  |
| 6007 | ENSG00000158292 |  |
| 6008 | ENSG00000177556 |  |
| 6009 | ENSG00000112559 |  |
| 6010 | ENSG00000156453 |  |
| 6011 | ENSG00000105258 |  |
| 6012 | ENSG00000171729 |  |
| 6013 | ENSG00000108784 |  |
| 6014 | ENSG00000268936 |  |
| 6015 | ENSG00000197147 |  |
| 6016 | ENSG00000125731 |  |
| 6017 | ENSG00000186106 |  |
| 6018 | ENSG00000175505 |  |
| 6019 | ENSG00000111262 |  |
| 6020 | ENSG00000196776 |  |
| 6021 | ENSG00000136048 |  |
| 6022 | ENSG00000103197 |  |
| 6023 | ENSG00000176155 |  |
| 6024 | ENSG00000198198 |  |
| 6025 | ENSG00000185305 |  |
| 6026 | ENSG00000160392 |  |
| 6027 | ENSG00000066136 |  |
| 6028 | ENSG00000119729 |  |
| 6029 | ENSG00000167100 |  |
| 6030 | ENSG00000171217 |  |
| 6031 | ENSG00000108106 |  |
| 6032 | ENSG00000171695 |  |
| 6033 | ENSG00000108819 |  |
| 6034 | ENSG00000171608 |  |
| 6035 | ENSG00000173366 |  |
| 6036 | ENSG00000205426 |  |
| 6037 | ENSG00000138942 |  |
| 6038 | ENSG00000172890 |  |
| 6039 | ENSG00000078699 |  |
| 6040 | ENSG00000150756 |  |
| 6041 | ENSG00000197063 |  |
| 6042 | ENSG00000149091 |  |
| 6043 | ENSG00000025770 |  |
| 6044 | ENSG00000269404 |  |
| 6045 | ENSG00000164105 |  |
| 6046 | ENSG00000130643 |  |
| 6047 | ENSG00000110852 |  |
| 6048 | ENSG00000115844 |  |
| 6049 | ENSG00000160216 |  |
| 6050 | ENSG00000125618 |  |
| 6051 | ENSG00000139531 |  |
| 6052 | ENSG00000123349 |  |
| 6053 | ENSG00000071575 |  |
| 6054 | ENSG00000179912 |  |
| 6055 | ENSG00000137312 |  |
| 6056 | ENSG00000183248 |  |
| 6057 | ENSG00000268484 |  |
| 6058 | ENSG00000029534 |  |
| 6059 | ENSG00000268656 |  |
| 6060 | ENSG00000267110 |  |
| 6061 | ENSG00000126217 |  |
| 6062 | ENSG00000168014 |  |
| 6063 | ENSG00000131748 |  |
| 6064 | ENSG00000003989 |  |
| 6065 | ENSG00000168685 |  |
| 6066 | ENSG00000220891 |  |
| 6067 | ENSG00000143341 |  |
| 6068 | ENSG00000255245 |  |
| 6069 | ENSG00000131042 |  |
| 6070 | ENSG00000163251 |  |
| 6071 | ENSG00000196878 |  |
| 6072 | ENSG00000203667 |  |
| 6073 | ENSG00000160714 |  |
| 6074 | ENSG00000179930 |  |
| 6075 | ENSG00000181039 |  |
| 6076 | ENSG00000008128 |  |
| 6077 | ENSG00000169826 |  |
| 6078 | ENSG00000086062 |  |
| 6079 | ENSG00000183292 |  |
| 6080 | ENSG00000143257 |  |
| 6081 | ENSG00000124171 |  |
| 6082 | ENSG00000135407 |  |
| 6083 | ENSG00000145907 |  |
| 6084 | ENSG00000205791 |  |
| 6085 | ENSG00000244474 |  |
| 6086 | ENSG00000170892 |  |
| 6087 | ENSG00000204950 |  |
| 6088 | ENSG00000155265 |  |
| 6089 | ENSG00000196557 |  |
| 6090 | ENSG00000166183 |  |
| 6091 | ENSG00000249087 |  |
| 6092 | ENSG00000171302 |  |
| 6093 | ENSG00000180929 |  |
| 6094 | ENSG00000198355 |  |
| 6095 | ENSG00000148090 |  |
| 6096 | ENSG00000130766 |  |
| 6097 | ENSG00000105698 |  |
| 6098 | ENSG00000117118 |  |
| 6099 | ENSG00000124839 |  |
| 6100 | ENSG00000168081 |  |
| 6101 | ENSG00000133069 |  |
| 6102 | ENSG00000143801 |  |
| 6103 | ENSG00000124222 |  |
| 6104 | ENSG00000090565 |  |
| 6105 | ENSG00000115866 |  |
| 6106 | ENSG00000164850 |  |
| 6107 | ENSG00000186416 |  |
| 6108 | ENSG00000175756 |  |
| 6109 | ENSG00000161714 |  |
| 6110 | ENSG00000054179 |  |
| 6111 | ENSG00000156873 |  |
| 6112 | ENSG00000186469 |  |
| 6113 | ENSG00000162241 |  |
| 6114 | ENSG00000101412 |  |
| 6115 | ENSG00000126709 |  |
| 6116 | ENSG00000177169 |  |
| 6117 | ENSG00000100739 |  |
| 6118 | ENSG00000204538 |  |
| 6119 | ENSG00000162062 |  |
| 6120 | ENSG00000134532 |  |
| 6121 | ENSG00000213002 |  |
| 6122 | ENSG00000261459 |  |
| 6123 | ENSG00000134020 |  |
| 6124 | ENSG00000172534 |  |
| 6125 | ENSG00000141562 |  |
| 6126 | ENSG00000047936 |  |
| 6127 | ENSG00000108309 |  |
| 6128 | ENSG00000131374 |  |
| 6129 | ENSG00000121281 |  |
| 6130 | ENSG00000103056 |  |
| 6131 | ENSG00000129315 |  |
| 6132 | ENSG00000132688 |  |
| 6133 | ENSG00000006451 |  |
| 6134 | ENSG00000155545 |  |
| 6135 | ENSG00000186832 |  |
| 6136 | ENSG00000154814 |  |
| 6137 | ENSG00000111328 |  |
| 6138 | ENSG00000104081 |  |
| 6139 | ENSG00000232748 |  |
| 6140 | ENSG00000142530 |  |
| 6141 | ENSG00000197106 |  |
| 6142 | ENSG00000115414 |  |
| 6143 | ENSG00000128298 |  |
| 6144 | ENSG00000119559 |  |
| 6145 | ENSG00000214844 |  |
| 6146 | ENSG00000146063 |  |
| 6147 | ENSG00000166925 |  |
| 6148 | ENSG00000105726 |  |
| 6149 | ENSG00000075275 |  |
| 6150 | ENSG00000068650 |  |
| 6151 | ENSG00000268146 |  |
| 6152 | ENSG00000107831 |  |
| 6153 | ENSG00000142623 |  |
| 6154 | ENSG00000111775 |  |
| 6155 | ENSG00000206262 |  |
| 6156 | ENSG00000168137 |  |
| 6157 | ENSG00000177700 |  |
| 6158 | ENSG00000101624 |  |
| 6159 | ENSG00000168291 |  |
| 6160 | ENSG00000164897 |  |
| 6161 | ENSG00000168876 |  |
| 6162 | ENSG00000134262 |  |
| 6163 | ENSG00000128849 |  |
| 6164 | ENSG00000072310 |  |
| 6165 | ENSG00000012660 |  |
| 6166 | ENSG00000162426 |  |
| 6167 | ENSG00000187456 |  |
| 6168 | ENSG00000251258 |  |
| 6169 | ENSG00000183598 |  |
| 6170 | ENSG00000179862 |  |
| 6171 | ENSG00000182218 |  |
| 6172 | ENSG00000131634 |  |
| 6173 | ENSG00000162398 |  |
| 6174 | ENSG00000186007 |  |
| 6175 | ENSG00000273025 |  |
| 6176 | ENSG00000253459 |  |
| 6177 | ENSG00000217702 |  |
| 6178 | ENSG00000148700 |  |
| 6179 | ENSG00000172197 |  |
| 6180 | ENSG00000188064 |  |
| 6181 | ENSG00000151726 |  |
| 6182 | ENSG00000109113 |  |
| 6183 | ENSG00000270800 |  |
| 6184 | ENSG00000162076 |  |
| 6185 | ENSG00000187824 |  |
| 6186 | ENSG00000103426 |  |
| 6187 | ENSG00000169717 |  |
| 6188 | ENSG00000084090 |  |
| 6189 | ENSG00000267545 |  |
| 6190 | ENSG00000196576 |  |
| 6191 | ENSG00000136490 |  |
| 6192 | ENSG00000183709 |  |
| 6193 | ENSG00000130545 |  |
| 6194 | ENSG00000175398 |  |
| 6195 | ENSG00000066557 |  |
| 6196 | ENSG00000169548 |  |
| 6197 | ENSG00000162772 |  |
| 6198 | ENSG00000222018 |  |
| 6199 | ENSG00000004799 |  |
| 6200 | ENSG00000100461 |  |
| 6201 | ENSG00000010295 |  |
| 6202 | ENSG00000206260 |  |
| 6203 | ENSG00000143590 |  |
| 6204 | ENSG00000181045 |  |
| 6205 | ENSG00000110848 |  |
| 6206 | ENSG00000126218 |  |
| 6207 | ENSG00000197136 |  |
| 6208 | ENSG00000105287 |  |
| 6209 | ENSG00000183576 |  |
| 6210 | ENSG00000123689 |  |
| 6211 | ENSG00000163516 |  |
| 6212 | ENSG00000169857 |  |
| 6213 | ENSG00000114956 |  |
| 6214 | ENSG00000143851 |  |
| 6215 | ENSG00000169885 |  |
| 6216 | ENSG00000260342 |  |
| 6217 | ENSG00000157540 |  |
| 6218 | ENSG00000185739 |  |
| 6219 | ENSG00000178171 |  |
| 6220 | ENSG00000083457 |  |
| 6221 | ENSG00000087365 |  |
| 6222 | ENSG00000155329 |  |
| 6223 | ENSG00000178997 |  |
| 6224 | ENSG00000145703 |  |
| 6225 | ENSG00000141720 |  |
| 6226 | ENSG00000156535 |  |
| 6227 | ENSG00000180066 |  |
| 6228 | ENSG00000074935 |  |
| 6229 | ENSG00000179085 |  |
| 6230 | ENSG00000109452 |  |
| 6231 | ENSG00000243477 |  |
| 6232 | ENSG00000167034 |  |
| 6233 | ENSG00000185442 |  |
| 6234 | ENSG00000060069 |  |
| 6235 | ENSG00000264187 |  |
| 6236 | ENSG00000065320 |  |
| 6237 | ENSG00000065057 |  |
| 6238 | ENSG00000259066 |  |
| 6239 | ENSG00000068001 |  |
| 6240 | ENSG00000166482 |  |
| 6241 | ENSG00000197980 |  |
| 6242 | ENSG00000133816 |  |
| 6243 | ENSG00000196167 |  |
| 6244 | ENSG00000205086 |  |
| 6245 | ENSG00000171861 |  |
| 6246 | ENSG00000059758 |  |
| 6247 | ENSG00000167617 |  |
| 6248 | ENSG00000165898 |  |
| 6249 | ENSG00000167157 |  |
| 6250 | ENSG00000108528 |  |
| 6251 | ENSG00000025156 |  |
| 6252 | ENSG00000148339 |  |
| 6253 | ENSG00000018625 |  |
| 6254 | ENSG00000119508 |  |
| 6255 | ENSG00000099968 |  |
| 6256 | ENSG00000109270 |  |
| 6257 | ENSG00000136379 |  |
| 6258 | ENSG00000166268 |  |
| 6259 | ENSG00000164185 |  |
| 6260 | ENSG00000095752 |  |
| 6261 | ENSG00000267845 |  |
| 6262 | ENSG00000100077 |  |
| 6263 | ENSG00000121073 |  |
| 6264 | ENSG00000180198 |  |
| 6265 | ENSG00000103061 |  |
| 6266 | ENSG00000188042 |  |
| 6267 | ENSG00000172057 |  |
| 6268 | ENSG00000205022 |  |
| 6269 | ENSG00000154945 |  |
| 6270 | ENSG00000101104 |  |
| 6271 | ENSG00000150593 |  |
| 6272 | ENSG00000137571 |  |
| 6273 | ENSG00000117335 |  |
| 6274 | ENSG00000230055 |  |
| 6275 | ENSG00000178951 |  |
| 6276 | ENSG00000084072 |  |
| 6277 | ENSG00000182749 |  |
| 6278 | ENSG00000187475 |  |
| 6279 | ENSG00000176401 |  |
| 6280 | ENSG00000142615 |  |
| 6281 | ENSG00000157343 |  |
| 6282 | ENSG00000136153 |  |
| 6283 | ENSG00000134323 |  |
| 6284 | ENSG00000197635 |  |
| 6285 | ENSG00000188372 |  |
| 6286 | ENSG00000101203 |  |
| 6287 | ENSG00000126246 |  |
| 6288 | ENSG00000205076 |  |
| 6289 | ENSG00000197217 |  |
| 6290 | ENSG00000188760 |  |
| 6291 | ENSG00000168385 |  |
| 6292 | ENSG00000107341 |  |
| 6293 | ENSG00000160214 |  |
| 6294 | ENSG00000169221 |  |
| 6295 | ENSG00000205047 |  |
| 6296 | ENSG00000185745 |  |
| 6297 | ENSG00000102445 |  |
| 6298 | ENSG00000160339 |  |
| 6299 | ENSG00000110237 |  |
| 6300 | ENSG00000164867 |  |
| 6301 | ENSG00000175711 |  |
| 6302 | ENSG00000233276 |  |
| 6303 | ENSG00000125046 |  |
| 6304 | ENSG00000125534 |  |
| 6305 | ENSG00000107554 |  |
| 6306 | ENSG00000138172 |  |
| 6307 | ENSG00000117408 |  |
| 6308 | ENSG00000173535 |  |
| 6309 | ENSG00000116497 |  |
| 6310 | ENSG00000205323 |  |
| 6311 | ENSG00000220205 |  |
| 6312 | ENSG00000109321 |  |
| 6313 | ENSG00000114812 |  |
| 6314 | ENSG00000187860 |  |
| 6315 | ENSG00000158987 |  |
| 6316 | ENSG00000117410 |  |
| 6317 | ENSG00000131966 |  |
| 6318 | ENSG00000146540 |  |
| 6319 | ENSG00000166912 |  |
| 6320 | ENSG00000141994 |  |
| 6321 | ENSG00000188130 |  |
| 6322 | ENSG00000105655 |  |
| 6323 | ENSG00000135926 |  |
| 6324 | ENSG00000178279 |  |
| 6325 | ENSG00000197782 |  |
| 6326 | ENSG00000136560 |  |
| 6327 | ENSG00000108799 |  |
| 6328 | ENSG00000031081 |  |
| 6329 | ENSG00000156140 |  |
| 6330 | ENSG00000136816 |  |
| 6331 | ENSG00000167774 |  |
| 6332 | ENSG00000111012 |  |
| 6333 | ENSG00000174231 |  |
| 6334 | ENSG00000253719 |  |
| 6335 | ENSG00000175215 |  |
| 6336 | ENSG00000100121 |  |
| 6337 | ENSG00000197057 |  |
| 6338 | ENSG00000204335 |  |
| 6339 | ENSG00000150045 |  |
| 6340 | ENSG00000073792 |  |
| 6341 | ENSG00000177606 |  |
| 6342 | ENSG00000147862 |  |
| 6343 | ENSG00000033170 |  |
| 6344 | ENSG00000184956 |  |
| 6345 | ENSG00000198382 |  |
| 6346 | ENSG00000144355 |  |
| 6347 | ENSG00000197353 |  |
| 6348 | ENSG00000184828 |  |
| 6349 | ENSG00000125910 |  |
| 6350 | ENSG00000165480 |  |
| 6351 | ENSG00000165943 |  |
| 6352 | ENSG00000162236 |  |
| 6353 | ENSG00000249008 |  |
| 6354 | ENSG00000114796 |  |
| 6355 | ENSG00000269120 |  |
| 6356 | ENSG00000118004 |  |
| 6357 | ENSG00000125967 |  |
| 6358 | ENSG00000161677 |  |
| 6359 | ENSG00000066654 |  |
| 6360 | ENSG00000163159 |  |
| 6361 | ENSG00000254858 |  |
| 6362 | ENSG00000163155 |  |
| 6363 | ENSG00000073910 |  |
| 6364 | ENSG00000168096 |  |
| 6365 | ENSG00000136689 |  |
| 6366 | ENSG00000149806 |  |
| 6367 | ENSG00000186517 |  |
| 6368 | ENSG00000161981 |  |
| 6369 | ENSG00000073756 |  |
| 6370 | ENSG00000108509 |  |
| 6371 | ENSG00000106546 |  |
| 6372 | ENSG00000160294 |  |
| 6373 | ENSG00000152611 |  |
| 6374 | ENSG00000165801 |  |
| 6375 | ENSG00000104889 |  |
| 6376 | ENSG00000118046 |  |
| 6377 | ENSG00000204316 |  |
| 6378 | ENSG00000143224 |  |
| 6379 | ENSG00000105229 |  |
| 6380 | ENSG00000004975 |  |
| 6381 | ENSG00000029364 |  |
| 6382 | ENSG00000018280 |  |
| 6383 | ENSG00000204653 |  |
| 6384 | ENSG00000005075 |  |
| 6385 | ENSG00000185332 |  |
| 6386 | ENSG00000143889 |  |
| 6387 | ENSG00000214087 |  |
| 6388 | ENSG00000132600 |  |
| 6389 | ENSG00000164930 |  |
| 6390 | ENSG00000258839 |  |
| 6391 | ENSG00000009413 |  |
| 6392 | ENSG00000168502 |  |
| 6393 | ENSG00000181625 |  |
| 6394 | ENSG00000133454 |  |
| 6395 | ENSG00000100412 |  |
| 6396 | ENSG00000149925 |  |
| 6397 | ENSG00000039523 |  |
| 6398 | ENSG00000137770 |  |
| 6399 | ENSG00000110944 |  |
| 6400 | ENSG00000136457 |  |
| 6401 | ENSG00000158428 |  |
| 6402 | ENSG00000149187 |  |
| 6403 | ENSG00000183723 |  |
| 6404 | ENSG00000182500 |  |
| 6405 | ENSG00000145569 |  |
| 6406 | ENSG00000185379 |  |
| 6407 | ENSG00000103489 |  |
| 6408 | ENSG00000133028 |  |
| 6409 | ENSG00000166341 |  |
| 6410 | ENSG00000103227 |  |
| 6411 | ENSG00000033100 |  |
| 6412 | ENSG00000135702 |  |
| 6413 | ENSG00000124615 |  |
| 6414 | ENSG00000222033 |  |
| 6415 | ENSG00000134108 |  |
| 6416 | ENSG00000130517 |  |
| 6417 | ENSG00000137936 |  |
| 6418 | ENSG00000185883 |  |
| 6419 | ENSG00000168569 |  |
| 6420 | ENSG00000139726 |  |
| 6421 | ENSG00000198920 |  |
| 6422 | ENSG00000168421 |  |
| 6423 | ENSG00000089820 |  |
| 6424 | ENSG00000167653 |  |
| 6425 | ENSG00000054148 |  |
| 6426 | ENSG00000110429 |  |
| 6427 | ENSG00000181652 |  |
| 6428 | ENSG00000089902 |  |
| 6429 | ENSG00000157184 |  |
| 6430 | ENSG00000198218 |  |
| 6431 | ENSG00000140379 |  |
| 6432 | ENSG00000121316 |  |
| 6433 | ENSG00000196449 |  |
| 6434 | ENSG00000072818 |  |
| 6435 | ENSG00000177981 |  |
| 6436 | ENSG00000128487 |  |
| 6437 | ENSG00000161179 |  |
| 6438 | ENSG00000179588 |  |
| 6439 | ENSG00000212993 |  |
| 6440 | ENSG00000143367 |  |
| 6441 | ENSG00000269650 |  |
| 6442 | ENSG00000155093 |  |
| 6443 | ENSG00000169618 |  |
| 6444 | ENSG00000167674 |  |
| 6445 | ENSG00000160584 |  |
| 6446 | ENSG00000189403 |  |
| 6447 | ENSG00000137200 |  |
| 6448 | ENSG00000170075 |  |
| 6449 | ENSG00000248835 |  |
| 6450 | ENSG00000010282 |  |
| 6451 | ENSG00000143614 |  |
| 6452 | ENSG00000108292 |  |
| 6453 | ENSG00000185404 |  |
| 6454 | ENSG00000173163 |  |
| 6455 | ENSG00000161692 |  |
| 6456 | ENSG00000183150 |  |
| 6457 | ENSG00000163075 |  |
| 6458 | ENSG00000157483 |  |
| 6459 | ENSG00000235568 |  |
| 6460 | ENSG00000099381 |  |
| 6461 | ENSG00000104941 |  |
| 6462 | ENSG00000132361 |  |
| 6463 | ENSG00000127423 |  |
| 6464 | ENSG00000183562 |  |
| 6465 | ENSG00000174871 |  |
| 6466 | ENSG00000174669 |  |
| 6467 | ENSG00000115875 |  |
| 6468 | ENSG00000160396 |  |
| 6469 | ENSG00000116396 |  |
| 6470 | ENSG00000110876 |  |
| 6471 | ENSG00000260362 |  |
| 6472 | ENSG00000074964 |  |
| 6473 | ENSG00000154153 |  |
| 6474 | ENSG00000166800 |  |
| 6475 | ENSG00000214556 |  |
| 6476 | ENSG00000137869 |  |
| 6477 | ENSG00000111640 |  |
| 6478 | ENSG00000139971 |  |
| 6479 | ENSG00000065802 |  |
| 6480 | ENSG00000181788 |  |
| 6481 | ENSG00000171552 |  |
| 6482 | ENSG00000126705 |  |
| 6483 | ENSG00000042493 |  |
| 6484 | ENSG00000174083 |  |
| 6485 | ENSG00000269549 |  |
| 6486 | ENSG00000141314 |  |
| 6487 | ENSG00000176788 |  |
| 6488 | ENSG00000180638 |  |
| 6489 | ENSG00000162852 |  |
| 6490 | ENSG00000099800 |  |
| 6491 | ENSG00000169926 |  |
| 6492 | ENSG00000130368 |  |
| 6493 | ENSG00000236699 |  |
| 6494 | ENSG00000106799 |  |
| 6495 | ENSG00000185022 |  |
| 6496 | ENSG00000141580 |  |
| 6497 | ENSG00000130363 |  |
| 6498 | ENSG00000206013 |  |
| 6499 | ENSG00000235878 |  |
| 6500 | ENSG00000258987 |  |
| 6501 | ENSG00000224389 |  |
| 6502 | ENSG00000132142 |  |
| 6503 | ENSG00000197061 |  |
| 6504 | ENSG00000169908 |  |
| 6505 | ENSG00000197599 |  |
| 6506 | ENSG00000010438 |  |
| 6507 | ENSG00000154319 |  |
| 6508 | ENSG00000185798 |  |
| 6509 | ENSG00000186868 |  |
| 6510 | ENSG00000087237 |  |
| 6511 | ENSG00000184985 |  |
| 6512 | ENSG00000129226 |  |
| 6513 | ENSG00000167967 |  |
| 6514 | ENSG00000187634 |  |
| 6515 | ENSG00000101447 |  |
| 6516 | ENSG00000141232 |  |
| 6517 | ENSG00000197826 |  |
| 6518 | ENSG00000104969 |  |
| 6519 | ENSG00000187498 |  |
| 6520 | ENSG00000137497 |  |
| 6521 | ENSG00000205899 |  |
| 6522 | ENSG00000204356 |  |
| 6523 | ENSG00000140688 |  |
| 6524 | ENSG00000204344 |  |
| 6525 | ENSG00000110108 |  |
| 6526 | ENSG00000187013 |  |
| 6527 | ENSG00000149792 |  |
| 6528 | ENSG00000070047 |  |
| 6529 | ENSG00000158769 |  |
| 6530 | ENSG00000169692 |  |
| 6531 | ENSG00000103707 |  |
| 6532 | ENSG00000168517 |  |
| 6533 | ENSG00000164332 |  |
| 6534 | ENSG00000126602 |  |
| 6535 | ENSG00000183742 |  |
| 6536 | ENSG00000107281 |  |
| 6537 | ENSG00000111843 |  |
| 6538 | ENSG00000128016 |  |
| 6539 | ENSG00000204262 |  |
| 6540 | ENSG00000180357 |  |
| 6541 | ENSG00000103502 |  |
| 6542 | ENSG00000185499 |  |
| 6543 | ENSG00000072182 |  |
| 6544 | ENSG00000126749 |  |
| 6545 | ENSG00000117419 |  |
| 6546 | ENSG00000122359 |  |
| 6547 | ENSG00000010932 |  |
| 6548 | ENSG00000140505 |  |
| 6549 | ENSG00000268275 |  |
| 6550 | ENSG00000102974 |  |
| 6551 | ENSG00000092607 |  |
| 6552 | ENSG00000187783 |  |
| 6553 | ENSG00000182040 |  |
| 6554 | ENSG00000088038 |  |
| 6555 | ENSG00000128928 |  |
| 6556 | ENSG00000180035 |  |
| 6557 | ENSG00000170266 |  |
| 6558 | ENSG00000212123 |  |
| 6559 | ENSG00000111678 |  |
| 6560 | ENSG00000182196 |  |
| 6561 | ENSG00000176490 |  |
| 6562 | ENSG00000170275 |  |
| 6563 | ENSG00000143624 |  |
| 6564 | ENSG00000072134 |  |
| 6565 | ENSG00000091542 |  |
| 6566 | ENSG00000101421 |  |
| 6567 | ENSG00000170525 |  |
| 6568 | ENSG00000255384 |  |
| 6569 | ENSG00000099812 |  |
| 6570 | ENSG00000115946 |  |
| 6571 | ENSG00000067082 |  |
| 6572 | ENSG00000183421 |  |
| 6573 | ENSG00000171989 |  |
| 6574 | ENSG00000188529 |  |
| 6575 | ENSG00000157303 |  |
| 6576 | ENSG00000169031 |  |
| 6577 | ENSG00000008513 |  |
| 6578 | ENSG00000168374 |  |
| 6579 | ENSG00000187134 |  |
| 6580 | ENSG00000197019 |  |
| 6581 | ENSG00000116586 |  |
| 6582 | ENSG00000107317 |  |
| 6583 | ENSG00000185730 |  |
| 6584 | ENSG00000147465 |  |
| 6585 | ENSG00000134242 |  |
| 6586 | ENSG00000142192 |  |
| 6587 | ENSG00000258474 |  |
| 6588 | ENSG00000116478 |  |
| 6589 | ENSG00000187838 |  |
| 6590 | ENSG00000106868 |  |
| 6591 | ENSG00000106327 |  |
| 6592 | ENSG00000141456 |  |
| 6593 | ENSG00000228919 |  |
| 6594 | ENSG00000158615 |  |
| 6595 | ENSG00000167768 |  |
| 6596 | ENSG00000159224 |  |
| 6597 | ENSG00000072657 |  |
| 6598 | ENSG00000171345 |  |
| 6599 | ENSG00000135097 |  |
| 6600 | ENSG00000145375 |  |
| 6601 | ENSG00000143776 |  |
| 6602 | ENSG00000157654 |  |
| 6603 | ENSG00000062485 |  |
| 6604 | ENSG00000158865 |  |
| 6605 | ENSG00000104814 |  |
| 6606 | ENSG00000177479 |  |
| 6607 | ENSG00000119977 |  |
| 6608 | ENSG00000142541 |  |
| 6609 | ENSG00000022556 |  |
| 6610 | ENSG00000128585 |  |
| 6611 | ENSG00000010361 |  |
| 6612 | ENSG00000115363 |  |
| 6613 | ENSG00000111679 |  |
| 6614 | ENSG00000100105 |  |
| 6615 | ENSG00000267882 |  |
| 6616 | ENSG00000232056 |  |
| 6617 | ENSG00000135736 |  |
| 6618 | ENSG00000100003 |  |
| 6619 | ENSG00000181847 |  |
| 6620 | ENSG00000111728 |  |
| 6621 | ENSG00000204613 |  |
| 6622 | ENSG00000168916 |  |
| 6623 | ENSG00000081237 |  |
| 6624 | ENSG00000124529 |  |
| 6625 | ENSG00000203546 |  |
| 6626 | ENSG00000152207 |  |
| 6627 | ENSG00000064309 |  |
| 6628 | ENSG00000102524 |  |
| 6629 | ENSG00000175826 |  |
| 6630 | ENSG00000269302 |  |
| 6631 | ENSG00000197586 |  |
| 6632 | ENSG00000083838 |  |
| 6633 | ENSG00000185669 |  |
| 6634 | ENSG00000251692 |  |
| 6635 | ENSG00000110492 |  |
| 6636 | ENSG00000140319 |  |
| 6637 | ENSG00000175029 |  |
| 6638 | ENSG00000158859 |  |
| 6639 | ENSG00000103599 |  |
| 6640 | ENSG00000182700 |  |
| 6641 | ENSG00000137673 |  |
| 6642 | ENSG00000105792 |  |
| 6643 | ENSG00000175538 |  |
| 6644 | ENSG00000215912 |  |
| 6645 | ENSG00000115596 |  |
| 6646 | ENSG00000120913 |  |
| 6647 | ENSG00000172262 |  |
| 6648 | ENSG00000127527 |  |
| 6649 | ENSG00000198353 |  |
| 6650 | ENSG00000268472 |  |
| 6651 | ENSG00000029725 |  |
| 6652 | ENSG00000114923 |  |
| 6653 | ENSG00000052749 |  |
| 6654 | ENSG00000106538 |  |
| 6655 | ENSG00000164691 |  |
| 6656 | ENSG00000145014 |  |
| 6657 | ENSG00000183558 |  |
| 6658 | ENSG00000176742 |  |
| 6659 | ENSG00000254402 |  |
| 6660 | ENSG00000116350 |  |
| 6661 | ENSG00000198431 |  |
| 6662 | ENSG00000198546 |  |
| 6663 | ENSG00000183579 |  |
| 6664 | ENSG00000150750 |  |
| 6665 | ENSG00000038274 |  |
| 6666 | ENSG00000248333 |  |
| 6667 | ENSG00000104915 |  |
| 6668 | ENSG00000178096 |  |
| 6669 | ENSG00000102001 |  |
| 6670 | ENSG00000103196 |  |
| 6671 | ENSG00000130508 |  |
| 6672 | ENSG00000173692 |  |
| 6673 | ENSG00000127377 |  |
| 6674 | ENSG00000166816 |  |
| 6675 | ENSG00000187840 |  |
| 6676 | ENSG00000184436 |  |
| 6677 | ENSG00000213977 |  |
| 6678 | ENSG00000099949 |  |
| 6679 | ENSG00000171346 |  |
| 6680 | ENSG00000160191 |  |
| 6681 | ENSG00000213930 |  |
| 6682 | ENSG00000136206 |  |
| 6683 | ENSG00000090971 |  |
| 6684 | ENSG00000007314 |  |
| 6685 | ENSG00000114554 |  |
| 6686 | ENSG00000170385 |  |
| 6687 | ENSG00000267710 |  |
| 6688 | ENSG00000160202 |  |
| 6689 | ENSG00000251537 |  |
| 6690 | ENSG00000054983 |  |
| 6691 | ENSG00000105197 |  |
| 6692 | ENSG00000134184 |  |
| 6693 | ENSG00000165113 |  |
| 6694 | ENSG00000072694 |  |
| 6695 | ENSG00000198356 |  |
| 6696 | ENSG00000153317 |  |
| 6697 | ENSG00000140488 |  |
| 6698 | ENSG00000095787 |  |
| 6699 | ENSG00000259003 |  |
| 6700 | ENSG00000137875 |  |
| 6701 | ENSG00000164442 |  |
| 6702 | ENSG00000120889 |  |
| 6703 | ENSG00000179041 |  |
| 6704 | ENSG00000164822 |  |
| 6705 | ENSG00000140443 |  |
| 6706 | ENSG00000127481 |  |
| 6707 | ENSG00000143556 |  |
| 6708 | ENSG00000169992 |  |
| 6709 | ENSG00000183762 |  |
| 6710 | ENSG00000215306 |  |
| 6711 | ENSG00000136536 |  |
| 6712 | ENSG00000168591 |  |
| 6713 | ENSG00000108433 |  |
| 6714 | ENSG00000162068 |  |
| 6715 | ENSG00000100106 |  |
| 6716 | ENSG00000215717 |  |
| 6717 | ENSG00000239332 |  |
| 6718 | ENSG00000158050 |  |
| 6719 | ENSG00000211456 |  |
| 6720 | ENSG00000114446 |  |
| 6721 | ENSG00000169403 |  |
| 6722 | ENSG00000175664 |  |
| 6723 | ENSG00000163468 |  |
| 6724 | ENSG00000103254 |  |
| 6725 | ENSG00000180098 |  |
| 6726 | ENSG00000262302 |  |
| 6727 | ENSG00000011332 |  |
| 6728 | ENSG00000183828 |  |
| 6729 | ENSG00000271810 |  |
| 6730 | ENSG00000176261 |  |
| 6731 | ENSG00000132768 |  |
| 6732 | ENSG00000140873 |  |
| 6733 | ENSG00000101074 |  |
| 6734 | ENSG00000108039 |  |
| 6735 | ENSG00000148346 |  |
| 6736 | ENSG00000147457 |  |
| 6737 | ENSG00000119231 |  |
| 6738 | ENSG00000099942 |  |
| 6739 | ENSG00000110274 |  |
| 6740 | ENSG00000158406 |  |
| 6741 | ENSG00000130584 |  |
| 6742 | ENSG00000140575 |  |
| 6743 | ENSG00000129460 |  |
| 6744 | ENSG00000140553 |  |
| 6745 | ENSG00000072952 |  |
| 6746 | ENSG00000177432 |  |
| 6747 | ENSG00000178104 |  |
| 6748 | ENSG00000268324 |  |
| 6749 | ENSG00000143815 |  |
| 6750 | ENSG00000100348 |  |
| 6751 | ENSG00000144029 |  |
| 6752 | ENSG00000149761 |  |
| 6753 | ENSG00000068976 |  |
| 6754 | ENSG00000137100 |  |
| 6755 | ENSG00000255641 |  |
| 6756 | ENSG00000131462 |  |
| 6757 | ENSG00000250424 |  |
| 6758 | ENSG00000113657 |  |
| 6759 | ENSG00000178921 |  |
| 6760 | ENSG00000171403 |  |
| 6761 | ENSG00000007545 |  |
| 6762 | ENSG00000135074 |  |
| 6763 | ENSG00000163485 |  |
| 6764 | ENSG00000174109 |  |
| 6765 | ENSG00000215375 |  |
| 6766 | ENSG00000103035 |  |
| 6767 | ENSG00000224997 |  |
| 6768 | ENSG00000141854 |  |
| 6769 | ENSG00000135535 |  |
| 6770 | ENSG00000196812 |  |
| 6771 | ENSG00000140983 |  |
| 6772 | ENSG00000164919 |  |
| 6773 | ENSG00000140368 |  |
| 6774 | ENSG00000019102 |  |
| 6775 | ENSG00000183048 |  |
| 6776 | ENSG00000006194 |  |
| 6777 | ENSG00000171813 |  |
| 6778 | ENSG00000076003 |  |
| 6779 | ENSG00000178814 |  |
| 6780 | ENSG00000179407 |  |
| 6781 | ENSG00000177764 |  |
| 6782 | ENSG00000158014 |  |
| 6783 | ENSG00000130640 |  |
| 6784 | ENSG00000204539 |  |
| 6785 | ENSG00000152944 |  |
| 6786 | ENSG00000006555 |  |
| 6787 | ENSG00000130294 |  |
| 6788 | ENSG00000269808 |  |
| 6789 | ENSG00000148411 |  |
| 6790 | ENSG00000154380 |  |
| 6791 | ENSG00000187135 |  |
| 6792 | ENSG00000111732 |  |
| 6793 | ENSG00000103507 |  |
| 6794 | ENSG00000100614 |  |
| 6795 | ENSG00000160551 |  |
| 6796 | ENSG00000198366 |  |
| 6797 | ENSG00000142046 |  |
| 6798 | ENSG00000135363 |  |
| 6799 | ENSG00000187837 |  |
| 6800 | ENSG00000065243 |  |
| 6801 | ENSG00000174844 |  |
| 6802 | ENSG00000166971 |  |
| 6803 | ENSG00000258315 |  |
| 6804 | ENSG00000167525 |  |
| 6805 | ENSG00000172992 |  |
| 6806 | ENSG00000170145 |  |
| 6807 | ENSG00000106948 |  |
| 6808 | ENSG00000124228 |  |
| 6809 | ENSG00000079462 |  |
| 6810 | ENSG00000135245 |  |
| 6811 | ENSG00000143494 |  |
| 6812 | ENSG00000025434 |  |
| 6813 | ENSG00000090661 |  |
| 6814 | ENSG00000169955 |  |
| 6815 | ENSG00000187555 |  |
| 6816 | ENSG00000100605 |  |
| 6817 | ENSG00000165424 |  |
| 6818 | ENSG00000141736 |  |
| 6819 | ENSG00000120458 |  |
| 6820 | ENSG00000183484 |  |
| 6821 | ENSG00000125730 |  |
| 6822 | ENSG00000227500 |  |
| 6823 | ENSG00000205106 |  |
| 6824 | ENSG00000011132 |  |
| 6825 | ENSG00000066933 |  |
| 6826 | ENSG00000169962 |  |
| 6827 | ENSG00000171456 |  |
| 6828 | ENSG00000174950 |  |
| 6829 | ENSG00000120254 |  |
| 6830 | ENSG00000248871 |  |
| 6831 | ENSG00000111490 |  |
| 6832 | ENSG00000162591 |  |
| 6833 | ENSG00000132334 |  |
| 6834 | ENSG00000174405 |  |
| 6835 | ENSG00000134986 |  |
| 6836 | ENSG00000162419 |  |
| 6837 | ENSG00000141338 |  |
| 6838 | ENSG00000196372 |  |
| 6839 | ENSG00000154118 |  |
| 6840 | ENSG00000100065 |  |
| 6841 | ENSG00000023330 |  |
| 6842 | ENSG00000163874 |  |
| 6843 | ENSG00000159197 |  |
| 6844 | ENSG00000197798 |  |
| 6845 | ENSG00000148737 |  |
| 6846 | ENSG00000102100 |  |
| 6847 | ENSG00000118194 |  |
| 6848 | ENSG00000139832 |  |
| 6849 | ENSG00000176659 |  |
| 6850 | ENSG00000158373 |  |
| 6851 | ENSG00000146857 |  |
| 6852 | ENSG00000113249 |  |
| 6853 | ENSG00000101608 |  |
| 6854 | ENSG00000108107 |  |
| 6855 | ENSG00000116044 |  |
| 6856 | ENSG00000269403 |  |
| 6857 | ENSG00000133056 |  |
| 6858 | ENSG00000140932 |  |
| 6859 | ENSG00000071127 |  |
| 6860 | ENSG00000011007 |  |
| 6861 | ENSG00000048471 |  |
| 6862 | ENSG00000168124 |  |
| 6863 | ENSG00000167996 |  |
| 6864 | ENSG00000124743 |  |
| 6865 | ENSG00000203684 |  |
| 6866 | ENSG00000126003 |  |
| 6867 | ENSG00000166881 |  |
| 6868 | ENSG00000198894 |  |
| 6869 | ENSG00000007129 |  |
| 6870 | ENSG00000063169 |  |
| 6871 | ENSG00000269182 |  |
| 6872 | ENSG00000187185 |  |
| 6873 | ENSG00000196704 |  |
| 6874 | ENSG00000133433 |  |
| 6875 | ENSG00000167257 |  |
| 6876 | ENSG00000005882 |  |
| 6877 | ENSG00000164171 |  |
| 6878 | ENSG00000003436 |  |
| 6879 | ENSG00000150995 |  |
| 6880 | ENSG00000159173 |  |
| 6881 | ENSG00000175110 |  |
| 6882 | ENSG00000041982 |  |
| 6883 | ENSG00000161682 |  |
| 6884 | ENSG00000215067 |  |
| 6885 | ENSG00000169372 |  |
| 6886 | ENSG00000064687 |  |
| 6887 | ENSG00000247596 |  |
| 6888 | ENSG00000255974 |  |
| 6889 | ENSG00000141449 |  |
| 6890 | ENSG00000222020 |  |
| 6891 | ENSG00000242689 |  |
| 6892 | ENSG00000121039 |  |
| 6893 | ENSG00000133985 |  |
| 6894 | ENSG00000130758 |  |
| 6895 | ENSG00000243646 |  |
| 6896 | ENSG00000146909 |  |
| 6897 | ENSG00000068697 |  |
| 6898 | ENSG00000142102 |  |
| 6899 | ENSG00000116815 |  |
| 6900 | ENSG00000082014 |  |
| 6901 | ENSG00000176340 |  |
| 6902 | ENSG00000100823 |  |
| 6903 | ENSG00000006625 |  |
| 6904 | ENSG00000089163 |  |
| 6905 | ENSG00000142634 |  |
| 6906 | ENSG00000145365 |  |
| 6907 | ENSG00000198873 |  |
| 6908 | ENSG00000119878 |  |
| 6909 | ENSG00000099624 |  |
| 6910 | ENSG00000153029 |  |
| 6911 | ENSG00000157881 |  |
| 6912 | ENSG00000121858 |  |
| 6913 | ENSG00000013523 |  |
| 6914 | ENSG00000239713 |  |
| 6915 | ENSG00000173575 |  |
| 6916 | ENSG00000149485 |  |
| 6917 | ENSG00000107897 |  |
| 6918 | ENSG00000070444 |  |
| 6919 | ENSG00000163872 |  |
| 6920 | ENSG00000103034 |  |
| 6921 | ENSG00000123405 |  |
| 6922 | ENSG00000172590 |  |
| 6923 | ENSG00000112773 |  |
| 6924 | ENSG00000213639 |  |
| 6925 | ENSG00000157214 |  |
| 6926 | ENSG00000107593 |  |
| 6927 | ENSG00000123416 |  |
| 6928 | ENSG00000243364 |  |
| 6929 | ENSG00000240747 |  |
| 6930 | ENSG00000140299 |  |
| 6931 | ENSG00000070388 |  |
| 6932 | ENSG00000183891 |  |
| 6933 | ENSG00000090372 |  |
| 6934 | ENSG00000076513 |  |
| 6935 | ENSG00000165548 |  |
| 6936 | ENSG00000100490 |  |
| 6937 | ENSG00000160991 |  |
| 6938 | ENSG00000123992 |  |
| 6939 | ENSG00000184281 |  |
| 6940 | ENSG00000107130 |  |
| 6941 | ENSG00000100201 |  |
| 6942 | ENSG00000088448 |  |
| 6943 | ENSG00000160685 |  |
| 6944 | ENSG00000066468 |  |
| 6945 | ENSG00000150977 |  |
| 6946 | ENSG00000053108 |  |
| 6947 | ENSG00000053918 |  |
| 6948 | ENSG00000203392 |  |
| 6949 | ENSG00000117602 |  |
| 6950 | ENSG00000168488 |  |
| 6951 | ENSG00000112739 |  |
| 6952 | ENSG00000156413 |  |
| 6953 | ENSG00000101413 |  |
| 6954 | ENSG00000189184 |  |
| 6955 | ENSG00000185811 |  |
| 6956 | ENSG00000140481 |  |
| 6957 | ENSG00000272916 |  |
| 6958 | ENSG00000166546 |  |
| 6959 | ENSG00000165752 |  |
| 6960 | ENSG00000124882 |  |
| 6961 | ENSG00000178726 |  |
| 6962 | ENSG00000110801 |  |
| 6963 | ENSG00000141012 |  |
| 6964 | ENSG00000167807 |  |
| 6965 | ENSG00000084652 |  |
| 6966 | ENSG00000173486 |  |
| 6967 | ENSG00000237190 |  |
| 6968 | ENSG00000163864 |  |
| 6969 | ENSG00000153157 |  |
| 6970 | ENSG00000137135 |  |
| 6971 | ENSG00000128973 |  |
| 6972 | ENSG00000174456 |  |
| 6973 | ENSG00000116990 |  |
| 6974 | ENSG00000153485 |  |
| 6975 | ENSG00000159618 |  |
| 6976 | ENSG00000166295 |  |
| 6977 | ENSG00000187066 |  |
| 6978 | ENSG00000160868 |  |
| 6979 | ENSG00000134321 |  |
| 6980 | ENSG00000213390 |  |
| 6981 | ENSG00000090432 |  |
| 6982 | ENSG00000133265 |  |
| 6983 | ENSG00000121680 |  |
| 6984 | ENSG00000196431 |  |
| 6985 | ENSG00000086848 |  |
| 6986 | ENSG00000008118 |  |
| 6987 | ENSG00000131097 |  |
| 6988 | ENSG00000198171 |  |
| 6989 | ENSG00000085982 |  |
| 6990 | ENSG00000206077 |  |
| 6991 | ENSG00000163636 |  |
| 6992 | ENSG00000112561 |  |
| 6993 | ENSG00000147573 |  |
| 6994 | ENSG00000105996 |  |
| 6995 | ENSG00000197697 |  |
| 6996 | ENSG00000103145 |  |
| 6997 | ENSG00000268111 |  |
| 6998 | ENSG00000189159 |  |
| 6999 | ENSG00000118690 |  |
| 7000 | ENSG00000187446 |  |
| 7001 | ENSG00000254415 |  |
| 7002 | ENSG00000136840 |  |
| 7003 | ENSG00000137642 |  |
| 7004 | ENSG00000177854 |  |
| 7005 | ENSG00000126243 |  |
| 7006 | ENSG00000143319 |  |
| 7007 | ENSG00000101191 |  |
| 7008 | ENSG00000187268 |  |
| 7009 | ENSG00000182329 |  |
| 7010 | ENSG00000106992 |  |
| 7011 | ENSG00000232382 |  |
| 7012 | ENSG00000133316 |  |
| 7013 | ENSG00000114978 |  |
| 7014 | ENSG00000198477 |  |
| 7015 | ENSG00000134755 |  |
| 7016 | ENSG00000078668 |  |
| 7017 | ENSG00000240505 |  |
| 7018 | ENSG00000090470 |  |
| 7019 | ENSG00000165682 |  |
| 7020 | ENSG00000145354 |  |
| 7021 | ENSG00000171119 |  |
| 7022 | ENSG00000262874 |  |
| 7023 | ENSG00000159239 |  |
| 7024 | ENSG00000111664 |  |
| 7025 | ENSG00000225614 |  |
| 7026 | ENSG00000166578 |  |
| 7027 | ENSG00000188493 |  |
| 7028 | ENSG00000168497 |  |
| 7029 | ENSG00000177340 |  |
| 7030 | ENSG00000159307 |  |
| 7031 | ENSG00000197992 |  |
| 7032 | ENSG00000092094 |  |
| 7033 | ENSG00000103449 |  |
| 7034 | ENSG00000110031 |  |
| 7035 | ENSG00000144230 |  |
| 7036 | ENSG00000103642 |  |
| 7037 | ENSG00000088836 |  |
| 7038 | ENSG00000184761 |  |
| 7039 | ENSG00000168280 |  |
| 7040 | ENSG00000175895 |  |
| 7041 | ENSG00000005513 |  |
| 7042 | ENSG00000140067 |  |
| 7043 | ENSG00000151914 |  |
| 7044 | ENSG00000148218 |  |
| 7045 | ENSG00000188368 |  |
| 7046 | ENSG00000131149 |  |
| 7047 | ENSG00000266997 |  |
| 7048 | ENSG00000157837 |  |
| 7049 | ENSG00000217442 |  |
| 7050 | ENSG00000116641 |  |
| 7051 | ENSG00000108515 |  |
| 7052 | ENSG00000177425 |  |
| 7053 | ENSG00000065911 |  |
| 7054 | ENSG00000138814 |  |
| 7055 | ENSG00000171169 |  |
| 7056 | ENSG00000197614 |  |
| 7057 | ENSG00000075240 |  |
| 7058 | ENSG00000116288 |  |
| 7059 | ENSG00000167780 |  |
| 7060 | ENSG00000198327 |  |
| 7061 | ENSG00000157538 |  |
| 7062 | ENSG00000115107 |  |
| 7063 | ENSG00000159409 |  |
| 7064 | ENSG00000119699 |  |
| 7065 | ENSG00000111432 |  |
| 7066 | ENSG00000128039 |  |
| 7067 | ENSG00000104518 |  |
| 7068 | ENSG00000012223 |  |
| 7069 | ENSG00000141030 |  |
| 7070 | ENSG00000090621 |  |
| 7071 | ENSG00000110851 |  |
| 7072 | ENSG00000116489 |  |
| 7073 | ENSG00000085063 |  |
| 7074 | ENSG00000146285 |  |
| 7075 | ENSG00000110651 |  |
| 7076 | ENSG00000196670 |  |
| 7077 | ENSG00000164591 |  |
| 7078 | ENSG00000060709 |  |
| 7079 | ENSG00000149798 |  |
| 7080 | ENSG00000205595 |  |
| 7081 | ENSG00000179218 |  |
| 7082 | ENSG00000127074 |  |
| 7083 | ENSG00000110048 |  |
| 7084 | ENSG00000130726 |  |
| 7085 | ENSG00000120075 |  |
| 7086 | ENSG00000198691 |  |
| 7087 | ENSG00000155463 |  |
| 7088 | ENSG00000089505 |  |
| 7089 | ENSG00000183098 |  |
| 7090 | ENSG00000142409 |  |
| 7091 | ENSG00000020922 |  |
| 7092 | ENSG00000187951 |  |
| 7093 | ENSG00000090920 |  |
| 7094 | ENSG00000101189 |  |
| 7095 | ENSG00000183153 |  |
| 7096 | ENSG00000155719 |  |
| 7097 | ENSG00000099308 |  |
| 7098 | ENSG00000129667 |  |
| 7099 | ENSG00000147548 |  |
| 7100 | ENSG00000159556 |  |
| 7101 | ENSG00000169435 |  |
| 7102 | ENSG00000164674 |  |
| 7103 | ENSG00000196605 |  |
| 7104 | ENSG00000140044 |  |
| 7105 | ENSG00000059122 |  |
| 7106 | ENSG00000142632 |  |
| 7107 | ENSG00000123358 |  |
| 7108 | ENSG00000173825 |  |
| 7109 | ENSG00000100302 |  |
| 7110 | ENSG00000172932 |  |
| 7111 | ENSG00000213937 |  |
| 7112 | ENSG00000129991 |  |
| 7113 | ENSG00000137094 |  |
| 7114 | ENSG00000146834 |  |
| 7115 | ENSG00000100281 |  |
| 7116 | ENSG00000196981 |  |
| 7117 | ENSG00000268797 |  |
| 7118 | ENSG00000170634 |  |
| 7119 | ENSG00000172831 |  |
| 7120 | ENSG00000172824 |  |
| 7121 | ENSG00000184697 |  |
| 7122 | ENSG00000144455 |  |
| 7123 | ENSG00000114993 |  |
| 7124 | ENSG00000072778 |  |
| 7125 | ENSG00000010292 |  |
| 7126 | ENSG00000158793 |  |
| 7127 | ENSG00000101152 |  |
| 7128 | ENSG00000179958 |  |
| 7129 | ENSG00000108021 |  |
| 7130 | ENSG00000173110 |  |
| 7131 | ENSG00000105732 |  |
| 7132 | ENSG00000130396 |  |
| 7133 | ENSG00000154889 |  |
| 7134 | ENSG00000174015 |  |
| 7135 | ENSG00000122691 |  |
| 7136 | ENSG00000168785 |  |
| 7137 | ENSG00000111817 |  |
| 7138 | ENSG00000133943 |  |
| 7139 | ENSG00000257950 |  |
| 7140 | ENSG00000163694 |  |
| 7141 | ENSG00000189319 |  |
| 7142 | ENSG00000135119 |  |
| 7143 | ENSG00000131747 |  |
| 7144 | ENSG00000269510 |  |
| 7145 | ENSG00000188820 |  |
| 7146 | ENSG00000269891 |  |
| 7147 | ENSG00000154839 |  |
| 7148 | ENSG00000168575 |  |
| 7149 | ENSG00000176984 |  |
| 7150 | ENSG00000125459 |  |
| 7151 | ENSG00000128789 |  |
| 7152 | ENSG00000176165 |  |
| 7153 | ENSG00000134077 |  |
| 7154 | ENSG00000188761 |  |
| 7155 | ENSG00000179044 |  |
| 7156 | ENSG00000169340 |  |
| 7157 | ENSG00000090316 |  |
| 7158 | ENSG00000163827 |  |
| 7159 | ENSG00000177943 |  |
| 7160 | ENSG00000162889 |  |
| 7161 | ENSG00000189409 |  |
| 7162 | ENSG00000161992 |  |
| 7163 | ENSG00000267706 |  |
| 7164 | ENSG00000179294 |  |
| 7165 | ENSG00000165030 |  |
| 7166 | ENSG00000174527 |  |
| 7167 | ENSG00000165716 |  |
| 7168 | ENSG00000108298 |  |
| 7169 | ENSG00000172840 |  |
| 7170 | ENSG00000072071 |  |
| 7171 | ENSG00000205784 |  |
| 7172 | ENSG00000148331 |  |
| 7173 | ENSG00000116679 |  |
| 7174 | ENSG00000165804 |  |
| 7175 | ENSG00000185630 |  |
| 7176 | ENSG00000177051 |  |
| 7177 | ENSG00000105221 |  |
| 7178 | ENSG00000197905 |  |
| 7179 | ENSG00000205808 |  |
| 7180 | ENSG00000143382 |  |
| 7181 | ENSG00000103528 |  |
| 7182 | ENSG00000101745 |  |
| 7183 | ENSG00000134375 |  |
| 7184 | ENSG00000117289 |  |
| 7185 | ENSG00000115457 |  |
| 7186 | ENSG00000049249 |  |
| 7187 | ENSG00000169925 |  |
| 7188 | ENSG00000166073 |  |
| 7189 | ENSG00000004777 |  |
| 7190 | ENSG00000104660 |  |
| 7191 | ENSG00000246366 |  |
| 7192 | ENSG00000100307 |  |
| 7193 | ENSG00000163884 |  |
| 7194 | ENSG00000179262 |  |
| 7195 | ENSG00000102786 |  |
| 7196 | ENSG00000170653 |  |
| 7197 | ENSG00000090097 |  |
| 7198 | ENSG00000146648 |  |
| 7199 | ENSG00000115828 |  |
| 7200 | ENSG00000255482 |  |
| 7201 | ENSG00000054965 |  |
| 7202 | ENSG00000161999 |  |
| 7203 | ENSG00000255150 |  |
| 7204 | ENSG00000176393 |  |
| 7205 | ENSG00000067596 |  |
| 7206 | ENSG00000137404 |  |
| 7207 | ENSG00000164404 |  |
| 7208 | ENSG00000113851 |  |
| 7209 | ENSG00000103351 |  |
| 7210 | ENSG00000164855 |  |
| 7211 | ENSG00000118276 |  |
| 7212 | ENSG00000145416 |  |
| 7213 | ENSG00000177873 |  |
| 7214 | ENSG00000163346 |  |
| 7215 | ENSG00000132386 |  |
| 7216 | ENSG00000006534 |  |
| 7217 | ENSG00000163479 |  |
| 7218 | ENSG00000125820 |  |
| 7219 | ENSG00000102802 |  |
| 7220 | ENSG00000142748 |  |
| 7221 | ENSG00000135077 |  |
| 7222 | ENSG00000268310 |  |
| 7223 | ENSG00000125868 |  |
| 7224 | ENSG00000183808 |  |
| 7225 | ENSG00000131400 |  |
| 7226 | ENSG00000031823 |  |
| 7227 | ENSG00000143748 |  |
| 7228 | ENSG00000181991 |  |
| 7229 | ENSG00000203814 |  |
| 7230 | ENSG00000141385 |  |
| 7231 | ENSG00000169327 |  |
| 7232 | ENSG00000169715 |  |
| 7233 | ENSG00000172889 |  |
| 7234 | ENSG00000177272 |  |
| 7235 | ENSG00000180104 |  |
| 7236 | ENSG00000239857 |  |
| 7237 | ENSG00000171532 |  |
| 7238 | ENSG00000124762 |  |
| 7239 | ENSG00000137878 |  |
| 7240 | ENSG00000117407 |  |
| 7241 | ENSG00000101109 |  |
| 7242 | ENSG00000144591 |  |
| 7243 | ENSG00000055118 |  |
| 7244 | ENSG00000134248 |  |
| 7245 | ENSG00000188176 |  |
| 7246 | ENSG00000173465 |  |
| 7247 | ENSG00000171574 |  |
| 7248 | ENSG00000136444 |  |
| 7249 | ENSG00000100239 |  |
| 7250 | ENSG00000156502 |  |
| 7251 | ENSG00000085644 |  |
| 7252 | ENSG00000149926 |  |
| 7253 | ENSG00000109320 |  |
| 7254 | ENSG00000167775 |  |
| 7255 | ENSG00000153933 |  |
| 7256 | ENSG00000204310 |  |
| 7257 | ENSG00000196391 |  |
| 7258 | ENSG00000135503 |  |
| 7259 | ENSG00000103047 |  |
| 7260 | ENSG00000131620 |  |
| 7261 | ENSG00000261732 |  |
| 7262 | ENSG00000162521 |  |
| 7263 | ENSG00000164284 |  |
| 7264 | ENSG00000065621 |  |
| 7265 | ENSG00000198018 |  |
| 7266 | ENSG00000147416 |  |
| 7267 | ENSG00000140526 |  |
| 7268 | ENSG00000119574 |  |
| 7269 | ENSG00000112115 |  |
| 7270 | ENSG00000129351 |  |
| 7271 | ENSG00000198553 |  |
| 7272 | ENSG00000078177 |  |
| 7273 | ENSG00000125968 |  |
| 7274 | ENSG00000124006 |  |
| 7275 | ENSG00000099260 |  |
| 7276 | ENSG00000188554 |  |
| 7277 | ENSG00000260869 |  |
| 7278 | ENSG00000198959 |  |
| 7279 | ENSG00000155275 |  |
| 7280 | ENSG00000183853 |  |
| 7281 | ENSG00000178093 |  |
| 7282 | ENSG00000146094 |  |
| 7283 | ENSG00000089356 |  |
| 7284 | ENSG00000159640 |  |
| 7285 | ENSG00000103174 |  |
| 7286 | ENSG00000165061 |  |
| 7287 | ENSG00000221823 |  |
| 7288 | ENSG00000188282 |  |
| 7289 | ENSG00000136854 |  |
| 7290 | ENSG00000066379 |  |
| 7291 | ENSG00000182909 |  |
| 7292 | ENSG00000033178 |  |
| 7293 | ENSG00000233280 |  |
| 7294 | ENSG00000185215 |  |
| 7295 | ENSG00000080854 |  |
| 7296 | ENSG00000158006 |  |
| 7297 | ENSG00000066855 |  |
| 7298 | ENSG00000167889 |  |
| 7299 | ENSG00000163249 |  |
| 7300 | ENSG00000177954 |  |
| 7301 | ENSG00000183291 |  |
| 7302 | ENSG00000132423 |  |
| 7303 | ENSG00000113430 |  |
| 7304 | ENSG00000114315 |  |
| 7305 | ENSG00000170881 |  |
| 7306 | ENSG00000013563 |  |
| 7307 | ENSG00000101306 |  |
| 7308 | ENSG00000125304 |  |
| 7309 | ENSG00000029363 |  |
| 7310 | ENSG00000256655 |  |
| 7311 | ENSG00000268423 |  |
| 7312 | ENSG00000151067 |  |
| 7313 | ENSG00000100632 |  |
| 7314 | ENSG00000183801 |  |
| 7315 | ENSG00000112877 |  |
| 7316 | ENSG00000153044 |  |
| 7317 | ENSG00000172289 |  |
| 7318 | ENSG00000118308 |  |
| 7319 | ENSG00000184986 |  |
| 7320 | ENSG00000147604 |  |
| 7321 | ENSG00000126934 |  |
| 7322 | ENSG00000130300 |  |
| 7323 | ENSG00000204414 |  |
| 7324 | ENSG00000144040 |  |
| 7325 | ENSG00000115008 |  |
| 7326 | ENSG00000181773 |  |
| 7327 | ENSG00000103245 |  |
| 7328 | ENSG00000189334 |  |
| 7329 | ENSG00000123427 |  |
| 7330 | ENSG00000124251 |  |
| 7331 | ENSG00000133460 |  |
| 7332 | ENSG00000039987 |  |
| 7333 | ENSG00000104142 |  |
| 7334 | ENSG00000214253 |  |
| 7335 | ENSG00000131795 |  |
| 7336 | ENSG00000180549 |  |
| 7337 | ENSG00000198771 |  |
| 7338 | ENSG00000070087 |  |
| 7339 | ENSG00000110011 |  |
| 7340 | ENSG00000034053 |  |
| 7341 | ENSG00000173389 |  |
| 7342 | ENSG00000143258 |  |
| 7343 | ENSG00000124249 |  |
| 7344 | ENSG00000108826 |  |
| 7345 | ENSG00000197077 |  |
| 7346 | ENSG00000159111 |  |
| 7347 | ENSG00000107819 |  |
| 7348 | ENSG00000111554 |  |
| 7349 | ENSG00000265096 |  |
| 7350 | ENSG00000206344 |  |
| 7351 | ENSG00000102007 |  |
| 7352 | ENSG00000167074 |  |
| 7353 | ENSG00000143839 |  |
| 7354 | ENSG00000008952 |  |
| 7355 | ENSG00000137166 |  |
| 7356 | ENSG00000140279 |  |
| 7357 | ENSG00000187010 |  |
| 7358 | ENSG00000197837 |  |
| 7359 | ENSG00000147687 |  |
| 7360 | ENSG00000168874 |  |
| 7361 | ENSG00000171860 |  |
| 7362 | ENSG00000188015 |  |
| 7363 | ENSG00000167105 |  |
| 7364 | ENSG00000121900 |  |
| 7365 | ENSG00000243414 |  |
| 7366 | ENSG00000142512 |  |
| 7367 | ENSG00000109794 |  |
| 7368 | ENSG00000197540 |  |
| 7369 | ENSG00000004866 |  |
| 7370 | ENSG00000167535 |  |
| 7371 | ENSG00000116985 |  |
| 7372 | ENSG00000141577 |  |
| 7373 | ENSG00000150867 |  |
| 7374 | ENSG00000185482 |  |
| 7375 | ENSG00000176723 |  |
| 7376 | ENSG00000158874 |  |
| 7377 | ENSG00000157111 |  |
| 7378 | ENSG00000186815 |  |
| 7379 | ENSG00000168539 |  |
| 7380 | ENSG00000103363 |  |
| 7381 | ENSG00000254206 |  |
| 7382 | ENSG00000095066 |  |
| 7383 | ENSG00000178952 |  |
| 7384 | ENSG00000137720 |  |
| 7385 | ENSG00000179526 |  |
| 7386 | ENSG00000134851 |  |
| 7387 | ENSG00000102804 |  |
| 7388 | ENSG00000173077 |  |
| 7389 | ENSG00000249209 |  |
| 7390 | ENSG00000140743 |  |
| 7391 | ENSG00000113296 |  |
| 7392 | ENSG00000198055 |  |
| 7393 | ENSG00000120910 |  |
| 7394 | ENSG00000161328 |  |
| 7395 | ENSG00000167118 |  |
| 7396 | ENSG00000105427 |  |
| 7397 | ENSG00000126062 |  |
| 7398 | ENSG00000160767 |  |
| 7399 | ENSG00000168140 |  |
| 7400 | ENSG00000234469 |  |
| 7401 | ENSG00000165685 |  |
| 7402 | ENSG00000173137 |  |
| 7403 | ENSG00000144021 |  |
| 7404 | ENSG00000171425 |  |
| 7405 | ENSG00000185624 |  |
| 7406 | ENSG00000163808 |  |
| 7407 | ENSG00000104522 |  |
| 7408 | ENSG00000091409 |  |
| 7409 | ENSG00000130653 |  |
| 7410 | ENSG00000092051 |  |
| 7411 | ENSG00000182253 |  |
| 7412 | ENSG00000153113 |  |
| 7413 | ENSG00000174255 |  |
| 7414 | ENSG00000163348 |  |
| 7415 | ENSG00000138030 |  |
| 7416 | ENSG00000186951 |  |
| 7417 | ENSG00000204010 |  |
| 7418 | ENSG00000167815 |  |
| 7419 | ENSG00000111424 |  |
| 7420 | ENSG00000114735 |  |
| 7421 | ENSG00000260027 |  |
| 7422 | ENSG00000124641 |  |
| 7423 | ENSG00000140107 |  |
| 7424 | ENSG00000142945 |  |
| 7425 | ENSG00000134324 |  |
| 7426 | ENSG00000183018 |  |
| 7427 | ENSG00000109046 |  |
| 7428 | ENSG00000150656 |  |
| 7429 | ENSG00000138363 |  |
| 7430 | ENSG00000100034 |  |
| 7431 | ENSG00000059145 |  |
| 7432 | ENSG00000139718 |  |
| 7433 | ENSG00000120645 |  |
| 7434 | ENSG00000104998 |  |
| 7435 | ENSG00000074410 |  |
| 7436 | ENSG00000070985 |  |
| 7437 | ENSG00000182963 |  |
| 7438 | ENSG00000164091 |  |
| 7439 | ENSG00000168394 |  |
| 7440 | ENSG00000183207 |  |
| 7441 | ENSG00000170549 |  |
| 7442 | ENSG00000168060 |  |
| 7443 | ENSG00000115758 |  |
| 7444 | ENSG00000198276 |  |
| 7445 | ENSG00000117632 |  |
| 7446 | ENSG00000155903 |  |
| 7447 | ENSG00000138641 |  |
| 7448 | ENSG00000105479 |  |
| 7449 | ENSG00000131746 |  |
| 7450 | ENSG00000107614 |  |
| 7451 | ENSG00000103550 |  |
| 7452 | ENSG00000135250 |  |
| 7453 | ENSG00000187650 |  |
| 7454 | ENSG00000184451 |  |
| 7455 | ENSG00000100368 |  |
| 7456 | ENSG00000110245 |  |
| 7457 | ENSG00000125970 |  |
| 7458 | ENSG00000152093 |  |
| 7459 | ENSG00000131375 |  |
| 7460 | ENSG00000049618 |  |
| 7461 | ENSG00000162510 |  |
| 7462 | ENSG00000261915 |  |
| 7463 | ENSG00000169018 |  |
| 7464 | ENSG00000225996 |  |
| 7465 | ENSG00000141294 |  |
| 7466 | ENSG00000220032 |  |
| 7467 | ENSG00000196456 |  |
| 7468 | ENSG00000130005 |  |
| 7469 | ENSG00000117543 |  |
| 7470 | ENSG00000178445 |  |
| 7471 | ENSG00000170190 |  |
| 7472 | ENSG00000101596 |  |
| 7473 | ENSG00000145675 |  |
| 7474 | ENSG00000129244 |  |
| 7475 | ENSG00000121297 |  |
| 7476 | ENSG00000070761 |  |
| 7477 | ENSG00000140853 |  |
| 7478 | ENSG00000105675 |  |
| 7479 | ENSG00000113504 |  |
| 7480 | ENSG00000107779 |  |
| 7481 | ENSG00000101210 |  |
| 7482 | ENSG00000259207 |  |
| 7483 | ENSG00000249679 |  |
| 7484 | ENSG00000185924 |  |
| 7485 | ENSG00000204305 |  |
| 7486 | ENSG00000089101 |  |
| 7487 | ENSG00000196843 |  |
| 7488 | ENSG00000135406 |  |
| 7489 | ENSG00000142789 |  |
| 7490 | ENSG00000102245 |  |
| 7491 | ENSG00000135018 |  |
| 7492 | ENSG00000182957 |  |
| 7493 | ENSG00000104888 |  |
| 7494 | ENSG00000259288 |  |
| 7495 | ENSG00000166924 |  |
| 7496 | ENSG00000160213 |  |
| 7497 | ENSG00000142252 |  |
| 7498 | ENSG00000126337 |  |
| 7499 | ENSG00000130598 |  |
| 7500 | ENSG00000160094 |  |
| 7501 | ENSG00000186790 |  |
| 7502 | ENSG00000164902 |  |
| 7503 | ENSG00000218537 |  |
| 7504 | ENSG00000172663 |  |
| 7505 | ENSG00000084623 |  |
| 7506 | ENSG00000168792 |  |
| 7507 | ENSG00000177354 |  |
| 7508 | ENSG00000163462 |  |
| 7509 | ENSG00000164938 |  |
| 7510 | ENSG00000181856 |  |
| 7511 | ENSG00000198933 |  |
| 7512 | ENSG00000103064 |  |
| 7513 | ENSG00000066739 |  |
| 7514 | ENSG00000138041 |  |
| 7515 | ENSG00000180861 |  |
| 7516 | ENSG00000157193 |  |
| 7517 | ENSG00000198914 |  |
| 7518 | ENSG00000167283 |  |
| 7519 | ENSG00000073578 |  |
| 7520 | ENSG00000139044 |  |
| 7521 | ENSG00000172469 |  |
| 7522 | ENSG00000081479 |  |
| 7523 | ENSG00000165879 |  |
| 7524 | ENSG00000261796 |  |
| 7525 | ENSG00000204345 |  |
| 7526 | ENSG00000227450 |  |
| 7527 | ENSG00000213132 |  |
| 7528 | ENSG00000132017 |  |
| 7529 | ENSG00000273266 |  |
| 7530 | ENSG00000087495 |  |
| 7531 | ENSG00000196758 |  |
| 7532 | ENSG00000177666 |  |
| 7533 | ENSG00000163659 |  |
| 7534 | ENSG00000132031 |  |
| 7535 | ENSG00000181610 |  |
| 7536 | ENSG00000231861 |  |
| 7537 | ENSG00000269690 |  |
| 7538 | ENSG00000187922 |  |
| 7539 | ENSG00000119703 |  |
| 7540 | ENSG00000160439 |  |
| 7541 | ENSG00000181789 |  |
| 7542 | ENSG00000108424 |  |
| 7543 | ENSG00000142197 |  |
| 7544 | ENSG00000180818 |  |
| 7545 | ENSG00000108953 |  |
| 7546 | ENSG00000260007 |  |
| 7547 | ENSG00000183784 |  |
| 7548 | ENSG00000180228 |  |
| 7549 | ENSG00000139826 |  |
| 7550 | ENSG00000184224 |  |
| 7551 | ENSG00000183718 |  |
| 7552 | ENSG00000267673 |  |
| 7553 | ENSG00000171720 |  |
| 7554 | ENSG00000255398 |  |
| 7555 | ENSG00000185888 |  |
| 7556 | ENSG00000198081 |  |
| 7557 | ENSG00000044574 |  |
| 7558 | ENSG00000114251 |  |
| 7559 | ENSG00000100647 |  |
| 7560 | ENSG00000159692 |  |
| 7561 | ENSG00000080603 |  |
| 7562 | ENSG00000188987 |  |
| 7563 | ENSG00000107736 |  |
| 7564 | ENSG00000139725 |  |
| 7565 | ENSG00000221932 |  |
| 7566 | ENSG00000143878 |  |
| 7567 | ENSG00000164303 |  |
| 7568 | ENSG00000048462 |  |
| 7569 | ENSG00000148677 |  |
| 7570 | ENSG00000131409 |  |
| 7571 | ENSG00000160190 |  |
| 7572 | ENSG00000181467 |  |
| 7573 | ENSG00000159840 |  |
| 7574 | ENSG00000151276 |  |
| 7575 | ENSG00000180745 |  |
| 7576 | ENSG00000134255 |  |
| 7577 | ENSG00000099377 |  |
| 7578 | ENSG00000160072 |  |
| 7579 | ENSG00000116017 |  |
| 7580 | ENSG00000176658 |  |
| 7581 | ENSG00000101463 |  |
| 7582 | ENSG00000100813 |  |
| 7583 | ENSG00000187806 |  |
| 7584 | ENSG00000130222 |  |
| 7585 | ENSG00000063127 |  |
| 7586 | ENSG00000173442 |  |
| 7587 | ENSG00000122035 |  |
| 7588 | ENSG00000262484 |  |
| 7589 | ENSG00000100664 |  |
| 7590 | ENSG00000189152 |  |
| 7591 | ENSG00000171551 |  |
| 7592 | ENSG00000164236 |  |
| 7593 | ENSG00000166801 |  |
| 7594 | ENSG00000165379 |  |
| 7595 | ENSG00000225828 |  |
| 7596 | ENSG00000124920 |  |
| 7597 | ENSG00000132000 |  |
| 7598 | ENSG00000119899 |  |
| 7599 | ENSG00000163399 |  |
| 7600 | ENSG00000228300 |  |
| 7601 | ENSG00000099917 |  |
| 7602 | ENSG00000122026 |  |
| 7603 | ENSG00000212899 |  |
| 7604 | ENSG00000222022 |  |
| 7605 | ENSG00000170074 |  |
| 7606 | ENSG00000116251 |  |
| 7607 | ENSG00000159363 |  |
| 7608 | ENSG00000215915 |  |
| 7609 | ENSG00000168661 |  |
| 7610 | ENSG00000115257 |  |
| 7611 | ENSG00000178927 |  |
| 7612 | ENSG00000011009 |  |
| 7613 | ENSG00000125122 |  |
| 7614 | ENSG00000273154 |  |
| 7615 | ENSG00000163050 |  |
| 7616 | ENSG00000096696 |  |
| 7617 | ENSG00000164088 |  |
| 7618 | ENSG00000162702 |  |
| 7619 | ENSG00000101346 |  |
| 7620 | ENSG00000269955 |  |
| 7621 | ENSG00000185372 |  |
| 7622 | ENSG00000132141 |  |
| 7623 | ENSG00000169607 |  |
| 7624 | ENSG00000100078 |  |
| 7625 | ENSG00000026652 |  |
| 7626 | ENSG00000159263 |  |
| 7627 | ENSG00000100207 |  |
| 7628 | ENSG00000186862 |  |
| 7629 | ENSG00000090273 |  |
| 7630 | ENSG00000060656 |  |
| 7631 | ENSG00000169398 |  |
| 7632 | ENSG00000160007 |  |
| 7633 | ENSG00000256591 |  |
| 7634 | ENSG00000088002 |  |
| 7635 | ENSG00000253633 |  |
| 7636 | ENSG00000185658 |  |
| 7637 | ENSG00000121594 |  |
| 7638 | ENSG00000184608 |  |
| 7639 | ENSG00000204104 |  |
| 7640 | ENSG00000188483 |  |
| 7641 | ENSG00000187079 |  |
| 7642 | ENSG00000019144 |  |
| 7643 | ENSG00000156030 |  |
| 7644 | ENSG00000002919 |  |
| 7645 | ENSG00000141568 |  |
| 7646 | ENSG00000184489 |  |
| 7647 | ENSG00000117155 |  |
| 7648 | ENSG00000171611 |  |
| 7649 | ENSG00000164654 |  |
| 7650 | ENSG00000188643 |  |
| 7651 | ENSG00000112494 |  |
| 7652 | ENSG00000139687 |  |
| 7653 | ENSG00000180900 |  |
| 7654 | ENSG00000145734 |  |
| 7655 | ENSG00000197409 |  |
| 7656 | ENSG00000147403 |  |
| 7657 | ENSG00000086475 |  |
| 7658 | ENSG00000115525 |  |
| 7659 | ENSG00000171115 |  |
| 7660 | ENSG00000167614 |  |
| 7661 | ENSG00000189079 |  |
| 7662 | ENSG00000196678 |  |
| 7663 | ENSG00000144848 |  |
| 7664 | ENSG00000164176 |  |
| 7665 | ENSG00000161638 |  |
| 7666 | ENSG00000108370 |  |
| 7667 | ENSG00000144843 |  |
| 7668 | ENSG00000176919 |  |
| 7669 | ENSG00000172653 |  |
| 7670 | ENSG00000189266 |  |
| 7671 | ENSG00000100053 |  |
| 7672 | ENSG00000150938 |  |
| 7673 | ENSG00000118263 |  |
| 7674 | ENSG00000106624 |  |
| 7675 | ENSG00000140836 |  |
| 7676 | ENSG00000175970 |  |
| 7677 | ENSG00000109332 |  |
| 7678 | ENSG00000114933 |  |
| 7679 | ENSG00000115138 |  |
| 7680 | ENSG00000150667 |  |
| 7681 | ENSG00000088280 |  |
| 7682 | ENSG00000168297 |  |
| 7683 | ENSG00000111731 |  |
| 7684 | ENSG00000143222 |  |
| 7685 | ENSG00000185198 |  |
| 7686 | ENSG00000177302 |  |
| 7687 | ENSG00000160188 |  |
| 7688 | ENSG00000162891 |  |
| 7689 | ENSG00000171223 |  |
| 7690 | ENSG00000158286 |  |
| 7691 | ENSG00000140612 |  |
| 7692 | ENSG00000239697 |  |
| 7693 | ENSG00000065135 |  |
| 7694 | ENSG00000129965 |  |
| 7695 | ENSG00000105609 |  |
| 7696 | ENSG00000079313 |  |
| 7697 | ENSG00000113732 |  |
| 7698 | ENSG00000131389 |  |
| 7699 | ENSG00000163875 |  |
| 7700 | ENSG00000221995 |  |
| 7701 | ENSG00000124120 |  |
| 7702 | ENSG00000141504 |  |
| 7703 | ENSG00000196371 |  |
| 7704 | ENSG00000171863 |  |
| 7705 | ENSG00000116237 |  |
| 7706 | ENSG00000167608 |  |
| 7707 | ENSG00000246705 |  |
| 7708 | ENSG00000170542 |  |
| 7709 | ENSG00000101695 |  |
| 7710 | ENSG00000149809 |  |
| 7711 | ENSG00000130783 |  |
| 7712 | ENSG00000142327 |  |
| 7713 | ENSG00000007392 |  |
| 7714 | ENSG00000163964 |  |
| 7715 | ENSG00000141959 |  |
| 7716 | ENSG00000173276 |  |
| 7717 | ENSG00000167642 |  |
| 7718 | ENSG00000104679 |  |
| 7719 | ENSG00000074755 |  |
| 7720 | ENSG00000088325 |  |
| 7721 | ENSG00000178928 |  |
| 7722 | ENSG00000141564 |  |
| 7723 | ENSG00000165507 |  |
| 7724 | ENSG00000173267 |  |
| 7725 | ENSG00000115718 |  |
| 7726 | ENSG00000119541 |  |
| 7727 | ENSG00000160360 |  |
| 7728 | ENSG00000125454 |  |
| 7729 | ENSG00000205795 |  |
| 7730 | ENSG00000120160 |  |
| 7731 | ENSG00000108262 |  |
| 7732 | ENSG00000162989 |  |
| 7733 | ENSG00000177191 |  |
| 7734 | ENSG00000159720 |  |
| 7735 | ENSG00000134812 |  |
| 7736 | ENSG00000269554 |  |
| 7737 | ENSG00000133055 |  |
| 7738 | ENSG00000104524 |  |
| 7739 | ENSG00000116353 |  |
| 7740 | ENSG00000168496 |  |
| 7741 | ENSG00000174197 |  |
| 7742 | ENSG00000159210 |  |
| 7743 | ENSG00000169371 |  |
| 7744 | ENSG00000163357 |  |
| 7745 | ENSG00000180953 |  |
| 7746 | ENSG00000138785 |  |
| 7747 | ENSG00000143842 |  |
| 7748 | ENSG00000118193 |  |
| 7749 | ENSG00000157895 |  |
| 7750 | ENSG00000107249 |  |
| 7751 | ENSG00000175294 |  |
| 7752 | ENSG00000235531 |  |
| 7753 | ENSG00000139351 |  |
| 7754 | ENSG00000183763 |  |
| 7755 | ENSG00000134545 |  |
| 7756 | ENSG00000164889 |  |
| 7757 | ENSG00000170498 |  |
| 7758 | ENSG00000173988 |  |
| 7759 | ENSG00000244687 |  |
| 7760 | ENSG00000175324 |  |
| 7761 | ENSG00000007402 |  |
| 7762 | ENSG00000153902 |  |
| 7763 | ENSG00000172613 |  |
| 7764 | ENSG00000173171 |  |
| 7765 | ENSG00000269883 |  |
| 7766 | ENSG00000143256 |  |
| 7767 | ENSG00000171488 |  |
| 7768 | ENSG00000073464 |  |
| 7769 | ENSG00000196562 |  |
| 7770 | ENSG00000214860 |  |
| 7771 | ENSG00000119048 |  |
| 7772 | ENSG00000167995 |  |
| 7773 | ENSG00000205560 |  |
| 7774 | ENSG00000203778 |  |
| 7775 | ENSG00000165916 |  |
| 7776 | ENSG00000188582 |  |
| 7777 | ENSG00000143515 |  |
| 7778 | ENSG00000070404 |  |
| 7779 | ENSG00000115268 |  |
| 7780 | ENSG00000185561 |  |
| 7781 | ENSG00000181915 |  |
| 7782 | ENSG00000268852 |  |
| 7783 | ENSG00000099974 |  |
| 7784 | ENSG00000113966 |  |
| 7785 | ENSG00000109062 |  |
| 7786 | ENSG00000108312 |  |
| 7787 | ENSG00000006125 |  |
| 7788 | ENSG00000165238 |  |
| 7789 | ENSG00000105523 |  |
| 7790 | ENSG00000118855 |  |
| 7791 | ENSG00000125445 |  |
| 7792 | ENSG00000139146 |  |
| 7793 | ENSG00000241852 |  |
| 7794 | ENSG00000164161 |  |
| 7795 | ENSG00000203301 |  |
| 7796 | ENSG00000137101 |  |
| 7797 | ENSG00000160201 |  |
| 7798 | ENSG00000137942 |  |
| 7799 | ENSG00000133121 |  |
| 7800 | ENSG00000107099 |  |
| 7801 | ENSG00000239732 |  |
| 7802 | ENSG00000268643 |  |
| 7803 | ENSG00000015592 |  |
| 7804 | ENSG00000215041 |  |
| 7805 | ENSG00000075711 |  |
| 7806 | ENSG00000204952 |  |
| 7807 | ENSG00000173801 |  |
| 7808 | ENSG00000159055 |  |
| 7809 | ENSG00000144224 |  |
| 7810 | ENSG00000166046 |  |
| 7811 | ENSG00000168003 |  |
| 7812 | ENSG00000119227 |  |
| 7813 | ENSG00000128714 |  |
| 7814 | ENSG00000107187 |  |
| 7815 | ENSG00000134825 |  |
| 7816 | ENSG00000142494 |  |
| 7817 | ENSG00000119771 |  |
| 7818 | ENSG00000066827 |  |
| 7819 | ENSG00000067064 |  |
| 7820 | ENSG00000157873 |  |
| 7821 | ENSG00000204049 |  |
| 7822 | ENSG00000130656 |  |
| 7823 | ENSG00000025772 |  |
| 7824 | ENSG00000128567 |  |
| 7825 | ENSG00000127561 |  |
| 7826 | ENSG00000185155 |  |
| 7827 | ENSG00000168056 |  |
| 7828 | ENSG00000177558 |  |
| 7829 | ENSG00000154025 |  |
| 7830 | ENSG00000103187 |  |
| 7831 | ENSG00000163219 |  |
| 7832 | ENSG00000014919 |  |
| 7833 | ENSG00000205629 |  |
| 7834 | ENSG00000117399 |  |
| 7835 | ENSG00000257341 |  |
| 7836 | ENSG00000172794 |  |
| 7837 | ENSG00000160712 |  |
| 7838 | ENSG00000117305 |  |
| 7839 | ENSG00000069998 |  |
| 7840 | ENSG00000241106 |  |
| 7841 | ENSG00000169231 |  |
| 7842 | ENSG00000267140 |  |
| 7843 | ENSG00000140465 |  |
| 7844 | ENSG00000152818 |  |
| 7845 | ENSG00000137210 |  |
| 7846 | ENSG00000164379 |  |
| 7847 | ENSG00000134202 |  |
| 7848 | ENSG00000015475 |  |
| 7849 | ENSG00000168334 |  |
| 7850 | ENSG00000117411 |  |
| 7851 | ENSG00000119638 |  |
| 7852 | ENSG00000128908 |  |
| 7853 | ENSG00000135723 |  |
| 7854 | ENSG00000128610 |  |
| 7855 | ENSG00000027847 |  |
| 7856 | ENSG00000136504 |  |
| 7857 | ENSG00000147421 |  |
| 7858 | ENSG00000160469 |  |
| 7859 | ENSG00000129038 |  |
| 7860 | ENSG00000204524 |  |
| 7861 | ENSG00000256029 |  |
| 7862 | ENSG00000089248 |  |
| 7863 | ENSG00000101197 |  |
| 7864 | ENSG00000254870 |  |
| 7865 | ENSG00000186010 |  |
| 7866 | ENSG00000170523 |  |
| 7867 | ENSG00000123144 |  |
| 7868 | ENSG00000153914 |  |
| 7869 | ENSG00000169504 |  |
| 7870 | ENSG00000077454 |  |
| 7871 | ENSG00000117139 |  |
| 7872 | ENSG00000162069 |  |
| 7873 | ENSG00000132478 |  |
| 7874 | ENSG00000204304 |  |
| 7875 | ENSG00000064313 |  |
| 7876 | ENSG00000089723 |  |
| 7877 | ENSG00000166949 |  |
| 7878 | ENSG00000109118 |  |
| 7879 | ENSG00000147586 |  |
| 7880 | ENSG00000197070 |  |
| 7881 | ENSG00000260272 |  |
| 7882 | ENSG00000127526 |  |
| 7883 | ENSG00000116882 |  |
| 7884 | ENSG00000161956 |  |
| 7885 | ENSG00000186960 |  |
| 7886 | ENSG00000103253 |  |
| 7887 | ENSG00000100242 |  |
| 7888 | ENSG00000182938 |  |
| 7889 | ENSG00000116544 |  |
| 7890 | ENSG00000180481 |  |
| 7891 | ENSG00000156970 |  |
| 7892 | ENSG00000217555 |  |
| 7893 | ENSG00000100246 |  |
| 7894 | ENSG00000182568 |  |
| 7895 | ENSG00000104499 |  |
| 7896 | ENSG00000198589 |  |
| 7897 | ENSG00000204516 |  |
| 7898 | ENSG00000178105 |  |
| 7899 | ENSG00000165233 |  |
| 7900 | ENSG00000132842 |  |
| 7901 | ENSG00000143786 |  |
| 7902 | ENSG00000106028 |  |
| 7903 | ENSG00000148362 |  |
| 7904 | ENSG00000103342 |  |
| 7905 | ENSG00000113269 |  |
| 7906 | ENSG00000037757 |  |
| 7907 | ENSG00000114395 |  |
| 7908 | ENSG00000183785 |  |
| 7909 | ENSG00000170779 |  |
| 7910 | ENSG00000036448 |  |
| 7911 | ENSG00000215193 |  |
| 7912 | ENSG00000259164 |  |
| 7913 | ENSG00000165118 |  |
| 7914 | ENSG00000101605 |  |
| 7915 | ENSG00000163463 |  |
| 7916 | ENSG00000267857 |  |
| 7917 | ENSG00000105650 |  |
| 7918 | ENSG00000257594 |  |
| 7919 | ENSG00000169062 |  |
| 7920 | ENSG00000103043 |  |
| 7921 | ENSG00000204866 |  |
| 7922 | ENSG00000214357 |  |
| 7923 | ENSG00000196260 |  |
| 7924 | ENSG00000092345 |  |
| 7925 | ENSG00000117640 |  |
| 7926 | ENSG00000254531 |  |
| 7927 | ENSG00000257028 |  |
| 7928 | ENSG00000114861 |  |
| 7929 | ENSG00000161929 |  |
| 7930 | ENSG00000162267 |  |
| 7931 | ENSG00000103024 |  |
| 7932 | ENSG00000114316 |  |
| 7933 | ENSG00000115459 |  |
| 7934 | ENSG00000114786 |  |
| 7935 | ENSG00000166387 |  |
| 7936 | ENSG00000138594 |  |
| 7937 | ENSG00000064666 |  |
| 7938 | ENSG00000174485 |  |
| 7939 | ENSG00000049283 |  |
| 7940 | ENSG00000213853 |  |
| 7941 | ENSG00000168566 |  |
| 7942 | ENSG00000095380 |  |
| 7943 | ENSG00000129173 |  |
| 7944 | ENSG00000273049 |  |
| 7945 | ENSG00000163499 |  |
| 7946 | ENSG00000174963 |  |
| 7947 | ENSG00000128245 |  |
| 7948 | ENSG00000179094 |  |
| 7949 | ENSG00000214109 |  |
| 7950 | ENSG00000100284 |  |
| 7951 | ENSG00000161572 |  |
| 7952 | ENSG00000159182 |  |
| 7953 | ENSG00000079435 |  |
| 7954 | ENSG00000188508 |  |
| 7955 | ENSG00000232653 |  |
| 7956 | ENSG00000197461 |  |
| 7957 | ENSG00000130487 |  |
| 7958 | ENSG00000131148 |  |
| 7959 | ENSG00000156966 |  |
| 7960 | ENSG00000135930 |  |
| 7961 | ENSG00000159208 |  |
| 7962 | ENSG00000214435 |  |
| 7963 | ENSG00000119514 |  |
| 7964 | ENSG00000166091 |  |
| 7965 | ENSG00000204003 |  |
| 7966 | ENSG00000263812 |  |
| 7967 | ENSG00000140307 |  |
| 7968 | ENSG00000070915 |  |
| 7969 | ENSG00000111786 |  |
| 7970 | ENSG00000109163 |  |
| 7971 | ENSG00000112769 |  |
| 7972 | ENSG00000181013 |  |
| 7973 | ENSG00000114115 |  |
| 7974 | ENSG00000128815 |  |
| 7975 | ENSG00000128322 |  |
| 7976 | ENSG00000089006 |  |
| 7977 | ENSG00000197858 |  |
| 7978 | ENSG00000157933 |  |
| 7979 | ENSG00000091137 |  |
| 7980 | ENSG00000120049 |  |
| 7981 | ENSG00000119720 |  |
| 7982 | ENSG00000013588 |  |
| 7983 | ENSG00000221883 |  |
| 7984 | ENSG00000129990 |  |
| 7985 | ENSG00000154767 |  |
| 7986 | ENSG00000214102 |  |
| 7987 | ENSG00000105738 |  |
| 7988 | ENSG00000105290 |  |
| 7989 | ENSG00000172339 |  |
| 7990 | ENSG00000197536 |  |
| 7991 | ENSG00000268818 |  |
| 7992 | ENSG00000008130 |  |
| 7993 | ENSG00000170927 |  |
| 7994 | ENSG00000203691 |  |
| 7995 | ENSG00000198612 |  |
| 7996 | ENSG00000129993 |  |
| 7997 | ENSG00000100227 |  |
| 7998 | ENSG00000110047 |  |
| 7999 | ENSG00000154237 |  |
| 8000 | ENSG00000125843 |  |
| 8001 | ENSG00000173626 |  |
| 8002 | ENSG00000087095 |  |
| 8003 | ENSG00000126453 |  |
| 8004 | ENSG00000158477 |  |
| 8005 | ENSG00000126262 |  |
| 8006 | ENSG00000185129 |  |
| 8007 | ENSG00000137726 |  |
| 8008 | ENSG00000232810 |  |
| 8009 | ENSG00000124145 |  |
| 8010 | ENSG00000137331 |  |
| 8011 | ENSG00000149557 |  |
| 8012 | ENSG00000124357 |  |
| 8013 | ENSG00000214595 |  |
| 8014 | ENSG00000142552 |  |
| 8015 | ENSG00000174233 |  |
| 8016 | ENSG00000198944 |  |
| 8017 | ENSG00000241644 |  |
| 8018 | ENSG00000161249 |  |
| 8019 | ENSG00000113594 |  |
| 8020 | ENSG00000108342 |  |
| 8021 | ENSG00000177426 |  |
| 8022 | ENSG00000177054 |  |
| 8023 | ENSG00000214866 |  |
| 8024 | ENSG00000104043 |  |
| 8025 | ENSG00000169894 |  |
| 8026 | ENSG00000196834 |  |
| 8027 | ENSG00000140043 |  |
| 8028 | ENSG00000249861 |  |
| 8029 | ENSG00000182534 |  |
| 8030 | ENSG00000135362 |  |
| 8031 | ENSG00000171368 |  |
| 8032 | ENSG00000181722 |  |
| 8033 | ENSG00000272214 |  |
| 8034 | ENSG00000183520 |  |
| 8035 | ENSG00000176994 |  |
| 8036 | ENSG00000100253 |  |
| 8037 | ENSG00000115091 |  |
| 8038 | ENSG00000188859 |  |
| 8039 | ENSG00000257727 |  |
| 8040 | ENSG00000117400 |  |
| 8041 | ENSG00000132589 |  |
| 8042 | ENSG00000111665 |  |
| 8043 | ENSG00000137513 |  |
| 8044 | ENSG00000099958 |  |
| 8045 | ENSG00000248511 |  |
| 8046 | ENSG00000213888 |  |
| 8047 | ENSG00000204237 |  |
| 8048 | ENSG00000103148 |  |
| 8049 | ENSG00000169242 |  |
| 8050 | ENSG00000163171 |  |
| 8051 | ENSG00000151320 |  |
| 8052 | ENSG00000213638 |  |
| 8053 | ENSG00000077713 |  |
| 8054 | ENSG00000196961 |  |
| 8055 | ENSG00000266953 |  |
| 8056 | ENSG00000149541 |  |
| 8057 | ENSG00000187726 |  |
| 8058 | ENSG00000197265 |  |
| 8059 | ENSG00000114383 |  |
| 8060 | ENSG00000163798 |  |
| 8061 | ENSG00000240654 |  |
| 8062 | ENSG00000204084 |  |
| 8063 | ENSG00000054796 |  |
| 8064 | ENSG00000204876 |  |
| 8065 | ENSG00000164405 |  |
| 8066 | ENSG00000151967 |  |
| 8067 | ENSG00000136878 |  |
| 8068 | ENSG00000266202 |  |
| 8069 | ENSG00000103460 |  |
| 8070 | ENSG00000268427 |  |
| 8071 | ENSG00000160993 |  |
| 8072 | ENSG00000157315 |  |
| 8073 | ENSG00000135127 |  |
| 8074 | ENSG00000142538 |  |
| 8075 | ENSG00000162302 |  |
| 8076 | ENSG00000184640 |  |
| 8077 | ENSG00000169189 |  |
| 8078 | ENSG00000066322 |  |
| 8079 | ENSG00000064199 |  |
| 8080 | ENSG00000255112 |  |
| 8081 | ENSG00000112584 |  |
| 8082 | ENSG00000166337 |  |
| 8083 | ENSG00000006327 |  |
| 8084 | ENSG00000139890 |  |
| 8085 | ENSG00000204128 |  |
| 8086 | ENSG00000063180 |  |
| 8087 | ENSG00000169683 |  |
| 8088 | ENSG00000187609 |  |
| 8089 | ENSG00000198286 |  |
| 8090 | ENSG00000003402 |  |
| 8091 | ENSG00000172817 |  |
| 8092 | ENSG00000103175 |  |
| 8093 | ENSG00000101220 |  |
| 8094 | ENSG00000178772 |  |
| 8095 | ENSG00000010270 |  |
| 8096 | ENSG00000269121 |  |
| 8097 | ENSG00000162065 |  |
| 8098 | ENSG00000228146 |  |
| 8099 | ENSG00000104936 |  |
| 8100 | ENSG00000163154 |  |
| 8101 | ENSG00000160877 |  |
| 8102 | ENSG00000182742 |  |
| 8103 | ENSG00000185220 |  |
| 8104 | ENSG00000163687 |  |
| 8105 | ENSG00000174013 |  |
| 8106 | ENSG00000153446 |  |
| 8107 | ENSG00000047056 |  |
| 8108 | ENSG00000141337 |  |
| 8109 | ENSG00000175514 |  |
| 8110 | ENSG00000088387 |  |
| 8111 | ENSG00000213918 |  |
| 8112 | ENSG00000196684 |  |
| 8113 | ENSG00000183103 |  |
| 8114 | ENSG00000204576 |  |
| 8115 | ENSG00000182158 |  |
| 8116 | ENSG00000105997 |  |
| 8117 | ENSG00000222047 |  |
| 8118 | ENSG00000003509 |  |
| 8119 | ENSG00000168675 |  |
| 8120 | ENSG00000198039 |  |
| 8121 | ENSG00000171603 |  |
| 8122 | ENSG00000065357 |  |
| 8123 | ENSG00000165526 |  |
| 8124 | ENSG00000090905 |  |
| 8125 | ENSG00000163635 |  |
| 8126 | ENSG00000112159 |  |
| 8127 | ENSG00000167306 |  |
| 8128 | ENSG00000143742 |  |
| 8129 | ENSG00000136811 |  |
| 8130 | ENSG00000079246 |  |
| 8131 | ENSG00000170667 |  |
| 8132 | ENSG00000164532 |  |
| 8133 | ENSG00000177600 |  |
| 8134 | ENSG00000066230 |  |
| 8135 | ENSG00000160471 |  |
| 8136 | ENSG00000143768 |  |
| 8137 | ENSG00000064225 |  |
| 8138 | ENSG00000179431 |  |
| 8139 | ENSG00000132259 |  |
| 8140 | ENSG00000065526 |  |
| 8141 | ENSG00000166619 |  |
| 8142 | ENSG00000259649 |  |
| 8143 | ENSG00000125503 |  |
| 8144 | ENSG00000173848 |  |
| 8145 | ENSG00000187642 |  |
| 8146 | ENSG00000222046 |  |
| 8147 | ENSG00000132692 |  |
| 8148 | ENSG00000131398 |  |
| 8149 | ENSG00000170370 |  |
| 8150 | ENSG00000062038 |  |
| 8151 | ENSG00000005483 |  |
| 8152 | ENSG00000123685 |  |
| 8153 | ENSG00000182983 |  |
| 8154 | ENSG00000088826 |  |
| 8155 | ENSG00000151917 |  |
| 8156 | ENSG00000241635 |  |
| 8157 | ENSG00000260371 |  |
| 8158 | ENSG00000129691 |  |
| 8159 | ENSG00000050165 |  |
| 8160 | ENSG00000146963 |  |
| 8161 | ENSG00000164078 |  |
| 8162 | ENSG00000159399 |  |
| 8163 | ENSG00000178026 |  |
| 8164 | ENSG00000228157 |  |
| 8165 | ENSG00000239961 |  |
| 8166 | ENSG00000198768 |  |
| 8167 | ENSG00000074054 |  |
| 8168 | ENSG00000187736 |  |
| 8169 | ENSG00000219435 |  |
| 8170 | ENSG00000149257 |  |
| 8171 | ENSG00000131504 |  |

**Supplementary Table 2.** Functional enrichment analysis of the regulated genes of cis-acting SE-lncRNAs.

| Biological Process | Total  Count | Our  Data | Fold | FDR |
| --- | --- | --- | --- | --- |
| transcription by RNA polymerase II | 899 | 412 | 1.25 | 7.98E-03 |
| regulation of metabolic process | 1401 | 602 | 1.18 | 3.38E-02 |
| innate immune response | 173 | 29 | 0.46 | 2.03E-03 |
| phagocytosis | 121 | 17 | 0.38 | 4.22E-03 |
| adaptive immune response | 139 | 18 | 0.35 | 4.35E-04 |
| defense response to bacterium | 125 | 15 | 0.33 | 4.46E-04 |
| immune effector process | 138 | 14 | 0.28 | 1.27E-05 |
| positive regulation of lymphocyte activation | 107 | 10 | 0.26 | 1.72E-04 |
| detection of chemical stimulus involved in sensory perception | 207 | 12 | 0.16 | 3.59E-13 |
| B cell receptor signaling pathway | 102 | 5 | 0.13 | 9.43E-07 |
| humoral immune response | 111 | 5 | 0.12 | 1.14E-07 |
| B cell activation | 95 | 3 | 0.09 | 1.92E-07 |
| keratinocyte differentiation | 34 | 1 | 0.08 | 2.28E-02 |

FDR=false discovery rate adjusted by Benjamini-Hochberg method. Total Count=all genes in the corresponding GO term. Our Data=the regulated genes of cis-acting SE-lncRNAs identified by this study. Fold=fold enrichment.

**Supplementary Table 3.** Functional enrichment analysis of the regulated genes of trans-acting SE-lncRNAs.

| Biological Process | Total  Count | Our  Data | Fold | FDR |
| --- | --- | --- | --- | --- |
| protein autophosphorylation | 38 | 22 | 2.77 | 2.23E-02 |
| enzyme linked receptor protein signaling pathway | 255 | 83 | 1.56 | 3.05E-02 |
| homeostatic process | 304 | 95 | 1.5 | 3.82E-02 |
| intracellular signal transduction | 1025 | 278 | 1.3 | 6.00E-03 |
| developmental process | 1035 | 271 | 1.25 | 3.74E-02 |
| regulation of macromolecule metabolic process | 1195 | 307 | 1.23 | 4.71E-02 |
| cellular component organization | 2085 | 512 | 1.18 | 3.04E-02 |
| innate immune response | 173 | 11 | 0.3 | 6.12E-04 |
| B cell receptor signaling pathway | 102 | 3 | 0.14 | 5.39E-04 |
| adaptive immune response | 139 | 4 | 0.14 | 1.38E-05 |
| positive regulation of lymphocyte activation | 107 | 3 | 0.13 | 2.82E-04 |
| humoral immune response | 111 | 3 | 0.13 | 2.15E-04 |
| immune effector process | 138 | 3 | 0.1 | 3.92E-06 |
| B cell activation | 95 | 1 | 0.05 | 5.29E-05 |
| defense response to bacterium | 125 | 1 | 0.04 | 6.24E-07 |
| detection of chemical stimulus involved in sensory perception | 207 | 1 | 0.02 | 9.15E-13 |

FDR=false discovery rate adjusted by Benjamini-Hochberg method. Total Count=all genes in the corresponding GO term. Our Data=the regulated genes of trans-acting SE-lncRNAs identified by this study. Fold=fold enrichment.
